# The Japanese Critical Care Nutrition Guideline 2024

**DOI:** 10.1186/s40560-025-00785-z

**Published:** 2025-03-21

**Authors:** Kensuke Nakamura, Ryo Yamamoto, Naoki Higashibeppu, Minoru Yoshida, Hiroomi Tatsumi, Yoshiyuki Shimizu, Hiroo Izumino, Taku Oshima, Junji Hatakeyama, Akira Ouchi, Rie Tsutsumi, Norihiko Tsuboi, Natsuhiro Yamamoto, Ayumu Nozaki, Sadaharu Asami, Yudai Takatani, Kohei Yamada, Yujiro Matsuishi, Shuhei Takauji, Akihito Tampo, Yusuke Terasaka, Takeaki Sato, Saiko Okamoto, Hideaki Sakuramoto, Tomoka Miyagi, Keisei Aki, Hidehito Ota, Taro Watanabe, Nobuto Nakanishi, Hiroyuki Ohbe, Chihiro Narita, Jun Takeshita, Masano Sagawa, Takefumi Tsunemitsu, Shinya Matsushima, Daisuke Kobashi, Yorihide Yanagita, Shinichi Watanabe, Hiroyasu Murata, Akihisa Taguchi, Takuya Hiramoto, Satomi Ichimaru, Muneyuki Takeuchi, Joji Kotani

**Affiliations:** 1 Department of Critical Care Medicine, Yokohama City University Hospital, 3-9 Fukuura, Kanazawa-ku, Yokohama, Kanagawa 236-0004 Japan; 2 Department of Emergency and Critical Care Medicine, Keio University School of Medicine, Tokyo, Japan; 3 Department of Anesthesia and Critical Care, Kobe City Medical Center General Hospital, Kobe, Japan; 4 Department of Emergency and Critical Care Medicine, St. Marianna University School of Medicine, Kanagawa, Japan; 5 Department of Intensive Care Medicine, Sapporo Medical University School of Medicine, Sapporo, Japan; 6 Department of Intensive Care Medicine, Osaka Women’s and Children’s Hospital, Osaka, Japan; 7 Acute and Critical Care Center, Nagasaki University Hospital, Nagasaki, Japan; 8 Department of Emergency and Critical Care Medicine, Chiba University Graduate School of Medicine, Chiba City, Japan; 9 Department of Emergency and Critical Care Medicine, Osaka Medical and Pharmaceutical University, Osaka, Japan; 10 Department of Adult Health Nursing, College of Nursing, Ibaraki Christian University, Hitachi, Japan; 11 Department of Anesthesiology and Critical Care, Hiroshima University Hospital, Hiroshima, Japan; 12 Department of Critical Care Medicine and Anesthesia, National Center for Child Health and Development, Tokyo, Japan; 13 Department of Anesthesiology and Critical Care Medicine, Yokohama City University School of Medicine, Kanagawa, Japan; 14 Department of Pharmacy, Kyoto-Katsura Hospital, Kyoto, Japan; 15 Department of Cardiology, Musashino Tokushukai Hospital, Tokyo, Japan; 16 Department of Primary Care and Emergency Medicine, Kyoto University Hospital, Kyoto, Japan; 17 Department of Traumatology and Critical Care Medicine, National Defense Medical College Hospital, Saitama, Japan; 18 Adult and Elderly Nursing, Faculty of Nursing, Tokyo University of Information Science, Chiba, Japan; 19 Department of Emergency Medicine, Hokkaido University Hospital, Sapporo, Japan; 20 Department of Emergency Medicine, Asahikawa Medical University, Asahikawa, Japan; 21 Department of Emergency Medicine, Kyoto Katsura Hospital, Kyoto, Japan; 22 Tohoku University Hospital Emergency Center, Miyagi, Japan; 23 Department of Nursing, Hitachi General Hospital, Hitachi, Japan; 24 Department of Acute Care Nursing, Japanese Red Cross Kyushu International College of Nursing, Munakata, Japan; 25 Anesthesiology and Critical Care Medicine, Master’s Degree Program, Graduate School of Medicine, Yokohama City University, Kanagawa, Japan; 26 Department of Pharmacy, Kokura Memorial Hospital, Fukuoka, Japan; 27 Department of Pediatrics, School of Medicine, The University of Tokyo, Tokyo, Japan; 28 Department of Intensive Care Medicine, Showa University School of Medicine, Tokyo, Japan; 29 Division of Disaster and Emergency Medicine, Department of Surgery Related, Kobe University Graduate School of Medicine, Kobe, Japan; 30 Department of Emergency and Critical Care Medicine, Tohoku University Hospital, Sendai, Japan; 31 Department of Emergency Medicine, Shizuoka General Hospital, Shizuoka, Japan; 32 Department of Anesthesiology, Osaka Women’s and Children’s Hospital, Izumi, Japan; 33 Department of Surgery, Tokyo Women’s Medical University Adachi Medical Center, Tokyo, Japan; 34 Department of Preventive Services, Graduate School of Medicine, Kyoto University, Kyoto, Japan; 35 Department of Physical Therapy, Faculty of Health Science, Kyorin University, Tokyo, Japan; 36 Department of Critical Care and Emergency Medicine, Japanese Red Cross Maebashi Hospital, Gunma, Japan; 37 Department of Health Sciences, Institute of Biomedical Sciences, Nagasaki University, Nagasaki, Japan; 38 Department of Physical Therapy, Faculty of Rehabilitation, Gifu University of Health Science, Gifu, Japan; 39 Department of Rehabilitation Medicine, Kyorin University Hospital, Tokyo, Japan; 40 Department of Anesthesia, Kyoto University Hospital, Kyoto, Japan; 41 Department of Internal Medicine, Tokyo Bay Urayasu Ichikawa Medical Center, Urayasu, Japan; 42 Food and Nutrition Service Department, Fujita Health University Hospital, Aichi, Japan; 43 Department of Critical Care Medicine, National Cerebral and Cardiovascular Center, Osaka, Japan

**Keywords:** ICU, Nutrition, Critical illness, Guideline

## Abstract

Nutrition therapy is important in the management of critically ill patients and is continuously evolving as new evidence emerges. The Japanese Critical Care Nutrition Guideline 2024 (JCCNG 2024) is specific to Japan and is the latest set of clinical practice guidelines for nutrition therapy in critical care that was revised from JCCNG 2016 by the Japanese Society of Intensive Care Medicine. An English version of these guidelines was created based on the contents of the original Japanese version. These guidelines were developed to help health care providers understand and provide nutrition therapy that will improve the outcomes of children and adults admitted to intensive care units or requiring intensive care, regardless of the disease. The intended users of these guidelines are all healthcare professionals involved in intensive care, including those who are not familiar with nutrition therapy. JCCNG 2024 consists of 37 clinical questions and 24 recommendations, covering immunomodulation therapy, nutrition therapy for special conditions, and nutrition therapy for children. These guidelines were developed in accordance with the Grading of Recommendations, Assessment, Development and Evaluation (GRADE) system by experts from various healthcare professionals related to nutrition therapy and/or critical care. All GRADE-based recommendations, good practice statements (GPS), future research questions, and answers to background questions were finalized by consensus using the modified Delphi method. Strong recommendations for adults include early enteral nutrition (EN) within 48 h and the provision of pre/synbiotics. Weak recommendations for adults include the use of a nutrition protocol, EN rather than parenteral nutrition, the provision of higher protein doses, post-pyloric EN, continuous EN, omega-3 fatty acid-enriched EN, the provision of probiotics, and indirect calorimetry use. Weak recommendations for children include early EN within 48 h, bolus EN, and energy/protein-dense EN formulas. A nutritional assessment is recommended by GPS for both adults and children. JCCNG 2024 will be disseminated through educational activities mainly by the JCCNG Committee at various scientific meetings and seminars. Since studies on nutritional treatment for critically ill patients are being reported worldwide, these guidelines will be revised in 4 to 6 years. We hope that these guidelines will be used in clinical practice for critically ill patients and in future research.

**Supplementary Information:**

The online version contains supplementary material available at 10.1186/s40560-025-00785-z.

## Introduction

Critically ill patients are at risk of significant nutritional disorders due to many factors, including diseases and their treatment, intensive care unit (ICU) care, and physical and psychiatric restrictions [1]. Since patients are often unable to take food orally on their own, care providers must design and provide nutrition therapy. Inappropriate nutrition therapy may contribute to the worsening of a patient’s prognosis via nutritional disorders [2]; therefore, evidence-based nutrition therapy in the acute phase is needed in clinical practice. In Japan, nutrition therapy guidelines for critically ill patients, the predecessor to the present guidelines, were developed in 2016 [3].

The European Society for Clinical Nutrition and Metabolism (ESPEN) and the American Society for Parenteral and Enteral Nutrition (ASPEN) published guidelines for nutrition therapy for critically ill patients to promote appropriate clinical practice and research [4, 5]. However, some of the recommendations markedly differ between these guidelines, and may not be commensurate with the realities and issues of clinical nutrition in different countries [6, 7]. Since the heterogeneity of ICU patients is very large [8], careful considerations by both the authors and readers are needed to ensure the correct interpretation and clinical application of these and the present guidelines. In Japan, the additional fee for early nutrition intervention and management in insurance reimbursement requires that nutrition therapy be implemented in accordance with the present guidelines and, thus, it is important to present recommendations commensurate with this stipulation.

In addition, it has been suggested in recent critical care that attention needs to be paid not only to mortality and the duration of ventilation, but also to long-term prognosis and physical dysfunction after critical care, called Post-Intensive Care Syndrome (PICS) [9]. While one of the targets of nutrition is to maintain body homeostasis, physical function and muscle mass volume also need to be assessed as outcomes of nutrition therapy [10], and are being adopted by an increasing number of randomized control trials (RCTs) [11]. These guidelines present the most up-to-date summary recommendations in that they include physical function as one of the primary outcomes for the first time.

The guidelines also introduce the Grading of Recommendations, Assessment, Development and Evaluation (GRADE) system, which was not possible in the previous edition^3^. Multidisciplinary experts developed answers to 37 relevant clinical questions (CQs) based on the latest systematic reviews and meta-analyses. These efforts differentiate Japanese Critical Care Nutrition Guideline 2024 (JCCNG 2024) from the current international guidelines [4, 5]. The purpose of these guidelines is to summarize the latest evidence on nutrition therapy for critically ill patients from a new perspective and to guide clinical practice.

## Methods

### Basic philosophy and overview

#### Name and purpose

JCCNG 2024 was developed to help healthcare providers understand and provide nutrition therapy that will improve the outcomes of patients, such as mortality, length of ICU stay, length of mechanical ventilation use, adverse events, and physical functions.

#### Target population and users

The target population includes children and adults admitted to ICUs or requiring intensive care, regardless of the type of disease. The intended users of these guidelines are all healthcare professionals involved in intensive care, including those who are not familiar with nutrition therapy.

#### Relationship with other guidelines

ASPEN [5] and ESPEN [4] have published guidelines on nutrition therapy for critically ill patients. However, there are still important CQs outside these guidelines, and the feasibility of nutrition therapy differs depending on countries and regions due to differences in healthcare systems. Therefore, it is important that guidelines for nutrition therapy using the GRADE approach are provided in Japan. These guidelines provide 37 CQs and 24 recommendations, covering immunomodulation therapy, nutrition therapy for special conditions, and nutrition therapy for children. It is important to note that these recommendations are not intended to limit any treatment or the management of patients.

#### Organization

JCCNG 2024 was developed in accordance with the GRADE system by a process of developing CQs; searching, collecting, and integrating evidence by a systematic review; evaluating the certainty of evidence (CoE); and formulating recommendations. Experts from various healthcare fields related to nutrition therapy and/or critical care were gathered, including physicians specialized in critical care, emergency medicine, surgery, internal medicine, pediatrics, and anesthesiology, a nurse, dietitian, pharmacist, and physical therapist. A guideline development group (GDG), working groups, systematic review teams, and a methodological support team were then established.

The GDG, named the JCCNG Committee, was established and approved by the Japanese Society of Intensive Care Medicine. The GDG developed and decided on the scope, CQs, and recommendations in the guidelines. The working group comprised members of the Japanese Society of Intensive Care Medicine who were recruited or nominated by the GDG, while some members of the GDG also served as a member of the working group. The working group supported the development of CQs, supervised the systematic review, and drafted the recommendations. Systematic review teams were recruited from members of the Japanese Society of Intensive Care Medicine and conducted the systematic review. The methodological support team comprised physicians and nurses appointed by the GDG, and educated and supported members of the working groups and systematic review teams by explaining the GRADE approach and preparing academic materials from a neutral standpoint.

#### Quality and transparency control

After a draft of the guidelines was formulated, it was peer-reviewed by external evaluators using AGREE II and revised according to their comments and suggestions. To ensure quality and transparency, the contents of the guidelines were peer-reviewed by the members of the GDG and public comments were obtained from several academic organizations. In addition, the drafting processes were disclosed to all members involved in the guideline development process, and all meetings of the GDG were held in public.

#### Plans for dissemination and revision

**Fig. 1:**
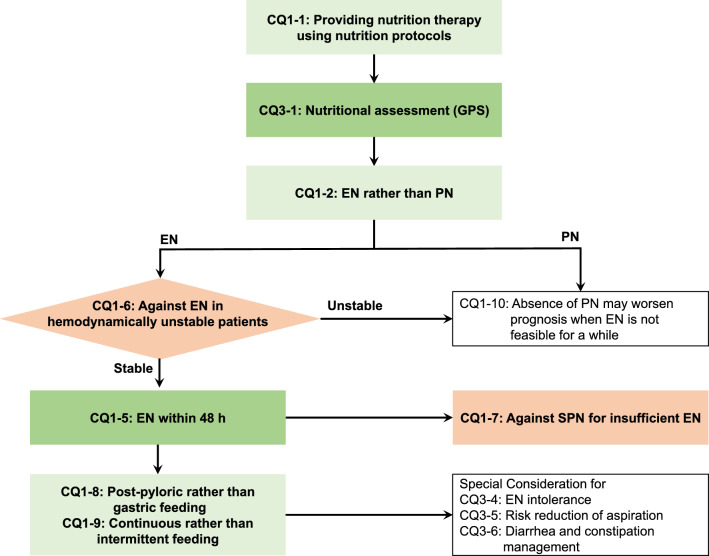
Nutrition delivery routes and methods for critically ill adult patients. Nutrition delivery routes and methods for critically ill adult patients answered in the guidelines are shown as a flowchart of clinical practice. Green boxes indicate answers recommended, red boxes indicate answers not recommended, and white boxes indicate answers for background questions. CQ: clinical question; GPS: good practice statements; EN: enteral nutrition; PN: parenteral nutrition; SPN: supplemental parenteral nutrition

**Fig. 2:**
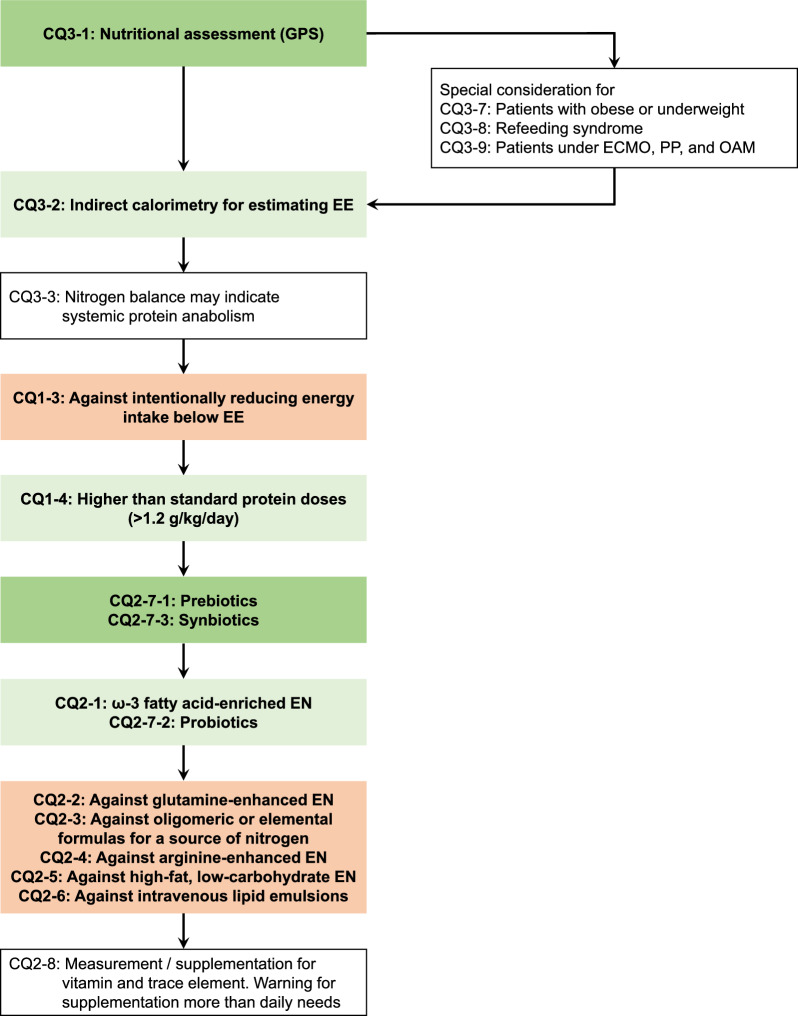
Nutrition amounts and nutrients for critically ill adult patients. Nutrition amounts and nutrients for critically ill adult patients answered in the guidelines are shown as a flowchart of clinical practice. Green boxes indicate answers recommended, red boxes indicate answers not recommended, and white boxes indicate answers for background questions. CQ: clinical question; GPS: good practice statements; ECMO: extracorporeal membrane oxygenation; PP: prone position; OAM: open abdomen management; EE: energy expenditure

**Fig. 3:**
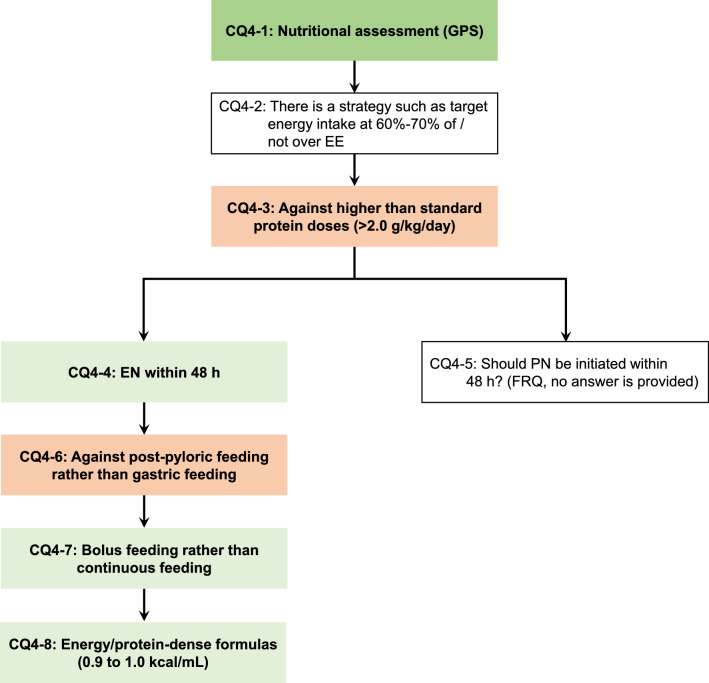
Nutrition therapy for critically ill pediatric patients. Nutrition therapy for critically ill pediatric patients answered in the guidelines is shown as a flowchart of clinical practice. Green boxes indicate answers recommended, red boxes indicate answers not recommended, and white boxes indicate answers for background questions. CQ: clinical question; GPS: good practice statements; EE: energy expenditure; EN: enteral nutrition; PN: parenteral nutrition; FRQ: future research question

Flowcharts to clinical practice (Figs. 1, 2, 3) and a quick reference list of CQs and answers (Table 1) based on JCCNG 2024 are shown. JCCNG 2024 will be disseminated by educational activities mainly by the JCCNG Committee at various scientific meetings and seminars. The dissemination of an application that includes the contents of the guidelines are also planned. In addition, monitoring the degree of the clinical adaptation of JCCNG 2024 using a questionnaire by the committee is planned.

**Table 1:** Quick reference list of CQs and answers

WG1. General principles of nutrition therapy	
CQ 1-1: Should nutrition therapy using nutrition protocols be provided in critically ill patients?	*Answer*: We suggest providing nutrition therapy using nutrition protocols to critically ill patients. (GRADE 2B: certainty of evidence = “moderate”)
CQ1-2: Should enteral nutrition be administered rather than parenteral nutrition in critically ill patients?	*Answer*: We suggest administering enteral nutrition rather than parenteral nutrition in critically ill patients. (GRADE 2C: certainty of evidence = “low”)
CQ1-3: Should energy provision be intentionally reduced below energy expenditure in the acute phase of critical illness?	*Answer*: We suggest against intentionally reducing energy intake below energy expenditure in the acute phase of critical illness (GRADE 2B: certainty of evidence = “moderate”)
CQ1-4: Should higher dose of protein above the standard (> 1.2 g/kg/day) be administered in critically ill patients?	*Answer*: We suggest administering higher protein doses above the standard (> 1.2 g/kg/day) in critically ill patients (GRADE 2D: certainty of evidence = “very low”)
CQ1-5: Should enteral nutrition be initiated within 48 h following the start of treatment in critically ill patients?	*Answer*: We recommend initiating enteral nutrition within 48 h following the start of treatment in critically ill patients. (GRADE 1B: certainty of evidence = “moderate”)
CQ1-6: Is enteral nutrition beneficial compared to no enteral nutrition or parenteral nutrition in critically ill patients with hemodynamic instability?	*Answer*: We suggest against administering enteral nutrition in critically ill patients with hemodynamic instability (GRADE 2D: certainty of evidence = “very low”)
CQ1-7: Should supplemental parenteral nutrition be initiated in critically ill patients receiving insufficient amounts of enteral nutrition?	*Answer*: We suggest against initiating supplemental parenteral nutrition in critically ill patients receiving insufficient amounts of enteral nutrition (GRADE 2A: certainty of evidence = “high”)
CQ1-8: Should post-pyloric feeding be used rather than gastric feeding for enteral nutrition in critically ill patients?	*Answer*: We suggest using post-pyloric feeding rather than gastric feeding for enteral nutrition in critically ill patients (GRADE 2D: certainty of evidence = “very low”)
CQ1-9: Should continuous feeding rather than intermittent feeding be used for gastric enteral nutrition in critically ill patients?	*Answer*: We suggest continuous feeding rather than intermittent feeding for gastric enteral nutrition in critically ill patients (GRADE 2D: certainty of evidence = “very low”)
CQ1-10: What is parenteral nutrition strategy in critically ill patients for whom enteral nutrition is not feasible?	*Answer*: In critically ill patients for whom enteral nutrition is not feasible for a while, the absence of parenteral nutrition, the only means of nutrition therapy, may worsen the prognosis (provision of information for background question)
WG2. Specific nutrients in nutrition therapy	
CQ2-1: Should omega-3 fatty acid-enriched enteral nutrition be administered to critically ill patients?	*Answer*: We suggest administering omega-3 fatty acid-enriched enteral nutrition to critically ill patients (GRADE 2C: certainty of evidence = “low”)
CQ2-2: Should glutamine-enhanced enteral nutrition be administered in critically ill patients?	*Answer*: We suggest against administering glutamine-enhanced enteral nutrition in critically ill patients receiving enteral nutrition (GRADE 2D: certainty of evidence = “very low”)
CQ2-3: Should enteral nutrition with oligomeric or elemental formulas rather than food-based blenderized formulas or polymeric formulas be used in critically ill patients?	*Answer*: We suggest against administering enteral nutrition with oligomeric or elemental formulas intended as a source of nitrogen in critically ill patients (GRADE 2D, certainty of evidence = “very low”)
CQ2-4: Should arginine-enhanced enteral nutrition be administered in critically ill patients?	*Answer*: We suggest against administering arginine-enhanced enteral nutrition in critically ill patients receiving enteral nutrition (GRADE 2D: certainty of evidence = “very low”)
CQ2-5: Should high-fat, low-carbohydrate enteral nutrition be used for critically ill patients?	*Answer*: We suggest against administering high-fat, low-carbohydrate enteral nutrition for critically ill patients (GRADE 2C: certainty of evidence = “low”)
CQ2-6: Should intravenous lipid emulsions be administered to critically ill patients receiving parenteral nutrition?	*Answer*: We suggest against the intravenous administration of lipid emulsions to critically ill patients receiving parenteral nutrition (GRADE 2D, certainty of evidence = “very low”)
CQ2-7-1: Should prebiotics be administered in critically ill patients?	*Answer*: We recommend administering prebiotics to critically ill patients (GRADE 1B: certainty of evidence = “moderate”)
CQ2-7-2: Should probiotics be administered in critically ill patients?	*Answer*: We suggest administering probiotics to critically ill patients (GRADE 2C: certainty of evidence = “low”)
CQ2-7-3: Should synbiotics be administered in critically ill patients?	*Answer*: We recommend the administration of synbiotics in critically ill patients (GRADE 1C: certainty of evidence = “low”)
CQ2-8: What is the concept of vitamin and trace element supplementation in nutrition therapy for critically ill patients?	*Answer*: Critically ill patients are at high risk for vitamin and trace element deficiencies, and appropriate measurement and supplementation should be considered for them. Unless a severe deficiency is suspected, active supplementation beyond the daily requirements should be warned (provision of information for background question)
WG3. Nutrition monitoring and specific conditions	
CQ3-1: Is a nutritional assessment necessary before providing nutrition therapy to critically ill patients?	*Answer*: Nutritional assessment is necessary before providing nutrition therapy to critically ill patients (Good Practice Statement)
CQ3-2: Should indirect calorimetry be used to estimate energy expenditure in critically ill patients?	*Answer*: We suggest using indirect calorimetry for estimating energy expenditure (EE) in critically ill patients (GRADE 2B: certainty of evidence = “moderate”)
CQ3-3: What is the role of nitrogen balance in critically ill patients?	*Answer*: Nitrogen balance reflects the increase or decrease of protein in the body and may be an indicator to assess systemic protein anabolism (provision of information for background question)
CQ3-4: How is enteral feeding intolerance assessed in critically ill patients?	*Answer*: Enteral feeding intolerance is assessed using a combination of gastric residual volume, gastric residue properties, abdominal physical findings, imaging findings such as abdominal ultrasonography and abdominal radiographs, and lactate levels (provision of information for background question)
CQ3-5: How can the risk of aspiration be reduced in critically ill patients receiving enteral nutrition?	*Answer*: Methods to reduce the risk of aspiration include continuous feeding, post-pyloric feeding, adjusting the patient's position, and pharmacological interventions (provision of information for background question)
CQ3-6: How can diarrhea and constipation be managed in critically ill patients?	*Answer*: There are several methods, including the selection of nutrition formulas and administration methods, pharmacotherapy, and the use of bowel management systems (provision of information for background question)
CQ3-7: What is the approach to nutrition therapy for critically ill patients who are obese or underweight?	*Answer*: For critically ill patients who are obese or underweight, nutrition therapy will be individually determined based on patient’s condition, including energy and protein targets according to actual body weight, ideal body weight, or adjusted body weight (provision of information for background question)
CQ3-8: What is the concept of nutrition therapy for refeeding syndrome in critically ill patients?	*Answer*: With a risk assessment specific to refeeding syndrome (RFS), energy restriction and electrolyte monitoring with correction are considered based on the risks of RFS, post-onset symptoms, and electrolyte abnormalities (provision of information for background question)
CQ3-9: What is the approach to nutrition therapy for critically ill patients undergoing special treatments such as extracorporeal membrane oxygenation (ECMO), prone position (PP), and open abdomen management (OAM)?	*Answer*: For critically ill patients undergoing special treatments such as ECMO, PP, and OAM, appropriate nutrition therapy, including early enteral nutrition, will be provided based on the pathophysiology, disease progression, and gastrointestinal tract status (provision of information for background question)
WG4. Nutrition therapy in pediatrics	
CQ4-1: Should nutritional assessment be performed in critically ill pediatric patients?	*Answer*: Nutritional assessment should be performed in critically ill pediatric patients (Good Practice Statement)
CQ4-2: What is the strategy for energy intake in the acute phase of treatment of critically ill pediatric patients?	*Answer*: In the acute phase of treatment of critically ill pediatric patients, there is a strategy that target energy intake is set at approximately 60% to 70% of the energy expenditure or does not exceed the energy expenditure (provision of information for background question)
CQ4-3: Should higher than standard protein doses (> 2.0 g/kg/day) be given to critically ill pediatric patients?	*Answer*: We suggest against giving higher than standard protein doses (> 2.0 g/kg/day) to critically ill pediatric patients (GRADE 2D: certainty of evidence = “very low”)
CQ4-4: Should enteral nutrition be initiated within 48 h of starting treatment for critically ill pediatric patients?	*Answer*: We suggest initiating enteral feeding within 48 h of starting treatment for critically ill pediatric patients (Grade 2D, certainty of evidence = “very low”)
CQ4-5: Should parenteral nutrition be initiated within 48 h of starting treatment for critically ill pediatric patients? (FRQ)	
CQ4-6: Should post-pyloric feeding rather than gastric feeding be started for enteral nutrition of critically ill pediatric patients?	*Answer*: We suggest against using post-pyloric feeding rather than gastric feeding for enteral nutrition of critically ill pediatric patients (GRADE 2D: certainty of evidence = “very low”)
CQ4-7: Should bolus feeding rather than continuous feeding be used in critically ill pediatric patients undergoing gastric feeding?	*Answer*: We suggest using bolus feeding rather than continuous feeding in critically ill pediatric patients undergoing gastric feeding (GRADE 2C, certainty of evidence = “low”)
CQ4-8: Should energy/protein-dense formulas be administered to critically ill pediatric patients who are receiving enteral nutrition?	*Answer*: We suggest administering of energy/protein-dense formulas (0.9 to 1.0 kcal/mL) to critically ill pediatric patients (Grade 2D, certainty of evidence = “very low”)

Since studies on nutritional treatment for critically ill patients are continuously reported worldwide, JCCNG 2024 will be revised in 4 to 6 years. When critical evidence is published, the GDG will discuss the necessity for revisions and will revise as needed.

#### Facilitators and barriers of application

In Japan, a new medical claim for early nutrition therapy has been introduced highlighting the importance of nutrition therapy for critically ill patients across numerous health care institutions, which will facilitate the application of JCCNG 2024. Conversely, the lack of designated dietitians in hospitals will hinder the clinical adaptation of recommendations in these guidelines. Therefore, the nationwide allocation of certified dietitians who support nutrition therapy needs to be promoted.

#### Conflicts of interest (COI) and funds

All members of the GDG, working groups, systematic review teams, and a methodological support team declare their economic and academic COI in accordance with the COI Management Guidelines of the Japanese Association of Medical Sciences. The guidelines were developed with funds from the Japanese Society of Intensive Care Medicine, but do not reflect the intentions or interests of the society and no members received any compensation.

### Methodology for the development of clinical practice guidelines

#### Principle of guideline development

Following “Minds Manual for Guidelines Development 2020” [12], recommendations were drafted for each CQ based on the findings of a systematic review. Each recommendation was ultimately selected based on an evaluation of the balance between benefits and risks obtained by a systematic review, patients’ interests and conditions, medical economics, and social backgrounds.

#### CQ planning and classification

In CQ planning, fundamental knowledge of nutrition therapy is thought to support physicians providing standard nutrition therapy for critically ill patients. Basic rules for CQ development include the following: (1) clinically important questions need to be selected regardless of high-quality evidence; (2) among CQs in the published international guidelines, questions requiring an updated search on and the collection and integration of evidence need to be selected; and (3) CQs need to be in the form of a question and presented in the Patients, Intervention, Control, and Outcome (PICO) framework if they are foreground questions (FQs).

CQs were classified into two categories: background questions (BQs) providing information rather than recommendations, and FQs providing recommendations (Table 2). The recommendations in FQs are further classified into two categories: GRADE recommendations developed based on evidence that is obtained by systematic reviews, and GPS selected by the GDG when the benefits of the intervention clearly outweigh its risks, a RCT is not ethically feasible, and the GDG decides to make a strong recommendation. FQs for which sufficient evidence was not obtained were shown as future research questions (FRQs), and no recommendation was made.

**Table 2:** Classification of a clinical question

Background question (BQ)	Standard knowledge is presented and information is provided; recommendations are not presented
Foreground question (FQ)	
GRADE Recommendation	A recommendation is presented based on evidence obtained from a systematic review in accordance with the GRADE system
Good practice statement (GPS)	A recommendation is selected by the GDG when the benefits of the intervention clearly outweigh its risks, a randomized controlled trial is not ethically feasible, and the GDG decides to make a strong recommendation
Future research question (FRQ)	A clinically important question with insufficient evidence to create a recommendation; no recommendation is presented

GRADE, Grading of Recommendations, Assessment, Development and Evaluation

CQs were allocated to four working groups: 1. General principles of nutrition therapy, 2. Specific nutrients in nutrition therapy, 3. Nutrition monitoring and specific conditions, 4. Nutrition therapy in pediatrics. Each working group prepared a draft of sentences for CQs, which was then approved by the GDG. PICO was confirmed by the same processes, in which the outcome of PICO was unified for all FQs. Details on the outcome adopted into PICO and its level of importance were selected by consensus of the GDG (Table 3).

**Table 3:** Basic outcomes adopted in JCCNG 2024

Outcome number	Contents	Benefit/harm	Priority scale
O1	Mortality	Benefit	9
O2	Length of ICU stay	Benefit	8
O3	Length of ventilator use	Benefit	8
O4	Infectious disease complications	Harm	8
O5	Physical function assessment at discharge or up to 1 year after discharge (ADL, QOL, BI, FIM, muscle strength, SPPB, 6-min walk distance, SF-36/8, and EQ5D5L)	Benefit	8
O6	Changes in muscle mass during hospitalization or after discharge (assessment using anthropometry, echocardiography, CT, and a bioelectrical impedance analysis)	Benefit	7
O7	All adverse events	Harm	7

JCCNG, Japanese Critical Care Nutrition Guideline; ADL, Activities of Daily Living; QOL, Quality of Life; BI, Barthel Index; FIM, Functional Independence Measure; SPPB, Short Physical Performance Battery; SF-36/8, MOS 36/8-Item Short-Form Health Survey; EQ5D5L, EuroQol-5 Dimensions-5Level

Answers to BQs were described as an explanation of fundamental knowledge using published international guidelines, previous systematic reviews, and statements from academic organizations. GPS for FQs were developed by each working group, and GRADE recommendations were developed by a search for and the collection and synthesis of evidence using a systematic review and an evaluation of CoE.

#### Literature search and data extraction

Articles were searched in CENTRAL, PubMed, and Ichushi, a database for Japanese articles, in April 2023, and those published until the day of the search were targeted. The search strategy was developed by the systematic review team using Medical Subject Headings terms and free search terms, and was supervised by the working group. Target articles were limited to RCTs written in English or Japanese. The search strategy was finalized after confirming that the predefined important articles were appropriately included. The date when the literature search was conducted and the number of papers searched in each database were recorded, and bibliographic information was obtained.

After excluding duplicates of the retrieved references, two or more members in the systematic review team independently conducted primary screening; references that matched PICO were screened from titles and abstracts using Rayyan (https://rayyan.qcri.org/welcome).

The secondary screening of the selected articles was independently conducted by two or more members in the systematic review team, in which the full text of the articles was obtained and eligibility based on the study design and PICO was evaluated. Any disagreements were discussed by a third member in the systematic review team or working group members. Reasons for exclusion were recorded in each step and summarized in a PRISMA flow diagram.

#### Data extraction and bias risk assessment

Data extraction was performed by two or more members in the systematic review team using a standardized data extraction form. The study design, inclusion and exclusion criteria, the number of patients, interventions and controls, and outcomes were extracted for each study. No inquiries to the original authors were conducted.

The risk of bias was assessed by two or more members in the systematic review team. In each study and outcome, the risk of bias was assessed in five domains according to the RoB 2.0 methodology. The potential of bias was evaluated as high, low, or unclear, and the reason was noted for a high potential of bias. The risk of bias is summarized in the risk-of-bias table.

#### Meta-analysis and CoE

A meta-analysis of extracted outcome data was performed using Revman 5. CoE, which encompasses the findings of a meta-analysis, was assessed for each outcome based on the GRADE approach. The risk of bias, indirectness, inconsistency, imprecision, and publication bias were evaluated, and CoE was then rated as high, moderate, low, and very low. The findings obtained were summarized as an evidence profile using Guideline Development Tool (GDT) online software (http://gdt.guidelinedevelopment.org). CoE was estimated as high, downgraded if there were serious limitations in each item, and ultimately classified into high, medium, low, and very low (Table 4).

**Table 4:** Definition of certainty of evidence

High	Strong confidence in effect estimates
Moderate	Moderate confidence in effect estimates
Low	Limited confidence in effect estimates
Very low	Unreliable estimates of effect estimates

#### Development of recommendations and consensus building

Based on the evidence profile generated by the systematic review, the working group developed GRADE recommendations using the Evidence to Decision (EtD) table. This table evaluates benefits and risks, CoE, values and preferences, resources, acceptability, and feasibility, and the overall evaluation then selects a recommendation, which is either support or against and weak or strong. Classifications of the strength and CoE in each recommendation are shown in Table 5. Certainty of evidence for the recommendations is determined as the highest one among those for outcomes when either benefits or harms exist, whereas it is determined as the lowest one when both benefits and harms exist.

**Table 5:** Strength and certainty of evidence in each recommendation

Strength of recommendation	
1	Strong
2	Weak
Certainty of evidence	
A	High
B	Moderate
C	Low
D	Very low

All GRADE recommendations developed by these processes, as well as GPS and answers to BQs, were finalized by consensus building by the GDG. Consensus was confirmed using the modified Delphi method, in which each member of the GDG independently voted on each proposal on a scale of 1 to 9 (1: disagree, 9: agree). Voting was conducted anonymously, and members with a COI related to the relevant CQ and those directly contributing to the development of a recommendation for the relevant CQ abstained from voting. A median score ≥ 7 and a disagreement index < 0.4 were used as consensus criteria. In the case of a disagreement, the EtD table and recommendations were revised by the responsible working group and GDG member. Revisions and votes were repeated until the consensus criteria were met. Minor revisions to CQs were made by consensus of the GDG.

Relevant information for a recommendation based on GRADE in each working group (working group 1–4) was available at Additional file 1–4, respectively.

### Terms in guidelines

*Nutrition therapy*: A component of medical treatment that involves enteral and/or parenteral nutrition including oral nutrition.

*Nutritional disorder*: The state of an altered body composition and/or diminished function due to deficiencies, excesses, or imbalances of energy and/or nutrients.

*Malnutrition*: A condition where pathological symptoms appear due to quantitative or qualitative deficiencies in nutrients necessary for normal metabolism and development.

*Overweight/obesity*: A condition of excessive fat deposits; a body mass index (BMI) between 25 and < 30 kg/m^2^ is classified as overweight, between 30 and < 35 kg/m^2^ as obese, and ≥ 35 kg/m^2^ as severely obese. Morbid obesity is defined as obesity with health issues that are caused by or related to obesity, regardless of BMI.

*Enteral nutrition *: (*EN*). A system of providing nutrition by the intestinal tract, broadly including oral nutrition.

*Enteral tube feeding*: A system of providing nutrition via a tube or stoma into the intestinal tract distal to the oral cavity.

*Parenteral nutrition (PN)*: The intravenous administration of nutrients, which may be provided by a central or peripheral venous line.

*Supplemental parenteral nutrition (SPN)*: PN added to EN when EN alone is insufficient.

*Gastric feeding*: The administration of EN into the stomach via a nasogastric tube or gastrostomy.

*Post-pyloric feeding*: The administration of EN beyond the pylorus via a nasoduodenal or nasojejunal tube or jejunostomy.

*Continuous feeding*: The hourly administration of EN over 24 h assisted by a feeding pump.

*Intermittent feeding*: The administration of EN several times a day (for example, 2–4 times) over a period of time (for example, 1–3 h each).

1. **General principles of nutrition therapy**

**CQ 1-1: Should nutrition therapy using nutrition protocols be provided in critically ill patients?**

**Answer**: We suggest providing nutrition therapy using nutrition protocols to critically ill patients. (GRADE 2B: certainty of evidence = “moderate”).

**Rationale**

Critically ill patients are more likely to have difficulty achieving nutritional targets for energy and protein, and their nutritional supply is typically insufficient. Therefore, when providing nutrition therapy to critically ill patients, it is favorable to have a protocol that outlines criteria for the initiation of EN and PN, the administration methods for EN formulas, the use of specific nutritional products, and the management of enteral feeding intolerance (EFI) and gastrointestinal complications. The clinical benefits of nutrition administration protocols have been demonstrated, particularly those that improve target nutritional dosing [13]. However, it remains unclear whether the implementation of nutritional protocols improves patient outcomes; thus, it is important to clarify the effectiveness of nutrition therapy using these protocols.

A meta-analysis was performed using 2 RCTs and 4 cluster RCTs (Additional file 1) [14–19]. The results of the favorable outcomes were as follows: pneumonia yielded a risk difference (RD) of 16 fewer per 1000 (95% CI 65 fewer to 148 more) (2 RCTs, *n* = 576), dialysis days yielded an RD of 29 fewer per 1000 (95% CI 60 fewer to 2 more) (2 RCTs, *n* = 2970), and enteral nutrition initiation time yielded a mean difference (MD) of 0.4 days shorter (95% CI 0.6 shorter to 0.1 shorter) (6 RCTs, *n* = 3854). A slight effect was observed on mortality and length of ICU stay. Therefore, the favorable outcomes were judged as small. The results of the unfavorable outcomes were as follows: duration of mechanical ventilation yielded a standardized mean difference (SMD) of 0.3 longer (95% CI 0.2 shorter to 0.7 longer) (4 RCTs: *n* = 2173) and vomiting yielded an RD of 9 more per 1000 (95% CI 18 fewer to 65 more) (2 RCTs, *n* = 576). Therefore, the unfavorable outcomes were judged as trivial. Based on the overall balance of effects, we thought that nutrition therapy using nutrition protocols was likely superior.

A nutrition intervention is acceptable from a patient’s perspective because there is no additional cost to the patient for the intervention. It is included in the current requirements for a medical service fee and is considered acceptable under current practice. Additionally, the intervention does not necessitate new medical equipment required for the intervention, and it is acceptable from the perspective of management by the medical institution. Furthermore, the implementation of the protocol does not impose a significant workload on healthcare professionals. Based on the balance of these effects, we concluded that nutrition therapy using nutrition protocols was likely superior.

**CQ 1-2** **:** **Should enteral nutrition be administered rather than parenteral nutrition in critically ill patients?**

**Answer**: We suggest administering enteral nutrition rather than parenteral nutrition in critically ill patients (GRADE 2C: certainty of evidence = “low”).

**Rationale**

EN is generally thought to have the potential to reduce infectious complications in critically ill patients [20]. PN is more likely to cause hyperglycemia and overfeeding, which are associated with infectious complications [21], whereas EN has been shown to improve intestinal epithelial cell function [22, 23]. However, recent large-scale RCTs did not report an improvement in prognosis [24, 25]. EN was found to increase gastrointestinal complications, such as vomiting, diarrhea, and mesenteric ischemia [25, 26]. The choice of a nutrition delivery route is a crucial process in the nutrition therapy, and it is important to establish whether PN or EN is more favorable based on the findings of recent studies. Therefore, this CQ has been identified as an important clinical issue.

A meta-analysis was performed using 36 RCTs [24, 25, 27–60] and secondary analyses of these RCTs (Additional file 1) [61–64]. The results of the favorable outcomes were as follows: length of ICU stay yielded an MD of 0.9 days shorter (95% CI 1.8 shorter to 0.1 shorter) (14 RCTs, *n* = 5431), duration of mechanical ventilation yielded an MD of 0.4 days shorter (95% CI 3.6 shorter to 2.7 longer) (5 RCTs, *n* = 268), sepsis (including bacteremia) yielded an RD of 28 fewer per 1000 (95% CI 37 fewer to 15 more) (15 RCTs, *n* = 5892), and pneumonia yielded an RD of 9 fewer per 1000 (95% CI 32 fewer to 19 more) (18 RCTs, *n* = 5943). Therefore, the favorable outcomes were judged as small. The results of the unfavorable outcomes were as follows: 90-day mortality yielded an RD of 20 more per 1000 (95% CI 8 fewer to 48 more) (3 RCTs, *n* = 4800) and mesenteric ischemia yielded an RD of 7 more per 1000 (95% CI 0 more to 22 more) (3 RCTs, *n* = 4861). Therefore, the unfavorable outcomes were judged as small. Based on the overall balance of effects, we thought that the favorable and unfavorable effects of enteral nutrition were balanced, leading to the conclusion that neither enteral nutrition nor parenteral nutrition was superior to the other.

To evaluate the balance of effects, we conducted three sensitivity analyses and three subgroup analyses. Sensitivity analyses included the following: “excluding studies that administered EN at the standard dose (20 kcal/kg/day) to hemodynamically unstable patients [24, 27–60]”, “including only studies with a low or some concerns regarding the risk of bias [24, 25, 31, 32, 37, 39, 43, 45, 46, 54–56, 59, 63]”, and “including only studies conducted after 2012 [24, 25, 38, 42–44, 49, 52, 55, 60]”. Subgroup analyses included the following: “patients with medical conditions other than acute pancreatitis [24, 25, 35, 41, 45, 46, 50]”, “patients with acute pancreatitis [34, 36, 38, 42, 43, 52, 54, 56]”, and “trauma patients [28, 29, 31–33, 48, 49, 53, 57, 59, 63]”. The findings of sensitivity analyses showed that desirable effects outweighed undesirable effects in “excluding studies that administered EN at the standard dose (20 kcal/kg/day) to hemodynamically unstable patients”, undesirable effects outweighed desirable effects in “including only studies with a low or some concerns regarding the risk of bias”, and desirable and undesirable effects were equivalent in “including only studies conducted after 2012”. Subgroup analyses showed that desirable effects outweighed undesirable effects in “patients with acute pancreatitis” and “trauma patients”, while undesirable effects outweighed desirable effects in “patients with medical conditions other than acute pancreatitis”. The findings of these sensitivity and subgroup analyses suggest that the effects of EN vary depending on the patient population and do not strongly support the use of EN or PN. Therefore, the balance of effects does not appear to favor EN or PN.

Cost-effectiveness was examined in the CALORIES trial [65]. Assuming a value of £20,000 per quality-adjusted life year, the net benefit of PN over one year was − £1320 (95% CI: − £3709 to £1069), indicating that PN is less cost-effective than EN. However, the CALORIES trial was conducted in the UK, where the cost of enteral formulas and parenteral solutions as well as medical fees differ from those in Japan; therefore, it is unclear whether the same conclusions may be drawn. To provide an informative answer, the cost-effectiveness of EN in Japan needs to be examined in future studies. Based solely on the findings of the CALORIES trial, the cost-effectiveness of EN is regarded as “probably good”. In terms of resource requirements, PN necessitates the insertion and management of a central venous catheter, the selection of nutritional formulations, and blood glycemic management, all of which involve significant physical and human resources. In contrast, while EN requires the insertion of a feeding tube, its management is generally easier and the overall resource requirement is considered less than that of PN. Therefore, it was concluded that the resource requirements of EN are “moderately less” than those of PN. In terms of acceptability, EN is mostly provided as meals, incurring a dietary treatment cost of 110 to 490 yen per meal. On the other hand, the daily drug cost for PN is approximately 1,000 to 2,000 yen. Additionally, the risk of complications from the insertion of a feeding tube is low when performed properly. However, the insertion of a central venous catheter, although rare, has been reported to cause serious complications [66]. Furthermore, since enteral formulas contain less water than parenteral solutions, the body’s water balance is more easily maintained with EN. Therefore, EN was deemed to be acceptable. Based on the balance of these effects, we concluded that EN was likely superior to PN.

**CQ 1-3: Should energy provision be intentionally reduced below energy expenditure in the acute phase of critical illness?**

**Answer**: We suggest against intentionally reducing energy intake below energy expenditure in the acute phase of critical illness (GRADE 2B: certainty of evidence = “moderate”).

**Remarks**: This recommendation is derived from studies that generally administered less energy than the target dose in both the intervention and control groups and does not necessarily support full feeding according to energy expenditure from the first day of ICU stay.

**Rationale**

Critically ill patients are often unable to feed themselves and require EN or PN. Overfeeding from an excessive energy prescription or an accumulated energy debt due to prolonged underfeeding may both lead to harmful complications [2]. Due to complex and dynamic changes in metabolism, namely, the catabolic response, the optimal energy provision target to improve clinical outcomes in the acute phase of critical illness remains unclear. Guidelines from various countries differ in their targets for initial energy provision to critically ill patients [5, 67]. There is ongoing controversy regarding optimal energy targets in the acute phase of critical illness due to the adverse effects of both overfeeding and underfeeding. Therefore, an important CQ is whether clinicians need to initially reduce energy provision below energy expenditure (EE).

A meta-analysis was performed using 27 RCTs (Additional file 1) [68–94]. The results of the favorable outcomes were as follows: 90-day mortality yielded an RD of 6 fewer per 1000 (95% CI 20 fewer to 10 more) (7 RCTs, *n* = 10,197), length of ICU stay yielded an MD of 0.04 days shorter (95% CI 1.2 shorter to 1.2 longer) (22 RCTs, *n* = 9339), all infectious complications yielded an RD of 8 fewer per 1000 (95% CI 53 fewer to 45 more) (10 RCTs, *n* = 5491), all adverse events yielded an RD of 3 fewer per 1000 (95% CI 10 fewer to 9 more) (3 RCTs, *n* = 4189), and vomiting yielded an RD of 5 fewer per 1000 (95% CI: 19 fewer to 11 more) (4 RCTs, *n* = 5940). Therefore, the favorable outcomes were judged as trivial. The results of the unfavorable outcomes were as follows: 28-day mortality yielded an RD of 14 more per 1000 (95% CI: 4 fewer to 33 more) (13 RCTs, *n* = 7960), and duration of mechanical ventilation yielded an MD of 0.2 days longer (95% CI 0.7 shorter to 1.0 longer) (13 RCTs, *n* = 6306). Therefore, the unfavorable outcomes were judged as small. Based on the overall balance of effects, we thought that not intentionally reducing energy intake below energy expenditure was likely superior.

Cost-effectiveness has been examined in two RCTs [72, 73]: one showed lower medical costs for PN in the group provided with less energy than in the group provided with energy according to EE (€106 ± 47 vs. €204 ± 119; *P* < 0.0001), while the other reported a reduction in medical costs of €1,100 per patient in the group provided with less energy than EE by discontinuing PN. Therefore, cost-effectiveness was judged to favor intentionally reducing energy intake. Based on these findings, we concluded that not intentionally reducing energy intake below EE was likely superior.

**CQ 1-4: Should higher dose of protein above the standard (> 1.2 g/kg/day) be administered in critically ill patients?**

**Answer**: We suggest administering higher protein doses above the standard (> 1.2 g/kg/day) in critically ill patients (GRADE 2D: certainty of evidence = “very low”).

**Rationale**

In many guidelines, early targeted protein administration is recommended for critically ill patients treated in ICUs [4, 5]. The standard protein requirement for adults is 50 g/day for men with a recommended dose of 60–65 g/day and 40 g/day for women with a recommended dose of 50 g/day [95]. However, observational studies showed that higher doses of protein in critically ill patients may maintain muscle mass, improve functional outcomes, such as reducing the duration of mechanical ventilation, and decrease infectious complications. On the other hand, higher protein doses in the acute phase have been suggested to inhibit autophagy necessary for repairing intracellular damage, delay recovery from organ dysfunction, and worsen clinical outcomes [96]. A recent RCT indicated that higher protein doses may worsen survival outcomes in patients with impaired renal function [97]. Therefore, an important clinical issue is whether to administer protein doses above the standard to critically ill patients. Since protein doses often deviate from target and actual doses due to a number of factors, such as the nutrient products used, this CQ will compare protein doses based on target doses.

A meta-analysis was performed using 10 RCTs (Additional file 1) [80, 97–105]. The results of the favorable outcomes of higher protein doses above standard (> 1.2 g/kg/day) were as follows: length of ICU stay yielded an MD of 0.5 days shorter (95% CI 1.5 shorter to 0.5 longer) (9 RCTs, *n* = 1921), infectious complications yielded an RD of 37 fewer per 1000 (95% CI 181 fewer to 165 more) (3 RCTs, *n* = 249), handgrip strength yielded an MD of 1.8 kg higher (95% CI 1.4 lower to 5.1 higher) (2 RCTs, *n* = 141), muscle mass reduction yielded an SMD of 0.6 lower (95% CI 1.0 lower to 0.3 lower) (3 RCTs, *n* = 191), and diarrhea yielded an RD of 65 fewer per 1000 (95% CI: 151 fewer to 40 more) (4 RCTs, *n* = 310). Therefore, the favorable outcomes were judged as medium. The results of the unfavorable outcomes were as follows: short-term mortality (≤ 60 days) yielded an RD of 17 more per 1000 (95% CI: 23 fewer to 62 more) (8 RCTs, *n* = 1825). A slight effect was observed with duration of mechanical ventilation (8 RCTs, *n* = 1814). Therefore, the unfavorable outcomes were judged as small. Based on the overall balance of effects, we thought that administering higher protein doses above the standard (> 1.2 g/kg/day) was likely superior.

Regarding acceptability, changes in protein dosing may require a modification to the type of elemental formula administered, the use of protein powders, or the use of intravenous amino acid fluid preparations, which may result in a slightly higher patient co-payment. However, the costs related to this are negligible because it only requires a change in nutritional products. Therefore, we consider there to be few reasons for hesitating to change the enteral formula based on economic considerations. Complications associated with these changes are also unlikely and may be acceptable from the patient’s perspective. Based on the balance of these effects, we concluded that administering higher protein doses above the standard (> 1.2 g/kg/day) was likely superior.

**CQ 1-5: Should enteral nutrition be initiated within 48 h following the start of treatment in critically ill patients?**

**Answer**: We recommend initiating enteral nutrition within 48 h following the start of treatment in critically ill patients (GRADE 1B: certainty of evidence = “moderate”).

**Remarks**: The studies included in this meta-analysis only included patients for whom enteral nutrition was feasible.

**Rationale**

Early EN for critically ill patients is recommended in various nutrition guidelines from the perspective of preventing infection. The underlying mechanisms involve the preservation of systemic immunocompetence and the prevention of bacterial translocation by maintaining the structure and function of the intestinal tract, which is also an immune organ. However, definitions of early EN vary from 24 to 72 h, and there is no pathophysiological evidence for any of these definitions. Although previous studies suggested a relationship between early EN and reduced mortality [106], early EN has also been reported to increase gastrointestinal complications and prolong the length of ICU stay [107]. Therefore, the effects of early EN are unclear [108], and it remains an important subject of clinical practice to reassess the benefits and risks of early EN in critically ill patients within 48 h of the start of treatment for critical illness.

A meta-analysis was performed using 16 RCTs (Additional file 1) [107, 109–123]. The results of the favorable outcomes were as follows: length of ICU stay yielded an MD of 2.4 days shorter (95% CI 4.0 shorter to 0.9 shorter) (12 RCTs, *n* = 729), duration of mechanical ventilation yielded an MD of 1.9 days shorter (95% CI 3.8 shorter to 0.04 shorter) (8 RCTs, *n* = 346), infectious complications yielded an RD of 148 fewer per 1000 (95% CI 231 fewer to 19 fewer) (7 RCTs, *n* = 366), and grip strength yielded an MD of 1.1 kg higher (95% CI 0.2 higher to 2.0 higher) (1 RCT: *n* = 100). Therefore, the favorable outcomes were judged as large. On the other hand, none of the effect estimates were in the direction of unfavorable effects. Therefore, the unfavorable outcomes were judged as trivial. Based on the overall balance of effects, we thought that the initiation of enteral nutrition within 48 h was likely superior.

The increased cost of the early initiation of EN is expected to be small. A previous study from the United States showed that the initiation of EN within 24 h of ICU admission reduced total acute care hospital costs by US$14,462 (95% CI US$5464 to US$23,669) per patient [124]. Although this timing differs from our definition of 48 h, the cost-effectiveness of the early initiation of EN is expected to be significant. The initiation of EN within 48 h of the start of intensive care appears feasible in many centers. Based on the balance of these effects, we concluded that the initiation of EN within 48 h of the start of treatment for critical illness was likely superior.

**CQ 1-6: Is enteral nutrition beneficial compared to no enteral nutrition or parenteral nutrition in critically ill patients with hemodynamic instability?**

**Answer**: We suggest against administering enteral nutrition in critically ill patients with hemodynamic instability (GRADE 2D: certainty of evidence = “very low”).

**Rationale**

The early initiation of EN within 48 h of ICU admission is recommended for critically ill patients with functional gastrointestinal tracts [4, 125]. However, safety concerns have been expressed based on previous findings showing increases in severe gastrointestinal complications, such as mesenteric ischemia, in hemodynamically unstable critically ill patients requiring high doses of vasopressors or massive infusions [4, 125]. Therefore, clarifying the balance between the desirable and undesirable effects of EN in hemodynamically unstable critically ill patients is an important clinical issue.

A meta-analysis was performed using 2 RCTs (Additional file 1) [25, 126]. The results of the favorable outcomes were as follows: length of ICU stay yielded an MD of 1.0 days shorter (95% CI 1.7 shorter to 0.3 shorter) (1RCT: *n* = 2410), hospital mortality yielded an RD of 87 fewer per 1000 (95% CI 273 fewer to 396 more) (2RCT: *n* = 2441), duration of mechanical ventilation-free days yielded an MD of 5.3 days longer (95% CI 7.7 shorter to 18.2 longer) (2RCT: *n* = 2441), ventilator-associated pneumonia yielded an RD of 4 fewer per 1000 (95% CI 24 fewer to 21 more) (2RCT: *n* = 2441), and vomiting yielded an RD of 31 fewer per 1000 (95% CI 169 fewer to 581 more) (2RCT: *n* = 2441). Therefore, the favorable outcomes were judged as small. The results of the unfavorable outcomes were as follows: mesenteric ischemia yielded an RD of 12 more per 1000 (95% CI 2 more to 38 more) (2RCT: *n* = 2441). Therefore, the unfavorable outcomes were judged as small. Based on the overall balance of effects, we thought that neither enteral nutrition nor the absence of nutrition was superior to the other.

EN is not recommended for hemodynamically unstable patients. However, in clinical practice, small amounts of EN are sometimes administered to these patients depending on their condition [127]. This practice may be attributed to differences in healthcare professionals’ values and the patient’s specific pathophysiology. Therefore, the acceptability of administering EN to hemodynamically unstable patients was assessed as “variable”. Since we considered neither EN nor the absence of EN to be superior to the other in critically ill patients with hemodynamic instability, we suggest against administering EN to these patients.

**CQ 1-7: Should supplemental parenteral nutrition be initiated in critically ill patients receiving insufficient amounts of enteral nutrition?**

**Answer**: We suggest against initiating supplemental parenteral nutrition in critically ill patients receiving insufficient amounts of enteral nutrition (GRADE 2A: certainty of evidence = “high”).

**Remarks**: The intervention period of the included studies was from the start of intensive care until days 7–9. Therefore, this recommendation does not apply to supplemental parenteral nutrition after that time window.

**Rationale**

EN is physiological when the digestive tract is usable; however, nutritional intake is often inadequate when relying on EN alone [128, 129]. Although the prognosis of a patient may improve with the provision of sufficient nutrition by SPN, it may also lead to overfeeding, potentially increasing the risk of infectious complications, significant hyperglycemia, and other adverse complications [73, 130]. Therefore, it is important to clarify the benefits and risks of SPN in critically ill patients who are receiving insufficient amounts of EN.

A meta-analysis was performed using 11 RCTs (Additional file 1) [68, 72, 73, 81, 90, 94, 130–134]. The results of the favorable outcomes were as follows: short-term mortality yielded an RD of 6 fewer per 1000 (95% CI 19 fewer to 7 more) (6 RCTs, *n* = 6731), long-term mortality yielded an RD of 1 fewer per 1000 (95% CI 18 fewer to 16 more) (5 RCTs, *n* = 6333), duration of mechanical ventilation yielded an MD of 0.1 days shorter (95% CI 0.9 shorter to 0.7 longer) (8RCTs, *n* = 6874), SF-36 physical functioning domain yielded an MD of 2.5 higher (95% CI 6.1 lower to 11.1 higher) (3RCTs, *n* = 1157), and adverse events yielded an RD of 6 fewer per 1000 (95% CI 26 fewer to 18 more) (2RCTs, *n* = 4760). Therefore, the favorable outcomes were judged as trivial. The results of the unfavorable outcomes were as follows: length of ICU stay yielded an MD of 0.5 days longer (95% CI 0.7 shorter to 1.7 longer) (9RCTs, *n* = 6873), infectious complications (bloodstream infections) yielded an RD of 15 more per 1000 (95% CI 3 more to 29 more) (6RCTs, *n* = 6704). Therefore, the unfavorable outcomes were judged as small. In addition, infectious complications (all causes) also yielded an RD of 15 more per 1000 (95% CI 11 fewer to 44 more) (6RCTs, *n* = 6655), the unfavorable outcomes were also judged as small. Based on the overall balance of effects, we thought that enteral nutrition alone was likely superior to SPN in critically ill patients receiving insufficient amounts of enteral nutrition.

PN is a common procedure that may be performed in any hospital. The daily cost of parenteral solutions is 1000 to 2000 yen which has a minimal impact on the overall intensive care setting. Rare, but serious complications have been reported with the insertion of a central venous catheter [135], and these risks must be considered when inserting a catheter solely for PN. In consideration of the associated costs and risks, tolerability for patients and their families is ‘likely acceptable’. Although tolerability is acceptable, EN alone was shown to be superior to SPN in terms of the balance of effects, leading to this recommendation.

**CQ 1-8: Should post-pyloric feeding be used rather than gastric feeding for enteral nutrition in critically ill patients?**

**Answer**: We suggest using post-pyloric feeding rather than gastric feeding for enteral nutrition in critically ill patients (GRADE 2D: certainty of evidence = “very low”).

**Rationale**

EN via the gastrointestinal tract is preferable for critically ill patients when gastrointestinal function is preserved. However, gastroparesis frequently occurs in critically ill patients [136], potentially leading to aspiration due to delayed gastric emptying and vomiting [137]. While prokinetic agents are suggested for gastroparesis [67], previous studies reported that these medications did not improve the outcomes of critically ill patients [137]. Therefore, post-pyloric EN, which bypasses the stomach with impaired motility, has emerged as an alternative method for gastrointestinal feeding in critically ill patients. Post-pyloric feeding is expected to ensure reliable nutrient delivery to the intestines beyond the duodenum while reducing the risk of aspiration and vomiting [138, 139]. However, this approach may delay the initiation of EN [140] or nutritional support itself, depending on the insertion technique and proficiency of practitioners. Furthermore, post-pyloric feeding is associated with some complications, such as gastrointestinal bleeding [138] and perforation. Therefore, it is crucial to clarify the benefits and risks associated with post-pyloric EN.

A meta-analysis was performed using 17 RCTs (Additional file 1) [138–154]. The results of the favorable outcomes were as follows: all-cause mortality yielded an RD of 6 fewer per 1000 (95% CI 50 fewer to 47 more) (13 RCTs, *n* = 1154), the length of ICU stay yielded an MD of 1.4 days shorter (95% CI 2.9 shorter to 0.2 longer) (11 RCTs, *n* = 941), duration of mechanical ventilation yielded an MD of 2.2 days shorter (95% CI 3.4 shorter to 1.0 shorter) (7 RCTs, *n* = 622), pneumonia (ventilator-associated pneumonia or aspiration pneumonia) yielded an RD of 113 fewer per 1000 (95% CI 158 fewer to 51 fewer) (13 RCTs, *n* = 1079), and vomiting yielded an RD of 90 fewer per 1000 (95% CI 153 fewer to 39 more) (8 RCTs, *n* = 689). Therefore, the favorable outcomes were judged as moderate. The results of the unfavorable outcomes were as follows: the time from ICU admission to initiation of enteral nutrition yielded an MD of 9.6 h longer (95% CI 0.5 shorter to 19.7 longer) (4 RCTs, *n* = 316) and diarrhea yielded an RD of 8 more per 1000 (95% CI 38 fewer to 72 more) (8 RCTs, *n* = 743). Therefore, the unfavorable outcomes were judged as small. Based on the overall balance of effects, we thought that post-pyloric feeding was likely superior to gastric feeding for enteral nutrition in critically ill patients.

Tubes used for post-pyloric feeding are generally more expensive than those used for gastric feeding. Reliable post-pyloric tube insertion requires endoscopic or fluoroscopic guidance. Blind insertion of a post-pyloric tube is not impossible, but the possibility depends on the operator's level of expertise. Devices to assist insertion have already been approved by Japan's public health insurance system, but they are not commonly used in Japan. In some cases, insertion may be deemed impossible by any method. Therefore, insertion of a post-pyloric tube is not always possible at all facilities, and only a few facilities are expected always to have it available.

Facilities and personnel limitations also present significant challenges to inserting and managing post-pyloric tubes. Even in institutions that meet the necessary conditions, it cannot be stated that post-pyloric feeding is consistently feasible for all patients at all times; hence, feasibility is described as "variable". Since the implementation of post-pyloric feeding for all ICU patients is expected to be challenging, it may only be provided to patients with a high risk of EFI. Furthermore, post-pyloric feeding is generally conducted with continuous or cautious infusions.

Based on the balance of these effects, we concluded that post-pyloric feeding was likely superior to gastric feeding for EN in critically ill patients.

**CQ 1-9: Should continuous feeding rather than intermittent feeding be used for gastric enteral nutrition in critically ill patients?**

**Answer**: We suggest continuous feeding rather than intermittent feeding for gastric enteral nutrition in critically ill patients (GRADE 2D: certainty of evidence = “very low”).

**Rationale**

When EN is administered to critically ill patients, it may be delivered via a gastric tube either continuously or intermittently. Continuous feeding provides nutrition throughout the day, while intermittent feeding divides the administration of nutrition into two to six feedings per day. Although continuous feeding is thought to have a lower infusion rate and fewer gastrointestinal complications [155], interruptions due to examinations or rehabilitation often result in a lower total volume of nutrition than the target volume. Intermittent feeding has been suggested to promote more natural hormonal and metabolic responses, such as improved protein synthesis and secretion of digestive hormones [156, 157]. However, gastrointestinal complications were previously reported to be more common in critically ill patients [158]. Intermittent feeding may also eliminate the need for EN management at night, but has the disadvantage of increasing nursing workload related to the preparation required for each administration. Therefore, clarifying the balance between the benefits and risks of continuous and intermittent feeding is of great clinical significance and an important clinical issue.

A meta-analysis was performed using 10 RCTs (Additional file 1) [155, 159–167]. The results of the favorable outcomes were as follows: mortality yielded an RD of 55 fewer per 1000 (95% CI 110 fewer to 22 more) (5 RCTs, *n* = 458), length of ICU stay yielded an MD of 0.8 days shorter (95% CI 4.2 shorter to 2.6 longer) (3 RCTs, *n* = 309), duration of mechanical ventilation yielded an MD of 2.3 days shorter (95% CI 5.4 shorter to 0.9 longer) (2 RCTs, *n* = 161), and diarrhea yielded an RD of 40 fewer per 1000 (95% CI 94 fewer to 40 more) (6 RCTs, *n* = 385). Therefore, the favorable outcomes were judged as small. The results of the unfavorable outcomes were as follows: infectious complications yielded an RD of 235 more per 1000 (95% CI 204 fewer to 1000 more) (2RCTs, *n* = 267), rate of muscle mass loss yielded an MD of 1.9% higher (95% CI 13.3 lower to 17.1 higher) (1RCT, *n* = 121), and vomiting yielded an RD of 43 more per 1000 (95% CI 62 fewer to 391 more) (3RCTs, *n* = 260). Therefore, the unfavorable outcomes were judged as small. Although both the favorable and unfavorable effects were small, the favorable effects included more critical outcomes such as mortality, length of ICU stay, and duration of mechanical ventilation. Based on the overall balance of effects, we thought that continuous feeding was likely superior.

There are a few concerns regarding the acceptability of continuous gastric feeding for patients and their families compared to the invasive procedure. Intermittent feeding requires more frequent tube equipment changes than continuous feeding using ready-to-hang formulations. Therefore, continuous feeding is thought to require fewer resources and results in cost saving. However, it is difficult to specify the exact amount of estimated cost saving. This is because equipment procurement costs vary by hospital, and the administration methods and frequency of intermittent administration differ by case. Based on the balance of these effects, we concluded that continuous feeding was likely superior.

**CQ 1-10: What is parenteral nutrition strategy in critically ill patients for whom enteral nutrition is not feasible?**

**Answer:** In critically ill patients for whom enteral nutrition is not feasible for a while, the absence of parenteral nutrition, the only means of nutrition therapy, may worsen the prognosis (provision of information for background question).

**Rationale**

Few studies have compared the presence or absence of early PN administration in critically ill patients for whom EN is not feasible. One RCT examined the timing of PN initiation in cases in which EN was relatively contraindicated [130]. Another RCT investigated the timing of PN initiation in patients with a significantly low EN intake [168]. However, the severity of patients in this study was low, making it challenging to create recommendations based on these findings. However, the aforementioned RCTs suggested that discontinuing nutrition therapy in critically ill patients unable to receive EN is likely to worsen their prognosis, which is also physiologically plausible.

Previous nutrition therapy guidelines for critically ill patients recommend the initiation of nutrition therapy if adequate oral intake is not possible within 2 to 3 days. To mitigate the risk of infectious complications and reduce healthcare costs, the initiation of EN is recommended within 48 h of ICU admission. However, the timing of PN initiation in cases in which EN is not feasible has yet to be examined in detail. The benefits of early EN are expected not only from the provision of nutrition, but also from the maintenance of immune function, thereby reducing the incidence of infections. In contrast, PN administration in patients with hemodynamic instability or severe inflammation may reduce intestinal blood flow [169], and even if the same amount of energy is administered, the amount of energy absorbed into the body may be higher with PN [170], leading to the potential for overfeeding. Furthermore, meta-analyses have shown that PN has a higher incidence of infectious complications than EN, and ASPEN guideline 2016 did not recommend early PN initiation within the first week of ICU admission for patients with low nutritional risk [125]. In Japan, based on the above physiological perspectives and the recommendations of these clinical guidelines, there are cases in which nutrition therapy is not administered to critically ill patients who cannot start EN during the acute phase. However, recent RCTs on the early initiation of EN or PN reported no significant differences in mortality or infectious complications [24, 25]. ASPEN guideline 2022 states that there are no differences between EN and PN [5]. Additionally, ESPEN guideline 2023 permits the early initiation of PN for malnourished patients where EN is contraindicated. If a patient is not malnourished, but EN is contraindicated, PN initiation is recommended between 3 and 7 days after ICU admission [4]. Therefore, although clear evidence is limited, the recommendations of recent guidelines related to this CQ have been evolving. The provision of further information is needed on whether to avoid PN and withhold nutrition for patients where EN is not feasible for a while.

A systematic review investigating the impact of early PN included only one RCT that compared a group started on early PN with a comparison group (fluids and electrolytes, but no nutrition therapy) [171]. Furthermore, only one RCT investigated whether SPN needed to be added when only small amounts of EN were given [168].

The findings of two RCTs are discussed below. An RCT on PN in the ICU (The Early PN trial) indicated that in cases in which EN was relatively contraindicated, 60-day mortality and infection rates did not significantly differ between the group that started PN early (44 min after allocation) and the control group (started EN or PN an average of 2.8 days after admission) [130]. There was no significant reduction in the ICU or hospital length of stay; however, the duration of mechanical ventilation was reduced by 0.47 days (95% CI − 0.82 to − 0.11; *P* = 0.01), and the duration of coagulation disorders was also decreased. Moreover, the amount of muscle and fat loss was significantly reduced in the early PN group, and the decrease in mid-upper arm circumference was also restrained in the early PN group, with no increase in adverse events due to early PN [130]. Additionally, an RCT compared early versus late PN initiation in patients who underwent scheduled major abdominal surgery and had a low EN intake (< 30% of the target dose of 25 kcal/kg/day for women and 30 kcal/kg/day for men) [168]. In the group in which PN was started on postoperative day 3, the incidence of nosocomial infections from postoperative day 3 to discharge was lower than in the group in which PN was started late (postoperative day eight or later) (10/115 [8.7%] vs. 21/114 [18.4%]; risk difference 9.7%, 95% CI 0.9 to 18.5%; *P* = 0.04) [168]. Moreover, two RCTs comparing EN and PN, the CALORIES trial and NUTRIREA-2 trial [24, 25], showed that if intake amounts were almost equal, mortality and infectious complications did not significantly differ between EN and PN initiated within 24 or 36 h of ICU admission, suggesting that not providing nutrition therapy worsens patient outcomes and also that providing nutrition therapy via PN prevents worsening outcomes in cases in which EN is difficult. However, the question of when to initiate PN when EN is not feasible could not be conclusively answered by the aforementioned RCTs with differing intervention timings. Furthermore, to imply a situation where EN cannot be provided for a certain period, this CQ uses the phrase “for a while” and specifies, “In critically ill patients for whom enteral nutrition is not feasible for a while”.

In summary, in critically ill patients for whom EN is not feasible for a while, the absence of PN, the only means of nutrition therapy, was likely to worsen prognosis. However, further clinical trials are necessary.

2. **Specific nutrients in nutrition therapy**

**CQ2-1: Should omega-3 fatty acid-enriched enteral nutrition be administered to critically ill patients?**

**Answer**: We suggest administering omega-3 fatty acid-enriched enteral nutrition to critically ill patients (GRADE 2C: certainty of evidence = “low”).

**Remarks**: The dosage of omega-3 fatty acid have not been examined.

**Rationale**

Omega-3 fatty acids, represented by eicosapentaenoic acid and docosahexaenoic acid, exert anti-inflammatory effects by suppressing the production of inflammatory eicosanoids (such as prostaglandins E_2_, and leukotrienes B_4_), unlike omega-6 fatty acids, including arachidonic acid, which are involved in the production of inflammatory cytokines [172]. Additionally, omega-3 fatty acids produce mediators, such as resolvins, protectins, and maresins, which promote the resolution of inflammation. These mediators were recently shown to suppress neutrophil infiltration at inflammation sites and enhance the phagocytosis of damaged cells by macrophages [173]. Therefore, the anti-inflammatory properties of omega-3 fatty acids are expected to improve outcomes in critically ill patients, such as those with sepsis or acute respiratory distress syndrome (ARDS), where cytokines and inflammatory mediators contribute to worsening of the disease [172]. However, the effects of EN enriched with omega-3 fatty acids has yet to be consistently demonstrated, making it a significant issue addressed by JCCNG 2024. Various EN formulas enriched with different amounts of omega-3 fatty acids are available in clinical practice worldwide, along with options to supplement foods and medications. On the other hand, EN formulas with high doses of omega-3 fatty acids have been discontinued in Japan. Therefore, we included all EN formulas enriched with omega-3 fatty acids, regardless of the dosage, as an intervention. In addition, we conducted a sensitivity analysis focusing on studies that intended to administer omega-3 fatty acid-enriched EN, as well as those involving bolus administration based on previous findings showing a relationship with adverse events [4].

A meta-analysis was performed using 41 RCTs (*n* = 5251) (Additional file 2) [80, 86, 87, 102, 103, 174–209]. The results of favorable outcomes were as follows: length of ICU stay yielded a MD of 2.0 days shorter (95% CI 3.2 days shorter to 0.8 days shorter) (30 RCTs, *n* = 3875), incidence of infectious complications yielded an RD of 42 fewer per 1000 patients (95% CI 111 fewer to 42 more) (15 RCTs, *n* = 2224), and duration of mechanical ventilation yielded a MD of 1.8 days shorter (95% CI 3.1 days shorter to 0.4 days shorter) (26 RCTs, *n* = 3198). Minor benefits were observed for short-term mortality, physical function QoL, and reduction in muscle mass. Therefore, the favorable outcomes were judged as small. No unfavorable outcomes, including adverse events, were observed. Based on the overall balance of effects, we thought that using the omega-3 fatty acid-enriched enteral nutrition was likely superior.

A sensitivity analysis on studies that intended to administer omega-3 fatty acid-enriched EN indicated that favorable outcomes were small and unfavorable outcomes were negligible. Based on the balance of these effects, we concluded that the use of omega-3 fatty acid-enriched EN was likely superior (Additional file 2).

Omega-3 fatty acid-enriched EN is acceptable because there are no moral or ethical issues due to differences in nutritional and drug contents. The intervention is sufficiently feasible due to the availability of commercially available formulas, including those with even small amounts of omega-3 fatty acids, and the only slightly increased cost, and there are no unfavorable outcomes. However, the bolus administration of omega-3 fatty acid-enriched EN requires caution due to the findings of the sensitivity analysis, which revealed undesirable effects in the duration of ventilator-free days (MD of 1.5 days shorter (95% CI 5.5 shorter to 2.4 longer) (2 RCTs, *n* = 361) and the incidence of adverse events yielding an RD of 402 per 1000 patients (95% CI 347 fewer to 1000 more) (2 RCTs, n = 463) (Additional file 2).

**CQ2-2: Should glutamine-enhanced enteral nutrition be administered in critically ill patients?**

**Answer**: We suggest against administering glutamine-enhanced enteral nutrition in critically ill patients receiving enteral nutrition (GRADE 2D: certainty of evidence = “very low”).

**Remarks**: The dosage of glutamine has not been examined.

**Rationale**

Glutamine is an essential nutrient that contributes to the proliferation of immune cells, cytokine production, macrophage phagocytosis, and neutrophil bactericidal activity, earning it the nickname “fuel for the immune system” [210]. Observational studies reported that blood glutamine levels rapidly decrease due to stress, and low glutamine levels are associated with increased mortality and complications [211]. Therefore, while it is a non-essential amino acid in healthy individuals, it is regarded as a conditionally essential amino acid under stress conditions. Numerous studies have been conducted on the efficacy of glutamine administration in critically ill patients. However, the intravenous administration of glutamine has been associated with increased mortality, particularly in critically ill patients with multiple organ failure [212]. On the other hand, the clinical benefits of enteral glutamine administration have been demonstrated in severely burned patients [213], and the findings of a large-scale study on the effects of enteral glutamine administration in severely burned patients were reported in 2022 [214]. Therefore, confirmation of its efficacy is of significant clinical importance, making it a critical clinical issue that needs to be addressed in JCCNG 2024.

A meta-analysis was performed using 23 RCTs (*n* = 3402) (Additional file 2) [185, 188, 195, 196, 214–232]. The results of the favorable outcomes were as follows: incidence of infectious complications yielded an RD of 23 fewer per 1000 patients (95% CI 103 fewer to 75 more) (8 RCTs, *n* = 1197) and incidence of all adverse events yielded an RD of 20 fewer per 1000 patients (95% CI 90 fewer to 78 more) (3 RCTs, *n* = 1825), Therefore, the favorable outcomes were judged as small. The results of the unfavorable outcomes were as follows: (incidence of all-cause mortality yielded an RD of 3 more per 1000 patients (95% CI 29 fewer to 36 more) (18 RCTs, *n* = 3195), length of ICU stay yielded a MD of 1.8 days longer (95% CI 0.07 shorter to 3.7 longer) (18 RCTs, *n* = 3195), duration of mechanical ventilation yielded a MD of 0.48 days longer (95% CI 0.51 shorter to 1.47 longer) (11 RCTs, *n* = 1990), and diarrhea yielded an RD of 56 more per 1000 patients (95% CI 10 more to 118 more) (6 RCTs, *n* = 799). Therefore, the unfavorable outcomes were also judged as small. Based on the overall balance of effects, we thought that neither using glutamine-enhanced enteral nutrition nor not using glutamine-enhanced enteral nutrition was superior to the other. Generally, mortality, ventilation duration, and ICU stay have higher relative importance than infections and adverse events. Based on the overall balance of effects, we made a weak recommendation against the intervention.

The use of glutamine-enhanced EN is ethically and morally acceptable, and many formulations are available, making the cost increase minimal and the intervention feasible. However, the increased risk of diarrhea associated with its use needs to be considered when evaluating its acceptability.

**CQ2-3: Should enteral nutrition with oligomeric or elemental formulas rather than food-based blenderized formulas or polymeric formulas be used in critically ill patients?**

**Answer**: We suggest against administering enteral nutrition with oligomeric or elemental formulas intended as a source of nitrogen in critically ill patients (GRADE 2D, certainty of evidence = “very low”).

**Remark**: The analysis could not be performed only for cases with malabsorption syndromes such as short bowel syndrome and pancreatic exocrine insufficiency, or enteral feeding intolerance. In addition, the recommendations do not indicate a benefit of food-based blenderized formulas or polymeric formulas.

**Rationale**

Critically ill patients may have impaired pancreatic exocrine function and intestinal mucosal damage [233]. Therefore, in comparison with food-based blenderized formulas or polymeric formulas in which the nitrogen source is protein, oligomeric or elemental formulas in which the nitrogen source consists of amino acids, low-molecular-weight peptides, or partially hydrolyzed products theoretically are more advantageous for digestion and absorption [234]. Therefore, oligomeric or elemental formulas may improve outcomes by reducing gastrointestinal intolerance and contributing to increased energy and protein provision and absorption in critically ill patients; however, their efficacy remains unclear [235]. Oligomeric or elemental formulas may also reduce diarrhea in critically ill patients. Diarrhea causes skin issues in critically ill patients, which not only impairs quality of life (QoL), but also places a burden on medical staff. Therefore, we consider this to be an important clinical issue that needs to be addressed in JCCNG 2024 because of the clinical significance of clarifying the efficacy of oligomeric and elemental formulas.

A meta-analysis was performed using 20 RCTs (Additional file 2) [18, 102, 103, 175, 188, 208, 224, 228, 236–247]. The results of the favorable outcomes were as follows: length of ICU stay yielded a MD of 1.2 days shorter (95% CI 2.4 days shorter to 0.08 days shorter) (9 RCTs, *n* = 669), duration of mechanical ventilation yielded a MD of 0.6 days shorter (95% CI 1.4 days shorter to 0.2 days longer) (5 RCTs, *n* = 486), diarrhea yielded an RD of 6 fewer per 1000 patients (95% CI 92 fewer to 108 more) (12 RCTs, *n* = 909). Therefore, the favorable outcomes were judged as small. The results of the unfavorable outcomes were as follows: incidence of mortality yielded an RD of 0 more per 1000 (95% CI 40 fewer to 53 more) (12 RCTs, *n* = 1021), infectious complication yielded an RD of 27 more per 1000 (95% CI 54 fewer to 126 more) (10 RCTs, *n* = 966). Therefore, the unfavorable outcomes were also judged as small. Based on the overall balance of effects, we thought that neither using enteral nutrition with oligomeric or elemental formulas nor not using enteral nutrition with oligomeric or elemental formulas was superior to the other.

The use of EN with oligomeric or elemental formulas is considered ethically and morally acceptable. Additionally, many formulations, including an elemental diet and low-molecule peptide formula, are available, and the cost increase is minimal, making this intervention highly feasible. However, the balance of effects does not favor the intervention.

**CQ2-4: Should arginine-enhanced enteral nutrition be administered in critically ill patients?**

**Answer**: We suggest against administering arginine-enhanced enteral nutrition in critically ill patients receiving enteral nutrition (GRADE 2D: certainty of evidence = “very low”).

**Remarks**: The dosage of arginine have not been examined.

**Rationale**

Arginine is a substrate for nitric oxide (NO), which plays a crucial role in regulating the microcirculation, and has been shown to enhance immune function, promote protein synthesis, and facilitate wound healing [248, 249]. The administration of arginine-enhanced nutrition during the perioperative period in gastrointestinal surgery has been suggested to reduce infectious complications and improve the prognosis of patients [250, 251]. On the other hand, the administration of arginine to critically ill patients, such as those with sepsis, raises concerns about the production of large amounts of NO, leading to excessive peripheral vasodilation and adverse effects on hemodynamics [252]. Therefore, various guidelines provide different recommendations depending on the disease [251, 252]. The efficacy of arginine-enhanced nutrition in critically ill patients, including those in the perioperative period, remains unclear and warrants reevaluation. This underscores the high importance of this CQ.

A meta-analysis was performed using 23 RCTs (*n* = 2311) (Additional file 2) [174–176, 178, 180, 182–185, 188, 215, 217, 218, 220, 223, 225, 232, 253–258]. The results of the favorable outcomes were as follows: incidence of infectious complications yielded an RD of 8 fewer per 1000 patients (95% CI 141 fewer to 206 more) (4 RCTs, *n* = 840), duration of mechanical ventilation yielded a MD of 0.23 days shorter (95% CI 1.6 shorter to 1.1 longer) (2 RCTs, *n* = 2970), rate of muscle mass loss yielded a MD of 3% lower (95% CI 7.3 lower to 166 more) (1 RCT, *n* = 50), and incidence of adverse events yielded an RD of 36 fewer per 1000 patients (95% CI 156 lower to 166 more) (1 RCT, *n* = 50). Therefore, the favorable outcomes were judged as small. The results of the unfavorable outcomes were as follows: incidence of all-cause mortality yielded an RD of 20 more per 1000 patients (95% CI 17 fewer to 61 more) (15 RCTs, *n* = 1965) and length of ICU stay yielded a MD of 0.5 days longer (95% CI 1.7 shorter to 2.7 longer). Therefore, the unfavorable outcomes were also judged as small. Based on the overall balance of effects, we thought both the favorable and unfavorable outcomes were small and concluded that neither the intervention nor the comparator is supported. Generally, the relative importance of mortality is high, and based on the balance of these effects, we do not recommend this intervention.

The use of arginine-enhanced EN is considered ethically and morally acceptable. Additionally, many formulations are available, and the cost increase is minimal, making this intervention highly feasible. However, the potential increase in mortality associated with its use cannot be ruled out and needs to be considered when evaluating its acceptability.

**CQ2-5: Should high-fat, low-carbohydrate enteral nutrition be used for critically ill patients?**

**Answer**: We suggest against administering high-fat, low-carbohydrate enteral nutrition for critically ill patients (GRADE 2C: certainty of evidence = “low”).

**Rationale**

In critically ill patients, high-fat, low-carbohydrate EN is expected to reduce CO_2_ production, and previous studies mainly examined patients with respiratory failure, such as those on mechanical ventilation [259]. The respiratory quotient, which is the volume ratio of CO_2_ produced to oxygen consumed, is smaller for fat at 0.71 than 1.00 for carbohydrates because fat has the lowest respiratory quotient among the three major nutrients. Additionally, a high-fat, low-carbohydrate content is expected to suppress rapid fluctuations in blood glucose levels, promote glucose stability, and facilitate easier blood glucose management, which may contribute to improvements in mortality [260]. Recent RCTs examined the effects of high-fat, low-carbohydrate EN in critically ill patients, with expectations of fewer complications and improvements in mortality [200, 260–269]. However, since these studies were small and often methodologically limited, the effects and risks of high-fat, low-carbohydrate EN remain unclear, making this an important issue addressed in JCCNG 2024.

A meta-analysis was performed using 11 RCTs (Additional file 2) [200, 260–269]. The results of the favorable outcomes were as follows: length of ICU stay yielded a MD of 0.2 days shorter (95% CI 1.9 days shorter to 1.6 longer) (3 RCTs, *n* = 299), duration of mechanical ventilation yielded a MD of 1.7 days shorter (95% CI 2.9 days shorter to 0.5 days shorter) (4 RCTs, *n* = 318), diarrhea yielded an RD of 78 fewer per 1,000 patients (95% CI 170 fewer to 39 more) (3 RCTs, *n* = 149), and gastric residual volume (> 250–500 mL) yielded an RD of 12 fewer per 1,000 patients (95% CI:131 fewer to 155 more) (3 RCTs, *n* = 149). Therefore, the favorable outcomes were judged as small. The results of the unfavorable outcomes were as follows: incidence of 30-day mortality yielded an RD of 26 more per 1000 patients (95% CI 38 fewer to 117 more) (6 RCTs, *n* = 487). Therefore, the unfavorable effects were also judged to be small. Based on the overall balance of effects, we thought that neither using high-fat, low-carbohydrate enteral nutrition nor the comparator was superior to the other.

The use of high-fat, low-carbohydrate enteral nutrition is considered acceptable ethically and morally. Additionally, many formulations are available, and the cost increase is minimal, making this intervention highly feasible. However, the potential increase in mortality associated with its use cannot be denied and should be considered when evaluating its acceptability.

**CQ2-6: Should intravenous lipid emulsions be administered to critically ill patients receiving parenteral nutrition?**

**Answer**: We suggest against administering intravenous lipid emulsions to critically ill patients receiving parenteral nutrition (GRADE 2D, certainty of evidence = “very low”).

**Remark**: In this meta-analysis, intravenous lipid emulsion was administered within 2 weeks of ICU admission.

**Rationale**

When administering PN to critically ill patients, lipid emulsions may be considered as an efficient source of energy (9 kcal/g) compared to proteins and carbohydrates (4 kcal/g), or prevention of essential fatty acid deficiency [20]. Lipid emulsions may be broadly classified into omega-3 fatty acids derived from fish oil and omega-6 fatty acids derived from soybean oil. In Japan, only omega-6 lipid emulsions are available for use. Omega-6 fatty acids have been shown to aggravate inflammation through the production of inflammatory substances, such as prostaglandins E_2_, thromboxanes A_2_, and leukotrienes B_4_, by the arachidonic acid pathway [270]. Therefore, caution is required when administering omega-6 lipid emulsions to patients with conditions involving cytokines and inflammatory mediators, such as sepsis or ARDS, because they may further exacerbate inflammation. On the other hand, omega-3 lipid emulsions were previously shown to be beneficial due to their antagonistic effects on inflammatory substances and their anti-inflammatory effects mediated by metabolic products such as prostaglandins I_3_, prostaglandins E_3_, thromboxanes A_23_, and leukotrienes B_5_ [271]. However, despite these mechanisms, the efficacy of and risks associated with the intravenous administration of lipid emulsions remain unclear, making its evaluation an important issue in clinical practice.

A meta-analysis was performed using 8 RCTs (Additional file 2) (*n* = 372) [83, 272–278]. The results of the favorable outcomes were as follows: incidence of hospital mortality yielded an RD of 22 fewer per 1000 patients (95% CI 110 fewer to 127 more) (7 RCTs, *n* = 345), length of ICU stay yielded a MD of 0.4 days shorter (95% CI 4.8 days shorter to 4.0 days longer) (6 RCTs, *n* = 324), infectious complication yielded an RD of 33 fewer per 1000 patients (95% CI 191 fewer to 310 more) (3 RCTs, *n* = 180), and adverse outcome yielded an RD of 166 fewer per 1000 patients (95% CI 196 fewer to 60 more) (1 RCTs, *n* = 60). Therefore, the favorable outcomes were judged as moderate. The results of the unfavorable outcomes were as follows: duration of mechanical ventilation yielded a MD 5.8 days longer (95% CI 4.6 days shorter to 16.1 days longer) (6 RCTs, *n* = 324). Therefore, the unfavorable outcomes were also judged as moderate. Consequently, we did not support either the intervention or the comparator based on an overall assessment of the balance of effects. Additionally, we conducted a sensitivity analysis regarding omega-3 lipid emulsions and non-omega-3 lipid emulsions containing omega-6 or medium-chain fatty acids. For omega-3 lipid emulsions (Additional file: CQ2–6), 4 RCTs (*n* = 227) showed a decrease of 78 per 1000 patients in-hospital mortality (95% CI 196 fewer to 175 more) and the length of ICU stay was MD 2.4 days shorter (95% CI 6.2 days shorter to 1.4 days longer), which were both positive directions. In contrast, for non-omega-3 lipid emulsions, 3 RCTs (*n* = 118) showed an increase of 41 per 1000 patients in-hospital mortality (95% CI 51 fewer to 244 more), and 2 RCTs (*n* = 97) showed a prolonged MD 4.5 days in length of ICU stay (95% CI 8.3 days shorter to 17.2 days longer), which were both negative directions.

The intravenous administration of lipid emulsions, being an adjunct to PN, does not raise ethical issues and is acceptable. Since lipid emulsions are widely available, the feasibility of this intervention is sufficient. However, the balance of effects does not favor the intervention. In Japanese prescribing information, lipid emulsions are contraindicated in cases of thrombosis, severe liver dysfunction, severe coagulation disorders, hyperlipidemia, and ketosis, which may occur in critically ill patients; therefore, we do not recommend the administration of lipid emulsions.

**CQ2-7-1: Should prebiotics be administered in critically ill patients?**

**Answer**: We recommend administering prebiotics to critically ill patients (GRADE 1B: certainty of evidence = “moderate”).

**Rationale**

Prebiotics are non-digestible food components that exert beneficial effects on the host through their selective stimulation of the growth or activity of a single or limited number of bacteria in the colon, such as non-digestible oligosaccharides (fructooligosaccharides, xylooligosaccharides, galactooligosaccharides, and lactosucrose) and soluble dietary fiber (non-digestible dextrin, polydextrose, and inulin). Prebiotics are an important energy source for many intestinal bacteria, and one of their metabolites, short-chain fatty acids, exerts various effects, such as serving as an energy source for intestinal epithelial cells, promoting intestinal peristalsis, and regulating systemic immunity by intestinal immunity [279]. However, there is no consensus on whether the administration of prebiotics is effective for critically ill patients. Therefore, we consider this to be an important clinical issue that needs to be addressed in JCCNG 2024.

A meta-analysis was performed using 17 RCTs (*n* = 1041) (Additional file 2) [224, 280–295].The results of the favorable outcomes were as follows: incidence of in-hospital mortality yielded an RD of 129 fewer per 1000 patients (208 fewer to 3 more) (4 RCTs, *n* = 177), length of ICU stay yielded a MD of 1.6 days shorter (3.3 days shorter to 0.2 days longer) (10 RCTs, *n* = 646), duration of mechanical ventilation yielded a MD of 3.2 days shorter (10.8 days shorter to 4.4 days longer) (3 RCTs, *n* = 155), incidence of infectious complications yielded an RD of 40 fewer per 1000 patients (118 fewer to 80 more) (3 RCTs, *n* = 155), and incidence of all adverse events (diarrhea, vomiting, constipation) yielded an RD of 149 fewer per 1,000 patients (243 fewer to 14 fewer) (14 RCTs, *n* = 964). Therefore, the favorable outcomes were judged as moderate. No unfavorable effects were observed, including adverse events. Based on the overall balance of effects, we thought that the administration of prebiotics was likely superior.

Regarding acceptability, the administration of prebiotics is considered ethically and morally acceptable. Additionally, many food products are available and the cost increase is minimal, making this intervention highly feasible.

**CQ2-7-2: Should probiotics be administered in critically ill patients?**

**Answer**: We suggest administering probiotics to critically ill patients (GRADE 2C: certainty of evidence = “low”).

**Rationale**

Probiotics are live microorganisms that exert beneficial effects on the host when taken in sufficient amounts and are represented by *Lactobacillus* and *Bifidobacterium* spp. In critically ill patients, the intestinal microbiota and intestinal environment are affected by various factors, which decreases the production of short-chain fatty acids [296]. This in turn reduces the activity of intestinal epithelial cells and dilutes the intestinal mucosal layer. These factors promote bacterial translocation, causing a systemic inflammatory response syndrome originating in the intestinal tract. The administration of probiotics is expected to restore a healthy intestinal environment and intestinal microbiota and reduce intestinal inflammation by stimulating the activity of intestinal epithelial cells and immunity through the production of short-chain fatty acids and other substances by viable bacteria in the host [297]. However, it currently remains unclear whether this is effective in patients with severe diseases. Therefore, we consider this to be an important clinical issue that needs to be addressed in JCCNG 2024.

A meta-analysis was performed using 16 RCTs (*n* = 4430) (Additional file 2) [298–313]. The results of the favorable outcomes were as follows: incidence of in-hospital mortality was yielded an RD of 10 fewer per 1000 patients (39 fewer to 18 more) (6 RCTs, *n* = 3307), length of ICU stay yielded a MD of 2.4 days shorter (4.2 days shorter to 0.7 days shorter) (9 RCTs, *n* = 3534), duration of mechanical ventilation yielded an MD of 0.8 days shorter (1.8 days shorter to 0.2 days longer) (7 RCTs, *n* = 3445), and incidence of infectious complications yielded an RD of 72 fewer per 1000 patients (113 fewer to 14 fewer) (13 RCTs, *n* = 4272). Therefore, the favorable outcomes were judged as small. The results of the unfavorable outcomes were as follows: incidence of all adverse events yielded an RD of 12 more per 1000 patients (90 fewer to 143 more) (6 RCTs, *n* = 3112) and physical function yielded a MD of 1 lower (7.96 lower to 5.96 higher) (1 RCT, *n* = 207). Therefore, the unfavorable outcomes were also judged as small. Based on the overall balance of effects, we thought that using the administration of probiotics was likely superior, given the significant reduction in length of ICU stay and infectious complications.

Regarding acceptability, although there are some restrictions due to bacterial species, drug interactions and patients’ conditions, there are no new costs to be borne by patients and negligible burden on medical institutions as a result of the intervention. Probiotics are available at all medical institutions, and the intervention is feasible. Based on the balance of these effects, we concluded that the administration of probiotics was likely superior.

**CQ2-7-3: Should synbiotics be administered in critically ill patients?**

**Answer**: We recommend administering synbiotics to critically ill patients (GRADE 1C: certainty of evidence = “low”).

**Rationale**

Synbiotics are a combination of both prebiotics and probiotics that are expected to exert beneficial effects in the host by improving the bacterial flora in the digestive tract. Furthermore, the ingestion or administration of synbiotics promotes the growth of the bacterial flora in the digestive tract, which contributes to the prevention and attenuation of diseases while maintaining the health of the bacterial flora [314]. However, there is currently no consensus on the effectiveness of synbiotics in critically ill patients. Therefore, we consider investigations on the usefulness of synbiotic interventions in critically ill patients to be of clinical significance and an important clinical issue that needs to be addressed in JCCNG 2024.

A meta-analysis was performed using 12 RCTs (*n* = 1001) (Additional file 2) [224, 315–325]. The results of the favorable outcomes were as follows: incidence of in-hospital mortality yielded an RD of 10 fewer per 1000 patients (66 fewer to 64 more) (6 RCTs, *n* = 614), length of ICU stay yielded a MD of 0.07 days shorter (0.9 days shorter to 0.7 days longer) (12 RCTs, *n* = 988), duration of mechanical ventilation yielded a MD of 1.6 days shorter (4.4 days shorter to 1.3 days longer) (5 RCTs, *n* = 500), incidence of infectious complications yielded an RD of 219 fewer per 1000 patients (288 fewer to 118 fewer) (10 RCTs, *n* = 858), and incidence of all adverse events yielded an RD of 24 fewer per 1000 patients (66 fewer to 91 more) (3 RCTs, *n* = 416). Therefore, the favorable outcomes were judged to be as moderate. No unfavorable effects were observed, including adverse events. Based on the overall balance of effects, we thought that the administration of synbiotics was likely superior.

Regarding acceptability, a prebiotic intervention is included in the cost of the inpatient diet and, thus, there are no new costs to be borne by the patient as a result of the intervention. Although this intervention requires extra efforts by healthcare professionals and additional costs, these increases are negligible when considered for the ICU as a whole. Despite a number of restrictions related to bacterial species, drug interactions, and patients’ conditions, various probiotics may be provided in Japan. Therefore, probiotics and prebiotics are both available at all medical institutions, and interventions are feasible. Furthermore, there are no new costs to be borne by the patient. Based on the balance of these effects, we concluded that the administration of synbiotics was likely superior.

**CQ2-8: What is the concept of vitamin and trace element supplementation in nutrition therapy for critically ill patients?**

**Answer**: Critically ill patients are at high risk for vitamin and trace element deficiencies, and appropriate measurement and supplementation should be considered for them. Unless a severe deficiency is suspected, active supplementation beyond the daily requirements should be warned (provision of information for background question).

**Rationale**

Vitamins and trace elements are essential nutrients for living organisms and are involved in antioxidant effects, cell division, and the maintenance of immune function. Collectively, they are referred to as micronutrients. Critically ill patients often have low blood levels of vitamins and trace elements, which are associated with increased mortality and decreased immune function [67, 326]. There is consensus on the necessity of administering the daily requirements of micronutrients [67, 326], with active supplementation beyond these requirements being anticipated to improve the prognosis of critically ill patients due to their antioxidant, anti-inflammatory, and immunomodulating effects [4, 67, 326]. However, few RCTs have been investigated the effects of micronutrients. Additionally, some RCTs and systematic reviews and meta-analyses reported adverse events [327–329]. Therefore, this is an important clinical issue to be addressed in JCCNG 2024. In this CQ, we summarize the findings of RCTs and meta-analyses on vitamins and trace elements and present them as BQs. The detail results of the systematic review and meta-analysis conducted by the guideline committee on vitamin B1, along with recent guidelines and systematic review/meta-analyses on other vitamins and trace elements, are provided in the additional file (Additional file 6).

**Vitamins**

Vitamin B1 (thiamine) is a water-soluble vitamin and essential coenzyme for carbohydrate and fatty acid metabolism. It plays a critical role in the conversion of pyruvate to acetyl CoA and the citric acid cycle. A deficiency in vitamin B1 leads to energy production disorders. Clinically, lactic acidosis, beriberi, and Wernicke’s encephalopathy are known symptoms of vitamin B1 deficiency. Critically ill patients often have a decreased vitamin B1 intake and increased requirements, making supplementation necessary to avoid severe complications. However, the target conditions and efficacy of vitamin B1 administration in critically ill patients remain inconclusive based on RCTs and meta-analyses [330, 331]. Therefore, we conducted a new systematic review and meta-analysis to evaluate the efficacy of vitamin B1 administration in critically ill patients (Additional file 6).

A meta-analysis was performed using 35 RCTs [327, 332–365]. Vitamin B1 administration in critically ill patients was effective in reducing the duration of shock by a MD of 11.4 h shorter (20.2 h shorter to 2.7 h shorter) and SOFA scores by a MD of 1.3 points lower (1.9 lower to 0.7 lower). However, the length of ICU stay was slightly but significantly prolonged by a MD of 0.4 days (0.01 to 0.8 days longer) [366].

Vitamin C (ascorbic acid) is a water-soluble vitamin with antioxidant and anti-inflammatory effects. Vitamin C deficiency causes cellular damage and decreased catecholamine production due to oxidative stress. Vitamin C supplementation has been effective in critically ill patients with vitamin C deficiency due to the increased demands associated with an invasive condition; however, there is still much debate regarding the target conditions. The Japanese Clinical Guidelines for Management of Sepsis and Septic Shock 2020 (J-SSCG 2020) suggest the administration of vitamin C to septic patients [367]. In 2022, Lamontagne et al. performed an RCT (LOVIT trial) [328] on sepsis patients (*n* = 872) at 35 centers, and found that intravenous vitamin C administration (50 mg/kg every 6 h) was associated with a significant increase in death or persistent organ dysfunction at 28 days (risk ratio (RR) 1.2, 95% CI 1.0 to 1.4). The guideline committee of J-SSCG 2020 conducted an updated meta-analysis of 23 RCTs, including the LOVIT trial (Detail CQ2-8-Supplement-Vitamin C), and revised the recommendation to “We suggest against administering vitamin C to septic patients” [368]. The Surviving Sepsis Campaign Guideline 2021 (SSCG 2021) has a similar recommendation: “For adults with sepsis or septic shock, we recommend not using IV vitamin C” [369]. On the other hand, a recently reported meta-analysis of 18 RCTs (*n* = 3364) on the administration of vitamin C to patients with sepsis showed improvements in Sequential Organ Failure Assessment (SOFA) scores (MD − 0.6 95% CI − 1.0 to − 0.3) and reductions in the use of vasoconstrictors (MD: − 15.1, 95% CI − 21.6 to − 8.6) [329]. However, there was no benefit in short-term mortality (odds ratio (OR): 0.9, 95% CI 0.8 to 1.0), and adverse events, such as organ dysfunction or dysglycemia, were significantly higher with the administration of vitamin C (OR: 2.0, 95% CI 1.1 to 3.7). In the subgroup analysis, there was a significant reduction in short-term mortality in the group administered vitamin C at 25–100 mg/kg/day (OR: 0.8, 95% CI 0.7 to 1.0). To evaluate the effects of vitamin C monotherapy in critically ill patients, a meta-analysis of 16 RCTs (*n* = 2130) showed that vitamin C treatment contributed to a reduction in mortality in critically ill patients (RR: 0.7, 95% CI 0.6 to 0.9) [370]. The findings of systematic reviews and meta-analyses conducted to date suggest that vitamin C alone is effective for patients with severe diseases. However, some adverse events were reported in large RCTs; therefore, it needs to be administered with caution. Further studies on the appropriate dosage, timing, duration of administration, and patient groups are warranted.

Vitamin D is a fat-soluble vitamin that is involved in the absorption of calcium and phosphorus, the regulation of blood levels, an increase in myocardial contractility, and the regulation of immune cells. It is metabolized in the liver to 25-hydroxyvitamin D (25(OH)D), which is subsequently metabolized in the kidneys to the active form, 1,25-dihydroxyvitamin D. Among patients with severe diseases, serum 25(OH)D levels were found to be reduced in critically ill patients and associated with an increased incidence of sepsis and mortality [371]. Since the production of vitamin D3 in the skin of ICU patients is reduced due to limited exposure to ultraviolet B, vitamin D is supplemented either enterally or intravenously. J-SSCG 2020, based on a meta-analysis of 11 RCTs (Additional file 6), suggested against the administration of vitamin D to septic patients [367]. However, this recommendation does not preclude the administration of vitamin D by regular EN. Additionally, SSCG 2021 does not mention the administration of vitamin D [369]. On the other hand, the findings of a meta-analysis of 16 RCTs (*n* = 2449) on the administration of vitamin D to critically ill patients were reported in 2022 [372]. Although the route of administration (intravenous, intramuscular, or oral) and dose (120,000–540,000 IU) varied, the favorable effects of vitamin D administration included reduced mortality (RR 0.8, 95% CI 0.6 to 1.0), a shorter ICU stay (MD: − 3.1 days, 95%CI − 5.4 to − 0.9), and a shorter duration of mechanical ventilation (MD: − 5.1 days, 95% CI − 7.4 to − 2.7). Future high-quality studies are needed to elucidate the appropriate dose, route of administration, and patient population for vitamin D. The partially revised version of the ESPEN ICU clinical nutrition guidelines (2023) [4] also changed its recommendations regarding vitamin D administration. It now emphasizes assessing 25(OH)D levels in patients at risk of vitamin D insufficiency or deficiency.

Although the efficacy of vitamin administration in critically ill patients has been examined in detail, it is important to note that the dosages and methods of administration (alone or in combination) vary as described above, and the need for supplementation in the target patients has not yet been proven. While supplementation for vitamin deficiency is considered essential, further evidence for appropriate dosages and administration methods is needed in the future. Daily reference intakes for vitamins in the Japanese and ESPEN recommendations are shown in Tables 6 and 7.

**Table 6:** Dietary reference intakes for Japanese (2020) (vitamins) [373]

	EAR		RDA		UL	
	Males	Females	Males	Females	Males	Females
Vitamin B1 (mg/day)	1.0–1.2	0.8–0.9	1.2–1.4	0.9–1.1		
Vitamin C (mg/day)	80–85	80–85	100	100		
Vitamin D (µg/day)	8.5	8.5	100	100		
Vitamin A (µg RAE/day)	550–650	450–500	800–900	650–700	2700	2700
Vitamin E (mg/day)	(AI) 6.0–7.0	(AI) 5.0–6.5			750–900	650–700

AI: adequate intake; EAR: estimated average requirement; RAE: retinol activity equivalent; RDA: recommended dietary allowance; RAE: retinol activity equivalent; UL: tolerable upper intake level

**Table 7:** ESPEN recommendations for daily vitamin intakes-2022 [326]

	DRI (31–70 years old)	EN high requirements in 1500 kcal	PN high requirements
Vitamin B1 (mg/day)	1.1–1.2	100	100–200
Vitamin C (mg/day)	75–90	200	200–500
Vitamin D3 (cholecalciferol) (µg/day)	15–20	30	20–25 (800–1000 IU)
Vitamin A (retinol) (µg/day)	700–900	1500	1100
Vitamin E (α-tocopherol) (mg/day)	15	40	20

DRI: dietary reference intake; EN: enteral nutrition; PN: parenteral nutrition high requirements: increased requirements may occur in patients with on-going increased losses, such as gastrointestinal losses, continuous renal replacement therapy, those who are hypermetabolic or who are depleted before commencing PN, and in pregnancy

**Trace elements**

Eight trace elements (iron, zinc, copper, manganese, iodine, selenium, chromium, and molybdenum) are listed in the Dietary Reference Intakes for Japanese (2020) [373]. Serum concentrations of trace elements decrease during critical illness and, thus, appropriate supplementation needs to be considered. Trace element deficiencies due to continuous renal replacement therapy (CRRT) have recently attracted attention. Fah et al. reported that 89.8% of patients in a CRRT group were deficient in one or more trace elements, which was significantly higher than 61.4% of patients in the non-CRRT group (*P* = 0.002). Copper depletion was more frequent (74.5%), followed by selenium (44.4%) and zinc (21.4%) [374]. Therefore, the monitoring of copper, selenium, and zinc in critically ill patients needs to be considered depending on their pathology and treatment [375]. Additionally, the measurement of these trace elements concurrently with C-reactive protein (CRP) levels is recommended because their plasma concentrations are affected by inflammation [326]. Caution needs to be exercised when interpreting measured values, particularly when CRP levels exceed 20 mg/L (SI units) (2.0 mg/dL in units commonly used in Japan). We herein discuss the important roles of selenium, zinc, and copper, which are abundant trace elements in the body, but are frequently deficient in critically ill patients.

Selenium is an important trace element involved in the regulation of oxidative stress by antioxidant activity and in the synthesis and metabolic regulation of thyroid hormones. Selenium deficiency is associated with the development of arrhythmia, cardiomyopathy, and thyroid dysfunction. Risk factors for selenium deficiency include burns, CRRT, EN with long-term central venous feeding or component feeding, and chronic renal failure. In addition, selenium deficiency has been associated with death in critically ill patients [376]. In 2023, a meta-analysis of 24 RCTs reported reductions in all-cause mortality (RR: 0.8 95% CI 0.7 to 1.0) and the incidence in acute kidney injury (RR: 0.7, 95% CI 0.5 to 1.0) with the administration of selenium [377] (Additional file 6).

Japanese guidelines for selenium deficiency suggest the regular monitoring of blood selenium levels in at-risk patients because selenium deficiency may lead to fatal complications. The ESPEN micronutrient guidelines suggest selenium supplementation based on CRP levels, which correlate with plasma selenium concentrations [326]. This highlights the need for further research on the necessity of selenium supplementation in critically ill patients, who often have elevated CRP levels.

Zinc is a trace element that is essential for maintaining immune function, deoxyribonucleic acid synthesis, protein synthesis, and steric stability, among other roles. A previous study examined the impact of zinc supplementation in critically ill patients with acute kidney injury using propensity score matching [378]. Zinc supplementation was associated with lower in-hospital mortality (hazard ratio (HR) 0.5, 95% CI 0.3 to 0.8) and lower 30-day mortality (HR: 0.5, 95% CI 0.3 to 0.9). A subgroup analysis also indicated better outcomes in stage 1 septic acute kidney injury. However, a meta-analysis of two RCTs (*n* = 168) found no mortality benefit with zinc (RR: 0.7, 95% CI 0.4 to 1.3) [379]. The ESPEN micronutrient guidelines recommend simultaneous measurements of CRP and albumin in the assessment of plasma zinc levels because of the possibility of false low levels due to inflammation [326] (Additional file 6).

Copper is essential for energy production, iron metabolism, connective tissue maturation, neurotransmitter production, and the elimination of reactive oxygen species. Deficiency may lead to a number of symptoms, such as anemia, immunodeficiency, arrhythmia, and delayed wound healing. Therefore, supplementation to address copper deficiency is crucial for the prevention of these issues. However, there is a lack of large RCTs on the effects of copper supplementation in critically ill patients, resulting in insufficient evidence. The causes of copper deficiency in critically ill patients include CRRT, gastrointestinal surgery (particularly when nutrients bypass the duodenum), and burns. Therefore, monitoring for copper deficiency is important in these patient groups [380]. An excessive zinc intake may also lead to copper deficiency. Unlike other micronutrients, plasma copper concentrations increase during the acute phase of inflammation. The ESPEN micronutrient guidelines recommend simultaneously measuring CRP when assessing copper levels. They also provide guidance on when copper supplementation needs to be considered based on CRP levels [326].

Daily reference intakes in the Japanese and ESPEN recommendations for trace elements are shown in Tables 8 and 9. Definitions of the terms used in this section to assess micronutrient requirements and the nutritional status are shown in Tables 10 and 11.

- **3. Nutrition monitoring and specific conditions**

**Table 8:** Dietary reference intakes for Japanese (2020) (trace elements) [373]

	EAR		RDA		UL	
	Males	Females	Males	Females	Males	Females
Iron (mg/day)	6.0–6.5	5.0–5.5 (menstruating) 8.5–9.0	7.0–7.5	6.0–6.5 (menstruating) 0.5–11.0	50	40
Zinc (mg/day)	9	6–7	10–11	8	40–45	30–35
Copper (mg/day)	0.7	0.6	0.8–0.9	0.7	7	7
Manganese (mg/day)			(AI) 4.0	(AI) 3.5	11	11
Iodine (µg/day)	95	95	130	130	3000	3000
Selenium (µg/day)	25	20	30	25	400–450	350
Chromium (µg/day)			(AI) 10	(AI) 10	500	500
Molybdenum (µg/ day)	20–25	20	25–30	25	600	500

AI: adequate intake; EAR: estimated average requirement; RDA: recommended dietary allowance; UL: tolerable upper intake level

**Table 9:** ESPEN recommendations for daily trace element intakes-2022 [326]

	DRI (31–70 years old)	EN high requirements in 1500 kcal	PN high requirements
Iron (mg/day)	8 (19–50 years old females 18 mg)	30	1
Zinc (mg/day)	8–11	20	6–12
Copper (mg/day)	0.9	1–3	0.5–1.0
Manganese (mg/day)	1.8–2.3	2–3	55
Iodine (µg/day)	150	150–300	130
Selenium (µg/day)	55	200	150–200
Chromium (µg/day)	20–35	200	15
Molybdenum (µg/day)	45	250	19–25

DRI: dietary reference intake, EN: enteral nutrition, PN: parenteral nutrition high requirements: increased requirements may occur in patients with on-going increased losses, such as gastrointestinal losses, continuous renal replacement therapy, those who are hypermetabolic or who are depleted before commencing PN, and in pregnancy

**Table 10:** Terminology related to micronutrient requirements [326]

Terminology	Definition
Estimated average requirement (EAR)	The average daily nutrient intake level estimated to meet the requirement of 50% of healthy individuals in a particular life stage and sex group It is equivalent to the term Average Requirement (AR) in the European Union (EU)
Recommended dietary allowance (RDA)	The average daily dietary nutrient intake level sufficient to meet the nutrient requirement of nearly all (97–98%) healthy individuals in a particular life stage and sex group. This concept is equivalent to Population Reference Intake (PRI) in the EU
Tolerable upper intake level (UL)	Daily MN doses that are safe to take without the risk of an overdose or serious side effects The highest average daily nutrient intake level likely to pose no risk of adverse health effects to almost all individuals in the general population
Adequate intake (AI)	The recommended average daily intake level is based on observed or experimentally selected approximations or estimates of nutrient intake by a group (or groups) of apparently healthy individuals that are assumed to be adequate; used when an RDA cannot be set
Dietary reference intake (DRI)	Set of reference values, including EAR, AI, RDA, and UL, that, when adhered to, predict a low probability of nutrient inadequacy or excessive intake

**Table 11:** Definitions in a nutritional status assessment [326]

Status	Definition
Adequate	Blood/plasma concentrations are within the local reference range (international range if no national reference is available), and the absence of any clinical signs or symptoms related to micronutrients (MN) The status may be adequate despite a low plasma value in a patient with inflammation
Depletion	The presence of an objective loss of a MN in body fluids, or intake below the standard recommendation with blood/plasma concentrations are below the reference range
Deficiency	Evidence for the objective loss of a MN in body fluids, or intake below the standard recommendation AND EITHER: The presence of clinical signs or symptoms, compatible with a MN deficiency OR blood/plasma concentrations below the reference range together with metabolic effects of inadequacy
Overdose	Detection (by monitoring blood concentrations) of higher than upper reference values, associated with the administration or intake (accidental or intentional) of amounts greater than the recommendations

MN: micronutrient

**CQ3-1: Is a nutritional assessment necessary before providing nutrition therapy to critically ill patients?**

**Answer**: Nutritional assessment is necessary before providing nutrition therapy to critically ill patients (Good Practice Statement).

**Rationale**

In critical illness, inflammation and increased catabolism may induce nutritional disorders, even in patients with a good nutritional status before the onset of disease. Therefore, critical illness is considered a risk factor for nutritional disorders, and a nutritional assessment is required for almost all patients. A nutritional assessment is defined here as the combination of nutritional screening to identify nutritional risk (the risk of having a nutritional disorder and related complications), a nutritional assessment to diagnose nutritional disorders, and a complete assessment to obtain details on the nutritional status necessary for individualized nutrition therapy. Since a nutritional assessment is an essential step in nutrition therapy, there have been no comparisons of outcomes with and without this assessment; however, many studies reported that patient outcomes (death and complications) were univocally related to the findings of a nutritional assessment [381, 382]. The concepts of nutritional screening and a nutritional assessment have not yet been clearly defined, and neither are well differentiated in real-world clinical practice and research. Therefore, this CQ will discuss the significance and concepts of nutritional screening and a nutritional assessment.

The process of nutrition therapy is generally understood to be screening, assessment, and treatment, in that order. Nutritional screening is the first step in nutrition therapy to identify patients at risk of developing nutritional disorders and related complications, even in critically ill patients. ASPEN recommends assessing the nutritional risk of all patients admitted to the ICU [381, 382].

A nutritional assessment consists of a diagnostic assessment to diagnose the presence and severity of nutritional disorders (primary assessment) and a full assessment to collect and evaluate detailed information necessary for nutrition therapy (complete assessment), which are collectively defined as nutritional assessment in these guidelines. These processes allow for the development of appropriate nutrition therapy plans [381, 383]. There is virtually no evidence to recommend a uniform tool for conducting nutritional screening and a nutritional assessment. On the other hand, each ICU or ward needs to use the same assessment concept as much as possible based on assessment concepts. A nutritional assessment needs to be repeated and reflected in the nutrition therapy plan as needed, bearing in mind that the general condition of a patient, including nutritional status, may change from day to day.

A search for nutritional assessment showed many publications that demonstrated the effectiveness of screening tools [381, 384], and ASPEN and ESPEN have accepted nutritional screening as part of a nutritional assessment. The term “nutritional assessment” has several definitions and may or may not include nutritional screening.

The nutritional screening of critically ill patients involves the identification of patients at nutritional risk. Of particular concern in critically ill patients is the high probability of nutrition disorders due to rapid protein catabolism [384] and impaired digestion and absorption [385], even in patients with a good nutritional status prior to the onset of severe disease. Correspondingly, a uniform nutritional screening tool may allow for an objective assessment of the nutritional status and appropriate nutrition therapy for individual patients [386]. Screening tools range from those specific to critically ill patients to those used in general wards, and many studies have compared the effectiveness of nutritional screening tools in critically ill patients. Screening tools commonly used in general wards and ICUs include the Malnutrition Screening Tool [387], Malnutrition Universal Screening Tool [388], Mini Nutritional Assessment Short Form, Nutritional Risk Screening (NRS) 2002 [389], which is specific to the acute setting, and the Perioperative Nutrition Score [390], which screens for perioperative undernutrition, often using BMI and recent weight loss as the assessment index.

Nutritional scoring, which scores items related to the nutritional status to predict outcomes and establish the risk of developing malnutrition (nutrition disorder) and related complications, may be used as nutritional screening or as part of a nutritional assessment. The Prognostic Nutritional Index (PNI), which is commonly used in Japan, was developed to predict the development of complications in gastrointestinal surgical patients. Other nutritional scoring systems include the Nutritional Risk Index (NRI) [391, 392], which has been suggested to be associated with outcomes in patients admitted to the ICU; the Geriatric Nutritional Risk Index [393], a modified version of the NRI for the elderly; and the Controlling Nutrition Status [394], which reflects the nutritional status and immunocompetence. A nutritional assessment of patients admitted to the ICU includes the Nutrition Risk in Critically Ill Score (NUTRIC) recommended by ASPEN guideline 2016, its modified version, the modified NUTRIC [381, 395–397], NRS 2002 [398], and the Screening of Nutritional Risk in Intensive Care risk prediction score [399] (Supplemental Table 1). Although the current guidelines do not recommend a specific nutritional screening and scoring tool, we define nutritional screening as the first step in nutrition therapy planning.

Nutritional assessments for a diagnosis include the Subjective Global Assessment (SGA) [400]; Patient-generated SGA, and the Mini Nutritional Assessment (MNA) [401, 402]. The Global Leadership Initiative on Malnutrition (GLIM) criteria, an international diagnostic basis for malnutrition, is used for the diagnosis and assessment of severity of malnutrition. One feature of these diagnostic criteria is the assessment of muscle mass, which corresponds to the assessment for a diagnosis. Although GLIM has not yet been fully evaluated in critically ill patients, a systematic review and meta-analysis by Diaz et al. showed that ICU undernourished patients selected by the GLIM criteria had high sensitivity and specificity for outcome predictions [403]; therefore, its use may be considered in ICU patients in the future. A nutritional assessment needs to be repeated because of the ever-changing nutritional risks that individual patients have in critical illness.

Items in a detailed nutritional assessment include a nutritional intake survey, medical examinations, such as visual and palpatory examinations, physical measurements, including body composition, blood and biochemical tests, physiological function tests, such as IC and respiratory function, and swallowing function. In body measurements, a lean body mass is a more important indicator than body weight, which is more sensitive to extracellular fluid [404, 405]. Blood and biochemistry test items vary according to the individual pathology; however, several common items need to be considered. In critically ill patients, albumin and prealbumin are not nutritional endpoints because they indicate an inflammatory status and are affected by disease severity. On the other hand, the total lymphocyte count, a measure of immunocompetence, is often used in a nutritional assessment [406]. Potassium and sodium, in addition to providing information on the pathophysiology, are important in the selection of formulations and nutrients for nutrition therapy. In addition, phosphorus and magnesium are useful indicators for assessing the risk of refeeding syndrome. Edema, ascites, and pleural effusion, which have a significant impact on weight changes, are also part of a nutritional assessment because they are often factors contributing to significant weight gain prior to ICU admission and weight loss during the ICU stay. Anemia due to gastrointestinal bleeding, pressure ulcers associated with prolonged malnutrition, and gastrointestinal symptoms, including diarrhea and constipation, also need to be identified and comprehensively evaluated in nutrition therapy. Moreover, dietary intake and medications prior to ICU admission are important for individualized nutrition therapy. In the future, it may be necessary to consider making a physical function assessment (SF-36) and frailty assessment (Clinical Frailty Score) a part of a nutritional assessment from the perspective of PICS [407–409].

**CQ3-2: Should indirect calorimetry be used to estimate energy expenditure in critically ill patients?**

**Answer**: We suggest using indirect calorimetry for estimating energy expenditure (EE) in critically ill patients (GRADE 2B: certainty of evidence = “moderate”).

**Rationale**

The estimation of EE is important in the design of nutritional administration for critically ill patients. Methods for estimating EE include indirect calorimetry (IC) and various predictive equations. IC is considered more necessary under conditions where body weight is inaccurate or metabolic dynamics markedly change [4, 125, 410]; however, there are issues with the limited number of facilities available in Japan, measurement conditions, techniques, and costs. On the other hand, predictive equations have limited accuracy. An important clinical issue is clarifying whether it is worthwhile to use IC or if the use of predictive equations is sufficient.

A meta-analysis was performed using 9 RCTs (*n* = 1178) (Additional file 3) [68, 411–418]. The results of the favorable outcomes were as follows: the results of effects for short-term mortality (< 90 days or in-hospital death) decreased by 36 per 1000 patients (95% CI 77 fewer to 15 more) (7 RCTs, *n* = 988). Therefore, the favorable outcomes were judged as small. The results of the unfavorable outcomes were as follows: length of ICU stay yielded a standardized mean difference (SMD) of 0.9 longer (95% CI 1.0 shorter to 2.7 longer) (7 RCTs, *n* = 1090) and total infection an RD of 13 more per 1000 (95% CI 40 fewer to 82 more) (4 RCTs, *n* = 785). Therefore, the unfavorable outcomes were judged as trivial. Based on the overall balance of effects, we thought that using IC was likely superior.

IC is regarded as non-invasive and acceptable for each patient, and the techniques required for measurements in modern devices are not difficult [419]. However, IC is more likely to be used exclusively for patients on mechanical ventilation. Additionally, the introduction of indirect calorimeter is costly in its own right because running costs are required for each measurement per patient, and since there is currently no insurance coverage for the measurement procedure, depreciation is not expected in Japan. Although it is highly acceptable for facilities that already have indirect calorimeters, it is not possible to perform measurements for all critically ill patients due to cost and time constraints.

IC is considered superior to predictive equations in terms of accuracy and the goal of personalized medicine; however, it is important to note that for facilities that do not possess indirect calorimeter, a moderate amount of resources is required. In addition, although the accuracy of the device is improving [419], it may be less accurate or difficult to perform for patients requiring non-intubated ventilation, extracorporeal membrane oxygenation (ECMO), high F_I_O_2_, advanced ventilator settings, or patients with pneumothorax.

In a post hoc analysis of a meta-analysis, the discrepancy between estimated and actual doses and differences in the timing and frequency of measurements among RCTs were identified as issues [420]. Future studies on the cost-effectiveness of IC and its effects on specific patient groups are also necessary.

**CQ3-3: What is the role of nitrogen balance in critically ill patients?**

**Answer**: Nitrogen balance reflects the increase or decrease of protein in the body and may be an indicator to assess systemic protein anabolism (provision of information for background question).

**Rationale**

During starvation, glycogen in the liver and muscles is used as an energy source, and fat and muscles are used with further starvation. When body weight, excluding fat, decreases to approximately 70% of normal weight, life cannot be maintained, leading to death. This is called nitrogen death in Japan. Therefore, monitoring changes in the systemic protein content corresponding to lean body mass is of clinical significance.

The nitrogen balance is the amount of nitrogen in administered protein minus the amount of nitrogen excreted (such as in urine, feces, skin, pleural fluid, ascites, and exudates). It is an indicator of an increase or decrease in protein in the organism. However, since difficulties are associated with accurately measuring nitrogen excretion and evaluations of its effectiveness have yet to be established, whether it is worth assessing nitrogen balance is an important clinical issue.

The nitrogen balance is the difference between the amount of nitrogen present in ingested proteins and amino acids and the amount of nitrogen excreted [421]. Ingested proteins are absorbed in the form of amino acids and peptides, some of which are free amino acids. Free amino acids are stored in the body as an amino acid pool and used synthetically, mainly in proteins. When systemic protein synthesis and breakdown are equal and body homeostasis is maintained, the amount of nitrogen ingested is equal to the amount of nitrogen excreted by urine and feces, thereby creating a state of nitrogen equilibrium. When nitrogen equilibrium is disrupted and the nitrogen balance is tilted to the positive or negative side, anabolism or catabolism is predominant.

The nitrogen balance is obtained by [amount of nitrogen ingested−amount of nitrogen excreted], and the amount of nitrogen ingested (g/day) is calculated by [amount of protein ingested (g/day)/6.25]. There are several methods for calculating the amount of nitrogen excreted [422–427] (Table 12), and [urinary nitrogen excretion divided by 0.8] is convenient. However, nitrogen is excreted by urine, feces, sweat, and exudates, and is also excreted as urea nitrogen in urine as well as ammonia and uric acid. The amount of nitrogen excreted also affects changes in blood urea nitrogen.

**Table 12:** Examples of formulas to calculate the amount of nitrogen excreted

Urine urea nitrogen in urine (g/day) + 4 [422]
Add 4 g of nitrogen excreted from sweat or stools to the urinary urea nitrogen content
Urine urea nitrogen in urine (g/day) + 2 [423]
Add 2 g of nitrogen excreted from sweat or stools to the urinary urea nitrogen content
Urine urea nitrogen (g/day)/0.85–2 [424]
Correct by dividing the urinary urea nitrogen content by 0.85 and subtract 2 g
Urine urea nitrogen (g/day)/0.8 [425]
The total amount of nitrogen in urine may be estimated by dividing the amount of urea nitrogen in urine by 0.8. However, nitrogen in stools and sweat is not considered
Urine urea nitrogen (g/day)/0.85 [426]
The total amount nitrogen in urine may be estimated by dividing the amount of urea nitrogen in urine by 0.85. However, nitrogen in stools and sweat is not considered
Urine urea nitrogen (g/day) + 2 ± ΔBUN [427]
Add 2 g of other nitrogen losses to the amount of urinary urea nitrogen and consider the amount of change in blood urea nitrogen. Evaluate the amount of nitrogen lost by a daily increase or decrease in blood urea nitrogen

ESPEN guideline 2018 and ASPEN guideline 2016 recommend the use of the nitrogen balance to adjust protein dosages in obese patients [67, 125]. A systematic review published in 2022 evaluated 8 studies and found that the absolute nitrogen balance at ICU admission was not associated with mortality, while an improved nitrogen balance after ICU admission was associated with reduced mortality [428].

Previous studies demonstrated that the nitrogen balance improved in critically ill patients when more protein was administered [101, 429–431]. However, there are currently no RCTs showing that protein administration improves the nitrogen balance. Although rapid muscle loss occurs early, up to day 7, in critically ill patients [384], few studies have examined its relationship with the nitrogen balance after day 7. A prospective observational study showed that the cumulative nitrogen balance between day 2 and 10 from ICU admission was positively correlated with the change in thigh muscle volume between day 1 and 10 of admission [432]. Therefore, the nitrogen balance may be used in acute care to assess changes in muscle mass and lean body mass [2].

Difficulties are associated with assessing the exact nitrogen balance because the absorption rate of nitrogen from the diet and EN, as well as unquantified amounts of nitrogen lost, such as in feces and sweat, need to be considered. In critically ill patients, the daily nitrogen balance fluctuates widely [433], making it difficult to accurately assess with a single measurement. To adjust for daily measurement errors as much as possible, the nitrogen balance needs to be considered as cumulative accumulation up to that day. In addition, since few RCTs have examined the relationship between the nitrogen balance and clinical outcomes in critically ill patients, further research is warranted.

**CQ3-4: How is enteral feeding intolerance assessed in critically ill patients?**

**Answer**: Enteral feeding intolerance is assessed using a combination of gastric residual volume, gastric residue properties, abdominal physical findings, imaging findings such as abdominal ultrasonography and abdominal radiographs, and lactate levels (provision of information for background question).

**Rationale**

While EN is recommended for critically ill patients, several factors, such as shock, intestinal edema, sedatives, and narcotic analgesics, may easily cause hypoperistalsis and impair digestion and absorption [434–436]. Furthermore, it is essential to evaluate EFI before and during EN because lethal complications, such as perforation and mesenteric ischemia, must be treated as soon as possible. On the other hand, there is currently no clear definition in Japan or in other countries, and specific monitoring and evaluation methods have not been sufficiently examined. Therefore, how to evaluate EFI is an important clinical issue.

Previous studies defined EFI as an increased gastric residual volume (GRV), nausea and vomiting, severe diarrhea, abdominal distention (feeling), abdominal pain, and abdominal discomfort. The incidence of EFI in critically ill patients widely varies from 2 to 75%, which is due in part to differences in definitions, the duration of observations, and administration methods among institutions [437].

In their systematic review, Blaser et al. divided the pattern for defining EFI in critically ill patients into four categories: (1) increased GRV with gastrointestinal symptoms, (2) increased GRV only, 3) gastrointestinal symptoms only, and 4) the inadequate delivery of enteral feeds, reporting an overall incidence of 38.3% (95% CI 30.7–46.2%). Yahyapoor et al. defined EFI as increased GRV (> 250 mL in 6 h) with two or more gastrointestinal symptoms and reported that during one week of observation after admission, EFI peaked at 66.1% on day 1 and 91.8% on day 2, and thereafter decreased to 38.8% on day 7 [438]. Gungabissoon et al. defined EFI as requiring the interruption of enteral feeding due to increased GRV, abdominal distention, vomiting, diarrhea, or abdominal discomfort, and detected it in 30.5% of critically ill patients [439]. Despite differences in its definition, the incidence of EFI is not low, and some studies indicated that it increased mortality, the length of ICU stay, and infectious complications [440–443].

A common indicator of EFI is the monitoring of GRV [444]. Increased GRV has been reported as the most frequent indicator of EFI [437–439] and is quantifiable and less invasive to patients. Therefore, it has become a benchmark for initiating and increasing or decreasing enteral feeding and starting prokinetic drugs in the ASPEN and ESPEN guidelines [4, 125, 445]. However, the monitoring of GRV may be affected by body position, the production of gastric juice itself, the type of tube, its tip location, and measurement intervals [446, 447]. In addition, although the monitoring of GRV may reduce the risk of emesis, no relationship to mortality, hospital stays, or the incidence of pneumonia has been demonstrated [448–450]. A Cochrane review reported in 2021 did not find any usefulness for monitoring GRV, including the optimal frequency (within 8 h or more than 8 h), threshold, and whether to return it to the stomach or discard it once aspirated [450]. Further research is necessary because there have only been one or two RCTs on each of these categories. In addition, there are concerns about the lack of energy administered due to the interruption of EN and the associated risk of infectious complications, as well as the increased cost of care, mainly due to the extra workload on healthcare workers. While this perspective exists, extremely high GRV needs to be considered as a reason to discontinue EN, and the Japanese guidelines for nutrition support therapy in adult and pediatric critically ill patients (JCCNG 2016, in Japanese only) stated that “enteral nutrition should not be discontinued if GRV is less than 500 mL” [20], while ESPEN guideline 2023 stated that “if GRV exceeds 500 mL per 6 h, the start of enteral nutrition should be delayed”, with a relatively high threshold of 500 ml GRV as the cut-off [4]. However, ESPEN guideline 2023 also stated that the confirmation of GRV was not mandatory for monitoring after EN has been established.

Although there is insufficient evidence to evaluate EFI by monitoring GRV alone, an increase in GRV suggests impaired bowel movement in the upper gastrointestinal tract, and other nutrition delivery routes, such as post-pyloric feeding, are deemed necessary if EN cannot be increased. In critically ill patients in whom the evaluation of EFI is important, a multifaceted assessment needs to be considered, combining not only GRV monitoring, but also other assessment indicators (gastrointestinal symptoms, gastric residue properties, abdominal physical examination findings, imaging findings, such as abdominal ultrasound and abdominal radiographs, and lactate levels).

**CQ3-5: How can the risk of aspiration be reduced in critically ill patients receiving enteral nutrition?**

**Answer**: Methods to reduce the risk of aspiration include continuous feeding, post-pyloric feeding, adjusting the patient's position, and pharmacological interventions (provision of information for background question).

**Rationale**

In critically ill patients receiving EN, inappropriate increases in feeding rates or insufficient monitoring for gastrointestinal intolerance may lead to gastroesophageal reflux and vomiting, which may, in turn, result in aspiration and increase the risk of pneumonia [451]. Ventilator-associated pneumonia (VAP) accounts for 80% of hospital-acquired pneumonia episodes [452]. This may lead to the increased use of antibiotics, a worsening nutritional status, and prolonged hospital stays. Therefore, it is essential to consider measures that reduce the risk of aspiration pneumonia in critically ill patients receiving EN.

Evidence-based interventions for the prevention of hospital-acquired pneumonia include measures to prevent the colonization of harmful bacteria in the gastrointestinal tract and airways. These measures involve avoiding unnecessary antibiotics and antacids, conducting selective digestive decontamination, and administering short-term prophylactic antibiotics to high-risk patients. Additionally, preventing the aspiration of contaminated secretions is critical and may be achieved by proper ICU staff allocation, maintaining a semi-recumbent position, avoiding gastric distension, using oral intubation, and shortening the duration of mechanical ventilation [453].

While the tracheal tube cuff isolates the gastrointestinal tract from the airways in patients receiving mechanical ventilation, the complete prevention of gastric content leakage around the cuff may not be achievable [452]. A previous study reported that elevating the head of the bed to 30–45 degrees correlated with a reduction in the incidence of pneumonia [454]; therefore, this positioning is recommended, particularly for patients receiving EN via a nasogastric tube.

Other components of the VAP prevention bundle include hand hygiene, appropriate suctioning, the use of subglottic secretion drainage endotracheal tubes, heated humidifiers, maintaining light sedation with a Richmond Agitation-Sedation Scale score of − 1 to 1, oral hygiene with chlorhexidine, maintaining cuff pressure between 20 and 30 mmHg, and minimizing unnecessary circuit changes [453].

Conventional methods, including the use of dyes, such as methylene blue, to detect aspiration, have low sensitivity [455]. In high-risk patients, the potential harm from these tests may outweigh the benefits, suggesting that they need to be avoided.

Increased GRV or vomiting during EN is associated with the development of pneumonia [440]. JCCNG 2024 weakly recommend continuous feeding over intermittent feeding, which is consistent with most guidelines [20, 67, 456] (see WG1CQ9 for details). A systematic review on GRV measurements in critically ill patients found no relationship between not measuring GRV and an increase in VAP [457]. Therefore, the routine measurement of GRV for the purpose of preventing aspiration may have limited significance [458].

Positioning the tip of the feeding tube beyond the pylorus has been associated with a reduction in pneumonia [459], and these guidelines weakly recommend this practice. The latest ESPEN guidelines suggest starting with gastric feeding and switching to post-pyloric feeding for patients at a high risk of aspiration (such as those using antacids, in the supine position, undergoing re-intubation, with tracheostomy, ARDS, head trauma, intracranial pressure monitoring, or an advanced age [440], see WG1CQ8 for details on post-pyloric feeding).

Studies on body positioning indicate that elevating the head and placing ventilated patients in the right lateral position during EN is associated with a reduction in GRV from that in the supine position [460]. However, the impact on VAP has yet to be examined. A systematic review of prone positioning during EN in critically ill patients found no significant difference in GRV or the incidence of VAP from those in the supine position [461]. Safe positioning for EN in agitated patients has not been investigated [382]. Although head elevation is often recommended during EN [460], comparisons with the supine position remain inconclusive.

Regarding the interruption of EN during procedures, a previous study on severe burn patients found no significant difference in mortality or aspiration pneumonia with the continuation or interruption of EN during surgery; however, the continued group had an increased caloric intake [462].

In pharmacological studies, methylnaltrexone, an opioid antagonist (naldemedine is available in Japan), is expected to counteract opioid-induced constipation and reductions in peristalsis. However, an RCT on ventilated patients using methylnaltrexone did not show a significant preventative effect against VAP [463]. These guidelines strongly recommend the use of synbiotics in critically ill patients. An RCT on the use of probiotics (lactic acid bacteria) for 14 days in ventilated patients showed a reduction in VAP and shorter ICU and hospital stays [306] (see WG2CQ7 for details).

Semi-solidified EN formulas have been developed to prevent aspiration pneumonia and gastroesophageal reflux [464]; however, due to the lack of standardization in content, administration methods, and viscosity measurement techniques, few RCTs are available.

Regarding physical therapy, an RCT examined the effects of abdominal massage performed twice daily for 15 min on mechanically ventilated patients, and found that it reduced GRV, decreased abdominal distension, normalized stool consistency, increased bowel movement frequency, and reduced the incidence of VAP [465]. In an RCT that compared acupuncture to prokinetic drugs in mechanically ventilated patients with neurosurgical conditions, the acupuncture group showed a reduction in GRV; however, VAP outcomes were not assessed [466].

**CQ3-6: How can diarrhea and constipation be managed in critically ill patients?**

**Answer**: There are several methods, including the selection of nutrition formulas and administration methods, pharmacotherapy, and the use of bowel management systems (provision of information for background question).

**Rationale**

The incidence of diarrhea in critically ill patients ranges between 3.3 and 78% and that of constipation between 20 and 83% [467]. Diarrhea and constipation not only decrease the QoL of patients, but also disrupt hemodynamic stability, affect the absorption of oral medications and nutrients, and may extend ICU stays [468]. Additionally, these conditions may hinder the achievement of energy intake goals by EN. The absorption of administered nutrients may be impaired, resulting in their ineffective utilization. Therefore, managing diarrhea and constipation (by pharmacotherapy, the type of nutritional formulas, and administration methods) in critically ill patients is a significant challenge.

In critically ill patients, diarrhea occurs in cases of sepsis, hypoalbuminemia, EN, and antibiotic treatment. The causes of diarrhea vary, and EN is considered one of the contributing factors [469]. Diarrhea in critically ill patients lacks a consistent definition. It is often defined as watery stools three or more times per day [470]; however, since healthy individuals may also exceed three bowel movements per day and fewer instances of watery stools are not necessarily normal, many facilities use stool consistency alone, assessed using tools such as the Bristol Stool Scale, rather than frequency [471]. Definitions based on stool volume include 300 mL/day [472], 200 g/day [473], and 250 mL/day [474]; however, the measurement of these volumes in critically ill patients is challenging, and it is impractical to place rectal catheters in all cases for measurement.

A systematic review that examined the epidemiology of diarrhea and constipation in critically ill patients reported that the frequency of diarrhea ranged between 3.3 and 78%, and diarrhea did not affect the duration of mechanical ventilation, ICU stay, or survival rates [467]. However, a correlation was observed between diarrhea and a number of conditions, such as ileus, acute kidney injury, metabolic acidosis, hypocalcemia, and steroid use, as well as severe sepsis [475]. Diarrhea may lead to malabsorption, malnutrition, electrolyte imbalances, dehydration, infections, and skin breakdown. It incurs additional costs due to the need for patient isolation and testing to exclude *Clostridioides difficile* infection, and it may prolong ICU stays [102, 470, 474, 476]. In an observational study on critically ill patients receiving EN, those with stool weights > 350 g per day more frequently developed malabsorption of 627 kcal/day than those with stool weights < 350 g per day [477]. Diarrhea is associated with a decrease in the QoL of patients and an increased workload for nurses [478, 479].

Diarrhea is often not infectious in critically ill patients. Special attention is needed for *Clostridioides difficile* infection when administering quinolone and cephalosporin antibiotics; however, *Clostridioides difficile* infection and norovirus infection were previously shown to account for only approximately 1 and 0.1% of cases, respectively [474]. Drug-induced diarrhea is common, with antibiotics, laxatives, prokinetic agents, and antacids being common causes [480]. More than 20% of diarrhea cases involved the use of stimulant laxatives just before the onset of symptoms [474].

EN may cause diarrhea in critically ill patients, which requires an evaluation of its nutritional content, administration method, and bowel function for proper management [470]. An observational study in the ICU involving patients receiving EN identified factors contributing to diarrhea, including the duration and volume of EN, high blood urea nitrogen levels, the use of probiotics, and the presence of respiratory diseases [481]. When diarrhea occurs, attention must be paid to the osmolarity of the EN formula, the rate of administration, and microbial contamination [470]. Continuous feeding, as opposed to intermittent feeding, has been shown to reduce the frequency of diarrhea. The present guidelines weakly recommend continuous feeding (see CQ1-9 for details). Previous studies demonstrated that the use of semi-elemental formulas in critically ill patients may suppress diarrhea and potentially reduce gastrointestinal intolerance [102, 482–485]. However, the present guideline suggests against administering enteral nutrition with semi-elemental or elemental formulas due to the observed worsening of mortality outcomes, despite their effectiveness at reducing diarrhea (see CQ2-3 for details).

A systematic review on the use of synbiotics in critically ill patients showed that while there were variations in the types, amounts, and methods of administration, they effectively reduced diarrhea. However, there were no improvements in intestinal tolerance or overall prognosis [486]. The present guidelines strongly recommend the use of synbiotics (see CQ2-7 for details). In an RCT on septic patients requiring mechanical ventilation, the use of soluble dietary fiber effectively attenuated diarrhea [280]. The present guidelines strongly recommend the administration of prebiotics containing soluble dietary fiber to critically ill patients (see CQ2-7 for details). According to a systematic review by the Eastern Association for the Surgery of Trauma, loperamide and diphenoxylate/atropine (not approved in Japan) were effective in the treatment of non-infectious diarrhea in patients with multiple trauma [487].

The use of rectal catheters in a fecal management system (FMS) was performed on more than 60% of acute cases outside the ICU, primarily for the management of diarrhea. Adverse events, such as fistulas or mucosal necrosis, were not reported; however, 4% experienced stool leakage around the catheter [488]. The use of FMS increases when diarrhea persists for more than five days [489]. Nurse satisfaction with FMS was previously shown to be 69%, with satisfaction increasing to 82% in cases in which diarrhea persisted more than 15 days [489]. However, FMS in the ICU requires caution due to the risk of rectal ulcers [490].

Since the causes of diarrhea are diverse and require comprehensive management, the implementation of protocols is recommended [470]. An observational study in a cardiac care unit where an excretion management protocol was used showed a correlation between protocol implementation and a reduction in the frequency of diarrhea. However, the overall adherence rate to the protocol was only 2.3%. Therefore, the effectiveness of the protocol needs to be re-evaluated with improved adherence rates [491].

Constipation is defined in the Chronic Constipation Clinical Guidelines 2017 [492] as “a condition in which stool that should normally be excreted cannot be passed in sufficient quantities and comfortably”. However, it is often also defined as “a state in which there is no bowel movement for three consecutive days” [493]. There are several definitions [494, 495]. Since critically ill patients often have difficulty expressing their symptoms, various clinical information needs to be gathered for an assessment. The epidemiology of constipation includes simple bowel diseases and conditions caused by severe illnesses. Due to these different definitions, the reported incidence of constipation in critically ill patients varies widely from 20 to 83% [467, 496–499].

A meta-analysis of prophylactic laxative use in critically ill patients to improve outcomes [467] showed no effects on ventilator duration, ICU stay, or mortality rates. A small-scale RCT targeting mechanically ventilated patients reported that the daily administration of lactulose for constipation management improved the SOFA score and shortened both the duration of mechanical ventilation and hospital stay [283]. An observational study comparing patients who had their first bowel movement within six days of ICU admission with those who did not find that early bowel movements were associated with reduced fever, decreased CRP levels, improved SOFA scores, and shorter ICU stays [496].

An observational study conducted at 44 ICU facilities reported that constipation management protocols were implemented in 79.5% of ICUs. The most commonly used medications were senna (81%) and bisacodyl (75.6%), while lactulose (29.7%) and magnesium (13.5%) were less common. Protocols were primarily managed by nurses (62.8%), and the main reasons for protocol activation included the absence of bowel movements for 24–96 h (35.1%), opioid use (18.9%), and high-risk cases (13.5%). However, there is no clear evidence regarding outcomes, and the ideal management of constipation has not been established [500]. In an RCT using synbiotics for patients with respiratory diseases requiring ICU care, constipation was reduced and no adverse events were observed [312]. These guidelines strongly recommend the administration of synbiotics to critically ill patients (see CQ2-7 for details).

Few ICUs have robust protocols for bowel management, and since only a small number of large-scale studies have been conducted to date, the causal relationship between constipation and poor outcomes remains unclear. However, bowel management may ameliorate gastrointestinal disorders and potentially improve the prognosis of critically ill patients [468]. Constipation and diarrhea are closely related. When polyethylene glycol was used as a preventive measure for constipation in critically ill patients, a correlation was observed between a decrease in constipation and an increase in diarrhea [501]. Therefore, care must be taken to monitor for diarrhea when using laxatives.

**CQ3-7: What is the approach to nutrition therapy for critically ill patients who are obese or underweight?**

**Answer**: For critically ill patients who are obese or underweight, nutrition therapy will be individually determined based on patient's condition, including energy and protein targets according to actual body weight, ideal body weight, or adjusted body weight (provision of information for background question).

**Rationale**

The WHO defines a BMI < 18.5 kg/m^2^ as underweight and ≥ 30 kg/m^2^ as obese, while the Japanese obesity guidelines define BMI < 18.5 kg/m^2^ as underweight and ≥ 25 kg/m^2^ as obese [502]. Obesity and being underweight may both be associated with nutritional disorders, which often require special consideration when providing nutrition therapy; therefore, nutrition therapy for obese and underweight patients with critical illnesses needs to be discussed as an important clinical topic. Nutrition therapy for these patients is documented in the ASPEN and the United States Society of Critical Care Medicine (SCCM) Guidelines, the ESPEN Guidelines, and the JSICM Guidelines.

Obesity is defined as BMI ≥ 25 kg/m^2^ and severe obesity as BMI ≥ 35 kg/m^2^ in Japan’s Obesity Treatment Guidelines 2022. On the other hand, the WHO defines obesity as BMI ≥ 30 kg/m^2^ and severe or morbid obesity as BMI ≥ 40 kg/m^2^. Differences in these criteria are mainly due to the higher incidence of obesity-related complications at BMI ≥ 25 kg/m^2^ in the Japanese population [503] and differences in body compositions among ethnic groups. Morbid obesity accounts for approximately 20% of severely obese patients in the U.S. and Europe [504, 505], but only 0.6% in Japan; however, this value is increasing [506]. Patients with BMI ≥ 30 kg/m^2^ may be at a lower risk of mortality than those with a BMI in the normal range or with severe obesity, a phenomenon known as the obesity paradox [504], there is no consistent research results have been obtained for critically ill patients [507]. In addition, morbidity and mortality rates are higher with sarcopenic obesity, a condition in which muscle strength and muscle mass are reduced and fat mass is maintained or increased, than with sarcopenia or obesity alone [508, 509].

Consensus on estimating the energy requirements of severely obese patients is lacking among the guidelines; nevertheless, based on limited evidence, the measurement of REE by IC is recommended [4, 20, 125]. When IC is not available, the Penn State equation, which was developed in a cohort of critically ill patients with a wide BMI range, and the Modified Penn State equation for patients older than 60 years have been shown to provide similar estimates to those by IC [510] (Table 13). Another method is to measure the energy target by multiplying a designated calculation factor for the corresponding BMI ranges with actual body weight, ideal body weight, or adjusted body weight. Actual body weight is described in ESPEN guideline 2023 [4] and JCCNG 2016 [20], while adjusted body weight is only described in ESPEN guideline 2023 [4] (Table 14). When ideal body weight is calculated at BMI 22 kg/m^2^, the estimated energy target will be the lowest when using ideal body weight, followed by adjusted body weight and actual body weight (see CQ. 3–2).

**Table 13:** Penn State and modified Penn State equation (Ref. [509])

Penn State equation	RMR (kcal/day) = MSJ (0.96) + Tmax (167) + VE (31)−6212
Modified Penn State equation	RMR (kcal/day) = MSJ (0.71) + Tmax (85) + VE (64)−3085
Male	5 + 10 × weight (kg) + 6.25 × height (cm)−5 × age (year) kcal/day
Female	− 161 + 10 × weight (kg) + 6.25 × height (cm)−5 × age (year) kcal/day

MSJ: Mifflin–St Jeor equation; VE: minute ventilation (l/min); Tmax: highest temperature in the past 24 h; RMR: resting metabolic rate

**Table 14:** Calculating ideal and adjusted body weights (Refs. [4, 67, 125])

Actual body weight	–
Ideal body weight: IBW	Japan: BMI 22 kg/m^2^
	Europe and America: BMI 25 kg/m^2^
	ESPEN:
	Male: 0.9 × (height [cm]−100)
	Female: 0.9 × (height [cm]−106)
Adjusted body weight	ESPEN:
	IBW + 0.2−0.25 (actual body weight−IBW)

Protein targets for severely obese patients may be calculated with the same method as the estimation of energy targets, using the estimation formula by multiplying either actual body weight, ideal body weight, or adjusted weight with the designated calculation factor for the corresponding BMI ranges. ESPEN guideline 2023 [4] recommends an evaluation by the nitrogen balance (for an evaluation of the nitrogen balance, see CQ. 3-3).

In severely obese patients, all guidelines recommend the calculation of energy and protein targets at a lower body weight than actual body weight, such as ideal or adjusted body weight [4, 20, 125]. Furthermore, the calculation of nutritional targets based on the actual body weight of these patients with a BMI of 30 kg/m^2^ or higher may lead to overfeeding; therefore, using ideal or adjusted body weight needs to be considered.

Being underweight is caused by the insufficient intake or absorption of nutrients, leading to a decrease in lean body mass and changes in body cell mass. This results in the deterioration of physical and mental functions, an increased risk of mortality, prolonged hospital stays, and increased hospitalization costs, thereby worsening clinical outcomes [1]. Being underweight is one of the criteria used to assess malnutrition.

Methods for estimating the energy and protein requirements of critically ill underweight patients are described in ASPEN guideline 2016 [125] and ESPEN guideline 2018 [67] as nutrition therapy for patients deemed severely underweight based on nutritional screening tools. The type of body weight used in the calculation of energy and protein targets is not specifically described; therefore, a practical recommendation is to use actual body weight for the calculation. Although evidence is limited on how to set energy targets for underweight patients, the use of REE measured by IC is recommended.

A nutritional assessment (see CQ. 3-1) and RFS-specific risk assessment (see CQ. 3-8) need to be performed for critically ill underweight patients; nutrition needs to be initiated as soon as possible when the patient is evaluated to be at risk of malnutrition, starting at 5–10 kcal/kg/day with progressive increases as needed to minimize the energy debt as recommended by the National Institute for Health and Clinical Excellence (NICE) in England and Wales criteria for patients in the high-risk category [511].

**CQ3-8: What is the concept of nutrition therapy for refeeding syndrome in critically ill patients?**

**Answer**: With a risk assessment specific to refeeding syndrome (RFS), energy restriction and electrolyte monitoring with correction are considered based on the risks of RFS, post-onset symptoms, and electrolyte abnormalities (provision of information for background question).

**Rationale**

RFS is one of the serious metabolic complications that may occur with nutritional administration. Although the pathogenesis of RFS remains unclear, the onset and worsening of the condition may be prevented with appropriate measures. It is important to address how these measures need to be implemented in these guidelines. In addition, a risk assessment is necessary for prevention, and risk factors, such as low BMI and electrolyte abnormalities, have been reported. However, the thresholds used for RFS risk factors vary widely, and in critically ill patients, the risk factor for developing RFS remain unclear. Therefore, a risk assessment and response for RFS are addressed in this CQ.

RFS is regarded as an electrolyte and metabolic abnormality associated with nutritional administration to patients with periods of insufficient intake or malnutrition. Critical illness is a highly invasive and hypercatabolic state that may result in RFS even without malnutrition [67]. During starvation, lipids and proteins are utilized as energy sources; however, the resumption of nutrition increases insulin secretion as the energy source switches to carbohydrates. Insulin shifts phosphorus, potassium, magnesium, and water into cells, resulting in hypophosphatemia, hypokalemia, and hypomagnesemia. Furthermore, phosphorus is consumed because of the rapid metabolic switch from catabolism to anabolism, the utilization of carbohydrates taken into cells, and the stimulation of ATP production. Therefore, the likelihood of hypophosphatemia developing increases, resulting in abnormal electrolyte and glucose metabolism, vitamin (particularly vitamin B_1_) and trace element deficiencies, and fluid overload. Through a series of mechanisms, the condition can range from mild symptoms to various severe manifestations such as edema, circulatory and respiratory failure, disorientation, coagulation abnormalities, and, in the most severe cases, death [512, 513].

To date, there are no internationally standardized diagnostic criteria for RFS. Most of the diagnostic criteria for RFS in critically ill patients used in individual studies are based on serum phosphorus concentrations. When serum phosphorus concentrations are used, a diagnosis is made when the serum phosphorus concentration decreases by more than 0.5 mg/dL and is < 2.0 mg/dL within 72 h of the start of nutrition [67, 513, 514]. Other diagnostic criteria include a combination of electrolyte abnormalities and organ damage attributable to vitamin B_1_ deficiency [515]. In addition, a diagnosis is made when the serum phosphorus concentration is ≤ 1.8 mg/dL or more than 30% below the reference value, and when two or more of the following criteria apply: (1) a serum phosphorus concentration ≤ 2.4 mg/Dl; (2) a serum magnesium concentration ≤ 1.8 mg/dL; and (3) a serum potassium concentration ≤ 3.4 mEq/L. When diagnostic criteria are met, the diagnosis is classified as either “imminent RFS” if only electrolyte abnormalities are present or “manifest RFS” if clinical symptoms appear in addition to electrolyte abnormalities [516] (Table 15).

**Table 15:** Diagnostic criteria for RFS in critically ill patients (Refs. [4, 67, 513–518])

Ref. [67, 513, 514]	Serum phosphorus concentration of any of the following within 72 h of initiating nutrition therapy	
	• ≤ 2.0 mg/dL	
	• Decrease of 0.5 mg/dL or more from the previously measured value	
ESPEN refeeding hypophosphatemia [4]	Serum phosphorus concentration after nutritional administration falls into one of the following categories	
	• ≤ 2.0 mg/dL	
	• Decrease of more than 0.5 mg/dL	
Evidence-based and consensus-supported algorithm [516]	• If the serum phosphorus concentration decreases by 30% or more from the baseline or falls to ≤ 1.8 mg/dL within 72 h of initiating nutrition therapy	
	• Or, if any two of 1) to 3) apply	
	1) Serum phosphorus concentration ≤ 2.4 mg/dL	
	2) Serum magnesium concentration ≤ 1.8 mg/dL	
	3) Serum potassium concentration ≤ 3.4 mEq/L	
	Diagnosis	
	• If electrolyte abnormalities are the only symptoms, then it is regarded as imminent RFS	
	• If clinical symptoms appear in addition to electrolyte abnormalities, it is regarded as manifest RFS	
ASPEN Consensus Recommendation for Refeeding Syndrome [515]	1) Serum phosphorus, potassium, and/or magnesium concentrations decrease by 10% or more	
	2) This decrease occurs within 5 days of the resumption of nutrition or an increase in administered energy	
	Additionally, the severity classification is as follows when the above criteria apply:	
	Mild	10–20% decrease
	Moderate	20–30% decrease
	Severe	• A decrease of 30% or more
		• And/or organ dysfunction resulting from a decrease in any of these electrolytes
		• And/or due to vitamin B_1_ (thiamin) deficiency

Risk assessments for RFS have primarily been developed for patients who are not critically ill, and NICE criteria were widely used worldwide [511, 517] (Table 16). The evidence-based and consensus-supported algorithm classifies risk into four categories based on the NICE criteria. In addition to Table 15 [516] and the NICE criteria, the ASPEN Consensus Recommendations for Refeeding Syndrome [515], which include additional criteria, such as the disease state, physical examination findings, including subcutaneous fat and muscle mass loss, and the Management of Really Sick Patients with Anorexia Nervosa (MARSIPAN), are discussed below [518] (Tables 17 and 18). A study that defined RFS as hypophosphatemia within 72 h of nutritional initiation, with serum phosphorus concentrations > 0.5 mg/dL and ≤ 2.0 mg/dL, found that 34% of critically ill patients had RFS. A subsequent study using the same definition reported an incidence of 8% in the at-risk population by the Simplified Nutrition Assessment Questionnaire [519]. Another prospective cohort study showed that the incidence of RFS was 2% among 243 individuals assessed as at risk by the NICE criteria, with severe electrolyte depletion (serum potassium, phosphorus, and magnesium concentrations), fluid overload, and organ dysfunction as the diagnostic criteria [520]. Since these are not uniform diagnostic criteria, the overall incidence of RFS has yet to be clarified. These criteria are not exhaustive, and the quantification of risk is not possible [515].

**Table 16:** Risk assessment of RFS (Refs. [511, 515, 516, 518])

**National Institute for Health and Care Excellence (NICE)** [511]		
Major risk factors		
1) BMI < 16 kg/m^2^		
2) Unintentional weight loss > 15% within the last 3–6 months		
3) Little or no nutritional intake for more than 10 days		
4) Low levels of potassium, phosphate, or magnesium prior to feeding		
Minor risk factors		
1) BMI < 18.5 kg/m^2^		
2) Unintentional weight loss > 10% within the last 3–6 months		
3) Little or no nutritional intake for more than 5 days		
4) A history of alcohol abuse or drugs, including insulin, chemotherapy, antacids, or diuretics		
**Evidence-based and consensus-supported algorithm** (Ref. [516])		
No risk	Neither a major nor minor risk factor is applicable	
Low risk	1 minor risk factor	
High risk	1 major or 2 minor risk factors	
Very high risk	One or more of the following apply:	
	• BMI < 14 kg/m^2^	
	• Weight loss > 20%	
	• Starvation > 15 days	
**ASPEN Consensus Recommendations for Refeeding Syndrome** (Ref. [515])		
	Moderate risk : 2 risk criteria needed	Significant risk : 1 risk criteria needed
BMI	16–18.5 kg/m^2^	< 16 kg/m^2^
Weight loss	> 5% in 1 month	• > 7.5% in 3 months • > 10% in 6 months
Caloric intake (satisfy any of the following)	• None or negligible oral intake for 5–6 days	• None or negligible oral intake for > 7 days
	• < 75% of the estimated energy requirement for > 7 days during an acute illness or injury	• < 50% of the estimated energy requirement for > 5 days during an acute illness or injury
	• < 75% of the estimated energy requirement for > 1 month	• < 50% of the estimated energy requirement for > 1 month
Abnormal prefeeding serum potassium, phosphorus, or magnesium concentrations (Fulfill either of the following)	• Minimally low levels or normal current levels and recent low levels necessitating minimal • Single-dose supplementation	• Moderately/significantly low levels or minimally low or normal levels and recent low levels necessitating significant • Multiple-dose supplementation
Loss of subcutaneous fat	Evidence of moderate loss	Evidence of severe loss
Loss of muscle mass	Evidence of mild or moderate loss	Evidence of severe loss
Higher risk comorbidities (see Table 17)	Moderate disease	Severe disease
**The Management of Really Sick Patients with Anorexia Nervosa** (Ref. [518])		
High risk	1) BMI < 13 kg/m^2^	
	2) Unintentional weight loss > 15% within the last 3 months	
	3) Little or no nutritional intake for more than 4 days	
	4) The following medical complications prior to the start of nutrition	
	• Electrolyte abnormalities	
	• Pneumonia or other serious infections	
	• Cardiac dysfunction or heart disease	
	• Hepatic disorder due to alcoholism

**Table 17:** Diseases and clinical conditions associated with an increased risk of refeeding syndrome (Ref. [515])

Acquired immunodeficiency syndrome
Chronic alcohol or drug use disorder
Dysphagia and esophageal dysmotility (e.g., eosinophilic esophagitis, achalasia, and gastric dysmotility)
Eating disorders (e.g., anorexia nervosa)
Food insecurity and homelessness
Failure to thrive, including physical and sexual abuse and victims of neglect (particularly children)
Hyperemesis gravidarum or protracted vomiting
Major stressors or surgery without nutrition for prolonged periods of time
Malabsorptive states (e.g., short bowel syndrome, Crohn’s disease, cystic fibrosis, pyloric stenosis, maldigestion, and pancreatic insufficiency)
Cancer
Advanced neurological impairment or a general inability to communicate needs
Post-bariatric surgery
Post-operative patients with complications
Prolonged fasting (e.g., individuals on hunger strikes or anorexia nervosa)
Refugees
Protein malnourishment

**Table 18:** Management and prevention of RFS (adapted from Ref. [516])

		No risk	Low risk	High risk	Very high risk
Preventive measures before/during nutrition therapy		–	200–300 mg of vitamin B_1_ for 5 days and a multivitamin for 10 days		
		Measuring electrolytes daily up to day 3, assessment of the hydration status			
Energy	Days 1–3	Unlimited	15–25 kcal/kg/day	10–15 kcal/kg/day	5–10 kcal/kg/day
	Day 4		30 kcal/kg/day	15–25 kcal/kg/day	10–20 kcal/kg/day
	Day 5		Unlimited		
	Day 6			30 kcal/kg/day	
	Days 7–9			Unlimited	20–30 kcal/kg/day
	> 10 days				Unlimited
Fluids		Unlimited	Fluids to maintain a zero balance, approx. 30–35 mL/kg/day	Fluids to maintain a zero balance, Days 1–3: 25–30 mL/kg/day > 4 days: 30–35 mL/kg/day	Fluids to maintain a zero balance, Days 1–3: 20–25 mL/kg/day Days 4–6: 25–30 mL/kg/day > 7 days: 30–35 mL/kg/day
Salt		Unlimited	Unlimited	Days 1–7: < 1 mmol/kg/day	Days 1–10: < 1 mmol/kg/day
Iron		No iron substitution within the first 7 days even if patients have iron deficiency			
Monitoring		• Assessment of serum electrolyte levels daily up to day 3, then every 2–3 days • Daily clinical examination focusing on the hydration status 1–2 times per day • Continuous monitoring of the cardiac rhythm or electrocardiogram daily in patients at a very high risk of RFS			

In a previous study on ICU patients who developed RFS within 72 h of the resumption of nutrition, a comparison of the long-term prognosis between the energy-restriction group and standard nutrition therapy group showed that survival was significantly longer in the former group [67]. In a retrospective cohort study on critically ill patients admitted to the ICU on ventilatory management, among those who developed RFS within 72 h of the resumption of nutrition, energy restriction up to the third day after admission was associated with 6-month survival [514]. Another study compared a group of nutritionally impaired elderly patients (medium to high risk by the NICE criteria) who started with low energy and slowly reached the goal with a group who started with high energy and quickly reached the goal, and found no significant differences in grip strength, the incidence of RFS, or mortality between the two groups, with dyspnea being more common in the latter [521].

Although there is consensus for monitoring electrolytes and providing supplementation of electrolyte as needed in patients at risk of developing RFS, the 2006 NICE criteria additionally recommend monitoring ECG and supplementation with vitamin B_1_, if the risk is extremely high (BMI < 14 kg/m^2^ or if the patient has been fasting for > 15 days) or if arrhythmia has already appeared [511]. The evidence-based and consensus-supported algorithm suggest measuring electrolytes daily up to day 3 and assessing the volume status in all patients, including those without risk factors [516] (Table 19).

**Table 19:** Guidelines from each country (Ref. [4, 20, 515])

JCCNG2016 (Ref. [20])	Serum potassium, phosphorus, and magnesium concentrations are measured in patients at risk of developing the disease
ESPEN guideline 2023 (Ref. [4])	• Serum potassium, phosphorus, and magnesium concentrations need to be measured at least once daily for the first week
	• If hypophosphatemia develops with the initiation of nutrition, regardless of symptoms, electrolytes need to be measured 2 to 3 times daily and supplemented as needed
	• If hypophosphatemia develops with the initiation of nutrition, regardless of symptoms, energy supply needs to be restricted for 48 h and then gradually increased
ASPEN Consensus Recommendations for Refeeding Syndrome (Ref. [515])	*At the start of nutrition*
	• The first 24 h need to begin with 100 to 150 g of glucose or 10 to 20 kcal/kg. This includes glucose administered enterally as well as parenterally
	• In patients with moderate to high-risk RFS who are electrolyte-deficient, consideration needs to be given to refraining from initiating or increasing nutritional doses until electrolytes are replenished and/or normalized
	• In patients with significantly low serum potassium, phosphorus, or magnesium concentrations, the initiation of or an increase in nutritional doses needs to be delayed until corrections are made
	*Water restriction*; not recommended
	*Sodium restriction*; not recommended
	*Protein restriction*; not recommended
	*Electrolytes*
	• Check serum potassium, phosphorus, and magnesium concentrations before starting nutritional administration
	• Monitor every 12 h for the first 3 days in high-risk patients. Increase the monitoring frequency depending on the disease status
	• Replenish electrolytes according to established standards of care
	• The prophylactic administration of electrolytes is not recommended when values are normal prior to nutritional initiation
	• If electrolytes are difficult to correct or decrease rapidly during the initiation of nutritional administration, based on pathophysiology, reduce energy/glucose intake by 50% and gradually increase them by approximately 33% of the target every one to two days. Recommendations are subject to change based on practitioner judgment and clinical presentation, and if electrolytes are severely and/or life-threateningly low or rapidly decreasing, the discontinuation of nutritional administration needs to be considered
	*Vitamin B*_*1*_* and multivitamins*
	• In at-risk patients, supplement with 100 mg of vitamin B_1_ prior to nutritional administration or before starting an infusion containing glucose
	• In patients at a high risk of severe starvation, chronic alcoholism, other deficiencies, and/or symptoms of vitamin B_1_ deficiency, supplement with 100 mg/day of vitamin B_1_ for 5 to 7 days or longer
	• The routine measurement of vitamin B_1_ is not meaningful
	• Multivitamins need to be added to PN daily as long as PN is continued without contraindications. In patients initiating oral/EN, add a comprehensive oral/enteral multivitamin once daily for at least 10 days based on the clinical status and therapy
	*Monitoring and long-term treatment*
	• In at-risk patients, the assessment of vital signs every 4 h for the first 24 h of nutritional administration is recommended • Cardiopulmonary monitoring based on established standards of care is recommended for unstable patients or those with severe heart failure
	• Assess intake and elimination and weigh the patient daily
	• Assess the short and long-term goals of nutrition therapy daily for the first few days until the patient is deemed stable (no need for electrolyte supplementation for 2 days), followed by the institutional standards of care

On the other hand, a systematic review and meta-analysis comparing energy doses for undernourished patients at risk of developing RFS included 4 RCTs and 14 observational studies and reported a shorter hospital stay (3.0 days) in the higher energy group (800–1400 kcal/day or 15–20 kcal/kg/day) than in the high energy group (1400–1800 kcal/day or 30 kcal/kg/day) despite high heterogeneity between the studies [522].

As described above, the recommended energy dose for patients at risk of developing RFS has been increasing in recent years; nevertheless, the policy of energy restriction for high-risk patients has not changed. In critically ill patients, the findings of large RCTs and observational studies suggest that energy doses need to be restricted in the presence of RFS or hypophosphatemia, even in the absence of obvious nutritional impairment [511].

The previous version of current guidelines (JCCNG 2016) [20] only describes the risk of RFS. ESPEN guideline 2023 [4] discussed the frequency of electrolyte measurements and how to intervene in patients who develop hypophosphatemia after nutritional administration. ASPEN guideline 2016 [125] recommends energy restriction, while its revised version, ASPEN guideline 2022 [5], does not discuss RFS (Tables 18 and 19).

While the 2006 NICE criteria were widely used [511], MARSIPAN developed by the British Psychiatric Association after a review of deaths from underfeeding due to compliance with these guidelines [518]. When the NICE guidelines were revised in 2017, they no longer specifically mentioned RFS measurements and recommended referring to the MARSIPAN resource for nutritional management [523]. Nutritional management for RFS are also described in the evidence-based and consensus-supported algorithm [516] and ASPEN Consensus Recommendations for Refeeding Syndrome [515].

**CQ3-9: What is the approach to nutrition therapy for critically ill patients undergoing special treatments such as extracorporeal membrane oxygenation (ECMO), prone position (PP), and open abdominal management (OAM)?**

**Answer**: For critically ill patients undergoing special treatments such as ECMO, PP, and OAM, appropriate nutrition therapy, including early enteral nutrition, will be provided based on the pathophysiology, disease progression, and gastrointestinal tract status (provision of information for background question).

**Rationale**

When providing special treatments to critically ill patients, such as ECMO, PP, and OAM, difficulties are associated with their combination with nutrition therapy, particularly EN.

ECMO is divided into veno-venous ECMO (VV-ECMO), which is used for severe respiratory failure, and veno-arterial ECMO (VA-ECMO), which is used for acute circulatory failure. All of these patients are in a critical condition and exhibit a number of symptoms, such as protein catabolism and insulin resistance. In addition, the administration of EN may be difficult due to the risk of vomiting and aspiration associated with reduced gastrointestinal motility and delayed gastric emptying as a result of invasion and sedation [524]. A prolonged ICU stay may lead to inadequate nutrition and iatrogenic malnutrition; however, this type of nutritional disorder is often overlooked [525]. VA-ECMO is associated with a risk of reduced organ blood flow and intestinal ischemia because vasopressors are administered to treat unstable hemodynamics [526]. Since there are no nutritional assessment tools specifically for ECMO patients, nutritional assessments are performed in the same manner as for other critically ill patients. The ASPEN guidance [525] recommends that all ECMO patients be considered at nutritional risk.

Regarding energy dosages, the German Society for Nutritional Medicine (DGEM) guidelines [527] recommend using a 25 kcal/kg/day formula for ECMO patients. IC estimates EE from CO_2_ production and O_2_ consumption and, thus, is not applicable to ECMO patients without special techniques [528, 529]. ECMO and blood purification therapy are associated with the loss of amino acids, particularly alanine, arginine, cysteine, glutamine, and isoleucine [530]. ECMO patients may require more protein than is generally recommended for critically ill patients [531]; however, due to a lack of evidence, protein dosages will be set according to those for other critically ill patients.

Even in ECMO patients, the target nutritional intake may be achieved with proper management. Lukas et al. reported that the mean energy sufficiency rate during ECMO was 62%, which was significantly higher in VV-ECMO than in VA-ECMO (67% vs. 50%) [532]. Scott et al. showed that the mean energy intake during ECMO was 80% of the target [533]. Furthermore, in an observational study on VA-/VV-ECMO patients, Ferrie et al. found that 79.7% of the target energy intake and 73.0% of the target protein intake were administered within the first 2 weeks [534]. The goal was achieved more quickly with VV-ECMO than with VA-ECMO, which was attributed to less circulatory instability with VV-ECMO.

Regarding the route of nutritional administration in ECMO patients, the DGEM guidelines [527] recommend that ECMO patients be provided with EN at any stage of the disease unless they have severe gastrointestinal or circulatory failure. Concerning the timing of nutrition therapy for patients, the ESPEN guidelines [67] recommend early EN within 24–48 h of initiating ECMO or the onset of critical illness.

During ECMO, particularly VA-ECMO, there is a risk of intestinal necrosis, such as non-occlusive mesenteric ischemia, when high doses of vasopressors are administered. However, severe intestinal dysfunction and circulatory failure rarely occur in clinical settings, and a review by Davis et al. showed that the incidence of intestinal ischemia was 0.7% [535]. In addition, Lu et al. demonstrated that in ECMO patients, achieving 80% of the target energy intake within 7 days was associated with a good prognosis, and EN may be started early even during ECMO with good gastrointestinal tolerance [536]. Since early EN has been associated with reduced in-hospital and 28-day mortality rates [537], it is considered safe and effective for ECMO patients and is recommended in the ASPEN guidance [525]. Karpasiti’s review also showed that early EN was safe and feasible for ECMO patients [538].

The ASPEN guidance [525] states that there is no need to hesitate to administer PN to ECMO patients if they have hyperbilirubinemia due to hypoperfusion or acute hepatic ischemia. If EN is not available to ECMO patients, PN is administered.

The DGEM guidelines [527] recommend the administration of lipid emulsions to ECMO patients when providing EN. Since previous studies demonstrated that triglycerides (TG) precipitate in the ECMO artificial lung at TG levels ≥ 1000 mg/dL [539, 540], adjustments to lipid emulsions and propofol dosages are recommended at TG levels ≥ 400 mg/dL [541].

PP is one of the treatments for severe respiratory failure. Recent studies showed that prolonged PP was effective [542, 543]. In PP, gastrointestinal movement is restricted due to the flat body position, increased abdominal pressure due to abdominal compression, and the use of high doses of sedatives and opioids, which may increase the risk of vomiting and aspiration. If EN is discontinued in PP and administered only in the supine position due to this concern, the amount administered will be limited due to the prolonged duration of PP.

The ESPEN guidelines [67] recommend no delay in enteral feeding due to PP. The DGEM guidelines [527] also recommend the administration of nutrition via the stomach or small intestines during PP if the gastrointestinal tract is functional, which is based on the findings of three studies showing no significant difference in GRV or other clinical outcomes between the prone and supine positions [544, 545], and one study that found significant increases in GRV, the frequency of vomiting, and the interruption of EN in PP, but not in pneumonia or mortality [546].

Regarding the amount of EN administered, Reignier et al. reported that it was significantly lower in PP than in the supine position over a 5-day observation period [546]. However, Savio et al. found no significant difference in the amounts of energy and protein administered in the supine position and PP (24.5 kcal/kg/day and 1.1 g/kg/day vs. 23.5 kcal/kg/day and 1.1 g/kg/day) [547]. Savio et al. also showed that enteral feeding via a nasogastric/orogastric tube during PP was feasible and well tolerated, with energy and protein intakes equivalent to that in the supine position [547]. Regarding the administration method of EN in PP, Reignier et al. [548] reported in a before–after study that the introduction of a protocol during PP, which consisted of gradually increasing the rate of nutrition administration, elevating the head by 25°, and administering erythromycin, significantly increased the amount of nutrition administered and did not result in an increase in GRV or in vomiting or VAP. Although EN may be administered during PP, due to the high risk of complications, such as vomiting, it may be necessary to carefully increase the dosage and pay close attention to the development of gastrointestinal symptoms.

OAM may be performed during emergency open surgery for trauma, abdominal compartment syndrome, or intra-abdominal infection. During OAM, patients are in a hypermetabolic state. McKibbin et al. investigated the effects of nutrition therapy after abdominal trauma, and found that the basal metabolic rate in patients with OAM increased by 40% [549]. Furthermore, abdominal effusion associated with OAM may result in nitrogen losses of 3.5 g per day or 1.9 g per liter [550]. Therefore, nutrition therapy based on the nitrogen balance must be considered. The World Society for Emergency Surgery (WSES) guidelines [551] recommend the administration of 1.5–2.5 g/kg/day of protein and 20–30 kcal/kg/day of non-protein calories to maintain a positive nitrogen balance.

The ESPEN guidelines [67] and DGEM guidelines [527] recommend early EN for patients during OAM. The WSES guidelines [551] also recommend early EN, even in cases of OAM after abdominal damage control surgery, the maintenance of gastrointestinal continuity, the absence of functional issues in the gastrointestinal tract, such as obstruction, and the achievement of appropriate fluid resuscitation and metabolic corrections. Early EN in patients during OAM is not associated with a risk of delayed wound healing or the formation of enterocutaneous fistulas [552], and has been reported to achieve favorable outcomes, such as increased wound closure rates and a reduction in the formation of enterocutaneous fistulas [553]. Furthermore, the incidence of VAP was lower in an early EN group than in a group not receiving EN [554]. Early enteral feeding during OAM has also been initiated with 15 mL/h feeding for the first 4 days followed by an increase to the target rate [555]. Based on these findings, early EN is possible even during OAM.

However, the WSES guidelines recommend a delay in enteral feeding in patients with an intestinal tract in discontinuity, in patients with a high output fistula with no possibility of feeding access distal to the fistula, and in patients with signs of intestinal obstruction [551]. In addition, a review by Bansal et al. [552] recommended considering total PN if gastrointestinal issues did not improve within 48 h. Additionally, since nitrogen loss significantly increases in patients with an enterocutaneous fistula, it is recommended that patients be provided with PN as soon as possible and that EN be started when feasible; however, EN is thought to be relatively contraindicated in patients with short bowel syndrome in whom the remaining intestine is less than 75 cm [551, 556].

4. **Nutrition therapy in pediatrics**

**CQ4-1: Should nutritional assessment be performed in critically ill pediatric patients?**

**Answer**: Nutritional assessment should be performed in critically ill pediatric patients (Good Practice Statement).

**Rationale**

When providing nutrition therapy to critically ill pediatric patients, it is important to evaluate the nutritional status and identify nutritional disorders by appropriate nutritional screening and assessments. Malnutrition may lead to the development of complications and prolong ICU and hospital stays; therefore, accurate assessments and prompt interventions are important. It has not yet been established whether the use of nutritional screening and assessment tools in critically ill pediatric patients directly improves their outcomes.

Hospitalized pediatric patients are more susceptible to nutritional disorders, particularly those with underlying diseases who are at a high risk of nutritional disorders [557, 558]. In a prospective study on 296 pediatric patients admitted to a French tertiary hospital, weight loss occurred in 65% of patients during their hospital stay [559]. Malnutrition is associated with poor outcomes in both pediatric and adult patients, including longer hospital stays, increased susceptibility to infection, delayed wound healing, and increased hospital costs [560, 561]. A previous study reported that 10.2% of patients admitted to the ICU for at least 5 days had a BMI z-score reduction of at least 1 SD, while 28.8% of patients had a BMI reduction of at least 0.5 SD [562]. Malnutrition has also been associated with prolonged mechanical ventilation in pediatric patients ≥ 2 years old admitted to the ICU [563], and poor postoperative weight gain in patients undergoing surgery for congenital heart disease strongly correlated with late mortality [564]. In addition, since childhood is a period of marked changes in physical and mental characteristics from the neonatal period to school age, growth and development need to be taken account in nutritional management. Nutrition is necessary not only for physical growth, but also for mental health development. Previous studies reported that acute and chronic nutritional disorders were associated with impaired cognitive development in school children [565, 566].

The confirmation of physical findings, the presence of gastrointestinal symptoms, and previous medical history are generally the basis for nutritional assessment indicators. In children, height and weight are the most familiar and important indicators of physical findings for nutritional management, and it is important to confirm whether physical development is age-appropriate using comparisons with a standard growth curve [567]. The standard growth curve based on the 2010 survey is shown in Japan’s Maternal and Child Health Handbook [568]. In addition, after infancy, nutritional disorders may be identified based on height-for-age or weight-for-height. Height-for-age is the ratio of a patient’s height to the standard height of a child of the same age and is used to detect chronic malnutrition, while weight-for-height is the ratio of a patient’s weight to the standard weight of a child of the same height and is used to identify acute malnutrition [569]. One approach is to classify nutritional disorders according to height-for-age and weight-for-height proposed by Waterlow in 1972 [570]. However, in critically ill pediatric patients admitted to the ICU, body weight fluctuates widely due to changes in the fluid balance and, thus, needs to be interpreted with caution. The SCCM-ASPEN nutrition guidelines for critically ill pediatric patients recommend an assessment of the BMI z-score on admission to the ICU (weight-for-height if < 2 years old, weight-for-age if height cannot be accurately measured, and measurement of the head circumference if < 3 years old) [571]. The European Society of Pediatric and Neonatal Intensive Care (ESPNIC) nutritional guidelines for critically ill pediatric patients recommend assessments of weight, height, upper arm circumference, and head circumference using z-scores [572]. In addition, physical findings, including emaciation, obesity, dehydration, edema, skin findings, and the presence of pressure ulcers, are important. Regarding gastrointestinal symptoms, vomiting and diarrhea need to be noted. Previous and present medical histories and dietary intake also need to be reviewed. Several pediatric nutritional assessment tools have been developed, such as the Simple Pediatric Nutrition Risk Score, United States, 2000 [559], Subjective Global Nutritional Assessment, Canada, 2007 [573], Screening Tool for the Assessment of Malnutrition in Paediatrics (STAMP, United Kingdom, 2008) [574], Paediatric Yorkhill Malnutrition Score (PYMS, United Kingdom, 2010) [575], and STRONGkids (Netherlands, 2010) [576]. However, in children, there are no nutritional assessment tools specific to critically ill patients, such as the NUTRIC score for adult patients. Furthermore, none of these assessment tools have been widely adopted. A systematic review of nutritional screening tools in pediatric inpatients ultimately reviewed 49 articles and reported that 3 tools, STRONGkids, STAMP, and PYMS, were the most commonly used, with STRONGkids being the most accurate at diagnosing malnutrition and predicting poor outcomes [577]. A systematic review of nutritional assessments for critically ill pediatric patients has also been conducted; however, only 6 of the 103 articles reviewed were limited to ICU patients. Therefore, none of the nutritional screening tools have been validated for critically ill patients [578]. Although albumin, prealbumin, transferrin, and retinol-binding protein may be used as indicators of the nutritional status based on blood test results, they cannot simply be used for critically ill patients because factors other than malnutrition may decrease or result in the loss of synthetic capacity [579, 580]. Recent studies investigated muscle mass changes (thicknesses of the quadriceps femoris and diaphragm muscles) over time in critically ill pediatric patients using ultrasound [581, 582].

**CQ4-2: What is the strategy for energy intake in the acute phase of treatment of critically ill pediatric patients?**

**Answer**: In the acute phase of treatment of critically ill pediatric patients, there is a strategy that target energy intake is set at approximately 60% to 70% of the energy expenditure or does not exceed the energy expenditure (provision of information for background question).

**Rationale**

In the early stages of treatment for critically ill adult patients, the impact of energy administration ≥ EE on clinical outcomes remains unclear. In addition, research and knowledge in the pediatric field are still limited, and the effects of low energy administration less than resting EE on clinical outcomes and subsequent growth and development have yet to be elucidated. However, setting the target energy intake is essential in nutritional management for critically ill pediatric patients, and it is important to examine the findings obtained to date, even those from non-randomized studies.

Under severe conditions or in invasive stages, stress hormones and cytokines induce protein catabolism and gluconeogenesis, and as a result, the corresponding endogenous energy supply is provided. If the administration of energy intake is equal to or greater than resting EE, there will be an excess of energy, and various overfeeding adverse effects, including hyperglycemia, will occur. However, until the early 2000s, EE itself was regarded as the amount of energy required [583]. Until the 2010s, limited research was conducted on the amount of energy that needs to be administered and there were no large-scale observational studies. The 2009 ASPEN guidelines for nutritional administration in critically ill pediatric patients [584] made recommendations regarding the amount of energy consumed, but did not discuss energy requirements, and there was no expert opinion on the recommended target energy intake in critically ill pediatric patients.

In 2012, Mehta et al. performed a multicenter prospective observational study (*n* = 500) [585] to investigate the nutritional administration content (amount of energy administered, protein amount, EN, and the presence or absence of PN) for 10 days after the admission to pediatric ICUs (31 facilities in 8 countries) of children aged 1 month to 18 years who required mechanical ventilation for more than 48 h. They also examined the relationship between the clinical course (duration of mechanical ventilation, length of ICU stay, and 60-day survival rate). The findings obtained showed that patients who received 33.3% or more of their target energy intake through EN had a lower mortality rate than those who received less than 33.3%, and those who were able to receive 66.7% or more of their target energy intake had an even lower mortality rate. On the other hand, the use of PN was associated with an increase in mortality (the dosage was not indicated). The 2017ASPEN guidelines [586] state that “we suggest achieving delivery of at least two-thirds of the prescribed daily energy requirement by the end of the first week in the PICU.”, while the ESPNIC guidelines state that “In the acute phase, energy intake provided to critically ill pediatric patients should not exceed resting energy expenditure.”, according to the Clinical Recommendations (2020) [572]. The United States and Europe both cite the study by Mehta et al. [585], and recommend that “the target energy intake in the acute phase should not exceed energy consumption indicated by the REE.” The REE for pediatric patients mentioned in this document is mainly calculated based on the Schofield equation [587] and the WHO equation [588]. These estimation formulae [Schofield’s equation (Table 20) and the WHO’s formula (Table 21)] are shown for reference. In addition, similar to many cases in clinical practice, the energy requirement per body weight is set as the target energy intake, and the target energy per body weight is shown, which is partially modified from the 2018 ESPEN guidelines (Table 22) (parenteral nutrition) [589]. Although the amount of energy indicated was the expert opinion, it is easy to use in clinical settings.

**Table 20:** Schofield’s formula [587] (kcal/day)

0–3 years	Male	REE = 59.5 × weight (kg) -30.4
	Female	REE = 58.3 × weight (kg) -31.1
3–10 years	Male	REE = 22.7 × weight (kg) + 504
	Female	REE = 20.3 × weight (kg) + 486
10–18 years	Male	REE = 17.7 × weight (kg) + 658
	Female	REE = 13.4 × weight (kg) + 693

1 cal = 4.184 J

**Table 21:** WHO formula [588] (kcal/day)

0–3 years	Male	REE = 60.9 × weight (kg) − 54
	Female	REE = 61.0 × weight (kg) − 51
3–10 years	Male	REE = 22.7 × weight (kg) + 495
	Female	REE = 22.5 × weight (kg) + 499
10–18 years	Male	REE = 17.5 × weight (kg) + 651
	Female	REE = 12.2 × weight (kg) + 746

**Table 22:** Energy requirements per body weight (parenteral nutrition) (kcal/kg/day)

	Acute phase (ICU)	Stable phase (ICU)	Recovery phase
Preterm	45–55	–	90–120
0–1 years	45–50	60–65	75–85
1–7 years	40–45	55–60	65–75
7–12 years	30–40	40–55	55–65
12–18 years	20–30	25–40	30–55

Table modified from reference [589]

The largest prospective observational study conducted in recent years was by Bechard and co-authors in 2021 (77 pediatric ICUs in 17 countries) [590]. They examined 1,844 children between the ages of 1 month and 18 years who required mechanical ventilation for 48 h from the time of admission and stayed in the ICU for more than 3 days. Energy and protein doses for 10 days after admission with EN and PN were examined, respectively, and a relationship was observed between nutritional intake and clinical outcomes as the 60-day mortality rate (primary outcome) as well as the onset of new infectious diseases and ventilator-free days (secondary outcomes). The target energy intake was set as estimated resting EE, which was derived from the Schofield equation in 752 patients (41%) and the WHO equation in 464 patients (25%). The target energy intake was set in only 8 cases (0.4%) based on the values measured by IC. Following adjustments for facility-by-institution, age, the admission status (emergency, planned), sex, a nutritional assessment by BMI, severity, complications, and the length of ICU stay, the findings obtained showed that patients who were able to receive more than 60% of their target energy intake up to 3 days or 4–7 days after admission had a lower 60-day mortality rate than those with lower energy levels (in both patient groups, regardless of whether energy administration was achieved by EN or supplemented with PN, similar outcomes were observed).

RCTs on this subject are expected in the future; however, until then, the findings of these observational studies suggest that 60–70% of EE (estimated or measured) by the first week is still the target energy intake.

Estimation formulae [Schofield’s equation (Table 20) and the WHO’s formula (Table 21)] for calculating the amount of resting energy consumed, which are mainly used in children, are shown. In addition, similar to many cases in clinical practice, the energy requirement per body weight is set as the target energy intake, and the target energy per body weight is shown, which is partially modified from the 2018 ESPEN guidelines (Table 22) (parenteral nutrition) [589]. Although the amount of energy indicated was the expert opinion, it is easy to use in clinical settings.

**CQ4-3: Should higher than standard protein doses (> 2.0 g/kg/day) be given to critically ill pediatric patients?**

**Answer**: We suggest against giving higher than standard protein doses (> 2.0 g/kg/day) to critically ill pediatric patients (GRADE 2D: certainty of evidence = “very low”).

**Rationale**

Protein dosage recommendations for children vary by age. The WHO recommended dose is 1.8 g/kg/day at 1 month old, 1.1 g/kg/day at 1 year old, 0.9 g/kg/day at 5 years old, and 0.9 g/kg/day at 10 years old [591]. The dietary reference intakes for Japanese are 10 g/day for 0 to 5 months old, 20 g/day for 1 to 2 years old, 25 g/day for 3 to 5 years old, and 45 g/day for boys and 50 g/day for girls at 10 to 11 years old [592]. The SCCM-ASPEN guidelines [586] and clinical recommendations from ESPNIC [572] recommend a minimum protein dose of 1.5 g/kg/day for critically ill pediatric patients. The standard protein dose of 1.0–2.0 g/kg/day is widely used for critically ill pediatric patients admitted to the PICU in Japan. It has yet to be clarified whether higher-than-normal protein doses improve the outcomes of critically ill pediatric patients. Children also need protein for growth, and higher-than-normal protein doses have been examined.

A meta-analysis was performed using 4 randomized control trials (RCTs) (Additional file 4) [593–596]. The results of the favorable outcomes were as follows: all-cause mortality yielded a RD of 16 fewer per 1000 (95% CI 97 fewer to 161 more) (2 RCTs, *n* = 121), length of mechanical ventilation yielded a mean difference (MD) of 1.3 days shorter (95% CI 4.0 shorter to 1.4 longer) (2 RCTs, *n* = 118). Therefore, the favorable outcomes were judged as small. The results of the unfavorable outcomes were as follows: length of ICU stay yielded a MD of 1.0 days longer (95% CI 1.9 shorter to 3.8 longer) (2 RCTs, *n* = 118), diarrhea yielded a RD of 66 more per 1000 (95% CI 71 fewer to 383 more) (2RCTs, *n* = 66), infectious complications yielded a RD of 8 more per 1000 (95% CI 83 fewer to 278 more) (2 RCTs, *n* = 66), the muscle mass change (upper arm circumference) yielded a MD of 0.2 cm shorter (95% CI 1.2 shorter to 0.8 longer) (1 RCT, *n* = 38), and hyperproteinemia did not occur in both groups (1 RCT, *n* = 41). Therefore, the unfavorable outcomes were judged as small. Based on the overall balance of effects, we thought that neither giving higher than standard protein doses nor not giving higher than standard protein doses was superior to the other.

As already discussed, protein dosing recommendations for children vary by age. Since the median and mean ages of patients included in the 4 RCTs reviewed in this CQ were 12 months or younger and older pediatric patients were not included, caution is needed with the interpretation of the findings obtained. Based on the balance of these effects, we concluded that an intervention with a higher dose of protein (> 2.0 g/kg/day) was likely inferior.

**CQ4-4: Should enteral nutrition be initiated within 48 h of starting treatment for critically ill pediatric patients?**

**Answer**: We suggest initiating enteral feeding within 48 h of starting treatment for critically ill pediatric patients (Grade 2D, certainty of evidence = “very low”).

**Rationale**

In the treatment of severe conditions in childhood, the initiation of EN is expected not only to improve the energy balance, but also to exert protective effects on the intestinal mucosa and thereby maintain the immune system; however, there is currently no established timing for its initiation. Although the initiation of EN as early as possible is generally considered advantageous, the risk of gastrointestinal complications, such as diarrhea, constipation, and necrotizing enterocolitis, as well as pneumonia due to aspiration has been noted. In the SCCM-ASPEN guidelines, “Early initiation of EN, within the first 24–48 h after admission to the PICU” was recommended for the CQ “When should EN be initiated?” (GRADE recommendation: weak, Quality of evidence: low) [586]. In the ESPNIC guidelines, “To commence early enteral nutrition within 24 h of admission unless contraindicated” was recommended for the CQ “In critically ill pediatric patients, when should enteral nutrition be commenced and how should it be increased?” (SIGN recommendation grade D, strong consensus) [572]. Given this balance between efficacy and safety and previous studies considering 48 h or less to be early, an important clinical issue is whether to start treatment for critically ill pediatric patients as early as 48 h or later.

One RCT was used for a meta-analysis (Additional file 4) [597]. The result of the favorable outcome was as follows: as length of ICU stay yielded a MD of 2.1 days shorter (95% CI 4.1 shorter to 0.1 shorter) (1 RCT, *n* = 30). Therefore, the favorable outcome was judged to be small. An unfavorable outcome was not observed in either group for all adverse events (necrotizing enterocolitis, vomiting, and diarrhea). Based on the overall balance of effects, the initiation of enteral feeding within 48 h of starting treatment was likely superior.

Enteral formulas and artificial milk are generally inexpensive, and the initiation of EN within 48 h may reduce costs because it avoids or delays the start of intravenous nutrition more than its initiation after 48 h. In addition, this intervention may be acceptable from the individual patient/family perspective, is feasible in any hospital, and requires minimal effort on the part of the healthcare provider. Based on the balance of these effects, we concluded that the intervention of initiating EN within 48 h was likely superior.

**CQ4-5: Should parenteral nutrition be initiated within 48 h of starting treatment for critically ill pediatric patients? (FRQ)**

**Rationale**

The findings from RCTs related to this CQ are inconsistent; therefore, difficulties are associated with providing useful recommendations based on these RCTs. On the other hand, it is possible to conduct RCTs on the timing of the start of PN in critically ill pediatric patients, and research on this question is expected in the future. Therefore, we decided that this CQ is a FRQ and only provided information.

Even under conditions where EN cannot be adequately administered, it is possible to start PN early after admission to the ICU in order to supplement the necessary nutrients. On the other hand, PN in the acute phase may result in an increased risk of infection and poor glycemic management, which may worsen the clinical outcomes of patients. Therefore, in view of the impact on clinical outcomes, it is important to consider the timing of PN administration in the nutritional management of critically ill pediatric patients.

Observational studies showed that malnutrition worsened clinical outcomes (mortality and the duration of mechanical ventilation) in critically ill pediatric patients [585, 598]. PN has been used when EN cannot meet energy requirements. The PEPaNIC trial [599], a multicenter RCT published in 2016, compared patients who started PN within 24 h of admission to the PICU (Early PN group: *n* = 723) with those who did not start PN until after 8 days of admission (Late PN group: *n* = 717). The findings obtained showed that the length of the ICU stay and the duration of mechanical ventilation were both significantly shorter in the Late PN group.

In a subsequent cross-sectional survey (81 PICUs in 39 countries, including Japan) conducted by van Puffelen et al. [600], there were 43 PICUs (53%) that were aware of the findings of the PEPaNIC trial, among which 10 (12%) responded that they would not administer PN to admitted children until one week after admission, whereas 17 (21%) responded that they would not administer PN with a few exceptions.

On the other hand, Mehta indicated that the actual administered energy in the Early PN group led to overfeeding in critically ill pediatric patients, it is important to be cautious when directly accepting the findings of the PEPaNIC trial [601].

One of the most frequent issues raised in the PEPaNIC trial is that PN was started on day 1 or after day 7 and it did not evaluate PN initiation from ICU day 2 to 6, during which PN is often started in actual clinical studies [587, 600]. Bechard et al. conducted a prospective study on nutritional administration in 77 PICUs (*n* = 1844) in 17 countries three years after the PEPaNIC trial and reported that the median start of PN was on the third day of admission [587]. In addition, a cross-sectional survey [600] demonstrated that the most common timing for the initiation of amino acid administration was on the 2nd to 4th day of admission. Therefore, the lack of consideration of the most common timing of the start of PN, which is typically practiced in the PICU, is an issue that needs to be resolved in the future.

Since the PEPaNIC trial, a number of studies have focused on the timing of the initiation of PN and important outcomes for ICU management (mortality, length of ICU stay, and duration of mechanical ventilation) in critically ill pediatric patients. Goday et al. conducted a multicenter retrospective observational study (*n* = 2069) and reported significantly higher mortality rates in the early PN group [602]. On the other hand, a prospective observational study by Ariagno et al. (*n* = 95) showed that the decrease in weight-for-age z-score was significantly less in the Early PN group [603] (weight loss was significantly less).

In 2023, Saleh et al. conducted a single-center, randomized controlled trial [604]. In children (1 month to 16 years old) who stayed in the ICU for more than 3 days when adequate EN was judged to be difficult, the duration of mechanical ventilation, length of ICU stay, and incidence of new infections were compared between the early PN group (initiation of PN within 24 h: *n* = 71) and the late PN group (initiation of PN on day 4 or 7: *n* = 69). The findings obtained, namely, a significant reduction in the duration of mechanical ventilation, a decrease in the length of ICU stay, and a decrease in the incidence of new infections, were in favor of the early initiation of PN. However, the external validity of this study needs to be considered, such as the findings obtained being based on a group of diseases that are indicated for early EN (respiratory and neurological diseases accounted for more than 60% of the total), and both groups were from a single institution that showed a higher actual mortality rate than the predicted mortality score (PRISM score).

Since the PEPaNIC trial, a number of studies have examined the effects of the timing of the initiation of PN after ICU admission on intensive care clinical outcomes (mortality, length of ICU stay, and duration of mechanical ventilation); however, discrepancies were observed in the findings obtained. Therefore, clinical studies in this field are awaited.

**CQ4-6: Should post-pyloric feeding rather than gastric feeding be started for enteral nutrition of critically ill pediatric patients?**

**Answer**: We suggest against using post-pyloric feeding rather than gastric feeding for enteral nutrition of critically ill pediatric patients (GRADE 2D: certainty of evidence = “very low”).

**Rationale**

Hypoperistalsis is a common complication in critically ill pediatric patients. Gastric feeding may not provide an adequate dose or may cause pneumonia due to increased vomiting and subclinical aspiration. Although post-pyloric feeding is expected to improve these issues, there are concerns about the inadequate absorption of some nutrients (vitamin B12 and trace elements), complications related to the technique of tube placement in the duodenum, and delays in the initiation of EN due to procedural difficulties. In the SCCM-ASPEN guidelines [586], data are insufficient to make recommendations regarding the optimal site to deliver EN to critically ill pediatric patients. In the ESPNIC clinical recommendations [572], gastric feeding was shown to be as safe as post-pyloric feeding in the majority of critically ill pediatric patients, and post-pyloric feeding may be considered for critically ill pediatric patients at a high risk of aspiration or requiring frequent fasting for surgery or procedures. Since the superiority of post-pyloric feeding over gastric feeding has not yet been established, it is an important clinical issue to clarify the benefits and harms of administering EN from the post-pyloric route.

A meta-analysis was performed using 4 randomized controlled trials (RCTs) (Additional file 4) [605–608]. The results of the favorable outcomes were as follows: ventilator-associated pneumonia yielded an RD of 100 fewer per 1000 (95% CI 180 fewer to 286 more) (1 RCT: *n* = 40) and vomiting yielded an RD of 145 fewer per 1000 (95% CI 283 fewer to 723 more) (2 RCTs: *n* = 102). Therefore, the favorable outcomes were judged as small. The results of the unfavorable outcomes were as follows: mortality yielded a RD of 62 more per 1000 (95% CI 33 fewer to 362 more) (2 RCTs: *n* = 102), duration of mechanical ventilation yielded a mean deference (MD) of was 6 days longer (95% CI 0.4 days shorter to 12.4 days longer) (2 RCTs: *n* = 102), length of hospital stay yielded a MD of 2.9 days longer (95% CI 5.5 days shorter to 11.3 days longer) (3 RCTs: *n* = 142), enteral nutrition initiation time yielded a RD of 18 h longer (95% CI 15.3 h shorter to 20.7 h longer) (1 RCT: *n* = 44), and aspiration yielded was a RD of 242 more per 1000 (95% CI 219 fewer to 1000 more) (2 RCTs: *n* = 106). Therefore, the unfavorable outcomes were judged as small. Based on the overall balance of effects, we thought that neither performing post-pyloric feeding nor not performing post-pyloric feeding was superior to the other.

Feeding tubes are generally inexpensive, but require manpower for post-pyloric placement, which is time consuming and sometimes requires fluoroscopy, resulting in X-ray exposure. In children, the post-pyloric placement of tubes is generally performed at the bedside without the use of an endoscope or other devices, and is easier and more feasible than in adults. However, hospitals with limited experience in treating critically ill pediatric patients may not have practitioners with sufficient technical experience in the post-pyloric placement of tubes and, thus, may be reluctant to perform the procedure.

The studies reviewed were comparisons of EN delivery as first-line-therapy, not comparisons of cases unable to tolerate gastric feeding. The effectiveness of post-pyloric feeding in cases of gastroesophageal reflux and delayed gastric emptying is not known. Based on the balance of these effects, we concluded that the intervention of performing post-pyloric feeding was likely inferior.

**CQ4-7: Should bolus feeding rather than continuous feeding be used in critically ill pediatric patients undergoing gastric feeding?**

**Answer**: We suggest using bolus feeding rather than continuous feeding in critically ill pediatric patients undergoing gastric feeding (GRADE 2C, certainty of evidence = “low”).

**Rationale**

Although gastric feeding is a common procedure for critically ill pediatric patients, there is a paucity of evidence regarding the difference between bolus gastric feeding (BGF) and CGF. CGF is expected to reduce intolerance and improve the nutritional status of critically ill pediatric patients. On the other hand, BGF may reduce the interruption of enteral feeding related to interventions, particularly in infants, who are very susceptible to gastroesophageal reflux [609]. Furthermore, BGF has been shown to accelerate protein synthesis in neonates more than CGF [610]. There are no recommendations in SCCM-ASPEN Guideline 2017 [571]. The ESPNIC guidelines state that there is no evidence to suggest that either method is superior to the other [572]. We conducted a systematic review and meta-analysis to investigate whether BGF is preferable to CGF for critically ill pediatric patients, except for post-pyloric feeding.

A meta-analysis was performed using 4 randomized controlled trials (RCTs) (Additional file 4) [611–614] and one post hoc analysis [615]. The results of the favorable outcomes were as follows: duration of mechanical ventilation yielded a mean difference (MD) of 1.0 day shorter (95% CI 6.0 shorter to 4.0 longer) (1 RCT: *n* = 25), emesis yielded a RD of 5 fewer per 1000 (95% CI 98 fewer to 206 more) (4 RCTs: *n* = 276), diarrhea yielded a RD of 23 fewer per 1000 (95% CI 212 fewer to 323 more) (2 RCTs: *n* = 105), and the incidence of increased GRV yielded a RD of 69 fewer per 1000 (95% CI 165 fewer to 76 more) (more than 5 mL/kg; 2 RCTs: *n* = 191) in the BGF group. Therefore, the favorable outcomes were judged as small. The results of the unfavorable outcomes were as follows: length of ICU stay yielded a MD of 0.6 days longer (95% CI 3.2 shorter to 4.4 longer) (2 RCTs: *n* = 85), time to target feeding goal yielded a MD of 0.4 days longer (95% CI 1.1 shorter to 1.9 longer) (2 RCTs: *n* = 206). Therefore, the unfavorable outcomes were judged as trivial. Based on the overall balance of effects, we thought that bolus gastric feeding was likely superior.

No evidence is available regarding the difference in medical resource requirements between BGF and CGF. BGF may reduce the requirements for medical equipment, such as feeding pumps, but may also require more nursing contact and is associated with an increased risk of infectious transmission. However, in the context of critical care, these factors will not give rise to significant concerns.

The present meta-analysis included a limited number of subjects with circulatory instability. Therefore, the optimal method for this population has yet to be clarified.

Furthermore, among the studies included in the meta-analysis, one that exclusively examined older children (5–17 years) [572] showed that the length of the ICU stay and time to the target feeding goal were both shorter in the CGF group than in the BGF group. In contrast, other studies involving younger children reported opposite findings. Although the current recommendation is generally acceptable for practical purposes, an age-stratified analysis is required to establish the optimal approach.

Another RCT (Table 23) [616] was published after the literature search. In a comparison with CGF, the meta-analysis including this study detected one large favorable outcome (a decrease in mortality), two small favorable outcomes (decreases in mechanical ventilation days and GRV), and two small unfavorable outcomes (emesis and diarrhea) of BGF. Consequently, the recommendation remained unchanged.

**Table 23:** Findings of the meta-analysis including Kumar 2023 (Ref [618])

Mortality	138 fewer per 1000 (229 fewer to 132 more)
ICU LOS	0.31 days shorter (3.45 days shorter to 2.83 days longer)
MV duration	2.5 days shorter (6.23 days shorter to 1.24 days longer)
Infectious events	0 (not reported in either group)
Emesis	54 more per 1000 (68 fewer to 351 more)
Diarrhea	34 more per 1000 (126 fewer to 299 more)
Incidence of increased GRV	69 fewer per 1000 (165 fewer to 76 more)
Time to the target feeding goal	0.45 days longer (0.64 days shorter to 1.55 days longer)

Based on the balance of these effects, we concluded that the intervention of bolus administration was likely superior.

**CQ4-8: Should energy/protein-dense formulas be administered to critically ill pediatric patients who are receiving enteral nutrition?**

**Answer**: We suggest administering of energy/protein-dense formulas (0.9 to 1.0 kcal/mL) to critically ill pediatric patients (Grade 2D, certainty of evidence = “very low”).

**Rationale**

Critically ill pediatric patients, including those who undergo surgical interventions for congenital heart disease, often require a restricted water intake because of acute heart failure or respiratory failure. The amount of water intake necessary for intravenous drug administration often limits the amount of water intake that can be utilized for nutritional administration in critically ill pediatric patients. On the other hand, elevated risk of malnutrition and growth impairment in these patients calls for careful consideration in the planning and implementation of nutrition therapy. To achieve water intake restriction while ensuring adequate nutritional provision, energy/protein-dense formulas (0.9 to 1.0 kcal/mL) are frequently utilized for critically ill pediatric patients. The utility and safety of energy/protein-dense formulas and standard formulas (0.67 to 0.82 kcal/mL) are attracting increasing interest and, thus, this CQ was established.

A meta-analysis was performed using 7 randomized controlled trials (RCTs) (Additional file 4) [617–623]. The results of the favorable outcomes were as follows: length of mechanical ventilation yielded a MD of 0.2 days shorter (95% CI 0.6 shorter to 0.2 longer) (4 RCTs, *n* = 371), gastrointestinal bleeding yielded a RD of 45 fewer per 1000 (95% CI 126 less to 130 more) (2 RCTs, *n* = 109), and weight-for-age Z score yielded a MD of 0.61 higher (95% CI 0.28 higher to 0.94 higher) (3 RCTs, *n* = 367). Therefore, the favorable outcomes were judged as moderate. The results of unfavorable outcomes were as follows: mortality yielded a RD of 25 more per 1000 (95% CI 6 less to 109 more per 1000) (4 RCTs, *n* = 417), length of ICU stay yielded a MD of 0.1 days longer (95% CI 0.7 shorter to 0.9 longer) (5 RCTs, *n* = 405), emesis yielded a RD of 16 more per 1000 (95% CI 7 less to 79 more) (5 RCTs, *n* = 469), and diarrhea yielded a RD of 18 more per 1000 (95% CI 11 less to 96 more) (5 RCTs, *n* = 430). Therefore, the unfavorable outcomes were judged as small. Based on the overall balance of effects, we thought that administering of energy/protein-dense formulas was likely superior. The effect on the Z score, which indicates the difference between the value and its average divided by its standard deviation, strongly contributed to our decision because critically ill pediatric patients with congenital heart disease have a lower average Z score than healthy subjects and the observed effect of 0.61 more needs to be regarded as a moderate effect.

Additionally, we performed a sensitivity analysis because of the high mortality rate observed in a previous study [619]. The sensitivity analysis excluding this study (6 RCTs, *n* = 461) revealed similar effects.

There are currently no data on the acceptability of administering energy/protein-dense formulas. However, the preparation of energy/protein-dense formulas is not burdensome. Furthermore, the administration of these formulas will be feasible at almost any health care facility because there are few additional human and material resources needed to maintain this treatment. Therefore, we considered the administration of energy/protein-dense formulas to be acceptable.

It is important to note that 6 of the 7 RCTs analyzed involved patients in the perioperative period for congenital heart disease. The remaining RCT examined patients with acute bronchitis. Most of the data analyzed were for pediatric patients who underwent surgical interventions for congenital heart diseases. Furthermore, in these RCTs, the actual energy intake fell within the standard dosage range in the majority of the patients receiving energy/protein-dense formulas, whereas insufficient energy intake was more prevalent among those receiving standard formulas.

Based on the balance of these effects, we concluded that the intervention of administering energy/protein-dense formulas was likely superior.

## Supplementary Information

Additional file 1: CQ1 Evidence profiles.

Additional file 2: CQ2 Evidence profiles.

Additional file 3: CQ3 Evidence profiles.

Additional file 4: CQ4 Evidence profiles.

Additional file 5: Nutritional Assessment.

Additional file 6: Other supplementary files.

## Data Availability

The datasets used and/or analyzed during the present study are available from the corresponding author upon reasonable request.
