# Correction: The Japanese Critical Care Nutrition Guideline 2024

**DOI:** 10.1186/s40560-026-00856-9

**Published:** 2026-02-28

**Authors:** Kensuke Nakamura, Ryo Yamamoto, Naoki Higashibeppu, Minoru Yoshida, Hiroomi Tatsumi, Yoshiyuki Shimizu, Hiroo Izumino, Taku Oshima, Junji Hatakeyama, Akira Ouchi, Rie Tsutsumi, Norihiko Tsuboi, Natsuhiro Yamamoto, Ayumu Nozaki, Sadaharu Asami, Yudai Takatani, Kohei Yamada, Yujiro Matsuishi, Shuhei Takauji, Akihito Tampo, Yusuke Terasaka, Takeaki Sato, Saiko Okamoto, Hideaki Sakuramoto, Tomoka Miyagi, Keisei Aki, Hidehito Ota, Taro Watanabe, Nobuto Nakanishi, Hiroyuki Ohbe, Chihiro Narita, Jun Takeshita, Masano Sagawa, Takefumi Tsunemitsu, Shinya Matsushima, Daisuke Kobashi, Yorihide Yanagita, Shinichi Watanabe, Hiroyasu Murata, Akihisa Taguchi, Takuya Hiramoto, Satomi Ichimaru, Muneyuki Takeuchi, Joji Kotani

**Affiliations:** 1 Department of Critical Care Medicine, Yokohama City University Hospital, 3‑9 Fukuura, Kanazawa‑ku, Yokohama, Kanagawa 236‑0004 Japan; 2 Department of Emergency and Critical Care Medicine, Keio University School of Medicine, Tokyo, Japan; 3 Department of Anesthesia and Critical Care, Kobe City Medical Center General Hospital, Kobe, Japan; 4 Department of Emergency and Critical Care Medicine, St. Marianna University School of Medicine, Kanagawa, Japan; 5 Department of Intensive Care Medicine, Sapporo Medical University School of Medicine, Sapporo, Japan; 6 Department of Intensive Care Medicine, Osaka Women’s and Children’s Hospital, Osaka, Japan; 7 Acute and Critical Care Center, Nagasaki University Hospital, Nagasaki, Japan; 8 Department of Emergency and Critical Care Medicine, Chiba University Graduate School of Medicine, Chiba City, Japan; 9 Department of Emergency and Critical Care Medicine, Osaka Medical and Pharmaceutical University, Osaka, Japan; 10 Department of Adult Health Nursing, College of Nursing, Ibaraki Christian University, Hitachi, Japan; 11 Department of Anesthesiology and Critical Care, Hiroshima University Hospital, Hiroshima, Japan; 12 Department of Critical Care Medicine and Anesthesia, National Center for Child Health and Development, Tokyo, Japan; 13 Department of Anesthesiology and Critical Care Medicine, Yokohama City University School of Medicine, Kanagawa, Japan; 14 Department of Pharmacy, Kyoto-Katsura Hospital, Kyoto, Japan; 15 Department of Cardiology, Musashino Tokushukai Hospital, Tokyo, Japan; 16 Department of Primary Care and Emergency Medicine, Kyoto University Hospital, Kyoto, Japan; 17 Department of Traumatology and Critical Care Medicine, National Defense Medical College Hospital, Saitama, Japan; 18 Adult and Elderly Nursing, Faculty of Nursing, Tokyo University of Information Science, Chiba, Japan; 19 Department of Emergency Medicine, Hokkaido University Hospital, Sapporo, Japan; 20 Department of Emergency Medicine, Asahikawa Medical University, Asahikawa, Japan; 21 Department of Emergency Medicine, Kyoto Katsura Hospital, Kyoto, Japan; 22 Tohoku University Hospital Emergency Center, Miyagi, Japan; 23 Department of Nursing, Hitachi General Hospital, Hitachi, Japan; 24 Department of Acute Care Nursing, Japanese Red Cross Kyushu International College of Nursing, Munakata, Japan; 25 Anesthesiology and Critical Care Medicine, Master’s Degree Program, Graduate School of Medicine, Yokohama City University, Kanagawa, Japan; 26 Department of Pharmacy, Kokura Memorial Hospital, Fukuoka, Japan; 27 Department of Pediatrics, School of Medicine, The University of Tokyo, Tokyo, Japan; 28 Department of Intensive Care Medicine, Showa University School of Medicine, Tokyo, Japan; 29 Division of Disaster and Emergency Medicine, Department of Surgery Related, Kobe University Graduate School of Medicine, Kobe, Japan; 30 Department of Emergency and Critical Care Medicine, Tohoku University Hospital, Sendai, Japan; 31 Department of Emergency Medicine, Shizuoka General Hospital, Shizuoka, Japan; 32 Department of Anesthesiology, Osaka Women’s and Children’s Hospital, Izumi, Japan; 33 Department of Surgery, Tokyo Women’s Medical University Adachi Medical Center, Tokyo, Japan; 34 Department of Preventive Services, Graduate School of Medicine, Kyoto University, Kyoto, Japan; 35 Department of Physical Therapy, Faculty of Health Science, Kyorin University, Tokyo, Japan; 36 Department of Critical Care and Emergency Medicine, Japanese Red Cross Maebashi Hospital, Gunma, Japan; 37 Department of Health Sciences, Institute of Biomedical Sciences, Nagasaki University, Nagasaki, Japan; 38 Department of Physical Therapy, Faculty of Rehabilitation, Gifu University of Health Science, Gifu, Japan; 39 Department of Rehabilitation Medicine, Kyorin University Hospital, Tokyo, Japan; 40 Department of Anesthesia, Kyoto University Hospital, Kyoto, Japan; 41 Department of Internal Medicine, Tokyo Bay Urayasu Ichikawa Medical Center, Urayasu, Japan; 42 Food and Nutrition Service Department, Fujita Health University Hospital, Aichi, Japan; 43 Department of Critical Care Medicine, National Cerebral and Cardiovascular Center, Osaka, Japan

**Correction: Journal of Intensive Care (2025) 13:18** 10.1186/s40560-025-00785-z

Following publication of the original article [1], the authors reported the following errors, which have been corrected:

1. The changes in the main text:

**Table:** 

Item	Old (pre-correction)	New (post-correction)
Main text		
Cited studies (p. 19)	“A meta-analysis was performed using **12** RCTs (Additional file 2) [18, 102, 103, 188, 208, 224, **236–241**].	“A meta-analysis was performed using **20** RCTs (Additional file 2) [18, 102, 103, **175**, 188, 208, 224, **228, 236–247**].”
Added references	➖	Added references 242–247 and renumbered subsequent references accordingly.
Mortality (p. 19)	incidence of mortality yielded an RD of **23** more per 1000 (95% CI **19** fewer to **87** more) (12 RCTs, n = **1467**).	incidence of mortality yielded an RD of **0** more per 1000 (95% CI **40** fewer to **53** more) (12 RCTs, n = **1021**).
Diarrhea (p. 19)	diarrhea yielded an RD of **28** fewer per 1000 patients (95% CI **118** fewer to **104** more) (12 RCTs, n = **877**).	diarrhea yielded an RD of **6** fewer per 1000 patients (95% CI **92** fewer to **108** more) (12 RCTs, n = **909**).
Length of ICU stay (p. 19)	length of ICU stay yielded a MD of 1.2 days shorter (95% CI 2.4 days shorter to 0.08 days shorter) (**10** RCTs, n = 669).	length of ICU stay yielded a MD of 1.2 days shorter (95% CI 2.4 days shorter to 0.08 days shorter) (**9** RCTs, n = 669).
Rationale text (p. 19)	However, **the potential increase in mortality associated with its use cannot be ruled out and needs to be considered when evaluating its acceptability**.	However, **the balance of effects does not favor the intervention.**

Note: the bold text indicates the specific parts that have been revised in the article

2. Additional files 2 and 6 have been updated.

The original article [1] has been updated.
