# Supplementary material for: The Japanese Critical Care Nutrition Guideline 2024
Source: J Intensive Care. 2025 Mar 21;13:18. doi: 10.1186/s40560-025-00785-z (PMC11927338; doi:10.1186/s40560-025-00785-z)
Supplement: Supplementary file 1 — Additional file 1: CQ1 Evidence profiles. [file 40560_2025_785_MOESM1_ESM.docx]

| **Table 1. CQ1-1 Evidence profile** | | | | | | | | | | |
| --- | --- | --- | --- | --- | --- | --- | --- | --- | --- | --- |
| **Certainty assessment** | | | | | | | **Summary of findings** | | | |
| **Participants (studies)** | **Risk of bias** | **Inconsistency** | **Indirectness** | **Imprecision** | **Publication bias** | **Overall certainty  of evidence** | **Study event rates (%)** | | **Relative effect (95% CI)** | **Anticipated absolute effects (95% CI)** |
|  |  |  |  |  |  |  | **With control** | **With feeding protocol** |  |  |
| **Overall mortality** | | | | | | | | | | |
| 2192 (6 RCTs) | not serious | not serious | not serious | not serious | None | ⨁⨁⨁⨁ High | 227/1072 (21.2%) | 274/1120 (24.5%) | **RR 0.96** (0.82 to 1.14) | **8 fewer per 1,000** (from 38 fewer to 30 more) |
| **Length of ICU stay** | | | | | | | | | | |
| 1092 (5 RCTs) | serious^a^ | not serious | not serious | serious^b^ | None | ⨁⨁◯◯ Low | 537 | 555 | - | **MD 0.41 shorter** (1.25 shorter to 0.43 longer) |
| **Duration of mechanical ventilation** | | | | | | | | | | |
| 2173 (4 RCTs) | serious^a^ | serious^c^ | not serious | not serious | None | ⨁⨁◯◯ Low | 1076 | 1097 | - | **SMD 0.27 longer** (0.17 shorter to 0.71 longer) |
| **Renal replacement therapy (assessed with 10 patient days)** | | | | | | | | | | |
| 2970 (2 RCTs) | not serious | not serious | serious^d^ | serious^b^ | None | ⨁⨁◯◯ Low | 1473 participants | 1497 participants | **RD -0.29** (-0.60 to 0.02) | **29 fewer per 1,000** (from 60 fewer to 2 more) |
| **Pneumonia** | | | | | | | | | | |
| 576 (2 RCTs) | serious^a^ | not serious | not serious | serious^b^ | None | ⨁⨁◯◯ Low | 25/294 (8.5%) | 22/282 (7.8%) | **RR 0.81** (0.24 to 2.74) | **16 fewer per 1,000** (from 65 fewer to 148 more) |
| **Vomiting** | | | | | | | | | | |
| 576 (2 RCTs) | serious^a^ | not serious | not serious | serious^b^ | None | ⨁⨁◯◯ Low | 12/294 (4.1%) | 14/282 (5.0%) | **RR 1.22** (0.57 to 2.60) | **9 more per 1,000** (from 18 fewer to 65 more) |
| **Enteral nutrition initiation time (days)** | | | | | | | | | | |
| 3854 (6 RCTs) | serious^a^ | not serious | not serious | not serious | None | ⨁⨁⨁◯ Moderate | 1906 | 1948 | - | **MD 0.35 shorter** (0.58 shorter to 0.12 longer) |

**CI:** confidence interval; **MD:** mean difference; **RR:** risk ratio; **SMD:** standardized mean difference

a. Downgraded one level due to risk of bias.

b. Downgraded one level due to imprecision (wide range of 95% confidence interval).

c. Downgraded one level due to considerable heterogeneity (I^2 = 97%).

d. Downgraded one level due to proxy outcome.

| **Table 2. CQ1-2 Evidence profile** | | | | | | | | | | |
| --- | --- | --- | --- | --- | --- | --- | --- | --- | --- | --- |
| **Certainty assessment** | | | | | | | **Summary of findings** | | | |
| **Participants (studies)** | **Risk of bias** | **Inconsistency** | **Indirectness** | **Imprecision** | **Publication bias** | **Overall certainty of evidence** | **Study event rates (%)** | | **Relative effect (95% CI)** | **Anticipated absolute effects (95% CI)** |
|  |  |  |  |  |  |  | **With PN** | **With EN** |  |  |
| **90-day mortality** | | | | | | | | | | |
| 4800 (3 RCTs) | not serious | not serious | not serious | not serious | none | ⨁⨁⨁⨁ High | 955/2399 (39.8%) | 999/2401 (41.6%) | **RR 1.05** (0.98 to 1.12) | **20 more per 1,000** (from 8 fewer to 48 more) |
| **Sepsis (including bacteremia)** | | | | | | | | | | |
| 5892 (15 RCTs) | serious^a^ | not serious | not serious | not serious | none | ⨁⨁⨁◯ Moderate | 189/2914 (6.5%) | 122/2978 (4.1%) | **RR 0.57** (0.43 to 0.77) | **28 fewer per 1,000** (from 37 fewer to 15 fewer) |
| **Pneumonia** | | | | | | | | | | |
| 5943 (18 RCTs) | serious^b^ | not serious | not serious | not serious | none | ⨁⨁⨁◯ Moderate | 379/2970 (12.8%) | 364/2973 (12.2%) | **RR 0.93** (0.75 to 1.15) | **9 fewer per 1,000** (from 32 fewer to 19 more) |
| **Length of ICU stay** | | | | | | | | | | |
| 5431 (14 RCTs) | serious^c^ | serious^d^ | not serious | not serious | none | ⨁⨁◯◯ Low | 2722 | 2709 | - | **MD 0.94 shorter** (1.8 shorter to 0.07 shorter) |
| **Duration of mechanical ventilation** | | | | | | | | | | |
| 268 (5 RCTs) | serious^e^ | very serious^f^ | not serious | very serious^g^ | none | ⨁◯◯◯ Very low | 128 | 140 | - | **MD 0.43 shorter** (3.6 shorter to 2.73 longer) |
| **One year EQ-5D** | | | | | | | | | | |
| 1335 (1 RCT) | not serious | not serious | not serious | not serious | none | ⨁⨁⨁⨁ High | 676 | 659 | - | **MD 0** (0.03 lower to 0.03 higher) |
| **Mesenteric ischemia** | | | | | | | | | | |
| 4861 (3 RCTs) | not serious | not serious | not serious | not serious | none | ⨁⨁⨁⨁ High | 13/2430 (0.5%) | 32/2431 (1.3%) | **RR 2.34** (1.07 to 5.13) | **7 more per 1,000** (from 0 more to 22 more) |

**CI:** confidence interval; **EN**: enteral nutrition; **MD:** mean difference; **RR:** risk ratio; **PN:** parenteral nutrition; **EQ5D5L:** EuroQol-5Dimensions-5Level

a. Downgraded one level: two key studies (Harvey 2014, Reignier 2018, combined weight 32.9%) are of some concern, and the other five studies accounting for more than 5% of the weight (Borzotta 1994, Lam 2008, Doley 2009, Altintas 2011, Altintas 2011) are of high risk of bias.

b. Downgraded one level: two key studies（Harvey2014, Reignier 2018, combined weight 32.8%）are of some concern、and the other six studies accounting for more than 5% of the weight（Adams1986, Hadley 1986, Borzotta 1994, Lam 2008, Aydogmus 2012, Fan 2016）are of high risk of bias.

c. Downgraded one level: of the seven studies with a weight of more than 5%, three (Kalfarentzos 1997, Harvey 2014, Reignier 2018, combined Weight 31.8%) are of some concern, and four (Kudsk 1992, Zhang 2005, Wan 2014, Takesue 2015, combined weight 52.4%) are of high risk of bias.

d. Downgraded one level: point estimates differ between studies and the differences are substantial, each confidence interval is not free of overlap, direction of effect is constant, proportion of variation is large (I^2 = 87%), P<0.00001.

e. Downgraded one level: out of 5 studies, 4 studies (combined weight 78.4%) are of high risk of bias, and 1 study (Kalfarentzos 1997, weight 21.6) is of some concern.

f. Downgraded two levels: point estimates differ between studies and the differences are substantial, each confidence interval is not free of overlap, direction of effect is not constant, proportion of variation is large (I^2 = 86%), P<0.00001.

g. Downgraded two levels: confidence interval for effect estimate includes considerable benefit and considerable harm.

| **Table 3. CQ1-2 Evidence profile (Sensitivity analysis: after 2012)** | | | | | | | | | | |
| --- | --- | --- | --- | --- | --- | --- | --- | --- | --- | --- |
| **Certainty assessment** | | | | | | | **Summary of findings** | | | |
| **Participants (studies)** | **Risk of bias** | **Inconsistency** | **Indirectness** | **Imprecision** | **Publication bias** | **Overall certainty of evidence** | **Study event rates (%)** | | **Relative effect (95% CI)** | **Anticipated absolute effects (95% CI)** |
|  |  |  |  |  |  |  | **With PN** | **With EN** |  |  |
| **90-day mortality** | | | | | | | | | | |
| 4749 (2 RCTs) | not serious | not serious | not serious | not serious | none | ⨁⨁⨁⨁ High | 949/2376 (39.9%) | 994/2373 (41.9%) | RR 1.05 (0.98 to 1.12) | **20 more per 1,000** (from 8 fewer to 48 more) |
| **Sepsis (including bacteremia)** | | | | | | | | | | |
| 5095 (5 RCTs) | very serious^a^ | serious^b^ | not serious | not serious | none | ⨁◯◯◯ Very low | 125/2513 (5.0%) | 85/2582 (3.3%) | RR 0.54 (0.33 to 0.89) | **23 fewer per 1,000** (from 33 fewer to 5 fewer) |
| **Pneumonia** | | | | | | | | | | |
| 4965 (5 RCTs) | serious^c^ | serious^d^ | not serious | serious^e^ | none | ⨁◯◯◯ Very low | 278/2482 (11.2%) | 288/2483 (11.6%) | RR 1.06 (0.79 to 1.43) | **7 more per 1,000** (from 24 fewer to 48 more) |
| **Length of ICU stay** | | | | | | | | | | |
| 5067 (6 RCTs) | very serious^f^ | not serious | not serious | not serious | none | ⨁⨁◯◯ Low | 2534 | 2533 | - | MD **1.1 shorter** (1.88 shorter to 0.31 longer) |
| **Duration of mechanical ventilation** | | | | | | | | | | |
| 80 (1 RCT) | very serious^g^ | not serious | not serious | serious^h^ | none | ⨁◯◯◯ Very low | 40 | 40 | - | MD **6.07 shorter** (8.6 shorter to 3.54 longer) |
| **One year EQ-5D** | | | | | | | | | | |
| 1335 (1 RCT) | not serious | not serious | not serious | not serious | none | ⨁⨁⨁⨁ High | 676 | 659 | - | MD **0**  (0.03 lower to 0.03 higher) |
| **Mesenteric ischemia** | | | | | | | | | | |
| 4798 (2 RCTs) | not serious | not serious | not serious | serious^e^ | none | ⨁⨁⨁◯ Moderate | 13/2399 (0.5%) | 30/2399 (1.3%) | RR 2.25 (0.82 to 6.17) | **7 more per 1,000** (from 1 fewer to 28 more) |

**CI:** confidence interval; **MD:** mean difference; **RR:** risk ratio

a. Downgraded two levels: high proportion of the information comes from studies with a high risk of bias.
b. Downgraded one level: heterogeneity test is p=0.04, I^2 is 61%. There is a slight variation in the point estimates, but the confidence intervals almost overlap.
c. Downgraded one level due to some concern about risk of bias.
d. Downgraded one level: heterogeneity test is p=0.06, I^2 is 55%. There is some variation in the point estimates, but the confidence intervals almost overlap.
e. Downgraded one level: confidence interval for effect estimate includes null and considerable harm.
f. Downgraded two levels: high proportion of the information comes from studies with a high risk of bias.
g. Downgraded two level: high proportion of the information comes from one study with a high risk of bias.
h. Downgraded one level: sample size does not meet the optimal information size.

| **Table 4. CQ1-2 Evidence profile (Sensitivity analysis: bias risk low and some concern)** | | | | | | | | | | |
| --- | --- | --- | --- | --- | --- | --- | --- | --- | --- | --- |
| **Certainty assessment** | | | | | | | **Summary of findings** | | | |
| **Participants (studies)** | **Risk of bias** | **Inconsistency** | **Indirectness** | **Imprecision** | **Publication bias** | **Overall certainty of evidence** | **Study event rates (%)** | | **Relative effect (95% CI)** | **Anticipated absolute effects (95% CI)** |
|  |  |  |  |  |  |  | **With PN** | **With EN** |  |  |
| **90-day mortality** | | | | | | | | | | |
| 4800 (3 RCTs) | not serious | not serious | not serious | not serious | none | ⨁⨁⨁⨁ High | 955/2399 (39.8%) | 999/2401 (41.6%) | RR 1.05 (0.98 to 1.12) | **20 more per 1,000** (from 8 fewer to 48 more) |
| **Sepsis (including bacteremia)** | | | | | | | | | | |
| 5123 (4 RCTs) | not serious | not serious | not serious | not serious | none | ⨁⨁⨁⨁ High | 87/2564 (3.4%) | 61/2559 (2.4%) | RR 0.71 (0.51 to 0.97) | **10 fewer per 1,000** (from 17 fewer to 1 fewer) |
| **Pneumonia** | | | | | | | | | | |
| 5123 (4 RCTs) | not serious | not serious | not serious | not serious | none | ⨁⨁⨁⨁ High | 268/2564 (10.5%) | 262/2559 (10.2%) | RR 0.97 (0.78 to 1.19) | **3 fewer per 1,000** (from 23 fewer to 20 more) |
| **Length of ICU stay** | | | | | | | | | | |
| 4943 (5 RCTs) | not serious | serious^a^ | not serious | very serious^b^ | none | ⨁◯◯◯ Very low | 2474 | 2469 | - | MD **0.18 shorter** (1.42 shorter to 1.06 longer) |
| **Duration of mechanical ventilation** | | | | | | | | | | |
| 38 (1 RCT) | not serious | not serious | not serious | serious^c^ | none | ⨁⨁⨁◯ Moderate | 20 | 18 | - | MD **4 longer** (0.93 longer to 7.07 longer) |
| **One year EQ-5D** | | | | | | | | | | |
| 1335 (1 RCT) | not serious | not serious | not serious | not serious | none | ⨁⨁⨁⨁ High | 676 | 659 | - | MD **0**  (0.03 lower to 0.03 higher) |
| **Mesenteric ischemia** | | | | | | | | | | |
| 4798 (2 RCTs) | not serious | serious^d^ | not serious | serious^e^ | none | ⨁⨁◯◯ Low | 13/2399 (0.5%) | 30/2399 (1.3%) | RR 2.25 (0.82 to 6.17) | **7 more per 1,000** (from 1 fewer to 28 more) |

**CI:** confidence interval; **MD:** mean difference; **RR:** risk ratio

a. Downgraded one level due to inconsistency (I^2=85).
b. Downgraded two levels: confidence interval for effect estimate includes considerable benefit and considerable harm.
c. Downgraded one level: sample size does not meet the optimal information size.
d. Downgraded one level due to inconsistency (I^2=56%).
e. Downgraded one level: confidence interval for effect estimate includes null and considerable harm.

| **Table 5. CQ1-2 Evidence profile (Sensitivity analysis: excluding studies in which enteral nutrition was administered at usual doses to patients with shock)** | | | | | | | | | | |
| --- | --- | --- | --- | --- | --- | --- | --- | --- | --- | --- |
| **Certainty assessment** | | | | | | | **Summary of findings** | | | |
| **Participants (studies)** | **Risk of bias** | **Inconsistency** | **Indirectness** | **Imprecision** | **Publication bias** | **Overall certainty of evidence** | **Study event rates (%)** | | **Relative effect (95% CI)** | **Anticipated absolute effects (95% CI)** |
|  |  |  |  |  |  |  | **With PN** | **With EN** |  |  |
| **90-day mortality** | | | | | | | | | | |
| 2423 (2 RCTs) | not serious | not serious | not serious | not serious | none | ⨁⨁⨁⨁ High | 448/1207 (37.1%) | 469/1216 (38.6%) | RR 1.04 (0.94 to 1.15) | **15 more per 1,000** (from 22 fewer to 56 more) |
| **Sepsis (including bacteremia)** | | | | | | | | | | |
| 3482 (14 RCTs) | serious^a^ | not serious | not serious | not serious | none | ⨁⨁⨁◯ Moderate | 134/1706 (7.9%) | 84/1776 (4.7%) | RR 0.55 (0.39 to 0.78) | **35 fewer per 1,000** (from 48 fewer to 17 fewer) |
| **Pneumonia** | | | | | | | | | | |
| 3533 (17 RCTs) | serious^a^ | not serious | not serious | serious^b^ | none | ⨁⨁◯◯ Low | 261/1762 (14.8%) | 251/1771 (14.2%) | RR 0.90 (0.69 to 1.17) | **15 fewer per 1,000** (from 46 fewer to 25 more) |
| **Length of ICU stay** | | | | | | | | | | |
| 3021 (13 RCTs) | serious^a^ | serious^c^ | not serious | not serious | none | ⨁⨁◯◯ Low | 1514 | 1507 | - | MD **1.01 shorter** (2 shorter to 0.02 shorter) |
| **Duration of mechanical ventilation** | | | | | | | | | | |
| 268 (5 RCTs) | serious^a^ | serious^d^ | not serious | very serious^e^ | none | ⨁◯◯◯ Very low | 128 | 140 | - | MD **0.43 shorter** (3.6 shorter to 2.73 longer) |
| **One year EQ-5D** | | | | | | | | | | |
| 1335 (1 RCT) | not serious | not serious | not serious | not serious | none | ⨁⨁⨁⨁ High | 676 | 659 | - | MD **0**  (0.03 lower to 0.03 higher) |
| **Mesenteric ischemia** | | | | | | | | | | |
| 2451 (2 RCTs) | not serious | not serious | not serious | very serious^e^ | none | ⨁⨁◯◯ Low | 8/1222 (0.7%) | 13/1229 (1.1%) | RR 1.52 (0.64 to 3.63) | **3 more per 1,000** (from 2 fewer to 17 more) |

**CI:** confidence interval; **MD:** mean difference; **RR:** risk ratio

a. Downgraded one level due to some concern about risk of bias.
b. Downgraded one level: confidence interval for effect estimate includes null and considerable benefit.
c. Downgraded one level due to inconsistency (I^2＝87％).
d. Downgraded one level due to inconsistency (I^2＝86％).
e. Downgraded two levels: confidence interval for effect estimate includes considerable benefit and considerable harm.

| **Table 6. CQ1-2 Evidence profile (Subgroup analysis: Trauma)** | | | | | | | | | | |
| --- | --- | --- | --- | --- | --- | --- | --- | --- | --- | --- |
| **Certainty assessment** | | | | | | | **Summary of findings** | | | |
| **Participants (studies)** | **Risk of bias** | **Inconsistency** | **Indirectness** | **Imprecision** | **Publication bias** | **Overall certainty of evidence** | **Study event rates (%)** | | **Relative effect (95% CI)** | **Anticipated absolute effects (95% CI)** |
|  |  |  |  |  |  |  | **With PN** | **With EN** |  |  |
| **90-day mortality** | | | | | | | | | | |
| 51 (1 RCT) | very serious^a^ | not serious | not serious | very serious^b^ | none | ⨁◯◯◯ Very low | 6/23 (26.1%) | 5/28 (17.9%) | RR 0.68 (0.24 to 1.96) | **83 fewer per 1,000** (from 198 fewer to 250 more) |
| **Sepsis (including bacteremia)** | | | | | | | | | | |
| 343 (6 RCTs) | very serious^a^ | not serious | not serious | very serious^b^ | none | ⨁◯◯◯ Very low | 32/163 (19.6%) | 16/180 (8.9%) | RR 0.49 (0.15 to 1.55) | **100 fewer per 1,000** (from 167 fewer to 108 more) |
| **Pneumonia** | | | | | | | | | | |
| 511 (9 RCTs) | very serious^a^ | not serious | not serious | very serious^b^ | none | ⨁◯◯◯ Very low | 64/247 (25.9%) | 73/264 (27.7%) | RR 1.06 (0.67 to 1.69) | **16 more per 1,000** (from 86 fewer to 179 more) |
| **Length of ICU stay** | | | | | | | | | | |
| 206 (4 RCTs) | very serious^a^ | serious^c^ | not serious | very serious^b^ | none | ⨁◯◯◯ Very low | 101 | 105 | - | MD **0.34 shorter** (4.03 shorter to 3.36 longer) |
| **Duration of mechanical ventilation** | | | | | | | | | | |
| 206 (3 RCTs) | very serious^a^ | serious^d^ | not serious | very serious^b^ | none | ⨁◯◯◯ Very low | 98 | 108 | - | MD **2.06 shorter** (6.7 shorter to 2.59 longer) |
| **One year EQ-5D** | | | | | | | | | | |
| 0 (0 studies) |  |  |  |  |  | - | 0 | 0 | - | not pooled |
| **Mesenteric ischemia** | | | | | | | | | | |
| 63 (1 RCT) | very serious^a^ | not serious | not serious | very serious^b^ | none | ⨁◯◯◯ Very low | 0/31 (0.0%) | 2/32 (6.3%) | RR 4.85 (0.24 to 97.11) | **0 more per 1,000** (from 0 fewer to 0 more) |

**CI:** confidence interval; **MD:** mean difference; **RR:** risk ratio

a. Downgraded two levels: high proportion of the information comes from studies with a high risk of bias.
b. Downgraded two levels: confidence interval for effect estimate includes considerable benefit and considerable harm.
c. Downgraded one level due to inconsistency (I^2＝79％).
d. Downgraded one level due to inconsistency (I^2＝90％).

| **Table 7. CQ1-2 Evidence profile (Subgroup analysis: acute pancreatitis)** | | | | | | | | | | |
| --- | --- | --- | --- | --- | --- | --- | --- | --- | --- | --- |
| **Participants (studies)** | **Risk of bias** | **Inconsistency** | **Indirectness** | **Imprecision** | **Publication bias** | **Overall certainty of evidence** | **Study event rates (%)** | | **Relative effect (95% CI)** | **Anticipated absolute effects (95% CI)** |
|  |  |  |  |  |  |  | **With PN** | **With EN** |  |  |
| **90-day mortality** | | | | | | | | | | |
| 0 (0 studies) |  |  |  |  |  | - | 0/0 | 0/0 | not pooled | not pooled |
| **Sepsis (including bacteremia)** | | | | | | | | | | |
| 271 (3 RCTs) | very serious^a^ | not serious | not serious | serious^b^ | none | ⨁◯◯◯ Very low | 35/105 (33.3%) | 27/166 (16.3%) | RR 0.46 (0.30 to 0.71) | **180 fewer per 1,000** (from 233 fewer to 97 fewer) |
| **Pneumonia** | | | | | | | | | | |
| 107 (2 RCTs) | serious^c^ | not serious | not serious | very serious^d^ | none | ⨁◯◯◯ Very low | 6/54 (11.1%) | 4/53 (7.5%) | RR 0.70 (0.21 to 2.34) | **33 fewer per 1,000** (from 88 fewer to 149 more) |
| **Length of ICU stay** | | | | | | | | | | |
| 230 (4 RCTs) | very serious^e^ | not serious | not serious | serious^b^ | none | ⨁◯◯◯ Very low | 117 | 113 | - | MD **1.8 shorter** (2.6 shorter to 1.01 shorter) |
| **Duration of mechanical ventilation** | | | | | | | | | | |
| 38 (1 RCT) | not serious | not serious | not serious | serious^b^ | none | ⨁⨁⨁◯ Moderate | 20 | 18 | - | MD **4 longer** (0.93 longer to 7.07 longer) |
| **One year EQ-5D** | | | | | | | | | | |
| 0 (0 studies) |  |  |  |  |  | - | 0 | 0 | - | not pooled |
| **Mesenteric ischemia** | | | | | | | | | | |
| 0 (0 studies) |  |  |  |  |  | - | 0/0 | 0/0 | not pooled | not pooled |

**CI:** confidence interval; **MD:** mean difference; **RR:** risk ratio

a. Downgraded two levels: high proportion of the information comes from studies with a high risk of bias.
b. Downgraded one level: sample size does not meet the optimal information size.
c. Downgraded one level due to some concern about risk of bias.
d. Downgraded two levels: confidence interval for effect estimate includes considerable benefit and considerable harm.
e. Downgraded two levels: high proportion of the information comes from studies with a high risk of bias.

| **Table 8. CQ1-2 Evidence profile (Subgroup analysis: internal medicine except acute pancreatitis)** | | | | | | | | | | |
| --- | --- | --- | --- | --- | --- | --- | --- | --- | --- | --- |
| **Certainty assessment** | | | | | | | **Summary of findings** | | | |
| **Participants (studies)** | **Risk of bias** | **Inconsistency** | **Indirectness** | **Imprecision** | **Publication bias** | **Overall certainty of evidence** | **Study event rates (%)** | | **Relative effect (95% CI)** | **Anticipated absolute effects (95% CI)** |
|  |  |  |  |  |  |  | **With PN** | **With EN** |  |  |
| **90-day mortality** | | | | | | | | | | |
| 4749 (2 RCTs) | not serious | not serious | not serious | not serious | none | ⨁⨁⨁⨁ High | 949/2376 (39.9%) | 994/2373 (41.9%) | RR 1.05 (0.98 to 1.12) | **20 more per 1,000** (from 8 fewer to 48 more) |
| **Sepsis (including bacteremia)** | | | | | | | | | | |
| 5156 (4 RCTs) | serious^a^ | not serious | not serious | not serious | none | ⨁⨁⨁◯ Moderate | 97/2585 (3.8%) | 67/2571 (2.6%) | RR 0.72 (0.53 to 0.97) | **11 fewer per 1,000** (from 18 fewer to 1 fewer) |
| **Pneumonia** | | | | | | | | | | |
| 5156 (4 RCTs) | serious^a^ | not serious | not serious | not serious | none | ⨁⨁⨁◯ Moderate | 275/2585 (10.6%) | 265/2571 (10.3%) | RR 0.95 (0.76 to 1.19) | **5 fewer per 1,000** (from 26 fewer to 20 more) |
| **Length of ICU stay** | | | | | | | | | | |
| 4948 (5 RCTs) | serious^a^ | not serious | not serious | not serious | none | ⨁⨁⨁◯ Moderate | 2481 | 2467 | - | MD **0.93 shorter** (1.32 shorter to 0.53 shorter) |
| **Duration of mechanical ventilation** | | | | | | | | | | |
| 0 (0 studies) |  |  |  |  |  | - | 0 | 0 | - | not pooled |
| **One year EQ-5D** | | | | | | | | | | |
| 1335 (1 RCT) | not serious | not serious | not serious | not serious | none | ⨁⨁⨁⨁ High | 676 | 659 | - | MD **0**  (0.03 lower to 0.03 higher) |
| **Mesenteric ischemia** | | | | | | | | | | |
| 4798 (2 RCTs) | not serious | not serious | not serious | serious^b^ | none | ⨁⨁⨁◯ Moderate | 13/2399 (0.5%) | 30/2399 (1.3%) | RR 2.25 (0.82 to 6.17) | **7 more per 1,000** (from 1 fewer to 28 more) |

**CI:** confidence interval; **MD:** mean difference; **RR:** risk ratio

a. Downgraded one level due to some concern about risk of bias.
b. Downgraded one level: confidence interval for effect estimate includes null and considerable harm.

| **Table 9. CQ1-3 Evidence profile** | | | | | | | | | | |
| --- | --- | --- | --- | --- | --- | --- | --- | --- | --- | --- |
| **Certainty assessment** | | | | | | | **Summary of findings** | | | |
| **Participants (studies)** | **Risk of bias** | **Inconsistency** | **Indirectness** | **Imprecision** | **Publication bias** | **Overall certainty  of evidence** | **Study event rates (%)** | | **Relative effect (95% CI)** | **Anticipated absolute effects**  **(95% CI)** |
|  |  |  |  |  |  |  | **With norm/hypercaloric** | **With hypocaloric** |  |  |
| **28-day mortality** | | | | | | | | | | |
| 7960 (13 RCTs) | not serious | not serious | serious^a^ | not serious | none | ⨁⨁⨁◯ Moderate | 781/4005 (19.5%) | 830/3955 (21.0%) | **RR 1.07** (0.98 to 1.17) | **14 more per 1,000** (from 4 fewer to 33 more) |
| **90-day mortality** | | | | | | | | | | |
| 10197 (7 RCTs) | not serious | not serious | serious^a^ | not serious | none | ⨁⨁⨁◯ Moderate | 998/5080 (19.6%) | 974/5117 (19.0%) | **RR 0.97** (0.90 to 1.05) | **6 fewer per 1,000** (from 20 fewer to 10 more) |
| **Length of ICU stay** | | | | | | | | | | |
| 9339 (22 RCTs) | not serious | serious^b^ | serious^a^ | not serious | none | ⨁⨁◯◯ Low | 4532 | 4807 | - | **MD 0.04 shorter** (1.23 shorter to 1.15 longer) |
| **Duration of mechanical ventilation** | | | | | | | | | | |
| 6306 (13 RCTs) | not serious | serious^b^ | serious^a^ | not serious | none | ⨁⨁◯◯ Low | 3141 | 3165 | - | **MD 0.15 longer** (0.67 shorter to 0.96 longer) |
| **All infectious complications** | | | | | | | | | | |
| 5941 (10 RCTs) | not serious | serious^b^ | serious^a^ | very serious^c^ | none | ⨁◯◯◯ Very low^c^ | 790/2969 (26.6%) | 705/2972 (23.7%) | **RR 0.97** (0.80 to 1.17) | **8 fewer per 1,000** (from 53 fewer to 45 more) |
| **All adverse events** | | | | | | | | | | |
| 4189 (3 RCTs) | not serious | not serious | serious^a^ | not serious | none | ⨁⨁⨁◯ Moderate | 59/2088 (2.8%) | 54/2101 (2.6%) | **RR 0.91** (0.63 to 1.31) | **3 fewer per 1,000** (from 10 fewer to 9 more) |
| **Vomiting** | | | | | | | | | | |
| 5940 (4 RCTs) | not serious | not serious | serious^a^ | not serious | none | ⨁⨁⨁◯ Moderate | 314/2956 (10.6%) | 301/2984 (10.1%) | **RR 0.95** (0.82 to 1.10) | **5 fewer per 1,000** (from 19 fewer to 11 more) |

**CI:** confidence interval; **MD:** mean difference; **RR:** risk ratio

a. Downgraded one level: the difference in nutritional dosage was predetermined in some studies while that difference arose as a result in the others.
b. Downgraded one level due to inconsistency.
c. Downgraded two levels: there were wide confidence intervals around line of no effect which failed to exclude significant benefit or harm.

| **Table 10. CQ1-4 Evidence profile** | | | | | | | | | | |
| --- | --- | --- | --- | --- | --- | --- | --- | --- | --- | --- |
| **Certainty assessment** | | | | | | | **Summary of findings** | | | |
| **Participants (studies)** | **Risk of bias** | **Inconsistency** | **Indirectness** | **Imprecision** | **Publication bias** | **Overall certainty  of evidence** | **Study event rates (%)** | | **Relative effect (95% CI)** | **Anticipated absolute effects (95% CI)** |
|  |  |  |  |  |  |  | **With normal-lower protein** | **With higher protein** |  |  |
| **Short-term mortality (≤60-day mortality)** | | | | | | | | | | |
| 1825 (8 RCTs) | not serious | not serious | not serious | serious^a^ | none | ⨁⨁⨁◯ Moderate | 258/916 (28.2%) | 271/909 (29.8%) | **RR 1.06** (0.92 to 1.22) | **17 more per 1,000** (from 23 fewer to 62 more) |
| **Length of ICU stay** | | | | | | | | | | |
| 1921 (9 RCTs) | serious^b^ | not serious | not serious | serious^c^ | none | ⨁⨁◯◯ Low | 963 | 958 | - | **MD 0.54 shorter** (1.53 shorter to 0.45 longer) |
| **Duration of mechanical ventilation** | | | | | | | | | | |
| 1814 (8 RCTs) | serious^b^ | not serious | not serious | not serious | none | ⨁⨁⨁◯ Moderate | 911 | 903 | - | **MD 0.01 longer** (0.52 shorter to 0.53 longer) |
| **Infectious complications** | | | | | | | | | | |
| 249 (3 RCTs) | serious^d^ | serious^e^ | not serious | very serious^f^ | none | ⨁◯◯◯ Very low | 65/122 (53.3%) | 59/127 (46.5%) | **RR 0.93** (0.66 to 1.31) | **37 fewer per 1,000** (from 181 fewer to 165 more) |
| **Handgrip strength** | | | | | | | | | | |
| 141 (2 RCTs) | not serious | not serious | not serious | very serious^f^ | none | ⨁⨁◯◯ Low | 76 | 65 | - | **MD 1.82 higher** (1.43 lower to 5.07 higher) |
| **Muscle mass reduction** | | | | | | | | | | |
| 191 (3 RCTs) | serious^d^ | not serious | not serious | serious^a^ | none | ⨁⨁◯◯ Low | 92 | 99 | - | **SMD 0.62 lower** (0.99 lower to 0.25 lower) |
| **Diarrhea** | | | | | | | | | | |
| 310 (4 RCTs) | not serious | not serious | serious^g^ | serious^c^ | none | ⨁⨁◯◯ Low | 77/153 (50.3%) | 66/157 (42.0%) | **RR 0.87** (0.70 to 1.08) | **65 fewer per 1,000** (from 151 fewer to 40 more) |

**CI:** confidence interval; **MD:** mean difference; **RR:** risk ratio; **SMD:** standardized mean difference

a. Downgraded one level: optimal Information size is not fulfilled.

b. Downgraded one level due to bias in measurement of the outcome.

c. Downgraded one level: confidence interval for effect estimate includes substantial benefit and null.

d. Downgraded one level due to bias in missing outcome data.

e. Downgraded one level due to due to high heterogeneity (I^2 = 60%).

f. Downgraded two levels: confidence interval for effect estimate includes both substantial benefit and harm.

g. Downgraded one level: the nutritional supplements administered in each study were not standardized, and it is possible that differences in components other than the amount of protein may have affected the incidence of diarrhea.

| **Table 11. CQ1-5 Evidence profile** | | | | | | | | | | |
| --- | --- | --- | --- | --- | --- | --- | --- | --- | --- | --- |
| **Certainty assessment** | | | | | | | **Summary of findings** | | | |
| **Participants (studies)** | **Risk of bias** | **Inconsistency** | **Indirectness** | **Imprecision** | **Publication bias** | **Overall certainty  of evidence** | **Study event rates (%)** | | **Relative effect (95% CI)** | **Anticipated absolute effects (95% CI)** |
|  |  |  |  |  |  |  | **With  comparison** | **With  intervention** |  |  |
| **Mortality (short term mortality)** | | | | | | | | | | |
| 666 (12 RCTs) | serious^a^ | not serious | not serious | serious^b^ | none | ⨁⨁◯◯ Low | 44/327 (13.5%) | 31/339 (9.1%) | **RR 0.74** (0.48 to 1.15) | **35 fewer per 1,000** (from 70 fewer to 20 more) |
| **Length of ICU stay** | | | | | | | | | | |
| 729 (12 RCTs) | very serious^c^ | serious^d^ | not serious | not serious | none | ⨁◯◯◯ Very low | 361 | 368 | - | **MD 2.44 shorter** (4.01 shorter to 0.87 shorter) |
| **Duration of mechanical ventilation** | | | | | | | | | | |
| 346 (8 RCTs) | serious^a^ | serious^d^ | not serious | not serious | none | ⨁⨁◯◯ Low | 172 | 174 | - | **MD 1.91 shorter** (3.77 shorter to 0.04 shorter) |
| **Infectious complications** | | | | | | | | | | |
| 366 (7 RCTs) | serious^a^ | not serious | not serious | serious^e^ | none | ⨁⨁◯◯ Low | 69/182 (37.9%) | 43/184 (23.4%) | **RR 0.61** (0.39 to 0.95) | **148 fewer per 1,000** (from 231 fewer to 19 fewer) |
| **Handgrip strength** | | | | | | | | | | |
| 100 (1 RCT) | not serious | not serious | not serious | serious^f^ | none | ⨁⨁⨁◯ Moderate | 50 | 50 | - | **MD 1.07 higher** (0.16 higher to 1.98 higher) |
| **All adverse events (diarrhea)** | | | | | | | | | | |
| 205 (3 RCTs) | serious^a^ | not serious | not serious | serious^g^ | none | ⨁⨁◯◯ Low | 9/98 (9.2%) | 6/107 (5.6%) | **RR 0.61** (0.23 to 1.66) | **36 fewer per 1,000** (from 71 fewer to 61 more) |
| **All adverse events (gastric retention)** | | | | | | | | | | |
| 257 (4 RCTs) | serious^a^ | not serious | not serious | serious^h^ | none | ⨁⨁◯◯ Low | 28/123 (22.8%) | 24/134 (17.9%) | **RR 0.81** (0.35 to 1.85) | **43 fewer per 1,000** (from 148 fewer to 193 more) |

**CI:** confidence interval; **MD:** mean difference; **RR:** risk ratio

a. Downgraded one level due to some concern about risk of bias.
b. Downgraded one level: total sample size = 666 < 800 and does not meet optimal information size (OIS).
c. Downgraded two levels: high proportion of the information comes from studies with high risk of bias.
d. Downgraded one level: I^2 is as high as 70%. The directionality of the forest plots differs slightly between the included studies.
e. Downgraded one level: total sample size = 366 < 800 and does not meet optimal information size (OIS).
f. Downgraded one level: total sample size＝100＜800 and does not meet optimal information size (OIS).
g. Downgraded one level: total sample size＝205 and does not meet optimal information size (OIS).
h. Downgraded one level: total sample size＝257 and does not meet optimal information size (OIS).

| **Table 12. CQ 1-5 Evidence profile (Subgroup analysis: enteral nutrition initiated within 24 hours)** | | | | | | | | | | |
| --- | --- | --- | --- | --- | --- | --- | --- | --- | --- | --- |
| **Certainty assessment** | | | | | | | **Summary of findings** | | | |
| **Participants (studies)** | **Risk of bias** | **Inconsistency** | **Indirectness** | **Imprecision** | **Publication bias** | **Overall certainty of evidence** | **Study event rates (%)** | | **Relative effect (95% CI)** | **Anticipated absolute effects (95% CI)** |
|  |  |  |  |  |  |  | **With Comparison** | **With Intervention** |  |  |
| **Mortality (short term mortality)** | | | | | | | | | | |
| 466 (8 RCTs) | serious^a^ | not serious | not serious | serious^b^ | none | ⨁⨁◯◯ Low | 31/231 (13.4%) | 18/235 (7.7%) | **RR 0.64** (0.37 to 1.12) | **48 fewer per 1,000** (from 85 fewer to 16 more) |
| **Length of ICU stay** | | | | | | | | | | |
| 523 (8 RCTs) | very serious^c^ | serious^d^ | not serious | not serious | none | ⨁◯◯◯ Very low | 262 | 261 | - | **MD 2.64 shorter** (4.55 shorter to 0.73 shorter) |
| **Duration of mechanical ventilation** | | | | | | | | | | |
| 206 (5 RCTs) | serious^a^ | not serious | not serious | serious^b^ | none | ⨁⨁◯◯ Low | 101 | 105 | - | **MD 1.69 shorter** (3.21 shorter to 0.17 shorter) |
| **Infectious complications** | | | | | | | | | | |
| 132 (3 RCTs) | serious^a^ | serious^d^ | not serious | very serious^e^ | none | ⨁◯◯◯ Very low | 31/65 (47.7%) | 22/67 (32.8%) | **RR 0.67** (0.35 to 1.25) | **157 fewer per 1,000** (from 310 fewer to 119 more) |
| **Handgrip strength** | | | | | | | | | | |
| 100 (1 RCT) | not serious | not serious | not serious | serious^b^ | none | ⨁⨁⨁◯ Moderate | 50 | 50 | - | **MD 1.07 higher** (0.16 higher to 1.98 higher) |
| **All adverse events (diarrhea)** | | | | | | | | | | |
| 87 (1 RCT) | serious^a^ | not serious | not serious | very serious^e^ | none | ⨁◯◯◯ Very low | 5/43 (11.6%) | 2/44 (4.5%) | **RR 0.39** (0.08 to 1.91) | **71 fewer per 1,000** (from 107 fewer to 106 more) |
| **All adverse events (gastric retention)** | | | | | | | | | | |
| 139 (2 RCTs) | serious^a^ | not serious | not serious | very serious^e^ | none | ⨁◯◯◯ Very low | 25/68 (36.8%) | 19/71 (26.8%) | **RR 0.38** (0.03 to 5.59) | **228 fewer per 1,000** (from 357 fewer to 1,000 more) |

**CI:** confidence interval; **MD:** mean difference; **RR:** risk ratio

a. Downgraded one level due to some concern about risk of bias.
b. Downgraded one level: sample size does not meet the optimal information size (OIS).
c. Downgraded two levels: high proportion of the information comes from studies with high risk of bias.
d. Downgraded one level: I^2 is around 50%. The directionality of the forest plots differs slightly between the included studies.
e. Downgraded two levels: confidence interval for effect estimate includes considerable benefit and considerable harm.

| **Table 13. CQ1-6 Evidence profile** | | | | | | | | | | |
| --- | --- | --- | --- | --- | --- | --- | --- | --- | --- | --- |
| **Certainty assessment** | | | | | | | **Summary of findings** | | | |
| **Participants (studies)** | **Risk of bias** | **Inconsistency** | **Indirectness** | **Imprecision** | **Publication bias** | **Overall certainty  of evidence** | **Study event rates (%)** | | **Relative effect (95% CI)** | **Anticipated absolute effects (95% CI)** |
|  |  |  |  |  |  |  | **With no EN** | **With EN** |  |  |
| **Hospital Mortality** | | | | | | | | | | |
| 2441 (2 RCTs) | not serious | serious^b^ | not serious | not serious | none | ⨁⨁⨁◯ Moderate | 485/1224 (39.6%) | 500/1217 (41.1%) | **RR 0.78** (0.31 to 2.00) | **87 fewer per 1,000** (from 273 fewer to 396 more) |
| **Length of ICU stay (days)** | | | | | | | | | | |
| 2410 (1 RCT) | serious^a^ | not serious | not serious | not serious | none | ⨁⨁⨁◯ Moderate | 1208 | 1202 | - | **MD 1 shorter** (1.68 shorter to 0.32 shorter) |
| **Duration of mechanical ventilation-free days** | | | | | | | | | | |
| 2441 (2 RCTs) | serious^a^ | serious^b^ | not serious | very serious^d^ | none | ⨁◯◯◯ Very low | 1224 | 1217 | - | **MD 5.25 longer** (7.72 shorter to 18.22 longer) |
| **ICU-acquired infection** | | | | | | | | | | |
| 2410 (1 RCT) | not serious | not serious | not serious | serious^c^ | none | ⨁⨁⨁◯ Moderate | 194/1208 (16.1%) | 173/1202 (14.4%) | **RR 0.90** (0.74 to 1.08) | **16 fewer per 1,000** (from 42 fewer to 13 more) |
| **Ventilator-associated pneumonia** | | | | | | | | | | |
| 2441 (2 RCTs) | not serious | not serious | not serious | serious^c^ | none | ⨁⨁⨁◯ Moderate | 119/1224 (9.7%) | 113/1217 (9.3%) | **RR 0.96** (0.75 to 1.22) | **4 fewer per 1,000** (from 24 fewer to 21 more) |
| **Mesenteric ischemia** | | | | | | | | | | |
| 2441 (2 RCTs) | serious^a^ | not serious | not serious | serious^e^ | none | ⨁⨁◯◯ Low | 5/1224 (0.4%) | 19/1217 (1.6%) | **RR 3.82** (1.43 to 10.19) | **12 more per 1,000** (from 2 more to 38 more) |
| **Vomiting** | | | | | | | | | | |
| 2441 (2 RCTs) | serious^a^ | serious^b^ | not serious | serious^c^ | none | ⨁◯◯◯ Very low | 255/1224 (20.8%) | 409/1217 (33.6%) | **RR 0.85** (0.19 to 3.79) | **31 fewer per 1,000** (from 169 fewer to 581 more) |

**CI:** confidence interval; **EN**: enteral nutrition; **MD:** mean difference; **RR:** risk ratio

a. Downgraded one level due to risk of bias.

b. Downgraded one level due to considerable heterogeneity.

c. Downgraded one level: confidence interval for effect estimate includes null and considerable benefit.

d. Downgraded two levels: confidence interval for effect estimate includes considerable benefit and considerable harm.

e. Downgraded one level: the number of events does not meet the optimal information size.

| **Table 14. CQ1-6 Evidence profile (Sensitivity analysis: Fixed-effect model)** | | | | | | | | | | |
| --- | --- | --- | --- | --- | --- | --- | --- | --- | --- | --- |
| **Certainty assessment** | | | | | | | **Summary of findings** | | | |
| **Participants (studies)** | **Risk of bias** | **Inconsistency** | **Indirectness** | **Imprecision** | **Publication bias** | **Overall certainty of evidence** | **Study event rates (%)** | | **Relative effect (95% CI)** | **Anticipated absolute effects (95% CI)** |
|  |  |  |  |  |  |  | **With No EN** | **With EN** |  |  |
| **Hospital Mortality** | | | | | | | | | | |
| 2441 (2 RCTs) | not serious | serious^b^ | not serious | not serious | none | ⨁⨁⨁◯ Moderate | 485/1224 (39.6%) | 500/1217 (41.1%) | **RR 1.04** (0.94 to 1.14) | **16 more per 1,000** (from 24 fewer to 55 more) |
| **Length of ICU stay (days)** | | | | | | | | | | |
| 2410 (1 RCT) | serious^a^ | not serious | not serious | not serious | none | ⨁⨁⨁◯ Moderate | 1208 | 1202 | - | MD **1 shorter** (1.68 shorter to 0.32 shorter) |
| **Duration of mechanical ventilation-free days** | | | | | | | | | | |
| 2441 (2 RCTs) | serious^a^ | serious^b^ | not serious | serious^c^ | none | ⨁◯◯◯ Very low | 1224 | 1217 | - | MD **0.18 shorter** (1.53 shorter to 1.18 longer) |
| **ICU-acquired infection** | | | | | | | | | | |
| 2410 (1 RCT) | not serious | not serious | not serious | serious^c^ | none | ⨁⨁⨁◯ Moderate | 194/1208 (16.1%) | 173/1202 (14.4%) | **RR 0.90** (0.74 to 1.08) | **16 fewer per 1,000** (from 42 fewer to 13 more) |
| **Ventilator-associated pneumonia** | | | | | | | | | | |
| 2441 (2 RCTs) | not serious | not serious | not serious | serious^c^ | none | ⨁⨁⨁◯ Moderate | 119/1224 (9.7%) | 113/1217 (9.3%) | **RR 0.95** (0.75 to 1.22) | **5 fewer per 1,000** (from 24 fewer to 21 more) |
| **Mesenteric ischemia** | | | | | | | | | | |
| 2441 (2 RCTs) | serious^a^ | not serious | not serious | serious^d^ | none | ⨁⨁◯◯ Low | 5/1224 (0.4%) | 19/1217 (1.6%) | **RR 3.82** (1.43 to 10.19) | **12 more per 1,000** (from 2 more to 38 more) |
| **Vomiting** | | | | | | | | | | |
| 2441 (2 RCTs) | serious^a^ | serious^b^ | not serious | not serious | none | ⨁⨁◯◯ Low | 255/1224 (20.8%) | 409/1217 (33.6%) | **RR 1.61** (1.41 to 1.85) | **127 more per 1,000** (from 85 more to 177 more) |

**CI:** confidence interval; **MD:** mean difference; **RR:** risk ratio

a. Downgraded one level due to risk of bias.

b. Downgraded one level due to considerable heterogeneity.

c. Downgraded one level: confidence interval for effect estimate includes null and considerable benefit.

d. Downgraded one level: the number of events does not meet the optimal information size.

| **Table 15. CQ1-7 Evidence profile** | | | | | | | | | | |
| --- | --- | --- | --- | --- | --- | --- | --- | --- | --- | --- |
| **Certainty assessment** | | | | | | | **Summary of findings** | | | |
| **Participants (studies)** | **Risk of bias** | **Inconsistency** | **Indirectness** | **Imprecision** | **Publication bias** | **Overall certainty  of evidence** | **Study event rates (%)** | | **Relative effect (95% CI)** | **Anticipated absolute effects (95% CI)** |
|  |  |  |  |  |  |  | **With EN** | **With EN+SPN** |  |  |
| **Mortality (60-/90-day)** | | | | | | | | | | |
| 6333 (5 RCTs) | not serious | not serious | not serious | not serious | none | ⨁⨁⨁⨁ High | 475/3176 (15.0%) | 467/3157 (14.8%) | **RR 0.99** (0.88 to 1.11) | **1 fewer per 1,000** (from 18 fewer to 16 more) |
| **Mortality (ICU-/28-day)** | | | | | | | | | | |
| 6731 (6 RCTs) | not serious | not serious | not serious | not serious | none | ⨁⨁⨁⨁ High | 314/3382 (9.3%) | 290/3349 (8.7%) | **RR 0.93** (0.80 to 1.08) | **6 fewer per 1,000** (from 19 fewer to 7 more) |
| **Duration of mechanical ventilation** | | | | | | | | | | |
| 6874 (8 RCTs) | not serious | serious^a^ | not serious | not serious | none | ⨁⨁⨁◯ Moderate | 3454 | 3420 | - | **MD 0.08 shorter** (0.87 shorter to 0.72 longer) |
| **Length of ICU Stay** | | | | | | | | | | |
| 6873 (9 RCTs) | not serious | serious^b^ | not serious | serious^c^ | none | ⨁⨁◯◯ Low | 3451 | 3422 | - | **MD 0.48 longer** (0.73 shorter to 1.68 longer) |
| **Infectious complications (all-cause)** | | | | | | | | | | |
| 6655 (6 RCTs) | not serious | not serious | not serious | not serious | none | ⨁⨁⨁⨁ High | 740/3346 (22.1%) | 802/3309 (24.2%) | **RR 1.07** (0.95 to 1.20) | **15 more per 1,000** (from 11 fewer to 44 more) |
| **Infectious complications (bloodstream infection)** | | | | | | | | | | |
| 6704 (6 RCTs) | not serious | not serious | not serious | not serious | none | ⨁⨁⨁⨁ High | 192/3370 (5.7%) | 242/3334 (7.3%) | **RR 1.26** (1.05 to 1.51) | **15 more per 1,000** (from 3 more to 29 more) |
| **SF-36 physical functioning domain** | | | | | | | | | | |
| 1157 (3 RCTs) | serious^d^ | not serious | not serious | very serious^e^ | none | ⨁◯◯◯ Very low | 578 | 579 | - | **MD 2.48 higher** (6.09 lower to 11.06 higher) |
| **Adverse events** | | | | | | | | | | |
| 4760 (2 RCTs) | not serious | not serious | not serious | not serious | none | ⨁⨁⨁⨁ High | 437/2372 (18.4%) | 428/2388 (17.9%) | **RR 0.97** (0.86 to 1.10) | **6 fewer per 1,000** (from 26 fewer to 18 more) |

**CI:** confidence interval; **EN**: enteral nutrition; **MD:** mean difference; **RR:** risk ratio; **SPN**: supplemental parenteral nutrition; **SF-36:** MOS 36-Item Short-Form Health Survey

a. Downgraded one level due to considerable heterogeneity (I^2 = 97%).

b. Downgraded one level due to considerable heterogeneity (I^2 = 96%).

c. Downgraded one level due to imprecision (95%CI of MD crosses 0 and MID crosses 1).

d. Downgraded one level due to risk of bias.

e. Downgraded two levels due to imprecision (wide range of 95% CI).

| **Table 16. CQ1-8 Evidence profile** | | | | | | | | | | |
| --- | --- | --- | --- | --- | --- | --- | --- | --- | --- | --- |
| **Certainty assessment** | | | | | | | **Summary of findings** | | | |
| **Participants (studies)** | **Risk of bias** | **Inconsistency** | **Indirectness** | **Imprecision** | **Publication bias** | **Overall certainty of evidence** | **Study event rates (%)** | | **Relative effect (95% CI)** | **Anticipated absolute effects (95% CI)** |
|  |  |  |  |  |  |  | **With  gastric feeding** | **With  post-pyloric feeding** |  |  |
| **All-cause mortality** | | | | | | | | | | |
| 1154 (13 RCTs) | not serious | not serious | not serious | serious^a^ | None | ⨁⨁⨁◯ Moderate | 176/594 (29.6%) | 169/560 (30.2%) | **RR 0.98** (0.83 to 1.16) | **6 fewer per 1,000** (from 50 fewer to 47 more) |
| **Length of ICU stay** | | | | | | | | | | |
| 941 (11 RCTs) | serious^b^ | serious^c^ | not serious | serious^d^ | None | ⨁◯◯◯ Very low | 484 | 457 | - | **MD 1.39 shorter** (2.92 shorter to 0.15 longer) |
| **Duration of mechanical ventilation** | | | | | | | | | | |
| 622 (7 RCTs) | serious^b^ | serious^c^ | not serious | not serious | None | ⨁⨁◯◯ Low | 320 | 302 | - | **MD 2.2 shorter** (3.36 shorter to 1.04 shorter) |
| **Pneumonia (ventilator-associated or aspiration)** | | | | | | | | | | |
| 1079 (13 RCTs) | serious^e^ | serious^f^ | not serious | serious^a^ | None | ⨁◯◯◯ Very low | 156/554 (28.2%) | 83/525 (15.8%) | **RR 0.60** (0.44 to 0.82) | **113 fewer per 1,000** (from 158 fewer to 51 fewer) |
| **The time from ICU admission to initiation of enteral nutrition (hours)** | | | | | | | | | | |
| 316 (4 RCTs) | serious^b^ | serious^g^ | not serious | serious^h^ | None | ⨁◯◯◯ Very low | 163 | 153 | - | **MD 9.57 longer** (0.52 shorter to 19.66 longer) |
| **Diarrhea** | | | | | | | | | | |
| 743 (8 RCTs) | serious^b^ | not serious | not serious | serious^h^ | None | ⨁⨁◯◯ Low | 62/380 (16.3%) | 63/363 (17.4%) | **RR 1.05** (0.77 to 1.44) | **8 more per 1,000** (from 38 fewer to 72 more) |
| **Vomiting** | | | | | | | | | | |
| 689 (8 RCTs) | serious^b^ | serious^f^ | not serious | serious^h^ | none | ⨁◯◯◯ Very low | 75/349 (21.5%) | 37/340 (10.9%) | **RR 0.58** (0.29 to 1.18) | **90 fewer per 1,000** (from 153 fewer to 39 more) |

**CI:** confidence interval; **MD:** mean difference; **RR:** risk ratio

a. Downgraded one level: optimal information size (OIS) is not met.
b. Downgraded one level due to some concern about risk of bias.
c. Downgraded one level due to moderate to high heterogeneity.
d. Downgraded one level: confidence interval for effect estimate includes null and considerable benefit.
e. Downgraded one level due to some concern about risk of bias.
f. Downgraded one level due to moderate heterogeneity.
g. Downgraded one level due to high heterogeneity.
h. Downgraded one level: confidence interval for effect estimate includes null and considerable harm.

| **Table 17. CQ1-9 Evidence profile** | | | | | | | | | | |
| --- | --- | --- | --- | --- | --- | --- | --- | --- | --- | --- |
| **Certainty assessment** | | | | | | | **Summary of findings** | | | |
| **Participants (studies)** | **Risk of bias** | **Inconsistency** | **Indirectness** | **Imprecision** | **Publication bias** | **Overall certainty of evidence** | **Study event rates (%)** | | **Relative effect (95% CI)** | **Anticipated absolute effects (95% CI)** |
|  |  |  |  |  |  |  | **With intermittent** | **With continuous** |  |  |
| **Mortality** | | | | | | | | | | |
| 458 (5 RCTs) | not serious | not serious | not serious | serious^a^ | none | ⨁⨁⨁◯ Moderate | 63/229 (27.5%) | 50/229 (21.8%) | **RR 0.80** (0.60 to 1.08) | **55 fewer per 1,000** (from 110 fewer to 22 more) |
| **Length of ICU stay** | | | | | | | | | | |
| 309 (3 RCTs) | serious^b^ | not serious | not serious | very serious^c^ | none | ⨁◯◯◯ Very low | 155 | 154 | - | **MD 0.8 shorter** (4.17 shorter to 2.56 longer) |
| **Duration of mechanical ventilation** | | | | | | | | | | |
| 161 (2 RCTs) | serious^b^ | not serious | not serious | serious^d^ | none | ⨁⨁◯◯ Low | 82 | 79 | - | **MD 2.26 shorter** (5.39 shorter to 0.88 longer) |
| **Infectious complications** | | | | | | | | | | |
| 267 (2 RCTs) | serious^b^ | very serious^e^ | not serious | very serious^f^ | none | ⨁◯◯◯ Very low | 46/135 (34.1%) | 59/132 (44.7%) | **RR 1.69** (0.40 to 7.17) | **235 more per 1,000** (from 204 fewer to 1,000 more) |
| **Diarrhea** | | | | | | | | | | |
| 385 (6 RCTs) | serious^b^ | not serious | not serious | serious^g^ | none | ⨁⨁◯◯ Low | 40/191 (20.9%) | 29/194 (14.9%) | **RR 0.81** (0.55 to 1.19) | **40 fewer per 1,000** (from 94 fewer to 40 more) |
| **Muscle mass loss** | | | | | | | | | | |
| 121 (1 RCT) | not serious | not serious | not serious | very serious^h^ | none | ⨁⨁◯◯ Low | 62 | 59 | - | **MD 1.9 higher** (13.3 lower to 17.1 higher) |
| **Vomiting** | | | | | | | | | | |
| 260 (3 RCTs) | serious^b^ | not serious | not serious | very serious^i^ | none | ⨁◯◯◯ Very low | 14/131 (10.7%) | 23/129 (17.8%) | **RR 1.40** (0.42 to 4.66) | **43 more per 1,000** (from 62 fewer to 391 more) |

**CI:** confidence interval; **MD:** mean difference; **RR:** risk ratio

a. Downgraded one level: confidence interval for effect estimate on mortality includes null and considerable harm.

b. Downgraded one level: none of the randomized trials blinded the caretakers.

c. Downgraded two levels: confidence interval for effect estimate on the length of ICU stay includes considerable benefit and considerable harm.

d. Downgraded one level: confidence interval for effect estimate on the duration of mechanical ventilation includes null and considerable benefit.

e. Downgraded two levels due to inconsistency by confidence interval overlap, difference in point estimate, and heterogeneity.

f. Downgraded two levels: confidence interval for effect estimate on infectious complications includes considerable benefit and considerable harm.

g. Downgraded one level: confidence interval for effect estimate on the duration of mechanical ventilation includes null and considerable benefit.

h. Downgraded two levels: confidence interval for effect estimate on muscle mass loss includes considerable benefit and considerable harm.

i. Downgraded two levels: confidence interval for effect estimate on vomiting includes considerable benefit and considerable harm.
