# Supplementary material for: The Japanese Critical Care Nutrition Guideline 2024
Source: J Intensive Care. 2025 Mar 21;13:18. doi: 10.1186/s40560-025-00785-z (PMC11927338; doi:10.1186/s40560-025-00785-z)
Supplement: Supplementary file 2 — Additional file 2: CQ2 Evidence profiles. [file 40560_2025_785_MOESM2_ESM.docx]

**Table 1. CQ2-1 Evidence profile**

| **Certainty assessment** | | | | | | | **Summary of findings** | | | |
| --- | --- | --- | --- | --- | --- | --- | --- | --- | --- | --- |
| **Participants (studies)** | **Risk of bias** | **Inconsistency** | **Indirectness** | **Imprecision** | **Publication bias** | **Overall certainty of evidence** | **Study event rates (%)** | | **Relative effect (95% CI)** | **Anticipated absolute effects**  **(95% CI)** |
|  |  |  |  |  |  |  | **With control** | **With omega-3 fatty acids** |  |  |
| **Short term mortality** | | | | | | | | | | |
| 4717 (33 RCTs) | serious^a^ | not serious | serious^b^ | not serious | none | ⨁⨁◯◯ Low | 539/2342 (23.0%) | 522/2375 (22.0%) | **RR 0.96** (0.87 to 1.07) | **9 fewer per 1,000** (from 30 fewer to 16 more) |
| **Length of ICU stay** | | | | | | | | | | |
| 3875 (30 RCTs) | serious^a^ | serious^c^ | serious^b^ | not serious | none | ⨁◯◯◯ Very low | 1938 | 1937 | - | **MD 1.98 shorter** (3.16 shorter to 0.8 shorter) |
| **Infectious complication rate** | | | | | | | | | | |
| 2224 (15 RCTs) | serious^a^ | serious^c^ | serious^b^ | not serious | none | ⨁◯◯◯ Very low | 523/1128 (46.4%) | 474/1096 (43.2%) | **RR 0.91** (0.76 to 1.09) | **42 fewer per 1,000** (from 111 fewer to 42 more) |
| **Ventilator days** | | | | | | | | | | |
| 3198 (26 RCTs) | serious^a^ | serious^c^ | serious^b^ | not serious | none | ⨁◯◯◯ Very low | 1613 | 1585 | - | **MD 1.77 shorter** (3.12 shorter to 0.41 shorter) |
| **HR-QoL** | | | | | | | | | | |
| 216 (2 RCTs) | very serious^d^ | not serious | very serious^b,e^ | serious^f^ | none | ⨁◯◯◯ Very low | 111 | 105 | - | **SMD 0.07 higher** (0.19 lower to 0.34 higher) |
| **Total adverse events** | | | | | | | | | | |
| 1691 (8 RCTs) | serious^a^ | not serious | not serious | not serious | none | ⨁⨁⨁◯ Moderate | 467/838 (55.7%) | 476/853 (55.8%) | **RR 1.00** (0.92 to 1.08) | **0 fewer per 1,000** (from 45 fewer to 45 more) |
| **Muscle volume** | | | | | | | | | | |
| 171 (2 RCTs) | very serious^d^ | serious^c^ | serious^b^ | serious^f^ | none | ⨁◯◯◯ Very low | 81 | 90 | - | **SMD 0.17 higher** (0.55 lower to 0.89 higher) |

**CI:** confidence interval; **HR-QoL:** health-related quality of life; **MD:** mean difference; **RR:** risk ratio; **SMD:** standardized mean difference

a. Downgraded one level due to bias from deviations from intended interventions.

b. Downgraded one level because some studies did not focus on protein enhancement or high-calorie intake, raising the possibility that the effects of ω-3 enhancement are not involved.

c. Downgraded one level due to substantial heterogeneity among the studies, but with overlapping confidence intervals.

d. Downgraded two levels due to bias from deviations from intended interventions and a contribution rate of over 40%.

e. Downgraded one level due to variations in the duration of the outcome.

f. Downgraded one level: optimal information size (OIS) is not met.

**Table 2. CQ2-1 Evidence profile** (Sensitivity analysis focused on studies intending to administer omega-3 fatty acids enriched enteral nutrition)

| **Certainty assessment** | | | | | | | **Summary of findings** | | | |
| --- | --- | --- | --- | --- | --- | --- | --- | --- | --- | --- |
| **Participants (studies)** | **Risk of bias** | **Inconsistency** | **Indirectness** | **Imprecision** | **Publication bias** | **Overall certainty of evidence** | **Study event rates (%)** | | **Relative effect (95% CI)** | **Anticipated absolute effects**  **(95% CI)** |
|  |  |  |  |  |  |  | **With control** | **With omega-3** **fatty acids** |  |  |
| **Short term mortality - omega-3** fatty acids **enriched enteral nutrition** | | | | | | | | | | |
| 3700 (25 RCTs) | serious^a^ | not serious | not serious | not serious | none | ⨁⨁⨁◯ Moderate | 416/1827 (22.8%) | 422/1873 (22.5%) | **RR 1.00** (0.90 to 1.12) | **0 fewer per 1,000** (from 23 fewer to 27 more) |
| **Length of ICU stay - omega-3** fatty acids **enriched enteral nutrition** | | | | | | | | | | |
| 2869 (22 RCTs) | serious^a^ | serious^b^ | not serious | not serious | none | ⨁⨁◯◯ Low | 1426 | 1443 | - | **MD 2.58 shorter** (3.98 shorter to 1.18 shorter) |
| **Infectious complication rate - omega-3** fatty acids **enriched enteral nutrition** | | | | | | | | | | |
| 1822 (11 RCTs) | serious^a^ | serious^b^ | not serious | serious^c^ | none | ⨁◯◯◯ Very low | 425/905 (47.0%) | 403/917 (43.9%) | **RR 0.89** (0.72 to 1.10) | **52 fewer per 1,000** (from 131 fewer to 47 more) |
| **Ventilator days - omega-3** fatty acids **enriched enteral nutrition** | | | | | | | | | | |
| 2479 (20 RCTs) | serious^a^ | serious^b^ | not serious | not serious | none | ⨁⨁◯◯ Low | 1236 | 1243 | - | **MD 2.22 shorter** (3.77 shorter to 0.66 shorter) |
| **HR-QoL- omega-3** fatty acids **enriched enteral nutrition** | | | | | | | | | | |
| 99 (1 RCT) | very serious^d^ | not serious | not serious | serious^c^ | none | ⨁◯◯◯ Very low | 54 | 45 | - | **MD 2.6 higher** (3.62 lower to 8.82 higher) |
| **Total adverse events- omega-3** fatty acids **enriched enteral nutrition** | | | | | | | | | | |
| 1691 (8 RCTs) | serious^a^ | not serious | not serious | not serious | none | ⨁⨁⨁◯ Moderate | 467/838 (55.7%) | 476/853 (55.8%) | **RR 1.00** (0.92 to 1.08) | **0 fewer per 1,000** (from 45 fewer to 45 more) |
| **Muscle volume- omega-3** fatty acids **enriched enteral nutrition** | | | | | | | | | | |
| 54 (1 RCT) | very serious^d^ | not serious | not serious | serious^c^ | none | ⨁◯◯◯ Very low | 24 | 30 | - | **MD 0.05 lower** (0.18 lower to 0.07 higher) |

**CI:** confidence interval; **HR-QoL:** health-related quality of life; **MD:** mean difference; **RR:** risk ratio

a. Downgraded one level due to bias from deviations from intended interventions.

b. Downgraded one level due to substantial heterogeneity among the studies, but with overlapping confidence intervals.

c. Downgraded one level: optimal information size (OIS) is not met.

d. Downgraded two levels due to bias from deviations from intended interventions and a contribution rate of over 40%.

**Table 3. CQ2-1 Evidence profile** (Sensitivity analysis focused on studies administering bolus omega-3 fatty acids enriched enteral nutrition)

| **Certainty assessment** | | | | | | | **Summary of findings** | | | |
| --- | --- | --- | --- | --- | --- | --- | --- | --- | --- | --- |
| **Participants (studies) Follow-up** | **Risk of bias** | **Inconsistency** | **Indirectness** | **Imprecision** | **Publication bias** | **Overall certainty of evidence** | **Study event rates (%)** | | **Relative effect (95% CI)** | **Anticipated absolute effects**  **(95% CI)** |
|  |  |  |  |  |  |  | **With control** | **With omega-3 fatty acids** |  |  |
| **Short term mortality_bolus** | | | | | | | | | | |
| 332 (3 RCTs) | not serious | not serious | not serious | serious^a^ | none | ⨁⨁⨁◯ Moderate | 29/170 (17.1%) | 21/162 (13.0%) | **RR 0.80** (0.45 to 1.44) | **34 fewer per 1,000** (from 94 fewer to 75 more) |
| **Infections complication_bolus** | | | | | | | | | | |
| 0 (0 RCTs) |  |  |  |  | none | - | 0/0 | 0/0 | not pooled | not pooled |
| **Length of ICU stay_bolus** | | | | | | | | | | |
| 244 (2 RCTs) | not serious | not serious | not serious | serious^a^ | none | ⨁⨁⨁◯ Moderate | 122 | 122 | - | **MD 2.37 shorter** (3.85 shorter to 0.89 shorter) |
| **Muscle volume_** **bolus** | | | | | | | | | | |
| 54 (1 RCT) | very serious^b^ | not serious | not serious | serious^a^ | none | ⨁◯◯◯ Very low^b^ | 24 | 30 | - | **MD 0.05 lower** (0.18 lower to 0.07 higher) |
| **HR QOL_bolus** | | | | | | | | | | |
| 0 (0 RCTs) |  |  |  |  |  | - | -/0 | -/0 | not pooled | not pooled |
| **Ventilator days_** **bolus** | | | | | | | | | | |
| 237 (2 RCTs) | not serious | serious^c^ | not serious | not serious | none | ⨁⨁⨁◯ Moderate | 120 | 117 | - | **MD 1.36 shorter** (4.78 shorter to 2.07 longer) |
| **Ventilator free days_** **bolus** | | | | | | | | | | |
| 361 (2 RCTs) | not serious | serious^c^ | not serious | not serious | none | ⨁⨁⨁◯ Moderate | 177 | 184 | - | **MD 1.51 shorter** (5.47 shorter to 2.44 longer) |
| **Total adverse events_** **bolus** | | | | | | | | | | |
| 463 (2 RCTs) | serious^d^ | not serious | not serious | not serious | none | ⨁⨁⨁◯ Moderate | 105/230 (45.7%) | 109/233 (46.8%) | **RR 1.88** (0.24 to 14.68) | **402 more per 1,000** (from 347 fewer to 1,000 more) |

**CI:** confidence interval; **HR-QoL:** health-related quality of life; **MD:** mean difference; **RR:** risk ratio

a. Downgraded one level: optimal information size (OIS) is not met.

b. Downgraded two levels due to high risk of bias and a contribution rate of more than 40%.

c. Downgraded one level due to substantial heterogeneity among the studies, but with overlapping confidence intervals.

d. Downgraded one level due to high risk of bias and a contribution rate of less than 40%

**Table 4. CQ2-2 Evidence profile**

| **Certainty assessment** | | | | | | | **Summary of findings** | | | |
| --- | --- | --- | --- | --- | --- | --- | --- | --- | --- | --- |
| **Participants (studies)** | **Risk of bias** | **Inconsistency** | **Indirectness** | **Imprecision** | **Publication bias** | **Overall certainty of evidence** | **Study event rates (%)** | | **Relative effect (95% CI)** | **Anticipated absolute effects**  **(95% CI)** |
|  |  |  |  |  |  |  | **With placebo/control** | **With glutamine** |  |  |
| **Mortality (hospital, 30day,180day)** | | | | | | | | | | |
| 3195 (18 RCTs) | not serious | not serious | not serious | not serious | publication bias strongly suspected^a^ | ⨁⨁⨁◯ Moderate | 515/1593 (32.3%) | 516/1602 (32.2%) | **RR 1.01** (0.91 to 1.11) | **3 more per 1,000** (from 29 fewer to 36 more) |
| **Length of ICU stay** | | | | | | | | | | |
| 3034 (17 RCTs) | serious^b^ | very serious^c^ | not serious | serious^d^ | publication bias strongly suspected^a^ | ⨁◯◯◯ Very low | 1511 | 1523 | - | **MD 1.82 longer** (0.07 shorter to 3.7 longer) |
| **Duration of Mechanical ventilation** | | | | | | | | | | |
| 1990 (11 RCTs) | serious^b^ | serious^e^ | not serious | serious^f^ | none | ⨁◯◯◯ Very low | 989 | 1001 | - | **MD 0.48 longer** (0.51 shorter to 1.47 longer) |
| **Infectious Complication** | | | | | | | | | | |
| 1197 (8 RCTs) | not serious | serious^g^ | not serious | not serious | none | ⨁⨁⨁◯ Moderate | 277/590 (46.9%) | 288/607 (47.4%) | **RR 0.95** (0.78 to 1.16) | **23 fewer per 1,000** (from 103 fewer to 75 more) |
| **Diarrhea** | | | | | | | | | | |
| 799 (6 RCTs) | serious^h^ | not serious | not serious | serious^i^ | none | ⨁⨁◯◯ Low | 49/402 (12.2%) | 65/397 (16.4%) | **RR 1.46** (1.08 to 1.97) | **56 more per 1,000** (from 10 more to 118 more) |
| **Adverse Events** | | | | | | | | | | |
| 1825 (3 RCTs) | not serious | serious^j^ | not serious | very serious^k^ | none | ⨁◯◯◯ Very low | 265/913 (29.0%) | 270/912 (29.6%) | **RR 0.93** (0.69 to 1.27) | **20 fewer per 1,000** (from 90 fewer to 78 more) |

**CI:** confidence interval; **MD:** mean difference; **OR:** odds ratio; **RR:** risk ratio

a. Downgraded one level due to visual assessment of the funnel plot, which indicated the presence of publication bias.

b. Downgraded due to concerns about bias in the majority of Domain 5 and a high risk of bias with a weight of over 20% in Domain 2, resulting in significant bias.

c. Downgraded two levels due to very serious inconsistency, as indicated by I² = 71% and visual assessment of variability of effects through the forest plot.

d. Downgraded one level: confidence interval for effect estimate includes null and considerable harm, despite the total sample size of 3034 meeting the optimal information size (OIS).

e. Downgraded one level due to serious non-consistency, as indicated by I² = 42% and visual assessment of variability of effects through the forest plot, despite clinical heterogeneity being determined as mild.

f. Downgraded one level: confidence interval for effect estimate includes null and considerable harm, despite the total sample size of 1990 meeting the optimal information size (OIS).

g. Downgraded one level due to serious non-consistency, as indicated by I² = 48% and visual assessment of variability of effects through the forest plot, despite clinical heterogeneity being determined as mild.

h. Downgraded due to concerns about bias in the majority of Domain 5 and high risk of bias with a weight of around 20% in Domain 3, resulting in significant bias.

i. Downgraded one level: total sample size of 799 and event count of 114 do not meet the optimal information size (OIS).

j. Downgraded one level due to serious non-consistency, as indicated by I² = 39% and visual assessment of variability of effects through the forest plot, despite clinical heterogeneity being determined as mild.

k. Downgraded two levels: confidence interval for effect estimate includes considerable benefit and considerable harm, despite a total sample size of 1825 and an event count of 535 meeting the optimal information size (OIS).

**Table 5. CQ2-3 Evidence profile**

| **Certainty assessment** | | | | | | | **Summary of findings** | | | |
| --- | --- | --- | --- | --- | --- | --- | --- | --- | --- | --- |
| **Participants (studies)** | **Risk of bias** | **Inconsistency** | **Indirectness** | **Imprecision** | **Publication bias** | **Overall certainty of evidence** | **Study event rates (%)** | | **Relative effect (95% CI)** | **Anticipated absolute effects**  **(95% CI)** |
|  |  |  |  |  |  |  | **With polymeric formula** | **With elemental / oligomeric** |  |  |
| **Mortality** | | | | | | | | | | |
| 1021 (12 RCTs) | very serious^a^ | serious^b^ | not serious | very serious^c^ | None | ⨁◯◯◯ Very low | 92/539 (17.1%) | 83/482 (17.2%) | **RR 1.00** (0.77 to 1.31) | **0 more per 1,000** (from 40 fewer to 53 more) |
| **Length of ICU stay** | | | | | | | | | | |
| 669 (9 RCTs) | very serious^a^ | not serious | not serious | serious^d^ | None | ⨁◯◯◯ Very low | 332 | 337 | - | **MD 1.24 shorter** (2.39 shorter to 0.08 shorter) |
| **Duration of mechanical ventilation** | | | | | | | | | | |
| 486 (5 RCTs) | very serious^a^ | not serious | not serious | serious^d^ | None | ⨁◯◯◯ Very low | 238 | 248 | - | **MD 0.62 shorter** (1.44 shorter to 0.2 shorter) |
| **Infectious complication** | | | | | | | | | | |
| 966 (10 RCTs) | very serious^a^ | not serious | not serious | serious^d^ | None | ⨁◯◯◯ Very low | 229/507 (45.2%) | 204/459 (44.4%) | **RR 1.06** (0.88 to 1.28) | **27 more per 1,000** (from 54 fewer to 126 more) |
| **Diarrhea** | | | | | | | | | | |
| 909 (12 RCTs) | very serious^a^ | serious^e^ | not serious | serious^f^ | None | ⨁◯◯◯ Very low | 167/481 (34.7%) | 136/428 (31.8%) | **RR 0.98** (0.71 to 1.34) | **6 fewer per 1,000** (from 92 fewer to 108 more) |

**CI:** confidence interval; **MD:** mean difference; **RR:** risk ratio

a. Downgraded two levels due to overall high risk of bias.

b. Downgraded one level due to inconsistency, caused by differing directions of effect size and high heterogeneity.

c. Downgraded two levels: confidence interval for effect estimate includes considerable benefit and considerable harm, due to the wide range of the 95% CI.

d. Downgraded one level: total sample size is not large enough to assess adverse events and does not meet optimal information size (OIS).

e. Downgraded one level due to serious inconsistency, with risk ratios ranging from 0.66 to 1.30 and I² = 57%.

f. Downgraded one level: total sample size is not large enough to assess adverse events and does not meet optimal information size (OIS).

**Table 6. CQ2-4 Evidence profile**

| **Certainty assessment** | | | | | | | **Summary of findings** | | | |
| --- | --- | --- | --- | --- | --- | --- | --- | --- | --- | --- |
| **Participants (studies)** | **Risk of bias** | **Inconsistency** | **Indirectness** | **Imprecision** | **Publication bias** | **Overall certainty of evidence** | **Study event rates (%)** | | **Relative effect (95% CI)** | **Anticipated absolute effects**  **(95% CI)** |
|  |  |  |  |  |  |  | **With placebo** | **With arginine** |  |  |
| **Mortality** | | | | | | | | | | |
| 1965 (15 RCTs) | not serious | not serious | not serious | not serious | None | ⨁⨁⨁⨁ High | 266/953 (27.9%) | 305/1012 (30.1%) | **RR 1.07** (0.94 to 1.22) | **20 more per 1,000** (from 17 fewer to 61 more) |
| **Length of ICU Stay** | | | | | | | | | | |
| 1333 (11 RCTs) | not serious | very serious^a^ | not serious | very serious^b^ | None | ⨁◯◯◯ Very low | 647 | 686 | - | **MD 0.52 days longer** (1.65 shorter to 2.68 longer) |
| **Infectious Complication** | | | | | | | | | | |
| 840 (4 RCTs) | serious^c^ | serious^d^ | not serious | very serious^e^ | None | ⨁◯◯◯ Very low | 158/414 (38.2%) | 162/426 (38.0%) | **RR 0.98** (0.63 to 1.54) | **8 fewer per 1,000** (from 141 fewer to 206 more) |
| **Duration of Mechanical Ventilation** | | | | | | | | | | |
| 1326 (11 RCTs) | serious^c^ | very serious^f^ | not serious | very serious^g^ | publication bias strongly suspected^h^ | ⨁◯◯◯ Very low | 641 | 685 | - | **MD 0.23 days shorter** (1.59 shorter to 1.13 longer) |
| **Physical Function** | | | | | | | | | | |
| 0 (0 studies) |  |  |  |  |  | - | 0 | 0 | - | not pooled |
| **Rate of Muscle Mass Loss** | | | | | | | | | | |
| 50 (1 RCT) | not serious | not serious | not serious | very serious^i^ | None | ⨁⨁◯◯ Low | 24 | 26 | - | **MD 3% lower** (7.3 lower to 1.3 higher) |
| **Adverse Events** | | | | | | | | | | |
| 86 (2 RCTs) | not serious | not serious | not serious | very serious^j^ | None | ⨁⨁◯◯ Low | 14/43 (32.6%) | 11/43 (25.6%) | **RR 0.89** (0.52 to 1.51) | **36 fewer per 1,000** (from 156 fewer to 166 more) |

**CI:** confidence interval; **MD:** mean difference; **RR:** risk ratio

a. Downgraded two levels due to very serious heterogeneity, as indicated by I² = 85% and visual assessment of variability of effects in the forest plot.

b. Downgraded two levels: confidence interval for effect estimate includes considerable benefit and considerable harm, despite the total sample size of 1333 meeting the optimal information size (OIS).

c. Downgraded one level due to the presence of high risk of bias in many studies.

d. Downgraded one level due to serious non-consistency, despite I² = 73%, as clinical heterogeneity was considered mild based on visual assessment of effect variability in the forest plot.

e. Downgraded two levels: confidence interval for effect estimate includes considerable benefit and considerable harm, despite the total sample size of 840 and event count of 320 meeting the optimal information size (OIS).

f. Downgraded two levels due to very serious heterogeneity, as indicated by I² = 77% and visual assessment of variability of effects in the forest plot.

g. Downgraded two levels: confidence interval for effect estimate includes considerable benefit and considerable harm, despite the total sample size of 1326 meeting the optimal information size (OIS).

h. Downgraded one level due to strong suspicion of publication bias, based on visual assessment of variability of effects in funnel plots for each study.

i. Downgraded two levels: confidence interval for effect estimate includes null and considerable benefit, with a very small total sample size of 50.

j. Downgraded two levels: confidence interval for effect estimate includes considerable benefit and considerable harm, with a very small total sample size of 86.

**Table 7. CQ2-5 Evidence profile**

| **Certainty assessment** | | | | | | | | **Summary of findings** | | | |
| --- | --- | --- | --- | --- | --- | --- | --- | --- | --- | --- | --- |
| **Participants (studies)** | **Risk of bias** | **Inconsistency** | **Indirectness** | **Imprecision** | **Publication bias** | **Overall certainty of evidence** | **Study event rates (%)** | | | **Relative effect (95% CI)** | **Anticipated absolute effects**  **(95% CI)** |
|  |  |  |  |  |  |  | **With control** | | **With HFLC** |  |  |
| **30-day mortality** | | | | | | | | | | | |
| 487 (6 RCTs) | serious^a^ | not serious | not serious | serious^b^ | none | ⨁⨁◯◯ Low | 39/207 (18.8%) | | 62/280 (22.1%) | **RR 1.14** (0.80 to 1.62) | **26 more per 1,000** (from 38 fewer to 117 more) |
| **Length of ICU stay** | | | | | | | | | | | |
| 299 (3 RCTs) | not serious | not serious | not serious | not serious | none | ⨁⨁⨁⨁ High | 122 | | 177 | - | **MD 0.16 days shorter** (1.94 shorter to 1.61 shorter) |
| **Duration of mechanical ventilation** | | | | | | | | | | | |
| 318 (4 RCTs) | serious^c^ | serious^d^ | not serious | not serious | none | ⨁⨁◯◯ Low | 134 | | 184 | - | **MD 1.73 days shorter** (2.94 shorter to 0.53 shorter) |
| **Diarrhea** | | | | | | | | | | | |
| 149 (3 RCTs) | serious^e^ | not serious | not serious | serious^f^ | none | ⨁⨁◯◯ Low | 32/66 (48.5%) | | 29/83 (34.9%) | **RR 0.84** (0.65 to 1.08) | **78 fewer per 1,000** (from 170 fewer to 39 more) |
| **GRV** | | | | | | | | | | | |
| 149 (3 RCTs) | serious^g^ | not serious | not serious | serious^h^ | none | ⨁⨁◯◯ Low | 27/66 (40.9%) | | 30/83 (36.1%) | **RR 0.97** (0.68 to 1.38) | **12 fewer per 1,000** (from 131 fewer to 155 more) |

**CI:** confidence interval; **GRV:** gastric residual volume; **HFLC:** high-fat, low-carbohydrate; **MD:** mean difference; **RR:** risk ratio

a. Downgraded one level: high risk of bias due to two randomized trials inappropriately excluding patients after randomization and three not reporting any trial protocol.

b. Downgraded one level: total sample size less than 2000 and does not meet optimal information size (OIS) criteria (calculated with α = 0.05, β = 0.2, event = 20%, and relative risk reduction = 25%).

c. Downgraded one level for risk of bias because, out of four randomized trials, two did not report the randomization process, one did not perform intention-to-treat analysis, and two did not report any trial protocol.

d. Downgraded one level for inconsistency due to statistically significant inconsistency observed (I² = 72% with P = 0.008).

e. Downgraded one level for risk of bias because, out of three randomized trials, one inappropriately excluded patients after randomization, one did not define diarrhea, and two did not report any trial protocol.

f. Downgraded one level: total sample size less than 800 and does not meet optimal information size (OIS) criteria (calculated with α = 0.05, β = 0.2, event = 40%, and relative risk reduction = 25%).

g. Downgraded one level for risk of bias because, out of three randomized trials, one inappropriately excluded patients after randomization, one did not define GRV, and two did not report any trial protocol.

h. Downgraded one level: total sample size less than 800 and does not meet optimal information size (OIS) criteria (calculated with α = 0.05, β = 0.2, event = 40%, and relative risk reduction = 25%).

**Table 8. CQ2-6-1 Evidence profile**

| **Certainty assessment** | | | | | | | **Summary of findings** | | | |
| --- | --- | --- | --- | --- | --- | --- | --- | --- | --- | --- |
| **Participants (studies)** | **Risk of bias** | **Inconsistency** | **Indirectness** | **Imprecision** | **Publication bias** | **Overall certainty of evidence** | **Study event rates (%)** | | **Relative effect (95% CI)** | **Anticipated absolute effects**  **(95% CI)** |
|  |  |  |  |  |  |  | **With control** | **With lipid emulsion** |  |  |
| **Hospital mortality** | | | | | | | | | | |
| 345 (7 RCTs) | not serious | not serious | not serious | very serious^a^ | None | ⨁⨁◯◯ Low | 41/168 (24.4%) | 41/177 (23.2%) | **RR 0.91** (0.55 to 1.52) | **22 fewer per 1,000** (from 110 fewer to 127 more) |
| **Length of ICU stay** | | | | | | | | | | |
| 324 (6 RCTs) | not serious | not serious | not serious | very serious^a^ | None | ⨁⨁◯◯ Low | 161 | 163 | - | **MD 0.41 shorter** (4.83 shorter to 4.01 longer) |
| **Duration of mechanical ventilation** | | | | | | | | | | |
| 117 (2 RCTs) | not serious | not serious | not serious | very serious^a^ | None | ⨁⨁◯◯ Low | 57 | 60 | - | **MD 5.79 longer** (4.56 shorter to 16.14 longer) |
| **Infectious complication** | | | | | | | | | | |
| 180 (3 RCTs) | not serious | not serious | not serious | serious^b^ | None | ⨁⨁⨁◯ Moderate | 30/91 (33.0%) | 25/89 (28.1%) | **RR 0.90** (0.42 to 1.94) | **33 fewer per 1,000** (from 191 fewer to 310 more) |
| **All adverse events** | | | | | | | | | | |
| 60 (1 RCT) | not serious | not serious | not serious | very serious^c^ | None | ⨁⨁◯◯ Low | 6/30 (20.0%) | 1/30 (3.3%) | **RR 0.17** (0.02 to 1.30) | **166 fewer per 1,000** (from 196 fewer to 60 more) |

**CI:** confidence interval; **MD:** mean difference; **RR:** risk ratio

a. Downgraded two levels: confidence interval for effect estimate includes considerable benefit and considerable harm, with a small number of cases.

b. Downgraded one level: confidence interval for effect estimate includes null and considerable benefit, due to wide confidence intervals.

c. Downgraded two levels because there is only one RCT

**Table 9. CQ2-6 Evidence profile (Sensitivity analysis of intravenous lipid emulsion administration, divided into omega-3 fatty acids and non-omega-3 fatty acids.)**

| **Certainty assessment** | | | | | | | **Summary of findings** | | | |
| --- | --- | --- | --- | --- | --- | --- | --- | --- | --- | --- |
| **Participants (studies) Follow-up** | **Risk of bias** | **Inconsistency** | **Indirectness** | **Imprecision** | **Publication bias** | **Overall certainty of evidence** | **Study event rates (%)** | | **Relative effect (95% CI)** | **Anticipated absolute effects**  **(95% CI)** |
|  |  |  |  |  |  |  | **With control** | **With lipid emulsion** |  |  |
| **Hospital mortality - omega-3 fatty acids** | | | | | | | | | | |
| 227 (4 RCTs) | not serious | not serious | not serious | very serious^a^ | none | ⨁⨁◯◯ Low | 34/113 (30.1%) | 29/114 (25.4%) | **RR 0.74** (0.35 to 1.58) | **78 fewer per 1,000** (from 196 fewer to 175 more) |
| **Hospital mortality - non_omega-3 fatty acids** | | | | | | | | | | |
| 118 (3 RCTs) | not serious | not serious | not serious | very serious^a^ | none | ⨁⨁◯◯ Low | 7/55 (12.7%) | 12/63 (19.0%) | **RR 1.32** (0.60 to 2.92) | **41 more per 1,000** (from 51 fewer to 244 more) |
| **Length of ICU stay - omega-3 fatty acids** | | | | | | | | | | |
| 227 (4 RCTs) | not serious | not serious | not serious | very serious^a^ | none | ⨁⨁◯◯ Low | 113 | 114 | - | **MD 2.43 shorter** (6.23 shorter to 1.36 longer) |
| **Length of ICU stay - non_omega-3 fatty acids** | | | | | | | | | | |
| 97 (2 RCTs) | not serious | not serious | not serious | very serious^a^ | none | ⨁⨁◯◯ Low | 48 | 49 | - | **MD 4.49 longer** (8.25 shorter to 17.23 longer) |

**CI:** confidence interval; **MD:** mean difference; **RR:** risk ratio

a. Downgraded two levels: confidence interval for effect estimate includes considerable benefit and considerable harm, with a small number of cases.

**Additional table 10. CQ2-7-1 Evidence profile**

| **Certainty assessment** | | | | | | | **Summary of findings** | | | |
| --- | --- | --- | --- | --- | --- | --- | --- | --- | --- | --- |
| **Participants (studies)** | **Risk of bias** | **Inconsistency** | **Indirectness** | **Imprecision** | **Publication bias** | **Overall certainty of evidence** | **Study event rates (%)** | | **Relative effect (95% CI)** | **Anticipated absolute effects**  **(95% CI)** |
|  |  |  |  |  |  |  | **With placebo** | **With prebiotics** |  |  |
| **In-hospital mortality** | | | | | | | | | | |
| 177 (4 RCTs) | not serious | not serious | not serious | serious^a^ | none | ⨁⨁⨁◯ Moderate | 29/88 (33.0%) | 17/89 (19.1%) | RR 0.61 (0.37 to 1.01) | **129 fewer per 1,000** (from 208 fewer to 3 more) |
| **Length of ICU stay** | | | | | | | | | | |
| 646 (10 RCTs) | not serious | not serious | not serious | serious^b^ | none | ⨁⨁⨁◯ Moderate | 316 | 330 | - | **MD 1.57 shorter** (3.32 shorter to 0.19 longer) |
| **Duration of mechanical ventilation** | | | | | | | | | | |
| 155 (3 RCTs) | serious^c^ | very serious^d^ | not serious | very serious^e^ | none | ⨁◯◯◯ Very low | 76 | 79 | - | **MD 3.2 shorter** (10.79 shorter to 4.39 longer) |
| **Prebiotics for infection control** | | | | | | | | | | |
| 479 (7 RCTs) | serious^f^ | not serious | serious^g^ | serious^h^ | none | ⨁◯◯◯ Very low | 64/239 (26.8%) | 58/240 (24.2%) | RR 0.85 (0.56 to 1.30) | **40 fewer per 1,000** (from 118 fewer to 80 more) |
| **Adverse events** | | | | | | | | | | |
| 964 (14 RCTs) | serious^i^ | serious^j^ | very serious^k^ | serious^l^ | none | ⨁◯◯◯ Very low | 215/477 (45.1%) | 156/487 (32.0%) | RR 0.67 (0.46 to 0.97) | **149 fewer per 1,000** (from 243 fewer to 14 fewer) |

**CI**: confidence interval; **MD**: mean difference; **RR**: risk ratio

a. Downgraded one level: confidence interval for effect estimate includes substantial gains.

b. Downgraded one level: total sample size is less than 800 and does not meet optimal information size (OIS).

c. Downgraded one level: high risk of reporting bias despite a large sample size.

d. Downgraded two levels due to I² = 88%, p = 0.0002.

e. Downgraded two levels: confidence interval for effect estimate includes both substantial benefit and harm.

f. Downgraded one level due to multiple high-risk biases.

g. Downgraded due to inconsistency in infectious complications such as VAP and CDI.

h. Downgraded one level: total sample size is less than 800 and does not meet optimal information size (OIS).

i. Downgraded due to multiple high-risk biases.

j. Downgraded one level due to I² = 87% and point estimate generally biased towards favoring one side.

k. Downgraded two levels due to varying outcomes (e.g., diarrhea, constipation, vomiting) applied in different studies.

l. Downgraded one level: total sample size is less than 800 and does not meet optimal information size (OIS).

**Additional table 11. CQ2-7-2 Evidence profile**

| Certainty assessment | | | | | | | Summary of findings | | | |
| --- | --- | --- | --- | --- | --- | --- | --- | --- | --- | --- |
| Participants (studies) | Risk of bias | Inconsistency | Indirectness | Imprecision | Publication bias | Overall certainty of evidence | Study event rates (%) | | Relative effect (95% CI) | Anticipated absolute effects  (95% CI) |
|  |  |  |  |  |  |  | With Placebo | With Probiotics |  |  |
| **In-hospital mortality** | | | | | | | | | | |
| 3307 (6 RCTs) | not serious | not serious | not serious | not serious | none | ⨁⨁⨁⨁ High | 435/1664 (26.1%) | 411/1643 (25.1%) | RR 0.96 (0.85 to 1.07) | **10 fewer per 1,000** (from 39 fewer to 18 more) |
| **Length of ICU stay** | | | | | | | | | | |
| 3534 (9 RCTs) | not serious | serious^a^ | not serious | not serious | none | ⨁⨁⨁◯ Moderate | 1773 | 1761 | - | **MD 2.43 shorter** (4.18 shorter to 0.67 longer) |
| **Duration of mechanical ventilation** | | | | | | | | | | |
| 3445 (7 RCTs) | not serious | serious^b^ | not serious | not serious | none | ⨁⨁⨁◯ Moderate | 1732 | 1713 | - | **MD 0.78. shorter** (1.76 shorter to 0.19 longer) |
| **Infectious complications** | | | | | | | | | | |
| 4272 (13 RCTs) | not serious | serious^c^ | not serious | serious^d^ | none | ⨁⨁◯◯ Low | 485/2142 (22.6%) | 429/2130 (20.1%) | RR 0.68 (0.50 to 0.94) | **72 fewer per 1,000** (from 113 fewer to 14 fewer) |
| **All adverse events** | | | | | | | | | | |
| 3112 (6 RCTs) | not serious | not serious | not serious | serious^e^ | none | ⨁⨁⨁◯ Moderate | 934/1563 (59.8%) | 939/1549 (60.6%) | RR 1.02  (0.85 to 1.24) | **12 more per 1,000** (from 90 fewer to 143 more) |
| **Physical function** | | | | | | | | | | |
| 207 (1 RCT) | not serious | not serious | not serious | not serious | none | ⨁⨁⨁⨁ High | 103 | 104 | - | **MD 1 lower**  (7.96 lower to 5.96 higher) |

**CI**: confidence interval; **MD**: mean difference; **RR**: risk ratio

a. Downgraded one level due to differing directions across studies and high heterogeneity with I² = 76%.

b. Downgraded one level due to I² = 81% and some studies showing a reduction in mechanical ventilation duration.

c. Downgraded one level due to differing directions across studies and high heterogeneity with I² = 65%.

d. Downgraded one level due to RD = -0.07 (-0.12 to 0.02). No further changes were made as the number of events was greater than 800.

e. Downgraded one level due to RD = -0.02 (-0.09 to 0.05). No further changes were made as the number of events was greater than 800.

**Additional table 12. CQ2-7-3 Evidence profile**

| Certainty assessment | | | | | | | Summary of findings | | | |
| --- | --- | --- | --- | --- | --- | --- | --- | --- | --- | --- |
| Participants (studies) | Risk of bias | Inconsistency | Indirectness | Imprecision | Publication bias | Overall certainty of evidence | Study event rates (%) | | Relative effect (95% CI) | Anticipated absolute effects  (95% CI) |
|  |  |  |  |  |  |  | With placebo | With  synbiotics |  |  |
| **In-hospital mortality** | | | | | | | | | | |
| 614 (6 RCTs) | serious^a^ | not serious | not serious | serious^b^ | none | ⨁⨁◯◯ Low | 78/307 (25.4%) | 75/307 (24.4%) | RR 0.96 (0.74 to 1.25) | **10 fewer per 1,000** (from 66 fewer to 64 more) |
| **Length of ICU stay** | | | | | | | | | | |
| 988  (12RCTs) | serious^c^ | serious^d^ | not serious | serious^e^ | none | ⨁◯◯◯ Very low | 487 | 501 | - | **MD 0.07 shorter** (0.87 shorter to 0.73 longer) |
| **Duration of mechanical ventilation** | | | | | | | | | | |
| 500 (5 RCTs) | serious^f^ | serious^g^ | not serious | serious^h^ | none | ⨁◯◯◯ Very low | 245 | 255 | - | **MD 1.56 shorter** (4.42 shorter to 1.31 longer) |
| **Prebiotics for infection control** | | | | | | | | | | |
| 858  (10RCTs) | very serious^i^ | serious^j^ | not serious | serious^k^ | none | ⨁◯◯◯ Very low | 184/421 (43.7%) | 110/437  (25.2%) | RR 0.50 (0.34 to 0.73) | **219 fewer per 1,000** (from 288 fewer to 118 fewer) |
| **Adverse events** | | | | | | | | | | |
| 416  (3RCTs) | serious^l^ | serious^m^ | not serious | very serious^n^ | none | ⨁◯◯◯ Very low | 19/207 (9.2%) | 15/209 (7.2%) | RR 0.74 (0.28 to 1.99) | **24 fewer per 1,000** (from 66 fewer to 91 more) |

**CI**: confidence interval; **MD**: mean difference; **RR**: risk ratio

a. Downgraded one level due to the presence of multiple studies with high risk in the randomization process and deviations from intended interventions.

b. Downgraded one level: optimal information size (OIS) is not met.

c. Downgraded one level due to overall data variability.

d. Downgraded one level due to I² = 57%.

e. Downgraded one level: confidence interval for effect estimate crosses zero.

f. Downgraded one level due to high risk from deviations from intended interventions.

g. Downgraded one level due to mixed results in mechanical ventilation duration, with I² = 77% indicating high heterogeneity.

h. Downgraded one level: small sample size does not meet the optimal information size (OIS) criteria.

i. Downgraded one level due to overall data variability.

j. Downgraded one level due to I² = 82%.

k. Downgraded one level because the confidence interval crosses zero.

l. Downgraded one level due to overall data variability.

m. Downgraded one level due to I² = 68%.

n. Downgraded one level because the confidence interval crosses zero.
