# Supplementary material for: The Japanese Critical Care Nutrition Guideline 2024
Source: J Intensive Care. 2025 Mar 21;13:18. doi: 10.1186/s40560-025-00785-z (PMC11927338; doi:10.1186/s40560-025-00785-z)
Supplement: Supplementary file 3 — Additional file 3: CQ3 Evidence profiles. [file 40560_2025_785_MOESM3_ESM.docx]

**Table 1. CQ3-2 Evidence profile**

| **Certainty assessment** | | | | | | | **Summary of findings** | | | |
| --- | --- | --- | --- | --- | --- | --- | --- | --- | --- | --- |
| **Participants (studies)** | **Risk of bias** | **Inconsistency** | **Indirectness** | **Imprecision** | **Publication bias** | **Overall certainty of evidence** | **Study event rates (%)** | | **Relative effect (95% CI)** | **Anticipated absolute effects (95% CI)** |
|  |  |  |  |  |  |  | **With Equation** | **With IC** |  |  |
| **Short-term Mortality** | | | | | | | | | | |
| 988 (7 RCTs) | not serious | not serious | not serious | serious^a^ | None | ⨁⨁⨁◯ Moderate | 128/498 (25.7%) | 107/490 (21.8%) | **RR 0.86** (0.70 to 1.06) | **36 fewer per 1,000** (from 77 fewer to 15 more) |
| **Length of ICU stay** | | | | | | | | | | |
| 1090 (7 RCTs) | not serious | serious^b^ | not serious | serious^c^ | None | ⨁⨁◯◯ Low | 550 | 540 | - | **MD 0.86 longer** (0.98 shorter to 2.7 longer) |
| **Duration of mechanical ventilation** | | | | | | | | | | |
| 1068 (7 RCTs) | not serious | not serious | not serious | serious^c^ | None | ⨁⨁⨁◯ Moderate | 539 | 529 | - | **MD 0.66 longer** (0.39 shorter to 1.72 longer) |
| **Total infections** | | | | | | | | | | |
| 785 (4 RCTs) | not serious | very serious^d^ | not serious | serious^a^ | None | ⨁◯◯◯ Very low | 88/399 (22.1%) | 90/386 (23.3%) | **RR 1.06** (0.82 to 1.37) | **13 more per 1,000** (from 40 fewer to 82 more) |
| **Pneumonia (included ventilator associated pneumonia)** | | | | | | | | | | |
| 785 (4 RCTs) | not serious | serious^b^ | not serious | serious^a^ | None | ⨁⨁◯◯ Low | 45/399 (11.3%) | 44/386 (11.4%) | **RR 1.02** (0.69 to 1.51) | **2 more per 1,000** (from 35 fewer to 58 more) |
| **Adverse event（kidney）** | | | | | | | | | | |
| 421 (2 RCTs) | not serious | not serious | not serious | serious^a^ | None | ⨁⨁⨁◯ Moderate | 65/209 (31.1%) | 68/212 (32.1%) | **RR 1.03** (0.78 to 1.36) | **9 more per 1,000** (from 68 fewer to 112 more) |
| **Adverse event（liver）** | | | | | | | | | | |
| 482 (2 RCTs) | not serious | not serious | not serious | serious^a^ | None | ⨁⨁⨁◯ Moderate | 33/241 (13.7%) | 33/241 (13.7%) | **RR 1.00** (0.64 to 1.57) | **0 fewer per 1,000** (from 49 fewer to 78 more) |

**CI:** confidence interval; **MD:** mean difference; **RR:** risk ratio, **IC**: indirect calorimetry

a. Downgraded one level due to imprecision: the sample size is less than N=2000 (calculate OIS based on α=0.05, β=0.2, Event=20%, RRR=25%, N=2000)

b. Downgraded one level due to inconsistency: the percentage of variation between studies (I2) is high

c. Downgraded one level due to imprecision: the sample size is less than N=800 (calculate OIS based on empirical thresholds; α=0.05, β=0.2, d=0.2~0.3, N=800)

d. Downgraded two levels due to inconsistency: the percentage of variation between studies (I2) is high and significant in the heterogeneity test
