# Supplementary material for: The Japanese Critical Care Nutrition Guideline 2024
Source: J Intensive Care. 2025 Mar 21;13:18. doi: 10.1186/s40560-025-00785-z (PMC11927338; doi:10.1186/s40560-025-00785-z)
Supplement: Supplementary file 4 — Additional file 4: CQ4 Evidence profiles. [file 40560_2025_785_MOESM4_ESM.docx]

| **Table 1. CQ 4-3 Evidence Profile** | | | | | | | | | | |
| --- | --- | --- | --- | --- | --- | --- | --- | --- | --- | --- |
| **Certainty assessment** | | | | | | | **Summary of findings** | | | |
| **Participants (studies)** | **Risk of bias** | **Inconsistency** | **Indirectness** | **Imprecision** | **Publication bias** | **Overall certainty of evidence** | **Study event rates (%)** | | **Relative effect (95% CI)** | **Anticipated absolute effects**  **(95%CI)** |
|  |  |  |  |  |  |  | **With standard protein intake** | **With high-protein intake** |  |  |
| **Mortality** | | | | | | | | | | |
| 121 (2 RCTs) | serious^a^ | very serious^b^ | not serious | very serious^d^ | none | ⨁◯◯◯ Very low | 10/61 (16.4%) | 9/60 (15.0%) | **RR 0.90** (0.41 to 1.98) | **16 fewer per 1,000** (from 97 fewer to 161 more) |
| **Length of ICU stay** | | | | | | | | | | |
| 118 (2 RCTs) | serious^a^ | serious^c^ | not serious | very serious^d^ | none | ⨁◯◯◯ Very low | 51 | 67 | - | **MD 0.95 longer** (1.87 shorter to 3.77 longer) |
| **Duration of mechanical ventilation** | | | | | | | | | | |
| 118 (2 RCTs) | serious^a^ | serious^c^ | not serious | very serious^d^ | none | ⨁◯◯◯ Very low | 51 | 67 | - | **MD 1.3 shorter** (3.95 shorter to 1.35 longer) |
| **Diarrhea** | | | | | | | | | | |
| 66 (2 RCTs) | serious^a^ | not serious | not serious | very serious^d^ | none | ⨁◯◯◯ Very low | 4/23 (17.4%) | 14/43 (32.6%) | **RR 1.38** (0.59 to 3.20) | **66 more per 1,000** (from 71 fewer to 383 more) |
| **Infection complications** | | | | | | | | | | |
| 66 (2 RCTs) | serious^a^ | not serious | not serious | very serious^d^ | none | ⨁◯◯◯ Very low | 3/23 (13.0%) | 8/43 (18.6%) | **RR 1.06** (0.36 to 3.13) | **8 more per 1,000** (from 83 fewer to 278 more) |
| **Muscle mass change** | | | | | | | | | | |
| 38 (1 RCT) | serious^a^ | not serious | not serious | serious^e^ | none | ⨁⨁◯◯ Low | 11 | 27 | - | **MD 0.2 shorter** (1.15 shorter to 0.75 longer) |
| **Hyperproteinemia** | | | | | | | | | | |
| 41 (1 RCT) | serious^a^ | not serious | not serious | not serious | none | ⨁⨁⨁◯ Moderate | 0/21 (0.0%) | 0/20 (0.0%) | not estimable | - |

**CI:** confidence interval; **MD:** mean difference; **RR:** risk ratio

a. Downgraded one level due to some concern about risk of bias.

b. Downgraded two level due to considerable heterogeneity.

c. Downgraded one level due to moderate heterogeneity.

d. Downgraded two level: optimal information size (OIS) is not met and confidence interval for effect estimate includes considerable benefit and considerable harm.

e. Downgraded one level: confidence interval for effect estimate includes considerable benefit and considerable harm.

| **Table 2. CQ 4-4 Evidence profile** | | | | | | | | | | |
| --- | --- | --- | --- | --- | --- | --- | --- | --- | --- | --- |
| **Certainty assessment** | | | | | | | **Summary of findings** | | | |
| **Participants (studies)** | **Risk of bias** | **Inconsistency** | **Indirectness** | **Imprecision** | **Publication bias** | **Overall certainty of evidence** | **Study event rates (%)** | | **Relative effect (95% CI)** | **Anticipated absolute effects (95%CI)** |
|  |  |  |  |  |  |  | **With late EN** | **With early EN** |  |  |
| **Length of ICU stay** | | | | | | | | | | |
| 30 (1 RCT) | not serious | not serious | not serious | very serious ^a^ | publication bias strongly suspected | ⨁◯◯◯ Very low | 15 | 15 | - | **MD 2.07 shorter** (4.07 shorter to 0.07 longer) |
| **Adverse events** | | | | | | | | | | |
| 30 (1 RCT) | not serious | not serious | not serious | very serious ^a^ | publication bias strongly suspected | ⨁◯◯◯ Very low | 0/15 (0.0%) | 0/15 (0.0%) | not estimable | - |

**CI:** confidence interval; **EN:** enteral nutrition **MD:** mean difference; **RR:** risk ratio

1. Downgraded two levels: The total sample size does not meet the optimal information size (OIS) and there is only one relevant RCT.

| **Table 3. CQ 4-6 Evidence profile** | | | | | | | | | | |
| --- | --- | --- | --- | --- | --- | --- | --- | --- | --- | --- |
| **Certainty assessment** | | | | | | | **Summary of findings** | | | |
| **Participants (studies)** | **Risk of bias** | **Inconsistency** | **Indirectness** | **Imprecision** | **Publication bias** | **Overall certainty of evidence** | **Study event rates (%)** | | **Relative effect (95% CI)** | **Anticipated absolute effects(95%CI)** |
|  |  |  |  |  |  |  | **With gastric feeding** | **With postpyloric feeding** |  |  |
| **Mortality** | | | | | | | | | | |
| 102 (2 RCTs) | not serious | not serious | not serious | very serious^d^ | none | ⨁⨁◯◯ Low | 4/52 (7.7%) | 7/50 (14.0%) | **RR 1.80** (0.57 to 5.71) | **62 more per 1,000** (from 33 fewer to 362 more) |
| **Length of hospital stay** | | | | | | | | | | |
| 142 (3 RCTs) | serious^a^ | not serious | not serious | serious^e^ | none | ⨁⨁◯◯ Low | 72 | 70 | - | **MD 2.9 longer** (5.46 shorter to 11.25 longer) |
| **Duration of mechanical ventilation** | | | | | | | | | | |
| 102 (2 RCTs) | not serious | not serious | not serious | serious^e^ | none | ⨁⨁⨁◯ Moderate | 52 | 50 | - | **MD 6 longer** (0.4 shorter to 12.4 longer) |
| **Ventilator-associated pneumonia** | | | | | | | | | | |
| 40 (1 RCT) | not serious | not serious | not serious | very serious^d^ | none | ⨁⨁◯◯ Low | 4/20 (20.0%) | 2/20 (10.0%) | **RR 0.50** (0.10 to 2.43) | **100 fewer per 1,000** (from 180 fewer to 286 more) |
| **Aspiration** | | | | | | | | | | |
| 106 (2 RCTs) | serious^a^ | not serious | serious^c^ | very serious^d^ | none | ⨁◯◯◯ Very low | 19/59 (32.2%) | 22/47 (46.8%) | **RR 1.75** (0.32 to 9.49) | **242 more per 1,000** (from 219 fewer to 1,000 more) |
| **Emesis** | | | | | | | | | | |
| 102 (2 RCTs) | serious^a^ | serious^b^ | not serious | very serious^d^ | none | ⨁◯◯◯ Very low | 16/52 (30.8%) | 11/50 (22.0%) | **RR 0.53** (0.08 to 3.35) | **145 fewer per 1,000** (from 283 fewer to 723 more) |
| **Enteral nutrition initiation time** | | | | | | | | | | |
| 44 (1 RCT) | Serious^a^ | not serious | not serious | serious^e^ | none | ⨁⨁◯◯ Low | 27 | 17 | - | **MD 18 longer** (15.31 shorter to 20.69 longer) |

**CI:** confidence interval; **MD:** mean difference; **RR:** risk ratio

a. Downgraded one level due to risk of bias.

b. Downgraded one level due to moderate heterogeneity.

c. Downgraded one level due to reported surrogate outcome.

d. Downgraded two levels: optimal information size (OIS) is not met and confidence interval for effect estimate includes considerable benefit and considerable harm.

e. Downgraded one level: optimal information size (OIS) is not met.

| **Table 4. CQ 4-7 Evidence profile** | | | | | | | | | | |
| --- | --- | --- | --- | --- | --- | --- | --- | --- | --- | --- |
| **Certainty assessment** | | | | | | | **Summary of findings** | | | |
| **Participants (studies)** | **Risk of bias** | **Inconsistency** | **Indirectness** | **Imprecision** | **Publication bias** | **Overall certainty of evidence** | **Study event rates (%)** | | **Relative effect (95% CI)** | **Anticipated absolute effects (95%CI)** |
|  |  |  |  |  |  |  | **With continuous EN** | **With intermittent** |  |  |
| **Length of ICU stay** | | | | | | | | | | |
| 85 (2 RCTs) | serious^a^ | not serious | not serious | very serious^f^ | none | ⨁◯◯◯ Very low | 44 | 41 | - | **MD 0.61 longer** (3.18 shorter to 4.4 longer) |
| **Duration of mechanical ventilation** | | | | | | | | | | |
| 25 (1 RCT) | very serious^b^ | serious^c^ | not serious | very serious^f^ | none | ⨁◯◯◯ Very low | 14 | 11 | - | **MD 1 shorter** (6.03 shorter to 4.03 longer) |
| **Ventilator-associated infections** | | | | | | | | | | |
| 144 (1 RCT) | very serious^b^ | serious^c^ | not serious | very serious^g^ | none | ⨁◯◯◯ Very low | 0/72 (0.0%) | 0/72 (0.0%) | not estimable | - |
| **Emesis** | | | | | | | | | | |
| 276 (4 RCTs) | serious^a^ | serious^d^ | not serious | very serious^h^ | none | ⨁◯◯◯ Very low | 24/140 (17.1%) | 20/136 (14.7%) | **RR 0.97** (0.43 to 2.20) | **5 fewer per 1,000** (from 98 fewer to 206 more) |
| **Diarrhea** | | | | | | | | | | |
| 105 (2 RCTs) | not serious | not serious | not serious | very serious^h^ | none | ⨁⨁◯◯ Low | 24/52 (46.2%) | 23/53 (43.4%) | **RR 0.95** (0.54 to 1.70) | **23 fewer per 1,000** (from 212 fewer to 323 more) |
| **Gastric residual volume** | | | | | | | | | | |
| 191 (2 RCTs) | very serious^b^ | not serious | not serious | very serious^h^ | none | ⨁◯◯◯ Very low | 33/96 (34.4%) | 26/95 (27.4%) | **RR 0.80** (0.52 to 1.22) | **69 fewer per 1,000** (from 165 fewer to 76 more) |
| **Time to target feeding goal** | | | | | | | | | | |
| 206 (2 RCTs) | serious^a^ | very serious^e^ | not serious | serious^i^ | none | ⨁◯◯◯ Very low | 104 | 102 | - | **MD 0.38 longer** (1.14 shorter to 1.89 longer) |

**CI:** confidence interval; **MD:** mean difference; **RR:** risk ratio

a. Downgraded one level: included some studies were analyzed per protocol analysis.

b. Downgraded two levels: included most studies were analyzed per protocol analysis.

c. Downgraded one level: only one RCT was included.

d. Downgraded one level due to moderate heterogeneity.

e. Downgraded two levels due to considerable heterogeneity.

f. Downgraded two levels: optimal information size is considerably small and confidence interval for effect estimate includes considerable benefit and considerable harm.

g. Downgraded two levels: optimal information size is considerably small and no event occurred.

h. Downgraded two levels: optimal information size is small and confidence interval for effect estimate includes considerable benefit and considerable harm.

i. Downgraded one level: confidence interval for effect estimate includes considerable benefit and considerable harm.

| **Table 5. CQ 4-8 Evidence Profile** | | | | | | | | | | |
| --- | --- | --- | --- | --- | --- | --- | --- | --- | --- | --- |
| **Certainty assessment** | | | | | | | **Summary of findings** | | | |
| **Participants (studies)** | **Risk of bias** | **Inconsistency** | **Indirectness** | **Imprecision** | **Publication bias** | **Overall certainty of evidence** | **Study event rates (%)** | | **Relative effect (95% CI)** | **Anticipated absolute effects (95%CI)** |
|  |  |  |  |  |  |  | **With standard-density formula** | **With higher-density formula** |  |  |
| **Mortality** | | | | | | | | | | |
| 417 (4 RCTs) | serious^a^ | not serious | not serious | serious^d^ | none | ⨁⨁◯◯ Low | 5/206 (2.4%) | 10/211 (4.7%) | **RR 2.05** (0.76 to 5.50) | **25 more per 1,000** (from 6 fewer to 109 more) |
| **Length of ICU stay** | | | | | | | | | | |
| 405 (5 RCTs) | serious^a^ | not serious | not serious | serious^d^ | none | ⨁⨁◯◯ Low | 202 | 203 | - | **MD 0.09 longer** (0.7 shorter to 0.87 longer) |
| **Duration of mechanical ventilation** | | | | | | | | | | |
| 371 (4 RCTs) | serious^a^ | not serious | not serious | serious^d^ | none | ⨁⨁◯◯ Low | 184 | 187 | - | **MD 0.2 shorter** (0.63 shorter to 0.23 longer) |
| **Emesis** | | | | | | | | | | |
| 469 (6 RCTs) | very serious^b^ | not serious | not serious | very serious^e^ | none | ⨁◯◯◯ Very low | 5/234 (2.1%) | 9/235 (3.8%) | **RR 1.76** (0.66 to 4.70) | **16 more per 1,000** (from 7 fewer to 79 more) |
| **Diarrhea** | | | | | | | | | | |
| 430 (5 RCTs) | very serious^b^ | not serious | not serious | very serious^e^ | none | ⨁◯◯◯ Very low | 6/213 (2.8%) | 14/217 (6.5%) | **RR 1.64** (0.61 to 4.40) | **18 more per 1,000** (from 11 fewer to 96 more) |
| **Gastrointestinal bleeding** | | | | | | | | | | |
| 109 (2 RCTs) | serious^a^ | not serious | not serious | very serious^e^ | none | ⨁◯◯◯ Very low | 11/54 (20.4%) | 9/55 (16.4%) | **RR 0.78** (0.38 to 1.64) | **45 fewer per 1,000** (from 126 fewer to 130 more) |
| **Weight for age Z score** | | | | | | | | | | |
| 367 (3 RCTs) | serious^c^ | not serious | not serious | serious^d^ | none | ⨁⨁◯◯ Low | 182 | 185 | - | **MD 0.61 higher** (0.28 higher to 0.94 higher) |

**CI:** confidence interval; **MD:** mean difference; **RR:** risk ratio

a. Downgraded one level due to some concerns in derivations from intended interventions and selection of reported results.

b. Downgraded two levels due to some concerns in derivations from intended interventions, measurement of the outcomes, and selection of reported results.

c. Downgraded one level due to some concerns in derivations from intended interventions, and missing values.

d. Downgraded one level: confidence interval for effect estimate includes considerable benefit and considerable harm.

e. Downgraded two levels: optimal information size is not met and confidence interval for effect estimate includes considerable benefit and considerable harm.
