# Supplementary material for: The Japanese Critical Care Nutrition Guideline 2024
Source: J Intensive Care. 2025 Mar 21;13:18. doi: 10.1186/s40560-025-00785-z (PMC11927338; doi:10.1186/s40560-025-00785-z)
Supplement: Supplementary file 5 — Additional file 5: Nutritional Assessment. [file 40560_2025_785_MOESM5_ESM.docx]

**CQ3-1: Is a nutritional assessment necessary before providing nutrition therapy to critically ill patients?**

Supplemental table 1. Nutritional Assessment

| Tool |  | MST; malnutrition screening tool | MUST; malnutrition universal screening tool | PONS; perioperative nutrition screening | NRS 2002; Nutrition Risk Screening | PNI; prognostic nutritional index | NRI; nutritional risk index | GNRI; Geriatric Nutritional Risk Index | CONUT; Controlling Nutrition Status | GLIM; Global Leadership Initiative on Malnutrition | NUTRIC; Nutrition Risk in the Critically Ill | mNUTRIC; modified NUTRIC | SCREENIC; Screening of Nutritional Risk in Intensive Care | MNA; mini-nutritional assessment | MNA-SF (mini nutritional assessment short form) | SGA; Subjective Global Assessment |
| --- | --- | --- | --- | --- | --- | --- | --- | --- | --- | --- | --- | --- | --- | --- | --- | --- |
|  |  |  |  |  |  |  |  |  |  |  |  |  |  |  |  |  |
| Assessment items | weight loss | ○ | ○ | ○ | ○ |  | ○ | ○ |  | ○ | ○ | ○ |  | ○ | ○ | ○ |
|  | BMI |  | ○ | ○ | ○ |  |  |  |  | ○ |  |  |  | ○ | ○ |  |
|  | severity/acute phase |  | ○ |  | ○ |  |  |  |  | ○ | ○ | ○ | 〇 | ○ | ○ |  |
|  | Gastrointestinal symptoms |  |  |  |  |  |  |  |  |  |  |  |  |  |  | ○ |
|  | physical assessment |  |  |  |  |  |  |  |  | 〇  (muscle) |  |  | 〇 | ○ | ○ | ○ |
|  | mobility, functional assessment |  |  |  |  |  |  |  |  |  | ○ | ○ |  | ○ | ○ | ○ |
|  | cognitive function |  |  |  |  |  |  |  |  |  |  |  |  | ○ | ○ |  |
|  | considered age |  |  | ○ | ○ |  | ○ | ○ |  |  | ○ | ○ | 〇 | ○ | ○ |  |
|  | serum albumin |  |  | ○ |  | ○ | ○ | ○ | ○ |  |  |  |  |  |  |  |
|  | nutritional intake | ○ | ○ | ○ | ○ |  |  |  |  | 〇 |  |  |  | ○ | ○ | ○ |
|  | Total lymphocyte count |  |  |  |  | ○ |  |  | ○ |  |  |  |  |  |  |  |
| subjects | can be used for critically ill |  | ○ |  | ○ |  |  |  |  | ○ | ○ | ○ | ○ |  |  |  |
|  | originally developed for critically ill |  |  |  | ○ |  |  |  |  |  | ○ | ○ | ○ |  |  |  |
|  | originally developed for elderly |  |  |  |  |  |  | ○ |  | ○ |  |  |  | ○ | ○ |  |
|  | can be used for pediatric |  |  | ○ |  | ○ | ○ |  |  |  |  |  |  |  |  |  |
| Characteristics | Evaluation Contents | Screening tool consisting by only weight loss and food intake | Screening tool consisting of BMI, weight loss, acute illness and nutritional deficiencies | Perioperative nutritional assessment add ed serum Alb levels to assess risk | initial screening（BMI, weight loss, nutritional intake, severity) and final screening | PNI=[10xserum alb (g/dl)] + [0.005xTLC (/mm3)] | Calculated by serum weight loss and serum albumin | weight, height, and serum albumin levels | only assessed by blood and biochemistry test; serum albumin, cholesterol, TLC | Phenotypic criteria (weight loss, BMI, muscle loss) and Etiologic criteria (nutritional intake, severity/acute phase) | age, APACHE II, SOFA, number of co-mobilities, days from hospital to ICU admission, IL-6 | Calculated excluding IL-6 from NUTRIC | comorbidities, hospitalized days before ICU, sepsis, ventilation upon ICU admission, aged >65 years, signs of moderate/severe muscle mass loss | 18 questions (option of using CC; Calf circumference when BMI cannot be calculated.) | 6 questions (food intake, weight loss, morbidity, psychological stress, neuropsychological stress, MI/CC) | disease history and physical examination |
|  | Characteristics | Scouring, but specify as screening tool | Scouring, but specify as screening tool | Tools to Screen Surgical Patients for Nutritional Risk | Screening method proposed by ESPEN, Two-step screening | Original score calculation formula. Prognostic indicators such as risk of postoperative suture failure | Original score calculation formula. Classical nutritional risk formula used to detect risk in the elderly | assess malnutritional risk in hospitalized adults over 65 years | Scouring to diagnosis severity of malnutrition | global consensus around core diagnostic criteria for malnutrition in adults in clinical settings. | first nutritional risk assessment tool developed specifically for ICU | modified NUTRIC | For ICU patients, can be assessed even without a detailed history. | nutrition screening and assessment tool that can identify geriatric patients who are malnourished or at risk of malnutrition. | Simplified version of NMA, used primarily for screening | Can be evaluated by history and physical examination alone. |
| Reference | Original | 1 | 2 | 3 | 4 | 5 | 6 | 7 | 8 | 9 | 10 | 11 | 12 | 13 | 14 | 15 |
|  | Example of use in critical ill patients | 16 | 16, 17, 18 | n/a | 18, 19 24 | 20, 21 | 22 | 21, 23 | 22 | 24 | 10 | 11,22,24, 25 | 12 | 26 | 27 | 27 |
