# Supplementary material for: The Japanese Critical Care Nutrition Guideline 2024
Source: J Intensive Care. 2025 Mar 21;13:18. doi: 10.1186/s40560-025-00785-z (PMC11927338; doi:10.1186/s40560-025-00785-z)
Supplement: Supplementary file 6 — Additional file 6: Other supplementary files. [file 40560_2025_785_MOESM6_ESM.docx]

**CQ1-1: Should nutrition therapy using nutrition protocols be provided in critically ill patients?**

Search Database

- MEDLINE (PubMed/Ovid)

Search Strategy and Hits:

PubMed search strategy（Search Date: April 18, 2023）

| #1 | "Intensive Care Units"[Mesh] OR “intensive care"[tiab] OR "Critical Illness"[Mesh] OR "critical care"[Mesh] OR “critically ill”[tiab] OR "critical care"[tiab] | 308,146 |
| --- | --- | --- |
| #2 | "Nutrition Therapy"[MeSH Terms] OR "Nutrition Therapy"[tiab] OR "Nutrition Support"[tiab] OR "Enteral Nutrition"[tiab] OR "Parenteral Nutrition"[tiab] OR "Diet Therapy"[tiab] OR "Feed*"[tiab] OR "Artificial Nutrition"[tiab] | 628,060 |
| #3 | "clinical protocols"[Mesh] OR "protocol*"[tiab] OR "algorithm*"[tiab] OR "guideline"[tiab] OR "procedure*"[tiab] OR "nursing assessment"[tiab] OR "nursing process"[tiab] | 2,333,491 |
| #4 | randomized controlled trial[pt] OR controlled clinical trial[pt] OR randomized[tiab] OR placebo[tiab] OR drug therapy[sh] OR randomly[tiab] OR trial[tiab] OR groups[tiab] NOT (animals [mh]  NOT humans [mh]) | 4,987,798 |
| #5 | #1 AND #2 AND #3 AND #4 | 886 |

Search Database

- CENTRAL

Search Strategy and Hits:

CENTRAL search strategy（Search Data: April 18, 2023）

| #1 | MeSH descriptor: [Intensive Care Units] explode all trees | 5,225 |
| --- | --- | --- |
| #2 | ("intensive care"):ti,ab,kw | 28,987 |
| #3 | MeSH descriptor: [Critical Illness] explode all trees | 3,218 |
| #4 | ("Critical illness"):ti,ab,kw | 4,441 |
| #5 | ("critically ill"):ti,ab,kw | 8,214 |
| #6 | MeSH descriptor: [Critical Care] explode all trees | 2,650 |
| #7 | ("critical care"):ti,ab,kw | 4,859 |
| #8 | ("intensive care"):ti,ab,kw | 28,987 |
| #9 | #1 OR #2 OR #3 OR #4 OR #5 OR #6 OR #7 #8 | 32,972 |
| #10 | MeSH descriptor: [Nutrition Therapy] explode all trees | 12,025 |
| #11 | ("Nutrition Therapy"):ti,ab,kw | 712 |
| #12 | ("Nutrition Support"):ti,ab,kw | 643 |
| #13 | ("Enteral Nutrition"):ti,ab,kw | 4,963 |
| #14 | ("Parenteral Nutrition"):ti,ab,kw | 4,450 |
| #15 | ("Diet Therapy"):ti,ab,kw | 12,748 |
| #16 | (Feed*):ti,ab,kw | 51,360 |
| #17 | ("Artificial Nutrition"):ti,ab,kw | 104 |
| #18 | #10 OR #11 OR #12 OR #13 OR #14 OR #15 OR #16 OR #17 | 71,596 |
| #19 | MeSH descriptor: [Clinical Protocols] explode all trees | 23,580 |
| #20 | (protocol*):ti,ab,kw | 151,142 |
| #21 | (algorithm*):ti,ab,kw | 17,065 |
| #22 | (guideline):ti,ab,kw | 19,593 |
| #23 | (procedure*):ti,ab,kw | 290,171 |
| #24 | ("nursing assessment"):ti,ab,kw | 632 |
| #25 | ("nursing process"):ti,ab,kw | 180 |
| #26 | #19 OR #20 OR #21 OR #22 OR #23 OR #24 OR #25 | 434,827 |
| #27 | #9 AND #18 AND #26 | 1,138 |

Search Database

■Igaku Chuo Zasshi

Search Strategy and Hits:

Igaku Chuo Zasshi search strategy（Search Data: April 20, 2023）

| #1 | ((ICU/TH or ICU/TA or "intensive care unit"/TA)) and (PT=会議録除く) | 55,436 |
| --- | --- | --- |
| #2 | ((クリティカルケア/TH or クリティカルケア/TA or クリティカル・ケア/TA)) and (PT=会議録除く) | 7,487 |
| #3 | ((集中治療/TA or 重症患者/TA)) and (PT=会議録除く) | 15,652 |
| #4 | #1 or #2 or #3 | 68,416 |
| #5 | ((栄養管理/TH or 栄養療法/TA or 栄養サポート/TA or 経腸栄養/TA or 静脈栄養/TA or 食事療法/TA or 摂取/TA or 人工栄養/TA)) and (PT=会議録除く) | 117,139 |
| #6 | ((臨床プロトコール/TH or 臨床プロトコール/TA or プロトコール/TA or アルゴリズム/TA or ガイドライン/TA or 手順/TA or 看護アセスメント/TA or 看護手順/TA)) and (PT=会議録除く) | 112,190 |
| #7 | ((ランダム化比較試験/TH or 準ランダム化比較試験/TH or ランダム化/AL or 無作為化/AL or 比較試験/AL or 臨床試験/AL or プラセボ/AL or 対照/AL or コントロール/AL or 臨床研究/AL)) | 349,708 |
| #8 | #4 and #5 and #6 and #7 | 43 |

Flow Diagram

Risk of bias

Overall mortality

Length of ICU stay

Duration of mechanical ventilation

Pneumonia

Renal replacement therapy

Vomiting

Enteral nutrition initiation time

Forest Plot and　Funnel Plot

Overall mortality

Length of ICU stay

Duration of mechanical ventilation

Pneumonia

Renal replacement therapy

Vomiting

Enteral nutrition initiation time

**CQ1-2：Should enteral nutrition be administered rather than parenteral nutrition in critically ill patients?**

Search Database

- MEDLINE (PubMed/Ovid)

Search Strategy and Hits:

PubMed search strategy （Search Data: April 9, 2023）

| #1 | "Intensive Care Units"[Mesh] OR "intensive care"[tiab] OR "Critical Illness"[Mesh] OR "critical care"[Mesh] OR "critically ill"[tiab] OR "critical care"[tiab] | 307,667 |
| --- | --- | --- |
| #2 | Sepsis[Mesh] OR "Shock, Septic"[Mesh] OR Bacteremia[Mesh] OR sepsis[tiab] OR "septic shock"[tiab] OR bacteremia[tiab] | 226,256 |
| #3 | "Systemic Inflammatory Response Syndrome"[Mesh] OR "systemic inflammatory response syndrome"[tiab] OR SIRS[tiab] | 153,268 |
| #4 | "Multiple Organ Failure"[Mesh] OR "organ failure*"[tiab] OR "organ dysfunction"[tiab] OR MOF[tiab] OR MODS[tiab] | 59,755 |
| #5 | Burns[Mesh] OR "Burn Units"[Mesh] OR burn[tiab] | 76,647 |
| #6 | "Multiple Trauma"[Mesh] OR "Brain Injuries"[Mesh] OR "multiple trauma"[tiab] OR "brain injur*"[tiab] OR "head trauma"[tiab] OR "head injur*"[tiab] | 154,972 |
| #7 | Pancreatitis[Mesh] OR pancreatitis[tiab] | 75,873 |
| #8 | "Respiratory Distress Syndrome"[Mesh] OR "Acute Lung Injury"[Mesh] OR "acute respiratory distress syndrome"[tiab] OR "acute lung injury"[tiab] OR "ARDS"[tiab] OR ALI[tiab] | 70,183 |
| #9 | "Respiration, Artificial"[Mesh] OR "mechanical ventilation"[tiab] OR "mechanically ventilated"[tiab] OR IMV[tiab] OR MV[tiab] | 200,995 |
| #10 | "Surgical Procedures, Operative"[Mesh] OR "Postoperative Period"[Mesh] OR "surgery"[tiab] OR surgical*[tiab] OR postoperative[tiab] | 4,689,054 |
| #11 | #1 OR #2 OR #3 OR #4 OR #5 OR #6 OR #7 OR #8 OR #9 OR #10 | 5,469,012 |
| #12 | "Enteral Nutrition"[Mesh] OR "enteral nutrition"[tiab] OR "enteral feeding"[tiab] OR "feeding tube*"[tiab] OR "force feeding*"[tiab] OR "tube feeding*"[tiab] OR "gastric tube*" [tiab] OR “PEG line*”[tiab] OR “enteric nutrition*”[tiab] OR “enteric feeding*”[tiab] OR EN[tiab] | 103,032 |
| #13 | "Parenteral Nutrition"[Mesh] OR "parenteral nutrition"[tiab] OR "parenteral feeding*"[tiab] OR "intravenous feeding*"[tiab] OR "intravenous nutrition*"[tiab] OR PN[tiab] | 56,635 |
| #14 | #12 AND #13 | 7,590 |
| #15 | randomized controlled trial[pt] OR controlled clinical trial[pt] OR randomized[tiab] OR placebo[tiab] OR drug therapy[sh] OR randomly[tiab] OR trial[tiab] OR groups[tiab] NOT (animals [Mesh] NOT humans [Mesh]) | 4,980,857 |
| #16 | #11 AND #14 AND #15 | 1,260 |

Search Database

- CENTRAL

Search Strategy and Hits: CENTRAL search strategy（Search Data: April 22, 2023）

| #1 | MeSH descriptor: [Intensive Care Units] explode all trees | 5225 |
| --- | --- | --- |
| #2 | ("intensive care"):ti,ab,kw | 28987 |
| #3 | MeSH descriptor: [Critical Illness] explode all trees | 3218 |
| #4 | (“Critical illness”):ti,ab,kw | 4441 |
| #5 | (“Critically ill”):ti,ab,kw | 8214 |
| #6 | MeSH descriptor: [Critical Care] explode all trees | 2650 |
| #7 | ("critical care"):ti,ab,kw | 4859 |
| #8 | MeSH descriptor: [Sepsis] explode all trees | 6710 |
| #9 | (sepsis):ti,ab,kw | 13042 |
| #10 | MeSH descriptor: [Shock, Septic] explode all trees | 1236 |
| #11 | ("septic shock"):ti,ab,kw | 3561 |
| #12 | MeSH descriptor: [Bacteremia] explode all trees | 1304 |
| #13 | (bacteremia):ti,ab,kw | 2769 |
| #14 | MeSH descriptor: [Systemic Inflammatory Response Syndrome] explode all trees | 7277 |
| #15 | ("systemic inflammatory response syndrome"):ti,ab,kw | 1244 |
| #16 | (SIRS):ti,ab,kw | 856 |
| #17 | MeSH descriptor: [Multiple Organ Failure] explode all trees | 640 |
| #18 | ("organ failure*"):ti,ab,kw | 4109 |
| #19 | ("organ dysfunction"):ti,ab,kw | 2094 |
| #20 | (MOF):ti,ab,kw | 195 |
| #21 | (MODS):ti,ab,kw | 316 |
| #22 | MeSH descriptor: [Burns] explode all trees | 2093 |
| #23 | MeSH descriptor: [Burn Units] explode all trees | 49 |
| #24 | (burn):ti,ab,kw | 4696 |
| #25 | MeSH descriptor: [Multiple Trauma] explode all trees | 310 |
| #26 | ("multiple trauma"):ti,ab,kw | 592 |
| #27 | MeSH descriptor: [Brain Injuries] explode all trees | 3175 |
| #28 | (“brain injury”):ti,ab,kw | 6865 |
| #29 | ("head trauma"):ti,ab,kw | 611 |
| #30 | ("head injury"):ti,ab,kw | 1556 |
| #31 | MeSH descriptor: [Pancreatitis] explode all trees | 1883 |
| #32 | (pancreatitis):ti,ab,kw | 5266 |
| #33 | MeSH descriptor: [Respiratory Distress Syndrome] explode all trees | 3262 |
| #34 | ("acute respiratory distress syndrome"):ti,ab,kw | 2247 |
| #35 | (ARDS):ti,ab,kw | 2543 |
| #36 | MeSH descriptor: [Acute Lung Injury] explode all trees | 697 |
| #37 | ("acute lung injury"):ti,ab,kw | 1435 |
| #38 | (ALI):ti,ab,kw | 2231 |
| #39 | MeSH descriptor: [Respiration, Artificial] explode all trees | 8194 |
| #40 | ("mechanical ventilation"):ti,ab,kw | 12365 |
| #41 | ("mechanically ventilated"):ti,ab,kw | 3146 |
| #42 | (IMV):ti,ab,kw | 274 |
| #43 | (MV):ti,ab,kw | 2812 |
| #44 | MeSH descriptor: [Surgical Procedures, Operative] explode all trees | 164459 |
| #45 | MeSH descriptor: [Postoperative Period] explode all trees | 8312 |
| #46 | (surgery):ti,ab,kw | 259689 |
| #47 | (surgical*):ti,ab,kw | 122959 |
| #48 | (postoperative):ti,ab,kw | 142964 |
| #49 | #1 OR #2 OR #3 OR #4 OR #5 OR #6 OR #7 OR #8 OR #9 OR #10 OR #11 OR #12 OR #13 OR #14 OR #15 OR #16 OR #17 OR #18 OR #19 OR #20 OR #21 OR #22 OR #23 OR #24 OR #25 OR #26 OR #27 OR #28 OR #29 OR #30 OR #31 OR #32 OR #33 OR #34 OR #35 OR #36 OR #37 OR #38 OR #39 OR #40 OR #41 OR #42 OR #43 OR #44 OR #45 OR #46 OR #47 OR #48 | 438607 |
| #50 | MeSH descriptor: [Enteral Nutrition] explode all trees | 2179 |
| #51 | ("enteral nutrition"):ti,ab,kw | 4963 |
| #52 | ("enteral feeding"):ti,ab,kw | 1610 |
| #53 | ("feeding tube*"):ti,ab,kw | 784 |
| #54 | ("force feeding*"):ti,ab,kw | 3 |
| #55 | ("tube feeding*"):ti,ab,kw | 801 |
| #56 | ("gastric tube*"):ti,ab,kw | 779 |
| #57 | (“PEG line*”):ti,ab,kw | 0 |
| #58 | (“enteric nutrition*”):ti,ab,kw | 7 |
| #59 | (“enteric feeding*”):ti,ab,kw | 1770 |
| #60 | (EN):ti,ab,kw | 17799 |
| #61 | #50 OR #51 OR #52 OR #53 OR #54 OR #55 OR #56 OR #57 OR #58 OR #59 OR #60 | 24723 |
| #62 | MeSH descriptor: [Parenteral Nutrition] explode all trees | 1949 |
| #63 | ("parenteral nutrition"):ti,ab,kw | 4450 |
| #64 | ("parenteral feeding*"):ti,ab,kw | 165 |
| #65 | ("intravenous feeding*"):ti,ab,kw | 129 |
| #66 | ("intravenous nutrition*"):ti,ab,kw | 177 |
| #67 | (PN):ti,ab,kw | 2297 |
| #68 | #62 OR #63 OR #64 OR #65 OR #66 OR #67 | 6274 |
| #69 | #44 AND #61 AND #68 | 1035 |

Search Database

■Igaku Chuo Zasshi

Search Strategy and Hits:

Igaku Chuo Zasshi search strategy（Search Data: April 22, 2023）

| #1 | (ICU/TH or “intensive care unit”/TA or ICU/TA) and (PT=会議録除く) | 55,436 |
| --- | --- | --- |
| #2 | (クリティカルケア/TH or クリティカルケア/TA or クリティカル・ケア/TA or　集中治療/TA or　重症患者/TA) and (PT=会議録除く) | 19,616 |
| #3 | (敗血症/TH or 敗血症/TA or ショック-敗血症性/TH or 菌血症/TH or 菌血症/TA) and (PT=会議録除く) | 28,259 |
| #4 | (全身性炎症反応症候群/TH or 全身性炎症反応症候群/TA or SIRS/TA or "systemic inflammatory response syndrome"/TA) and (PT=会議録除く) | 22,148 |
| #5 | (多臓器不全/TH or 多臓器不全/TA or 多臓器障害/TA or MOF/TA or "multiple organ failure"/TA or MODS/TA or "multiple organ dysfunction syndrome"/TA) and (PT=会議録除く) | 8,730 |
| #6 | (熱傷/TH or 熱傷/TA) and (PT=会議録除く) | 11,626 |
| #7 | (多発性外傷/TH or 多発性外傷/TA or 多発外傷/TA or 頭部外傷/TH or 頭部外傷/TA or 脳血管外傷/TH or 脳血管外傷/TA) and (PT=会議録除く) | 32,891 |
| #8 | (膵炎/TH or 膵炎/TA) and (PT=会議録除く) | 21,529 |
| #9 | (呼吸窮迫症候群/TH or 急性肺損傷/TH or 呼吸窮迫症候群/TA or 急性肺損傷/TA or急性肺障害/TA or 急性肺傷害/TA or ARDS/TA or “acute respiratory distress syndrome”/TA or ALI/TA or “acute lung injury”/TA) and (PT=会議録除く) | 77,518 |
| #10 | (人工呼吸/TH or 人工呼吸/TA or “Mechanical Ventilation”/TA or IMV/TA or MV/TA) and (PT=会議録除く) | 46,527 |
| #11 | (外科手術/TH or 外科手術/TA or 術後期/TH or 術後/TA or 手術/TA) and (PT=会議録除く) | 1,071,785 |
| #12 | #1 or #2 or #3 or #4 or #5 or #6 or #7 or #8 or #9 or #10 or #11 | 1,265,270 |
| #13 | (経腸栄養/TH or 経腸栄養/TA or 経管栄養/TA or 経鼻栄養/TA or 経腸栄養剤/TH) and (PT=会議録除く) | 16,938 |
| #14 | (静脈栄養/TH or 静脈栄養/TA or 中心静脈栄養/TH or 静脈栄養剤/TH) and (PT=会議録除く) | 13,267 |
| #15 | #13 and #14 | 3,911 |
| #16 | ランダム化比較試験/TH or 準ランダム化比較試験/TH or ランダム化/AL or 無作為化/AL or 比較試験/AL or 臨床試験/AL orプラセボ/AL or 対照/AL or コントロール/AL or 臨床研究/AL | 349,708 |
| #17 | #12 and #15 and #16 | 163 |

Flow Diagram

Risk of Bias

90-day mortality

Sepsis（including bacteremia）

Pneumonia

Length of ICU stay

Duration of mechanical ventilation

One year EQ-5D

Mesenteric ischemia

Forest Plot and　Funnel Plot

90-day mortality

Sepsis (including bacteremia)

Pneumonia

Length of ICU stay

Duration of mechanical ventilation

One year EQ-5D

Mesenteric ischemia

（Sensitive analysis）

1. Excluding studies in which enteral nutrition was administered at usual dose to patients with shock

90-day mortality

Sepsis (including bacteremia)

Pneumonia

Length of ICU stay

Duration of mechanical ventilation

One year EQ-5D

Mesenteric ischemia

2. Bias risk low and some concern

90-day mortality

Sepsis (including bacteremia)

Pneumonia

Length of ICU stay

Duration of mechanical ventilation

One year EQ-5D

Mesenteric ischemia

3. After 2012

90-day mortality

Sepsis (including bacteremia)

Pneumonia

Length of ICU stay

Duration of mechanical ventilation

One year EQ-5D

Mesenteric ischemia

（Subgroup analysis）

1. Internal medicine except acute pancreatitis

90-day mortality

Sepsis (including bacteremia)

Pneumonia

Length of ICU stay

One year EQ-5D

Mesenteric ischemia

2. Acute pancreatitis

Sepsis (including bacteremia)

Pneumonia

Length of ICU stay

Duration of mechanical ventilation

3. Trauma

90-day mortality

Sepsis (including bacteremia)

Pneumonia

Length of ICU stay

Duration of mechanical ventilation

Mesenteric ischemia

**CQ1-3：Should energy provision be intentionally reduced below energy expenditure in the acute phase of critical illness?**

Search Database

■MEDLINE (PubMed/Ovid)

Search Strategy and Hits:

PubMed search strategy（Search Date: May 4, 2023）

| #1 | "Intensive Care Units"[MeSH Terms] OR "intensive care"[Title/Abstract] OR "Critical Illness"[MeSH Terms] OR "critical care"[MeSH Terms] OR "critically ill"[Title/Abstract] OR "critical care"[Title/Abstract] OR "respiration, artificial"[MeSH Terms] OR "mechanical ventilation"[Title/Abstract] | 389385 |
| --- | --- | --- |
| #2 | "energy intake"[MeSH Terms] OR "Caloric Restriction"[MeSH Terms] OR "calorimetry, indirect"[MeSH Terms] OR "calorie intake"[Title/Abstract] OR "underfeeding"[Title/Abstract] OR "hypocaloric"[Title/Abstract] OR "trophic"[Title/Abstract] OR "energy expenditure"[Title/Abstract] OR "low-calorie"[Title/Abstract] OR "calorimetry"[Title/Abstract] OR "energy intake"[Title/Abstract] OR "isocaloric"[Title/Abstract] OR "eucaloric"[Title/Abstract] OR "standard feeding"[Title/Abstract] OR "normocaloric"[Title/Abstract] | 170395 |
| #3 | "randomized controlled trial"[Publication Type] OR "controlled clinical trial"[Publication Type] OR "randomized"[Title/Abstract] OR "placebo"[Title/Abstract] OR "drug therapy"[MeSH Subheading] OR "randomly"[Title/Abstract] OR "trial"[Title/Abstract] OR "groups"[Title/Abstract]) NOT ("animals"[MeSH Terms] NOT "humans"[MeSH Terms] | 684698 |

Search Database

■CENTRAL

Search Strategy and Hits: CENTRAL search strategy（Search Date: April 19, 2023）

| #1 | MeSH descriptor: [Intensive Care Units] explode all trees | 6440 |
| --- | --- | --- |
| #2 | MeSH descriptor: [Critical Illness] explode all trees | 3218 |
| #3 | (“critical illness”):ti,ab,kw | 5670 |
| #4 | (“critically ill”):ti,ab,kw | 8214 |
| #5 | MeSH descriptor: [Critical Care] explode all trees | 2650 |
| #6 | ("critical care"):ti,ab,kw | 4859 |
| #7 | ("intensive care"):ti,ab,kw | 28987 |
| #8 | MeSH descriptor: [Respiration, Artificial] explode all trees | 8194 |
| #9 | ("mechanical ventilation"):ti,ab,kw | 12365 |
| #10 | #1 OR #2 OR #3 OR #4 OR #5 OR #6 OR #7 OR #8 OR #9 | 46925 |
| #11 | MeSH descriptor: [Energy Intake] explode all trees | 6440 |
| #12 | MeSH descriptor: [Caloric Restriction] explode all trees | 1132 |
| #13 | MeSH descriptor: [Calorimetry] explode all trees | 749 |
| #14 | ("energy intake"):ti,ab,kw | 9188 |
| #15 | ("calorie intake"):ti,ab,kw | 784 |
| #16 | ("hypocaloric"):ti,ab,kw | 1221 |
| #17 | ("underfeeding"):ti,ab,kw | 90 |
| #18 | ("trophic"):ti,ab,kw | 436 |
| #19 | ("energy expenditure"):ti,ab,kw | 5250 |
| #20 | ("low-calorie"):ti,ab,kw | 1988 |
| #21 | ("calorimetry"):ti,ab,kw | 2440 |
| #22 | ("isocaloric"):ti,ab,kw | 2669 |
| #23 | ("eucaloric"):ti,ab,kw | 171 |
| #24 | ("standard feeding"):ti,ab,kw | 71 |
| #25 | ("normocaloric"):ti,ab,kw | 88 |
| #26 | #11 OR #12 OR #13 OR #14 OR #15 OR #16 OR #17 OR #18 OR #19 OR #20 OR #21 OR #22 OR #23 OR #24 OR #25 | 20515 |
| #27 | #10 AND #26 | 751 |
| #38 |  |  |

**Search Database**

■Igaku Chuo Zasshi

**Search Strategy and Hits:**

Igaku Chuo Zasshi search strategy（Search Date: May 3, 2023）

| #1 | ((ICU/TH or "intensive care unit"/TA or ICU/TA) and (PT=会議録除く)) and (PT=会議録除く) | 55,436 |
| --- | --- | --- |
| [#2](https://search-old.jamas.or.jp/index.php?module=Advanced&action=Search&sid=%232) | (((クリティカルケア/TH or クリティカルケア/TA or クリティカル・ケア/TA or 集中治療/TA) and (PT=会議録除く))) | 16,525 |
| [#3](https://search-old.jamas.or.jp/index.php?module=Advanced&action=Search&sid=%233) | (重症患者/TA) and (PT=会議録除く) | 3,899 |
| [#4](https://search-old.jamas.or.jp/index.php?module=Advanced&action=Search&sid=%234) | (危篤/TH or 危篤/TA) and (PT=会議録除く) | 225 |
| [#5](https://search-old.jamas.or.jp/index.php?module=Advanced&action=Search&sid=%235) | (人工呼吸/TH or 人工呼吸/TA) and (PT=会議録除く) | 37,121 |
| [#6](https://search-old.jamas.or.jp/index.php?module=Advanced&action=Search&sid=%236) | #1 or #2 or #3 or #4 or #5 | 99,702 |
| [#7](https://search-old.jamas.or.jp/index.php?module=Advanced&action=Search&sid=%237) | ((エネルギー摂取量/TH or エネルギー摂取量/TA) and (PT=会議録除く)) | 8,334 |
| [#8](https://search-old.jamas.or.jp/index.php?module=Advanced&action=Search&sid=%238) | ((カロリー制限/TH or カロリー制限/TA) and (PT=会議録除く)) | 1,534 |
| [#9](https://search-old.jamas.or.jp/index.php?module=Advanced&action=Search&sid=%239) | ((間接的熱量測定/TH or 間接的熱量測定/TA or 間接熱量測定/TA) and (PT=会議録除く)) | 435 |
| [#10](https://search-old.jamas.or.jp/index.php?module=Advanced&action=Search&sid=%2310) | (熱量測定/TA) and (PT=会議録除く) | 424 |
| [#11](https://search-old.jamas.or.jp/index.php?module=Advanced&action=Search&sid=%2311) | (カロリー摂取/TA) and (PT=会議録除く) | 226 |
| [#12](https://search-old.jamas.or.jp/index.php?module=Advanced&action=Search&sid=%2312) | (エネルギー摂取/TA) and (PT=会議録除く) | 1,211 |
| [#13](https://search-old.jamas.or.jp/index.php?module=Advanced&action=Search&sid=%2313) | (低栄養/TA) and (PT=会議録除く) | 5,090 |
| [#14](https://search-old.jamas.or.jp/index.php?module=Advanced&action=Search&sid=%2314) | (低カロリー/TA) and (PT=会議録除く) | 344 |
| [#15](https://search-old.jamas.or.jp/index.php?module=Advanced&action=Search&sid=%2315) | (低エネルギー/TA) and (PT=会議録除く) | 758 |
| [#16](https://search-old.jamas.or.jp/index.php?module=Advanced&action=Search&sid=%2316) | (カロリー測定/TA) and (PT=会議録除く) | 11 |
| [#17](https://search-old.jamas.or.jp/index.php?module=Advanced&action=Search&sid=%2317) | (エネルギー測定/TA) and (PT=会議録除く) | 36 |
| [#18](https://search-old.jamas.or.jp/index.php?module=Advanced&action=Search&sid=%2318) | (等カロリーTA/AL) and (PT=会議録除く) | 0 |
| [#19](https://search-old.jamas.or.jp/index.php?module=Advanced&action=Search&sid=%2319) | (等エネルギー/TA) and (PT=会議録除く) | 21 |
| [#20](https://search-old.jamas.or.jp/index.php?module=Advanced&action=Search&sid=%2320) | (標準栄養/TA) and (PT=会議録除く) | 5 |
| [#21](https://search-old.jamas.or.jp/index.php?module=Advanced&action=Search&sid=%2321) | (標準カロリー/TA) and (PT=会議録除く) | 3 |
| [#22](https://search-old.jamas.or.jp/index.php?module=Advanced&action=Search&sid=%2322) | (標準エネルギー/TA) and (PT=会議録除く) | 1 |
| [#23](https://search-old.jamas.or.jp/index.php?module=Advanced&action=Search&sid=%2323) | #7 or #8 or #9 or #10 or #11 or #12 or #13 or #14 or #15 or #16 or #17 or #18 or #19 or #20 or #21 or #22 | 15,138 |
| [#24](https://search-old.jamas.or.jp/index.php?module=Advanced&action=Search&sid=%2324) | ランダム化比較試験/TH or 準ランダム化比較試験/TH or ランダム化/AL or 無作為化/AL or 比較試験/AL or 臨床試験/AL or プラセボ/AL or 対照/AL or コントロール/AL or 臨床研究/AL | 349,708 |
| [#25](https://search-old.jamas.or.jp/index.php?module=Advanced&action=Search&sid=%2325) | #6 and #23 and #24 | 35 |

Flow Diagram

Risk of Bias

28-day mortality

90-day mortality

Length of ICU stay

Duration of mechanical ventilation

All infectious complications

All adverse events

Vomiting

Forest Plot and Funnel Plot

28-day mortality

90-day mortality

Length of ICU stay

Duration of mechanical ventilation

All infectious complications

All adverse events

Vomiting

**CQ1-4 ：Should higher dose of protein above the standard (>1.2 g/kg/day) be administered in critically ill patients?**

Search Database

- MEDLINE (PubMed/Ovid)

Search Strategy and Hits:

PubMed search strategy（Search Date: April 22, 2023）

| #1 | "Intensive Care Units"[Mesh] OR "intensive care"[tiab] OR "Critical Illness"[Mesh] OR "critical care"[Mesh] OR "critically ill"[tiab] OR "critical care"[tiab] | 308,344 |
| --- | --- | --- |
| #2 | "Dietary Proteins/administration and dosage"[Mesh] OR "protein*"[tiab] OR "amino acid*"[tiab] OR "peptide*"[tiab] | 4,065,114 |
| #3 | intake[tiab] OR "requirement*"[tiab] OR dosage[tiab] OR "administration"[tiab] OR "dose*"[tiab] OR "deliver*"[tiab] | 3,519,307 |
| #4 | #1 AND #2 AND #3 | 3,658 |
| #5 | randomized controlled trial[pt] OR controlled clinical trial[pt] OR randomized[tiab] OR placebo[tiab] OR clinical trials as topic[mesh:noexp] OR randomly[tiab] OR trial[ti] NOT (animals [mh] NOT humans [mh]) | 1,409,883 |
| #6 | #4 AND #5 | 761 |

Search Database

- CENTRAL

Search Strategy and Hits

| #1 | MeSH descriptor: [Intensive Care Units] explode all trees | 5225 |
| --- | --- | --- |
| #2 | MeSH descriptor: [Critical Illness] explode all trees | 3218 |
| #3 | (“critical illness”):ti,ab,kw | 4441 |
| #4 | (“critically ill”):ti,ab,kw | 8214 |
| #5 | MeSH descriptor: [Critical Care] explode all trees | 2650 |
| #6 | ("critical care"):ti,ab,kw | 4859 |
| #7 | ("intensive care"):ti,ab,kw | 28987 |
| #8 | #1 OR #2 OR #3 OR #4 OR #5 OR #6 OR #7 | 35462 |
| #9 | MeSH descriptor: [Dietary Proteins] explode all trees and with qualifier(s): [administration & dosage - AD] | 2690 |
| #10 | ("protein*"):ti,ab,kw | 95002 |
| #11 | ("amino acid*"):ti,ab,kw | 7414 |
| #12 | ("peptide*"):ti,ab,kw | 23327 |
| #13 | #9 OR #10 OR #11 OR #12 | 117860 |
| #14 | ("intake"):ti,ab,kw | 63784 |
| #15 | ("requirement"):ti,ab,kw | 15556 |
| #16 | ("dosage"):ti,ab,kw | 230410 |
| #17 | ("administration"):ti,ab,kw | 386808 |
| #18 | ("dose*"):ti,ab,kw | 321061 |
| #19 | ("deliver*"):ti,ab,kw | 10084 |
| #20 | #14 OR #15 OR #16 OR #17 OR #18 OR #19 | 632256 |
| #21 | #8 AND #13 AND #20 | 1667 |

CENTRAL search strategy（Search Date: April 22, 2023）

Search Database

■Igaku Chuo Zasshi

Search Strategy and Hits

Igaku Chuo Zasshi search strategy（Search Date: April 22, 2023）

| #1 | (ICU/TH or “intensive care unit”/TA or ICU/TA) and (PT=会議録除く) | 55,436 |
| --- | --- | --- |
| #2 | (クリティカルケア/TH or クリティカルケア/TA or クリティカル・ケア/TA) and (PT=会議録除く) | 7,487 |
| #3 | 重症患者/TA | 5,959 |
| #4 | #1 or #2 or #3 | 65,531 |
| #5 | (((タンパク質/TH) or (タンパク質/TA))) and (PT=会議録除く) | 646,731 |
| #6 | ([Amino Acids]/TH or (アミノ酸/TA)) and (PT=会議録除く) | 71,131 |
| #7 | ((Peptides/TH) or (ペプチド/TA)) and (PT=会議録除く) | 285,632 |
| #8 | #5 or #6 or #7 | 753,273 |
| #9 | (摂取量/TA) and (PT=会議録除く) | 13,112 |
| #10 | (必要量/TA) and (PT=会議録除く) | 1,788 |
| #11 | (投与量/TA) and (PT=会議録除く) | 23,582 |
| #12 | #9 or #10 or #11 | 37,935 |
| #13 | ランダム化比較試験/TH or 準ランダム化比較試験/TH or ランダム化/AL or 無作為化/AL or 比較試験/AL or 臨床試験/AL or プラセボ/AL or 対照/AL or コントロール/AL or 臨床研究/AL | 349,708 |
| #14 | #4 AND #8　AND #12 AND #13 | 45 |

Flow Diagram

Risk of Bias

Short-term mortality

Length of ICU stay

Duration of mechanical ventilation

Infectious complications

Handgrip strength

Muscle mass reduction

Diarrhea

Forest Plot and　Funnel Plot

Short-term mortality

Length of ICU stay

Duration of mechanical ventilation

Infectious complications

Handgrip strength

Muscle mass reduction

Diarrhea

**CQ1-5：Should enteral nutrition be initiated within 48 hours following the start of treatment in critically ill patients?**

Search Database

- MEDLINE (PubMed/Ovid)

Search Strategy and Hits

PubMed search strategy （Search Date: April 21, 2023）

| #1 | "Intensive Care Units"[Mesh] OR “intensive care"[tiab] OR "Critical Illness"[Mesh] OR "critical care"[Mesh] OR “critically ill”[tiab] OR "critical care"[tiab] | 308312 |
| --- | --- | --- |
| #2 | "sepsis"[MeSH Terms] OR "postoperative care"[MeSH Terms] OR "wounds and injuries"[MeSH Terms] | 11911224 |
| #3 | #1 OR #2 | 1454848 |
| #4 | "Nutrition Therapy"[MeSH Terms] OR "Nutrition Therapy"[Title/Abstract] OR "feeding methods"[Title/Abstract] OR "feeding methods"[MeSH Terms] OR "nutritional support"[MeSH Terms] OR "nutritional support"[Title/Abstract] OR "enteral nutrition"[Title/Abstract] OR "enteral nutrition"[MeSH Terms] OR "Diet"[MeSH Terms] OR "Diet"[Title/Abstract] OR "energy intake"[MeSH Terms] OR "energy intake"[Title/Abstract] OR "enteral feeding"[Title/Abstract] OR "tube feeding"[Title/Abstract] | 640644 |
| #5 | "Time Factors"[MeSH Terms] OR "Early Enteral Nutrition"[Title/Abstract] OR "48hours"[Title/Abstract] OR "48 hours"[Title/Abstract] OR "24hours"[Title/Abstract] OR "24 hours"[Title/Abstract] OR "early nutrition*"[Title/Abstract] OR "early feeding"[Title/Abstract] OR "early EN"[Title/Abstract] | 1371036 |
| #6 | randomized controlled trial[pt] OR controlled clinical trial[pt] OR randomized[tiab] OR placebo[tiab] OR drug therapy[sh] OR randomly[tiab] OR trial[tiab] OR groups[tiab] NOT (animals [mh]  NOT humans [mh]) | 4990195 |
| #7 | #4 AND #2 AND #5 AND #6 | 1113 |

Search Database

- CENTRAL

Search Strategy and Hits

CENTRAL search strategy （Search Date: April 21, 2023）

| #1 | MeSH descriptor: [Intensive Care Units] explode all trees | 5225 |
| --- | --- | --- |
| #2 | MeSH descriptor: [Critical Illness] explode all trees | 3218 |
| #3 | (“critical illness”):ti,ab,kw | 4441 |
| #4 | (“critically ill”):ti,ab,kw | 8214 |
| #5 | MeSH descriptor: [Critical Care] explode all trees | 2650 |
| #6 | ("critical care"):ti,ab,kw | 4859 |
| #7 | ("intensive care"):ti,ab,kw | 28987 |
| #8 | #1 OR #2 OR #3 OR #4 OR #5 OR #6 OR #7 | 35462 |
| #9 | MeSH descriptor: [Sepsis] explode all trees | 6710 |
| #10 | MeSH descriptor: [Postoperative Care] explode all trees | 5478 |
| #11 | MeSH descriptor: [Wounds and Injuries] explode all tree | 34224 |
| #12 | #9 OR #10 OR #11 | 45846 |
| #13 | #8 OR #12 | 77846 |
| #14 | MeSH descriptor: [Nutrition Therapy] explode all trees | 12025 |
| #15 | ("Nutrition Therapy"):ti,ab,kw | 712 |
| #16 | ("feeding methods"):ti,ab,kw | 365 |
| #17 | MeSH descriptor: [Feeding Methods] explode all trees | 4035 |
| #18 | MeSH descriptor: [Nutritional Support] explode all trees | 4156 |
| #19 | ("nutritional support"):ti,ab,kw | 2277 |
| #20 | ("enteral nutrition"):ti,ab,kw | 4963 |
| #21 | MeSH descriptor: [Enteral Nutrition] explode all trees | 2179 |
| #22 | MeSH descriptor: [Diet] explode all trees | 25523 |
| #23 | (Diet):ti,ab,kw | 73355 |
| #24 | MeSH descriptor: [Energy Intake] explode all trees | 6440 |
| #25 | (“energy intake”):ti,ab,kw | 9188 |
| #26 | ("enteral feeding"):ti,ab,kw | 1610 |
| #27 | ("tube feeding"):ti,ab,kw | 801 |
| #28 | #14 OR #15 OR #16 OR #17 OR #18 OR #19 OR #20 OR #21 OR #22 OR #23 OR #24 OR #25 OR #26 OR #27 | 87810 |
| #29 | MeSH descriptor: [Time Factors] explode all trees | 72994 |
| #30 | ("Early Enteral Nutrition"):ti,ab,kw | 416 |
| #31 | (48hours):ti,ab,kw | 187 |
| #32 | ("48 hours"):ti,ab,kw | 18574 |
| #33 | (24hours):ti,ab,kw | 652 |
| #34 | ("24 hours"):ti,ab,kw | 41632 |
| #35 | ("early nutrition"):ti,ab,kw | 145 |
| #36 | ("early nutritional"):ti,ab,kw | 156 |
| #37 | ("early feeding"):ti,ab,kw | 366 |
| #38 | ("early EN"):ti,ab,kw | 67 |
| #39 | #29 OR #30 OR #31 OR #32 OR #33 OR #34 OR #35 OR #36 OR #37 OR #38 | 127041 |
| #40 | #13 AND #28 AND #39 | 926 |

Search Database

■Igaku Chuo Zasshi

**Search Strategy and Hits**

Igaku Chuo Zasshi search strategy （Search Date: April 21, 2023）

| #1 | (ICU/TH or “intensive care unit”/TA or ICU/TA) and (PT=会議録除く) | 55436 |
| --- | --- | --- |
| #2 | (集中治療/TH or 集中治療/TA) and (PT=会議録除く) | 15549 |
| #3 | (クリティカルケア/TH or クリティカルケア/TA or クリティカル・ケア/TA) and (PT=会議録除く) | 7487 |
| #4 | （重症患者/TA or敗血症/TA or 術後/TA or 外傷/TA or sepsis/TH or sepsis/TA) and (PT=会議録除く) | 407395 |
| #5 | #1 or #2 or #3 or #4 | 461960 |
| #6 | (栄養療法/TH or 栄養療法/TA栄養/TH or 栄養/TA) and (PT=会議録除く) | 105918 |
| #7 | (投与方法/TH or 投与方法/TA) and (PT=会議録除く) | 3860 |
| #8 | (栄養サポート/TH or 栄養サポート/TA) and (PT=会議録除く) | 23034 |
| #9 | (エネルギー摂取/TH or エネルギー摂取/TA) and (PT=会議録除く) | 1211 |
| #10 | (経腸栄養/TH or 経腸栄養/TA or 経管栄養/TH or 経管栄養/TA) and (PT=会議録除く) | 16142 |
| #11 | #6 or #7 or #8 or #9 or #10 | 115866 |
| #12 | (時期/TH or 時期/TA or タイミング/TH or タイミング/TA) and (PT=会議録除く) | 61527 |
| #13 | (早期経管栄養/TH or 早期経管栄養/TA or早期経腸栄養/TH or 早期経腸栄養/TA or 早期/TH or 早期/TA) and (PT=会議録除く) | 154682 |
| #14 | (24時間/TH or 24時間/TA or 48時間/TH or 48時間/TA) and (PT=会議録除く) | 33204 |
| #15 | #12 or #13 or #14 | 238187 |
| #16 | ランダム化比較試験/TH or 準ランダム化比較試験/TH or ランダム化/AL or 無作為化/AL or 比較試験/AL or 臨床試験/AL or プラセボ/AL or 対照/AL or コントロール/AL or 臨床研究/AL | 349708 |
| #17 | #5 AND #11 AND #15 AND #16 | 232 |

Flow Diagram

Risk of Bias

１．Mortality (short term mortality)

2．Length of ICU stay

３．Duration of mechanical ventilation

４．Infectious complications

５．Handgrip strength

６．All adverse events（diarrhea）

７．All adverse events（gastric retention）

Forest Plot and Funnel Plot

Mortality（short team mortality）

Length of ICU stay

Duration of mechanical ventilation

Infectious complications

Pneumonia

Sepsis/Bacteremia

Wound infection

Urinary tract infection

Handgrip strength

All adverse events

All adverse events（diarrhea）

All adverse events（gastric retention）

Forest Plot and　Funnel Plot

**（Subgroup analysis：Enteral nutrition initiated within 24 hours）**

Mortality（short team mortality）

Length of ICU stay

Duration of mechanical ventilation

Infectious complications

Pneumonia

Sepsis/Bacteremia

Wound infection

Urinary tract infection

Handgrip strength

All adverse events

All adverse events（diarrhea）

All adverse events（gastric retention）

**CQ1-6：Is enteral nutrition beneficial compared to no enteral nutrition or parenteral nutrition in critically ill patients with hemodynamic instability?**

Search Database

- MEDLINE (PubMed/Ovid)

Search Strategy and Hits

PubMed search strategy（Search Date: April 21, 2023）

| #1 | "shock"[MeSH Terms] OR "shock"[Title/Abstract] OR "hypotension"[MeSH Terms] OR "hypotension"[Title/Abstract] OR "catecholamines"[MeSH Terms] OR "catecholamine*"[Title/Abstract] OR "vasoconstrictor agents"[MeSH Terms] OR "vasoconstrictor agent*"[Title/Abstract] OR "hemodynamically unstable"[Title/Abstract] OR "hemodynamic instability"[Title/Abstract] | 610,279 |
| --- | --- | --- |
| #2 | ((((nutritional support[MeSH Terms]) OR (diet therapy[MeSH Terms])) OR (feeding method[MeSH Terms])) OR (nutrit*[Title/Abstract])) OR (feed*[Title/Abstract]) | 914,461 |
| #3 | randomized controlled trial [pt] OR controlled clinical trial [pt] OR randomized [tiab] OR placebo [tiab] OR clinical trials as topic [mesh:noexp] OR randomly [tiab] OR trial [ti] NOT(animals[mh] NOT humans [mh]) | 1,409,817 |
| #4 | #1 AND #2 AND #3 | 643 |

Search Database

- CENTRAL

Search Strategy and Hits

CENTRAL search strategy（Search Date: April 21, 2023）

| #1 | MeSH descriptor: [Shock] explode all trees | 3294 |
| --- | --- | --- |
| #2 | (shock):ti,ab,kw | 12827 |
| #3 | MeSH descriptor: [Hypotension] explode all trees | 4001 |
| #4 | (hypotension):ti,ab,kw | 18494 |
| #5 | MeSH descriptor: [Catecholamines] explode all trees | 11443 |
| #6 | (catecholamine*):ti,ab,kw | 4249 |
| #7 | MeSH descriptor: [“Vasoconstrictor Agents”] explode all trees | 2048 |
| #8 | (“vasoconstrictor agent*”):ti,ab,kw | 2076 |
| #9 | (“hemodynamically unstable”):ti,ab,kw | 155 |
| #10 | (“hemodynamic instability”):ti,ab,kw | 1245 |
| #11 | #1 OR #2 OR #3 OR #4 OR #5 OR #6 OR #7 OR #8 OR #9 OR#10 | 46342 |
| #12 | MeSH descriptor: [Nutritional Support] explode all trees | 4156 |
| #13 | MeSH descriptor: [Diet therapy] explode all trees | 7684 |
| #14 | MeSH descriptor: [Feeding Methods] explode all trees | 4035 |
| #15 | (nutrit*):ti,ab,kw | 52137 |
| #16 | (feed*):ti,ab,kw | 51360 |
| #17 | #12 OR #13 OR #14 OR #15 OR #16 | 98882 |
| #18 | #11 AND #17 | 1253 |

Search Database

■Igaku Chuo Zasshi

Search Strategy and Hits

Igaku Chuo Zasshi search strategy（Search Date: April 21, 2023）

| #1 | (ショック/TH or ショック/TA) and (PT=会議録除く) | 37,642 |
| --- | --- | --- |
| #2 | (低血圧/TH or 低血圧/TA) and (PT=会議録除く) | 14,028 |
| #3 | (Catecholamines/TH or catecholamines/TA or カテコラミン/TA) and (PT=会議録除く) | 26,100 |
| #4 | (血管収縮剤/TH or 血管収縮剤/TA) and (PT=会議録除く) | 44,287 |
| #5 | #1 OR #2 OR #3 OR #4 | 106,163 |
| #6 | (経腸栄養/TH or 経腸栄養/TA) and (PT=会議録除く) | 15,873 |
| #7 | (((ランダム化比較試験/TH or 準ランダム化比較試験/TH or ランダム化/AL or 無作為化/AL or 比較試験/AL or 臨床試験/AL or プラセボ/AL or 対照/AL or コントロール/AL or 臨床研究/AL))) | 349,708 |
| #8 | #5 AND #6 AND #7 | 35 |

Flow Diagram

l

Risk of Bias

Hospital Mortality

Length of ICU stay (days)

Duration of mechanical ventilation-free days

ICU-acquired infection

Ventilator-associated pneumonia

Mesenteric ischemia

Vomiting

Forest Plot and Funnel Plot (Main)

Hospital Mortality

Length of ICU stay (days)

Duration of mechanical ventilation-free days

ICU-acquired infection

Ventilator-associated pneumonia

Mesenteric ischemia

Vomiting

Forest Plot and Funnel Plot
（Subgroup analysis: Fixed-effect model）

Hospital Mortality

Length of ICU stay (days)

Duration of mechanical ventilation-free days

ICU-acquired infection

Ventilator-associated pneumonia

Mesenteric ischemia

Vomiting

**CQ1-7：Should supplemental parenteral nutrition be initiated in critically ill patients receiving insufficient amounts of enteral nutrition?**

Search Database

■ MEDLINE (PubMed)

Search Strategy and Hits:

PubMed search strategy （Search Date: April 21, 2023）

| #1 | "Intensive Care Units"[Mesh] OR “intensive care"[tiab] OR "Critical Illness"[Mesh] OR "critical care"[Mesh] OR “critically ill”[tiab] OR "critical care"[tiab] | 308,344 |
| --- | --- | --- |
| #2 | "Sepsis"[Mesh] OR "sepsis"[tiab] OR "septic"[tiab] | 237,305 |
| #3 | "Shock"[Mesh] OR "shock"[tiab] | 244,188 |
| #4 | "Systemic Inflammatory Response Syndrome"[Mesh] OR "systemic inflammatory response syndrome"[tiab] OR "SIRS" [tiab] | 153,455 |
| #5 | "Multiple Organ Failure"[Mesh] OR "organ failure"[tiab] OR "organ dysfunction"[tiab] OR "MODS"[tiab] OR "MOF"[tiab] | 59,295 |
| #6 | "Wounds and Injuries"[Mesh] OR "trauma*"[tiab] OR "burn*"[tiab] OR "injur*"[tiab] | 1,887,448 |
| #7 | "Specialties, Surgical"[Mesh] OR "Surgical Procedures, Operative"[Mesh] OR "surgery"[tiab] OR "surgical"[tiab] | 4,684,457 |
| #8 | "Respiratory distress syndrome"[Mesh] OR "acute respiratory distress syndrome"[tiab] OR "ARDS"[tiab] OR "Respiratory Insufficiency"[Mesh] OR "respiratory failure"[tiab] | 142,825 |
| #9 | "Pancreatitis"[Mesh] OR "pancreatitis"[tiab] | 75,947 |
| #10 | "Heart failure"[Mesh] OR "heart failure"[tiab] | 251,483 |
| #11 | #1 OR #2 OR #3 OR #4 OR #5 OR #6 OR #7 OR #8 OR #9 OR #10 | 6,761,746 |
| #12 | "Enteral nutrition"[Mesh] OR "enteral nutrition"[tiab] OR "enteral feed*"[tiab] OR "enterally fed"[tiab] OR "enteric nutrition"[tiab] OR "enteric feed*"[tiab] OR "tubefeed*"[tiab] OR "tube feed*"[tiab] OR "jejunal tube"[tiab] OR "j-tube"[tiab] OR "jejunal feed*"[tiab] OR "gastric tube"[tiab] OR "g-tube"[tiab] OR "gastric feed*"[tiab] OR "postpyloric"[tiab] OR "EN"[tiab] | 102,188 |
| #13 | "Parenteral nutrition"[Mesh] OR "parenteral nutrition"[tiab] OR "parenteral feed*"[tiab] OR "parenterally fed"[tiab] OR "intravenous nutrition"[tiab] OR "intravenously fed"[tiab] OR "IV fed"[tiab] OR "PN"[tiab] | 56,674 |
| #14 | #12 AND #13 | 7,823 |
| #15 | "supplement*"[tiab] AND #13 | 3,339 |
| #16 | #14 OR #15 | 10,096 |
| #17 | randomized controlled trial[pt] OR controlled clinical trial[pt] OR randomized[tiab] OR placebo[tiab] OR drug therapy[sh] OR randomly[tiab] OR trial[tiab] OR groups[tiab] NOT (animals[mh] NOT humans[mh]) | 4,990,195 |
| #18 | #11 AND #16 AND #17 | 1,690 |

Search Database
■ CENTRAL

Search Strategy and Hits: CENTRAL search strategy (Search Date: April 21, 2023）

| #1 | MeSH descriptor: [Intensive Care Units] explode all trees | 5225 |
| --- | --- | --- |
| #2 | MeSH descriptor: [Critical Illness] explode all trees | 3218 |
| #3 | (“critical illness”):ti,ab,kw | 4441 |
| #4 | (“critically ill”):ti,ab,kw | 8214 |
| #5 | MeSH descriptor: [Critical Care] explode all trees | 2650 |
| #6 | ("critical care"):ti,ab,kw | 4859 |
| #7 | ("intensive care"):ti,ab,kw | 28987 |
| #8 | #1 OR #2 OR #3 OR #4 OR #5 OR #6 OR #7 | 35462 |
| #9 | MeSH descriptor: [Sepsis] explode all trees | 6710 |
| #10 | ("sepsis" OR "septic"):ti,ab,kw | 15642 |
| #11 | MeSH descriptor: [Shock] explode all trees | 3294 |
| #12 | ("shock"):ti,ab,kw | 12827 |
| #13 | MeSH descriptor: [Systemic Inflammatory Response Syndrome] explode all trees | 7277 |
| #14 | ("systemic inflammatory response syndrome" OR "SIRS"):ti,ab,kw | 1602 |
| #15 | MeSH descriptor: [Multiple Organ Failure] explode all trees | 640 |
| #16 | ("organ failure" OR "organ dysfunction" OR "MODS" OR "MOF"):ti,ab,kw | 5622 |
| #17 | MeSH descriptor: [Wounds and Injuries] explode all trees | 34224 |
| #18 | ("trauma*" OR "burn*" OR "injur*"):ti,ab,kw | 22886 |
| #19 | MeSH descriptor: [Specialties, Surgical] explode all trees | 3675 |
| #20 | MeSH descriptor: [Surgical Procedures, Operative] explode all trees | 164459 |
| #21 | ("surgery" OR "surgical"):ti,ab,kw | 293745 |
| #22 | MeSH descriptor: [Respiratory Distress Syndrome] explode all trees | 3262 |
| #23 | ("acute respiratory distress syndrome" OR "ARDS"):ti,ab,kw | 3205 |
| #24 | MeSH descriptor: [Respiratory Insufficiency] explode all trees | 3676 |
| #25 | ("respiratory failure"):ti,ab,kw | 5838 |
| #26 | MeSH descriptor: [Pancreatitis] explode all trees | 1883 |
| #27 | ("pancreatitis"):ti,ab,kw | 5266 |
| #28 | MeSH descriptor: [Heart Failure] explode all trees | 14340 |
| #29 | ("heart failure"):ti,ab,kw | 34829 |
| #30 | #8 OR #9 OR #10 OR #11 OR #12 OR #13 OR #14 OR #15 OR #16 OR #17 OR #18 OR #19 OR #20 OR #21 OR #22 OR #23 OR #24 OR #25 OR #26 OR #27 OR #28 OR #29 | 463875 |
| #31 | MeSH descriptor: [Enteral Nutrition] explode all trees | 2179 |
| #32 | ("enteral nutrition" OR "enteral feed*" OR "enterally fed" OR "enteric nutrition" OR "enteric feed*" OR "tubefeed*" OR "tube feed*" OR "jejunal tube" OR "j-tube" OR "jejunal feed*" OR "gastric tube" OR "g-tube" OR "gastric feed*" OR "postpyloric" OR "EN"):ti,ab,kw | 23108 |
| #33 | #31 OR #32 | 23108 |
| #34 | MeSH descriptor: [Parenteral Nutrition] explode all trees | 1949 |
| #35 | ("parenteral nutrition" OR "parenteral feed*" OR "parenterally fed" OR "intravenous nutrition" OR "intravenously fed" OR "IV fed" OR "PN"):ti,ab,kw | 6176 |
| #36 | #34 OR #35 | 6176 |
| #37 | #33 AND #36 | 1264 |
| #38 | ("supplement*"):ti,ab,kw | 19382 |
| #39 | #36 AND #38 | 149 |
| #40 | #37 OR #39 | 1354 |
| #41 | #30 AND #40 | 913 |

Search Database

- Igaku Chuo Zasshi

Search Strategy and Hits:

Igaku Chuo Zasshi search strategy (Search Date: April 21, 2023)

| #1 | (ICU/TH or “intensive care unit”/TA or ICU/TA) and (PT=会議録除く) | 55,436 |
| --- | --- | --- |
| #2 | (集中治療/TA) and (PT=会議録除く) | 12,382 |
| #3 | (クリティカルケア/TH or クリティカルケア/TA or クリティカル・ケア/TA) and (PT=会議録除く) | 7,487 |
| #4 | 重症患者/TA | 5,959 |
| #5 | (敗血症/TH or 敗血症/TA) and (PT=会議録除く) | 27,247 |
| #6 | (ショック/TH or ショック/TA) and (PT=会議録除く) | 37,642 |
| #7 | (全身性炎症反応症候群/TH or SIRS/TA) and (PT=会議録除く) | 22,013 |
| #8 | (多臓器不全/TH or　臓器障害/TA) and (PT=会議録除く) | 9,547 |
| #9 | (創傷と損傷/TH or 外傷/TA or 熱傷/TA or 損傷/TA) and (PT=会議録除く) | 290,294 |
| #10 | (外科専門分野/TH or 外科手術/TH or 手術/TA) and (PT=会議録除く) | 1,023,859 |
| #11 | (呼吸窮迫症候群/TH or 呼吸窮迫症候群/TA or 呼吸促迫/TA or ARDS/TA or　呼吸不全/TH or 呼吸不全/TA) and (PT=会議録除く) | 34,185 |
| #12 | (膵炎/TH or 膵炎/TA) and (PT=会議録除く) | 21,529 |
| #13 | (心不全/TH or 心不全/TA) and (PT=会議録除く) | 53,690 |
| #14 | #1 or #2 or #3 or #4 or #5 or #6 or #7 or #8 or #9 or #10 or #11 or #12 or #13 | 1,340,492 |
| #15 | (経腸栄養/TH or 経腸栄養/TA or 経管栄養/TA) and (PT=会議録除く) | 16,142 |
| #16 | (胃ゾンデ/TH or 胃管/TA) and (PT=会議録除く) | 5,271 |
| #17 | (空腸栄養/TA or 空腸管/TA or 幽門後栄養/TA ) and (PT=会議録除く) | 80 |
| #18 | (静脈栄養/TH or 静脈栄養/TA or 経静脈栄養/TA or 中心静脈栄養/TH or 中心静脈栄養/TA) and (PT=会議録除く) | 11,551 |
| #19 | (#15 or #16 or #17) and #18 | 3,851 |
| #20 | (補助/TA or 追加/TA or 補充/TA or 補足/TA or 補完/TA or 付加/TA or 添加/TA) and #18 | 562 |
| #21 | #19 or #20 | 4,190 |
| #22 | ランダム化比較試験/TH or 準ランダム化比較試験/TH or ランダム化/AL or 無作為化/AL or 比較試験/AL or 臨床試験/AL or プラセボ/AL or 対照/AL or コントロール/AL or 臨床研究/AL | 349,708 |
| #23 | #14 and #21 and #22 | 168 |

Flow Diagram

Risk of bias

Short term mortality

Long term mortality

Duration of mechanical ventilation

Length of ICU stay

Infectious complications (bloodstream infection)

Infectious complications (all-cause)

SF-36 physical functioning domain

Adverse events

Forest plot

Short term mortality

Long term mortality

Duration of mechanical ventilation

Length of ICU stay

Infectious complications (bloodstream infection)

Infectious complications (all-cause)

SF-36 physical functioning domain

Adverse events

**CQ1-8: Should post-pyloric feeding be used rather than gastric feeding for enteral nutrition in critically ill patients?**

Search Database

- MEDLINE (PubMed/Ovid)

Search Strategy and Hits:

PubMed search strategy （Search Date: April 17, 2023）

| #1 | “Intensive Care Units”[Mesh] OR “intensive care”[tiab] OR “Critical Illness”[Mesh] OR “critical care”[Mesh] OR “critically ill”[tiab] OR “critical care”[tiab] OR pancreatitis[Mesh] OR pancreatitis[tiab] OR burns[Mesh] OR burn*[tiab] OR trauma[Mesh] OR trauma[tiab] | 1577364 |
| --- | --- | --- |
| #2 | “randomized controlled trial”[pt] OR “controlled clinical trial”[pt] OR randomized[tiab] OR placebo[tiab] OR “drug therapy”[sh] OR randomly[tiab] OR trial[tiab] OR groups[tiab] NOT (animals [mh]  NOT humans [mh]) | 4986010 |
| #3 | “Enteral Nutrition”[Mesh] OR “Enteral Nutrition”[tiab] OR “enteral feed*”[tiab] | 28736 |
| #4 | gastr*[tiab] OR nasogastr*[tiab] OR intragastr*[tiab] | 735556 |
| #5 | intestines[Mesh] OR duoden*[tiab] OR nasoduoden* [tiab] OR jejun*[tiab] OR nasojejun*[tiab] OR enter*[tiab] OR nasoenter*[tiab] OR intestine*[tiab] OR "small bowel*"[tiab] OR postpylor*[tiab] OR transpylor*[tiab] OR post-pylor*[tiab] OR trans-pylor*[tiab] | 1008775 |
| #6 | #1 AND #2 AND #3 AND #4 AND #5 | 778 |

Search Database

- CENTRAL

Search Strategy and Hits:

CENTRAL search strategy （Search Date: April 18, 2023）

| #1 | MeSH descriptor: [Intensive Care Units] explode all trees | 5225 |
| --- | --- | --- |
| #2 | MeSH descriptor: [Critical Illness] explode all trees | 3218 |
| #3 | (“critical illness”):ti,ab,kw | 4441 |
| #4 | (“critically ill”):ti,ab,kw | 8214 |
| #5 | MeSH descriptor: [Critical Care] explode all trees | 2650 |
| #6 | ("critical care"):ti,ab,kw | 4859 |
| #7 | ("intensive care"):ti,ab,kw | 28987 |
| #8 | MeSH descriptor: [Pancreatitis] explode all trees | 1883 |
| #9 | (pancreatitis):ti,ab,kw | 5266 |
| #10 | MeSH descriptor: [Burns] explode all trees | 2093 |
| #11 | (burn*):ti,ab,kw | 12640 |
| #12 | MeSH descriptor: [Wounds and Injuries] explode all trees | 34224 |
| #13 | (trauma):ti,ab,kw | 18467 |
| #14 | #1 OR #2 OR #3 OR #4 OR #5 OR #6 OR #7 OR #8 OR #9 OR #10 OR #11 OR #12 OR #13 | 96453 |
| #15 | MeSH descriptor: [Enteral Nutrition] explode all trees | 2179 |
| #16 | ("enteral nutrition"):ti,ab,kw | 4963 |
| #17 | ("enteral feed*"):ti,ab,kw | 150 |
| #18 | #15 OR #16 OR #17 | 5042 |
| #19 | (gastr*):ti,ab,kw | 89161 |
| #20 | (nasogastr*):ti,ab,kw | 2601 |
| #21 | (intragastr*):ti,ab,kw | 1902 |
| #22 | #19 OR #20 OR #21 | 90611 |
| #23 | MeSH descriptor: [Intestines] explode all trees | 7814 |
| #24 | (duoden*):ti,ab,kw | 8252 |
| #25 | (nasoduoden*):ti,ab,kw | 112 |
| #26 | (jejun*):ti,ab,kw | 1857 |
| #27 | (nasojejun*):ti,ab,kw | 240 |
| #28 | (enter*):ti,ab,kw | 50250 |
| #29 | (nasoenter*):ti,ab,kw | 117 |
| #30 | (intestine*):ti,ab,kw | 14536 |
| #31 | ("small bowel*"):ti,ab,kw | 1959 |
| #32 | (postpylor*):ti,ab,kw | 114 |
| #33 | (transpylor*):ti,ab,kw | 65 |
| #34 | (post-pylor*):ti,ab,kw | 72 |
| #35 | (trans-pylor*):ti,ab,kw | 7 |
| #36 | #23 OR #24 OR #25 OR #26 OR #27 OR #28 OR #29 OR #30 OR #31 OR #32 OR #33 OR #34 OR #35 | 74841 |
| #37 | #14 AND #18 AND #22 AND #36 | 731 |

Search Database

■Igaku Chuo Zasshi

Search Strategy and Hits:

Igaku Chuo Zasshi search strategy（Search Date: April 23, 2023）

| #1 | (ICU/TH or “intensive care unit”/TA or ICU/TA) and (PT=会議録除く) | 55436 |
| --- | --- | --- |
| #2 | (クリティカルケア/TH or クリティカルケア/TA or クリティカル・ケア/TA or 集中治療/TA) and (PT=会議録除く) | 16525 |
| #3 | 重症患者/TA | 5959 |
| #4 | (熱傷/TH or 熱傷/TA) and (PT=会議録除く) | 11626 |
| #5 | (創傷と損傷/TH or 外傷/TA) and (PT=会議録除く) | 256932 |
| #6 | (膵炎/TH or 膵炎/TA) and (PT=会議録除く) | 21529 |
| #7 | #1 or #2 or #3 or #4 or #5 or #6 | 346279 |
| #8 | ランダム化比較試験/TH or 準ランダム化比較試験/TH or ランダム化/AL or 無作為化/AL or 比較試験/AL or 臨床試験/AL or プラセボ/AL or 対照/AL or コントロール/AL or 臨床研究/AL | 349708 |
| #9 | (経腸栄養/TH or 経腸栄養/AL or 経腸栄養/TA or 経鼻栄養/AL or 経鼻栄養/TA) and (PT=会議録除く) | 15957 |
| #10 | (胃/TH or 胃/AL or 胃/TA) and (PT=会議録除く) | 200909 |
| #11 | (十二指腸/TH or 十二指腸/AL or 十二指腸/TA or 小腸/TH or 小腸/AL or 小腸/TA or 空腸/TH or 空腸/AL or 空腸/TA or 幽門/TH or 幽門/AL or 幽門/TA) and (PT=会議録除く) | 92751 |
| #12 | #7 and #8 and #9 and #10 and #11 | 15 |

Flow Diagram

Risk of Bias

All-cause mortality

Length of ICU stay

Duration of mechanical ventilation

Pneumonia (ventilator-associated or aspiration)

The time from ICU admission to initiation of enteral nutrition

Diarrhea

Vomiting

Forest Plot and Funnel Plot

All-cause mortality

Length of ICU stay

Duration of mechanical ventilation

Pneumonia (ventilator-associated or aspiration)

The time from ICU admission to initiation of enteral nutrition

Diarrhea

Vomiting

**CQ1-9: Should continuous feeding rather than intermittent feeding be used for gastric enteral nutrition in critically ill patients?**

Search Database

- MEDLINE (PubMed/Ovid)

Search Strategy and Hits:

PubMed search strategy（Search Date: April 21, 2023）

| #1 | "Intensive Care Units"[MeSH Terms] OR "intensive care"[Title/Abstract] OR "Critical Illness"[MeSH Terms] OR "critical care"[MeSH Terms] OR "critically ill"[Title/Abstract] OR "critical care"[Title/Abstract] | 308,312 |
| --- | --- | --- |
| #2 | "sepsis"[MeSH Terms] OR "shock, septic"[MeSH Terms] OR "bacteremia"[MeSH Terms] OR "sepsis"[Title/Abstract] OR "septic shock"[Title/Abstract] OR "bacteremia"[Title/Abstract] | 226,604 |
| #3 | "Systemic Inflammatory Response Syndrome"[MeSH Terms] OR "Systemic Inflammatory Response Syndrome"[Title/Abstract] OR "SIRS"[Title/Abstract] | 153,443 |
| #4 | "Multiple Organ Failure"[MeSH Terms] OR "organ failure*"[Title/Abstract] OR "organ dysfunction"[Title/Abstract] OR "MOF"[Title/Abstract] OR "MODS"[Title/Abstract] | 59,969 |
| #5 | "burns"[MeSH Terms] OR "Burn Units"[MeSH Terms] OR "burn"[Title/Abstract] OR "burn unit*"[Title/Abstract] | 76,729 |
| #6 | "Multiple Trauma"[MeSH Terms] OR "Brain Injuries"[MeSH Terms] OR "brain injuries, traumatic"[MeSH Terms] OR "Multiple Trauma"[Title/Abstract] OR "brain injury"[Title/Abstract] OR "traumatic brain injury"[Title/Abstract] OR "head trauma"[Title/Abstract] OR "head injury"[Title/Abstract] | 148,270 |
| #7 | "Respiratory Distress Syndrome"[MeSH Terms] OR "Acute Lung Injury"[MeSH Terms] OR "acute respiratory distress syndrome"[Title/Abstract] OR "Acute Lung Injury"[Title/Abstract] OR "ARDS"[Title/Abstract] OR "ALI"[Title/Abstract] | 70,311 |
| #8 | "respiration, artificial"[MeSH Terms] OR "invasive mechanical ventilation"[Title/Abstract] OR "mechanical ventilation"[Title/Abstract] OR "mechanically ventilated"[All Fields] OR "IMV"[Title/Abstract] OR "MV"[Title/Abstract] | 201,341 |
| #9 | "surgical procedures, operative"[MeSH Terms] OR "Postoperative Period"[MeSH Terms] OR "surgery"[Title/Abstract] OR "surgical*"[Title/Abstract] OR "postoperative"[Title/Abstract] | 4,694,791 |
| #10 | (((((((((#1) OR (#2)) OR (#3)) OR (#4)) OR (#5)) OR (#6)) OR (#7)) OR (#8)) OR (#9) | 5,426,390 |
| #11 | "enteral nutrition"[MeSH Terms] OR "enteral nutrition"[Title/Abstract] OR "enteral feeding"[Title/Abstract] OR "gastric"[Title/Abstract] | 311,744 |
| #12 | "continuous"[Title/Abstract] OR "intermittent"[Title/Abstract] OR "bolus"[Title/Abstract] | 588,073 |
| #13 | ("randomized controlled trial"[Publication Type] OR "controlled clinical trial"[Publication Type] OR "randomized"[Title/Abstract] OR "placebo"[Title/Abstract] OR "drug therapy"[MeSH Subheading] OR "randomly"[Title/Abstract] OR "trial"[Title/Abstract] OR "groups"[Title/Abstract]) NOT ("animals"[MeSH Terms] NOT "humans"[MeSH Terms]) | 4,989,795 |
| #14 | (((#10) AND (#11)) AND (#12)) AND (#13) | 1,279 |

Search Database

- CENTRAL

Search Strategy and Hits:

CENTRAL search strategy（Search Date: April 21, 2023）

| #1 | MeSH descriptor: [Enteral Nutrition] explode all trees | 2,179 |
| --- | --- | --- |
| #2 | (enteral nutrition):ti,ab,kw | 5,603 |
| #3 | (enteral feeding):ti,ab,kw | 3,763 |
| #4 | (gastric):ti,ab,kw | 29,594 |
| #5 | #1 OR #2 OR #3 OR #4 | 35,114 |
| #6 | MeSH descriptor: [Intensive Care Units] explode all trees | 5,225 |
| #7 | MeSH descriptor: [Critical Illness] explode all trees | 3,218 |
| #8 | (critical illness):ti,ab,kw | 5,670 |
| #9 | ("critically ill"):ti,ab,kw | 8,214 |
| #10 | MeSH descriptor: [Critical Care] explode all trees | 2,650 |
| #11 | (critical care):ti,ab,kw | 13,220 |
| #12 | ("intensive care"):ti,ab,kw | 28,987 |
| #13 | MeSH descriptor: [Sepsis] explode all trees | 6,710 |
| #14 | MeSH descriptor: [Shock, Septic] explode all trees | 1,236 |
| #15 | MeSH descriptor: [Bacteremia] explode all trees | 1,304 |
| #16 | (sepsis):ti,ab,kw | 13,042 |
| #17 | (septic shock):ti,ab,kw | 3,776 |
| #18 | (bacteremia):ti,ab,kw | 2,769 |
| #19 | MeSH descriptor: [Systemic Inflammatory Response Syndrome] explode all trees | 7,277 |
| #20 | (Systemic Inflammatory Response Syndrome):ti,ab,kw | 1,553 |
| #21 | (SIRS):ti,ab,kw | 856 |
| #22 | MeSH descriptor: [Multiple Organ Failure] explode all trees | 640 |
| #23 | (organ failure):ti,ab,kw | 8,079 |
| #24 | (organ dysfunction):ti,ab,kw | 3,701 |
| #25 | (MOF):ti,ab,kw | 195 |
| #26 | (MODS):ti,ab,kw | 316 |
| #27 | MeSH descriptor: [Burns] explode all trees | 2,093 |
| #28 | MeSH descriptor: [Burn Units] explode all trees | 49 |
| #29 | (burn):ti,ab,kw | 4,696 |
| #30 | (burn unit):ti,ab,kw | 408 |
| #31 | MeSH descriptor: [Multiple Trauma] explode all trees | 310 |
| #32 | MeSH descriptor: [Brain Injuries] explode all trees | 3,175 |
| #33 | MeSH descriptor: [Brain Injuries, Traumatic] explode all trees | 1,336 |
| #34 | ("Multiple Trauma"):ti,ab,kw | 592 |
| #35 | ("brain injury"):ti,ab,kw | 6,865 |
| #36 | ("traumatic brain injury"):ti,ab,kw | 4,317 |
| #37 | ("head trauma"):ti,ab,kw | 611 |
| #38 | ("head injury"):ti,ab,kw | 1,556 |
| #39 | MeSH descriptor: [Respiratory Distress Syndrome] explode all trees | 3,262 |
| #40 | MeSH descriptor: [Acute Lung Injury] explode all trees | 697 |
| #41 | (acute respiratory distress syndrome):ti,ab,kw | 3,264 |
| #42 | (acute lung injury):ti,ab,kw | 2,522 |
| #43 | (ARDS):ti,ab,kw | 2,543 |
| #44 | (ALI):ti,ab,kw | 2,231 |
| #45 | MeSH descriptor: [Respiration, Artificial] explode all trees | 8,194 |
| #46 | (invasive mechanical ventilation):ti,ab,kw | 2,576 |
| #47 | (mechanical ventilation):ti,ab,kw | 13,085 |
| #48 | (mechanically ventilated):ti,ab,kw | 3,188 |
| #49 | (IMV):ti,ab,kw | 274 |
| #50 | (MV):ti,ab,kw | 2,812 |
| #51 | MeSH descriptor: [Surgical Procedures, Operative] explode all trees | 164,459 |
| #52 | MeSH descriptor: [Postoperative Period] explode all trees | 8,312 |
| #53 | (Surgery):ti,ab,kw | 259,689 |
| #54 | (surgical):ti,ab,kw | 119,000 |
| #55 | (postoperative):ti,ab,kw | 142,964 |
| #56 | #6 OR #7 OR #8 OR #9 OR #10 OR #11 OR #12 | 41,404 |
| #57 | #13 OR #14 OR #15 OR #16 OR #17 OR #18 | 18,226 |
| #58 | #19 OR #20 OR #21 OR #22 OR #23 OR #24 OR #25 OR #26 | 17,532 |
| #59 | #27 OR #28 OR #29 OR #30 | 5,296 |
| #60 | #31 OR #32 OR #33 OR #34 OR #35 OR #36 OR #37 OR #38 | 9,490 |
| #61 | #39 OR #40 OR #41 OR #42 OR #43 OR #44 | 8,709 |
| #62 | #45 OR #46 OR #47 OR #48 OR #49 OR #50 | 21,679 |
| #63 | #51 OR #52 OR #53 OR #54 OR #55 | 375,298 |
| #64 | #56 OR #57 OR #58 OR #59 OR #59 OR #60 OR #61 OR #62 OR #63 | 441,805 |
| #65 | (continuous):ti,ab,kw | 63,158 |
| #66 | (intermittent):ti,ab,kw | 16,889 |
| #67 | (bolus):ti,ab,kw | 18,791 |
| #68 | #65 OR #66 OR #67 | 89,559 |
| #69 | #5 AND #64 AND #68 | **1,143** |

- Igaku Chuo Zasshi

Search Strategy and Hits:

Igaku Chuo Zasshi search strategy（Search Date: April 21, 2023）

| #1 | (ICU/TH or ICU/TA) and (PT=会議録除く) | 55,401 |
| --- | --- | --- |
| #2 | (クリティカルケア/TH or クリティカルケア/TA) and (PT=会議録除く) | 7,420 |
| #3 | (重症患者/TA) and (PT=会議録除く) | 3,899 |
| #4 | ((ショック-敗血症性/TH or 敗血症性ショック/TA)) and (PT=会議録除く) | 7,380 |
| #5 | ((全身性炎症反応症候群/TH or SIRS/TA)) and (PT=会議録除く) | 22,013 |
| #6 | ((多臓器不全/TH or 多臓器不全/TA)) and (PT=会議録除く) | 6,918 |
| #7 | ((熱傷/TH or 熱傷/TA)) and (PT=会議録除く) | 11,626 |
| #8 | ((多発性外傷/TH or 多発外傷/TA)) and (PT=会議録除く) | 2,897 |
| #9 | ((呼吸不全/TH or 呼吸不全/TA)) and (PT=会議録除く) | 24,527 |
| #10 | ((術後期/TH or 術後/TA)) and (PT=会議録除く) | 337,542 |
| #11 | #1 or #2 or #3 or #4 or #5 or #6 or #7 or #8 or #9 or #10 | 446,818 |
| #12 | ((経腸栄養/TH or 経腸栄養/TA)) and (PT=会議録除く) | 14,354 |
| #13 | (持続投与/TA) and (PT=会議録除く) | 3,691 |
| #14 | (間欠投与/TA) and (PT=会議録除く) | 449 |
| #15 | #12 or #13 or #14 | 18,360 |
| #16 | ランダム化比較試験/TH or 準ランダム化比較試験/TH or ランダム化/AL or 無作為化/AL or 比較試験/AL or 臨床試験/AL or プラセボ/AL or 対照/AL or コントロール/AL or 臨床研究/AL | 349,708 |
| #17 | #11 and #15 and #16 | 530 |

Flow Diagram

Risk of Bias

Mortality

Length of ICU stay

Duration of mechanical ventilation

Infectious complications

Diarrhea

Muscle mass loss

Vomiting

Forest Plot and Funnel Plot

Mortality

Length of ICU stay

Duration of mechanical ventilation

Infectious complications

Diarrhea

Muscle mass loss

Vomiting

**CQ2-1：Should omega-3 fatty acid-enriched enteral nutrition be administered to critically ill patients?**

Search database

- MEDLINE (PubMed/Ovid)

Search Formula and Hit Count

PubMed search strategy （Date of Search: April 16, 2023）

| #1 | "Intensive Care Units"[Mesh] OR “intensive care"[tiab] OR "Critical Illness"[Mesh] OR "critical care"[Mesh] OR “critically ill”[tiab] OR "critical care"[tiab] OR ICU[tiab] | 324112 |
| --- | --- | --- |
| #2 | “Enteral Nutrition”[Mesh] | 21807 |
| #3 | “Enteral Nutrition”[tiab] | 10787 |
| #4 | “Fatty Acids, Omega-3” [Mesh] | 28931 |
| #5 | "omega-3"[tiab] | 19552 |
| #6 | eicosapentaenoic[tiab] | 12027 |
| #7 | EPA[tiab] | 20142 |
| #8 | docosahexaenoic[tiab] | 16076 |
| #9 | DHA[tiab] | 17568 |
| #10 | “alpha-linolenic Acid” [Mesh] | 3251 |
| #11 | “alpha-linolenic” [tiab] | 6193 |
| #12 | ALA[tiab] | 43420 |
| #13 | "Fish Oils"[MESH] | 32280 |
| #14 | "Fish Oils"[tiab] | 1643 |
| #15 | “n 3 fatty acids” [tiab] | 4391 |
| #16 | #2 OR #3 OR #4 OR #5 OR #6 OR #7 OR #8 OR #9 OR #10 OR #11 OR #12 OR #13 OR #14 OR #15 OR #16 | 132725 |
| #17 | randomized controlled trial[pt] OR controlled clinical trial[pt] OR randomized[tiab] OR placebo[tiab] OR drug therapy[sh] OR randomly[tiab] OR trial[tiab] OR groups[tiab] NOT (animals [mh]  NOT humans [mh]) | 4985825 |
| #18 | #1 AND #16 AND #17 | 1999 |

Search database

- CENTRAL

Search Formula and Hit Count

CENTRAL search strategy （Date of Search: April 16, 2023）

| #1 | [mh ”Intensive Care Units”] | 5225 |
| --- | --- | --- |
| #2 | [mh “Critical Illness”] | 3218 |
| #3 | (“Critical Illness”):ti,ab,kw | 4441 |
| #4 | (“Critically Ill”):ti,ab,kw | 8214 |
| #5 | [mh ”Critical Care”] | 2650 |
| #6 | ("critical care"):ti,ab,kw | 4859 |
| #7 | ("intensive care"):ti,ab,kw | 28987 |
| #8 | #1 OR #2 OR #3 OR #4 OR #5 OR #6 OR #7 | 35462 |
| #9 | [mh ”Fatty Acids, Omega-3”] | 3780 |
| #10 | (Omega-3):ti,ab,kw | 6833 |
| #11 | ("n 3 fatty acids"):ti,ab,kw | 926 |
| #12 | ("eicosapentaenoic"):ti,ab,kw | 2981 |
| #13 | (EPA):ti,ab,kw | 3078 |
| #14 | ("docosahexaenoic"):ti,ab,kw | 3396 |
| #15 | (DHA):ti,ab,kw | 3536 |
| #16 | [mh ”alpha-Linolenic Acid”] | 255 |
| #17 | ("alpha-linolenic"):ti,ab,kw | 556 |
| #18 | (ALA):ti,ab,kw | 1628 |
| #19 | [mh “Fish Oils”] | 4182 |
| #20 | ("fish oils"):ti,ab,kw | 1421 |
| #21 | [mh “Enteral Nutrition”] | 2179 |
| #22 | (“Enteral Nutrition”):ti,ab,kw | 4963 |
| #23 | #9 OR #10 OR #11 OR #12 OR #13 OR #14 OR #15 OR #16 OR #17 OR #18 OR #19 OR #20 # OR #21 OR #22 | 16698 |
| #24 | #8 AND #23 | 1504 |

Search database

- Igaku Chuo Zasshi

Search Formula and Hit Count

Igaku Chuo Zasshi search strategy （Date of Search: April 22, 2023）

| #1 | (ICU/TH or “intensive care unit”/TA or ICU/TA) and (PT=会議録除く) | 55436 |
| --- | --- | --- |
| #2 | (集中治療/TH or 集中治療/TA) and (PT=会議録除く) | 15549 |
| #3 | (クリティカルケア/TH or クリティカルケア/TA or クリティカル・ケア/TA) and (PT=会議録除く) | 7487 |
| #4 | 重症患者/TA and (PT=会議録除く) | 3899 |
| #5 | #1 or #2 or #3 or #4 | 68416 |
| #6 | ランダム化比較試験/TH or 準ランダム化比較試験/TH or ランダム化/AL or 無作為化/AL or 比較試験/AL or 臨床試験/AL or プラセボ/AL or 対照/AL or コントロール/AL or 臨床研究/AL | 349708 |
| #7 | (経腸栄養/TH or 経腸栄養/TA or 経腸栄養/TA or 経管栄養/TA or 経鼻栄養/TA or 強制栄養/TA or 鼻腔栄養/TA) and (PT=会議録除く) | 16288 |
| #8 | (オメガ3脂肪酸/TH or オメガ3脂肪酸/TA or n3脂肪酸/TA or ω3/TA) and (PT=会議録除く) | 4742 |
| #9 | ("Eicosapentaenoic Acid"/TH or "Eicosapentaenoic Acid"/TA or エイコサペンタエン酸/TA or EPA/TA) and (PT=会議録除く) | 30807 |
| #10 | ("Docosahexaenoic Acids"/TH or "Docosahexaenoic Acids"/TA or ドコサヘキサエン酸/TA or DHA/TA) and (PT=会議録除く) | 2602 |
| #11 | ("Alpha-Linolenic Acid"/TH or "Alpha-Linolenic Acid"/TA or αリノレン酸/TA or α-リノレン酸/TA or ALA/TA) and (PT=会議録除く) | 26081 |
| #12 | (魚油/TH or 魚油/TA or "fish oils"/TA) and (PT=会議録除く) | 4926 |
| #13 | #7 OR #8 OR #9 OR #10 OR #11 OR #12 | 75377 |
| #14 | #5 AND #6 AND #13 | 257 |

Flow Diagram

Risk of Bias

Short term mortality

Length of ICU stay

Infectious complication

Duration of mechanical ventilation

QoL

All adverse event

Muscle mass

Forest Plot and Funnel Plot

Short term mortality

Length of ICU stay

Infectious complication

Duration of mechanical ventilation

QoL

All adverse event

Muscle mass

（Sensitivity analysis）

１．Intended to administer enteral nutrition-enriched omega-3 fatty acids

Short term mortality

ICU length of stay

Infectious complication

Duration of mechanical ventilation

QoL

All adverse event

Muscle mass

２．Bolus administration

Short term mortality

ICU length of stay

Infectious complication

Duration of mechanical ventilation

Ventilator free days

QoL

N/A

All adverse event

Muscle mass

**CQ2-2: Should glutamine-enhanced enteral nutrition be administered in critically ill patients?**

Database

- MEDLINE (PubMed/Ovid)

Search formula and number of hits

PubMed search strategy（Search date: April 13, 2023）

| #1 | "Intensive Care Units"[Mesh] OR "intensive care"[tiab] OR "Critical Illness"[Mesh] OR "critical care"[Mesh] OR "critically ill"[tiab] OR "critical care"[tiab] OR "Perioperative period"[Mesh] OR "Perioperative period"[tiab] OR "Pancreatitis"[Mesh] OR "Pancreatitis"[tiab] OR "Wounds and Injuries"[Mesh] OR "Wounds and Injuries"[tiab] OR "Burns"[Mesh] OR "Burns"[tiab] OR "Renal Insufficiency"[Mesh] OR "Renal Insufficiency"[tiab] | 1,671,677 |
| --- | --- | --- |
| #2 | "Enteral Nutrition"[mesh] OR "Enteral Nutrition"[tiab] OR "Glutamine"[mesh] OR "Glutamine"[tiab] | 73,769 |
| #3 | "randomized controlled trial"[pt] OR "controlled clinical trial"[pt] OR "randomized"[tiab] OR "placebo"[tiab] OR "clinical trials as topic"[mesh:noexp] OR "randomly"[tiab] OR "trial"[ti] | 1,529,272 |
| #4 | "animals"[mh] NOT "humans"[mh] | 5,109,178 |
| #5 | #3 NOT #4 | 1,408,126 |
| #6 | #1 AND #2 AND #5 | 1,707 |

Database

- CENTRAL

Search formula and number of hits

CENTRAL search strategy（Search date: April 14, 2023）

| #1 | MeSH descriptor: [Intensive Care Units] explode all trees | 5225 |
| --- | --- | --- |
| #2 | MeSH descriptor: [Critical Illness] explode all trees | 3218 |
| #3 | (“critical illness”):ti,ab,kw | 5670 |
| #4 | (“critically ill”):ti,ab,kw | 8229 |
| #5 | MeSH descriptor: [Critical Care] explode all trees | 2650 |
| #6 | ("critical care"):ti,ab,kw | 13220 |
| #7 | ("intensive care"):ti,ab,kw | 35612 |
| #8 | #1 OR #2 OR #3 OR #4 OR #5 OR #6 OR #7 | 47685 |
| #9 | MeSH descriptor: [Enteral Nutrition] explode all trees | 2179 |
| #10 | (Enteral Nutrition):ti,ab,kw | 5603 |
| #11 | MeSH descriptor: [Glutamine] explode all trees | 748 |
| #12 | (Glutamine):ti,ab,kw | 2247 |
| #13 | #9 OR #10 OR #11 OR #12 | 7561 |
| #14 | #8 AND #13 | 1689 |

Database

- Igaku Chuo Zasshi

Search formula and number of hits

Igaku Chuo Zasshi search strategy（Search date: April 21, 2023）

| #1 | (ICU/TH or “intensive care unit”/TA or ICU/TA) and (PT=会議録除く) | 55,436 |
| --- | --- | --- |
| #2 | (集中治療/TH or 集中治療/TA) and (PT=会議録除く) | 15,549 |
| #3 | (クリティカルケア/TH or クリティカルケア/TA or クリティカル・ケア/TA) and (PT=会議録除く) | 7,487 |
| #4 | 重症患者/TA | 5,959 |
| #5 | #1 or #2 or #3 or #4 | 70,476 |
| #6 | ランダム化比較試験/TH or 準ランダム化比較試験/TH or ランダム化/AL or 無作為化/AL or 比較試験/AL or 臨床試験/AL or プラセボ/AL or 対照/AL or コントロール/AL or 臨床研究/AL | 349,708 |
| #7 | ((経腸栄養/TH or 経腸栄養/TA or 経管栄養/TH or 経管栄養/TA)) and (PT=会議録除く) | 16,142 |
| #8 | ((Glutamine/TH or Glutamine/TA)) and (PT=会議録除く) | 1,434 |
| #9 | #7 and #8 | 241 |
| #10 | #5 and #6 and #9 | 13 |

Flow diagram

ROB table

**Mortality**

**Length of ICU stay**

**Duration of Mechanical ventilation**

**Infectious Complication**

**Diarrhea**

**Adverse Events**

Forest Plot and Funnel Plot

**Mortality**

**Length of ICU stay**

**Duration of Mechanical ventilation**

**Infectious Complication**

**Diarrhea** **Diarrhea**

**Adverse Events**

**Mortality(Subgroup analysis)**

**Length of ICU stay (Subgroup analysis)**

**Duration of Mechanical ventilation(Subgroup analysis)**

**Infectious Complication(Subgroup analysis)**

**Adverse Events (Subgroup analysis)**

**Infectious Complication(Subgroup analysis**

有害事象サブグループ

**CQ2-3：Should enteral nutrition with oligomeric or elemental formulas rather than food-based blenderized formulas or polymeric formulas be used in critically ill patients?**

Search Database

- MEDLINE (PubMed/Ovid)

Search strategy and records identified

PubMed search strategy（Search Date, April 26, 2023）

| #1 | "Intensive Care Units"[Mesh] OR "intensive care"[tiab] OR "Critical Illness"[Mesh] OR "critical care"[Mesh] OR "critically ill"[tiab] OR "critical care"[tiab] | 308,551 |
| --- | --- | --- |
| #2 | "Sepsis"[Mesh] OR "sepsis"[tiab] OR "septic"[tiab] OR "wounds and injuries"[Mesh] OR trauma[tiab] OR burn[tiab] OR "perioperative period"[mesh] OR "Pancreatitis"[MeSH Terms] OR pancreatitis[tiab] OR Respiratory Distress Syndrome[mesh] OR "Acute respiratory failure"[tiab] OR "Acute respiratory distress syndrome"[tiab] OR "Stroke"[Mesh] OR "stroke"[tiab] OR "Shock"[mesh] OR "Shock"[tiab] OR "systemic inflammatory response syndrome"[tiab] OR "multiple organ failure"[tiab] | 2,101,178 |
| #3 | #1 OR #2 | 2,320,919 |
| #4 | "Food, Formulated"[mesh] OR "amino acids, peptides, and proteins"[Mesh] OR "Amino Acids"[tiab] OR "protein*"[tiab] OR "Peptide"[tiab] OR "oligomeric"[tiab] OR "Semi Elemental"[tiab] OR "Elemental Formulas"[tiab] OR "elemental diet"[tiab] | 8,981,087 |
| #5 | "Enteral Nutrition"[Mesh] OR "enteral nutrition"[tiab] OR "enteral feeding"[tiab] OR "feeding tube*"[tiab] OR "force feeding*"[tiab] OR "tube feeding*"[tiab] OR "gastric tube*" | 35,333 |
| #6 | #4 AND #5 | 8,703 |
| #7 | randomized controlled trial[pt] OR controlled clinical trial[pt] OR randomized[tiab] OR placebo[tiab] OR drug therapy[sh] OR randomly[tiab] OR trial[tiab] OR groups[tiab] NOT (animals [mh] NOT humans [mh]) | 4,992,832 |
| #8 | #3 AND #6 AND #7 | 1,189 |

Search Database

- CENTRAL

Search strategy and records identified

CENTRAL search strategy（Search Date, April 26, 2023）

| #1 | MeSH descriptor: [Intensive Care Units] explode all trees | 5,247 |
| --- | --- | --- |
| #2 | ("intensive care"):ti,ab,kw | 29,434 |
| #3 | MeSH descriptor: [Critical Illness] explode all trees | 3,239 |
| #4 | MeSH descriptor: [Critical Care] explode all trees | 2,658 |
| #5 | (critical illness):ti,ab,kw | 5,736 |
| #6 | (critically ill):ti,ab,kw | 8,357 |
| #7 | (critical care):ti,ab,kw | 13,418 |
| #8 | #1 OR #2 OR #3 OR #4 OR #5 OR #6 OR #7 | 42,083 |
| #9 | MeSH descriptor: [Sepsis] explode all trees | 6,746 |
| #10 | (sepsis):ti,ab,kw | 13,229 |
| #11 | (septic):ti,ab,kw | 5,408 |
| #12 | MeSH descriptor: [Wounds and Injuries] explode all trees | 34,513 |
| #13 | (trauma):ti,ab,kw | 18,803 |
| #14 | (burn):ti,ab,kw | 4,753 |
| #15 | MeSH descriptor: [Perioperative Period] explode all trees | 12,241 |
| #16 | MeSH descriptor: [Pancreatitis] explode all trees | 1,894 |
| #17 | (pancreatitis):ti,ab,kw | 5,307 |
| #18 | MeSH descriptor: [Respiratory Distress Syndrome] explode all trees | 3,286 |
| #19 | (Acute respiratory failure):ti,ab,kw | 4,807 |
| #20 | (Acute respiratory distress syndrome):ti,ab,kw | 3,317 |
| #21 | MeSH descriptor: [Stroke] explode all trees | 14,802 |
| #22 | (stroke):ti,ab,kw | 67,513 |
| #23 | MeSH descriptor: [Shock] explode all trees | 3,309 |
| #24 | (shock):ti,ab,kw | 13,041 |
| #25 | (Systemic Inflammatory Response Syndrome):ti,ab,kw | 1,562 |
| #26 | (multiple organ failure):ti,ab,kw | 2,172 |
| #27 | #9 OR #10 OR #11 OR #12 OR #13 OR #14 OR #15 OR #16 OR #17 OR #18 OR #19 OR #20 OR #21 OR #22 OR #23 OR #24 OR #25 OR #26 | 167,191 |
| #28 | #8 OR #27 | 197,313 |
| #29 | MeSH descriptor: [Enteral Nutrition] explode all trees | 2,184 |
| #30 | (enteral nutrition):ti,ab,kw | 5,661 |
| #31 | (enteral feeding):ti,ab,kw | 3,811 |
| #32 | (feeding tube):ti,ab,kw | 2,415 |
| #33 | (force feeding):ti,ab,kw | 87 |
| #34 | (tube feeding):ti,ab,kw | 2,415 |
| #35 | (gastric tube):ti,ab,kw | 2,130 |
| #36 | #29 OR #30 OR #31 OR #32 OR #33 OR #34 OR #35 | 9,351 |
| #37 | MeSH descriptor: [Food, Formulated] explode all trees | 1,584 |
| #38 | MeSH descriptor: [Amino Acids] explode all trees | 25,250 |
| #39 | MeSH descriptor: [Peptides] explode all trees | 88,710 |
| #40 | MeSH descriptor: [Proteins] explode all trees | 149,184 |
| #41 | (amino acid):ti,ab,kw | 8,754 |
| #42 | (protein):ti,ab,kw | 96,465 |
| #43 | (peptide):ti,ab,kw | 23,634 |
| #44 | (oligomeric):ti,ab,kw | 270 |
| #45 | (semi elemental):ti,ab,kw | 61 |
| #46 | (elemental diet):ti,ab,kw | 500 |
| #47 | (elemental formulas):ti,ab,kw | 125 |
| #48 | #37 OR #38 OR #39 OR #40 OR #41 OR #42 OR #43 OR #44 OR #45 OR #46 OR #47 | 270,495 |
| #49 | #28 AND #36 AND #48 | 1,166 |

Search Database

- Igaku Chuo Zasshi

Search strategy and records identified

Igaku Chuo Zasshi（Search Date, April 26, 2023）

| #1 | (ICU/TH or "intensive care"/TA or ICU/TA or クリティカルケア/TH or クリティカルケア/TA or クリティカル・ケア/TA or 集中治療/TA or 危篤/TH or 重症患者/TA) and (PT=会議録除く) | 68,541 |
| --- | --- | --- |
| #2 | (ショック/TH or 敗血症/TA or セプシス/TA or sepsis/TA or 敗血症性ショック/TA or "septic shock"/TA or ショック/TA or 全身性炎症反応症候群/TA or "systemic inflammatory response syndrome"/TA or SIRS/TA or or 多臓器不全/TA or 多臓器障害/TA or 多臓器機能不全/TA or 多臓器機能障害/TA or "organ failure"/TA or "organ dysfunction"/TA or MOF/TA or MODS/TA or創傷と損傷/TH or 重症外傷/TA or 熱傷/TA or or 膵炎/TH or 重症急性膵炎/TA or呼吸窮迫症候群/TH or 呼吸窮迫症候群/TA or 脳卒中/TH or 脳梗塞/TA or 脳出血/TA 脳卒中/TA) and (PT=会議録除く) | 17,201 |
| #3 | #1 or #2 | 85,366 |
| #4 | （"Amino Acids, Peptides, and Proteins"/TH or アミノ酸/TA or ペプチド/TA or タンパク質/TA or 成分栄養/TA or 消化態栄養/TA）and (PT=会議録除く) | 754,031 |
| #5 | （経腸栄養/TH or 経腸栄養/TA or 経管栄養/TA or 経鼻栄養/TA or 強制栄養/TA OR 鼻腔栄養/TA）and (PT=会議録除く) | 16,288 |
| #6 | #4 AND #5 | 3,005 |
| #7 | ランダム化比較試験/TH or 準ランダム化比較試験/TH or ランダム化/AL or 無作為化/AL or 比較試験/AL or 臨床試験/AL or プラセボ/AL or 対照/AL or コントロール/AL or 臨床研究/AL | 349,708 |
| #8 | #3 AND #6 AND #7 | 56 |

Flow diagram

Risk of Bias

**Mortality**

**Length of ICU stay**

**Duration of mechanical ventilation**

**Infectious complication**

**Diarrhea**

Forest Plot and Funnel Plot

**Mortality**

**Length of ICU stay**

**Duration of mechanical ventilation**

**Infectious complication**

**Diarrhea**

**CQ2-4: Should arginine-enhanced enteral nutrition be administered in critically ill patients?**

Database

- MEDLINE (PubMed/Ovid)

Search formula and number of hits

PubMed search strategy（Search date: May 1, 2023）

| #1 | “Intensive Care Units”[mh] OR “intensive care”[tiab] OR “Critical Illness”[mh] OR “critical care”[mh] OR “critically ill”[tiab] OR “critical care”[tiab] | 308904 |
| --- | --- | --- |
| #2 | “respiratory distress syndrome”[mh] OR “respiratory distress syndrome”[tiab] | 57261 |
| #3 | “Wounds and Injuries”[mh] OR Trauma[tiab] OR Burn[tiab] OR Burns[tiab] OR injur*[tiab] | 1781484 |
| #4 | “Multiple organ failure”[mh] OR “Multiple organ failure”[tiab] OR MOF[tiab] OR MODS[tiab] OR “Multiple organ dysfunction syndrome”[tiab] | 34632 |
| #5 | sepsis[mh] OR “shock, septic”[mh] OR sepsis[tiab] OR Shock[mh] OR Shock[tiab] OR septic[tiab] | 431773 |
| #6 | “pancreatitis, acute necrotizing”[mh] OR (severe[tiab] AND pancreatitis[tiab]) | 14527 |
| #7 | “ventilators, mechanical”[mh] OR “respiration, artificial”[mh] OR ventilator*[tiab] | 137158 |
| #8 | #1 OR #2 OR #3 OR #4 OR #5 OR #6 OR #7 | 2510206 |
| #9 | Arginine [mh] OR Arginine[tiab] | 126953 |
| #10 | “Immunonutrition diet”[mh] OR immunonutri*[tiab] OR “immune function”[tiab] OR “immune enhancing”[tiab] OR “immuno modulatory” [tiab] | 28177 |
| #11 | "enteral nutrition"[mh] OR “enteral nutrition"[tiab] OR "enteral diet"[tiab] | 26116 |
| #12 | #9 OR #10 OR #11 | 179773 |
| #13 | (randomized controlled trial [pt] OR controlled clinical trial [pt] OR randomized [tiab] OR placebo [tiab] OR clinical trials as topic [mesh:noexp] OR randomly [tiab] OR trial [ti]) NOT (animals [mh] NOT humans [mh]) | 1411734 |
| #14 | #8 AND #12 AND #13 | 2098 |

Database

■CENTRAL

Search formula and number of hits

CENTRAL search strategy（Search date: May 2, 2023）

| #1 | MeSH descriptor: [Intensive Care Units] explode all trees | 5225 |
| --- | --- | --- |
| #2 | ("intensive care"):ti,ab,kw | 28987 |
| #3 | MeSH descriptor: [Critical Illness] explode all trees | 3218 |
| #4 | MeSH descriptor: [Critical Care] explode all trees | 2650 |
| #5 | (“Critically ill”):ti,ab,kw | 8214 |
| #6 | ("critical care"):ti,ab,kw | 4859 |
| #7 | #1 OR #2 OR #3 OR #4 OR #5 OR #6 | 35106 |
| #8 | MeSH descriptor: [respiratory distress syndrome] explode all trees | 3263 |
| #9 | (“respiratory distress syndrome”):ti,ab,kw | 6515 |
| #10 | #8 OR #9 | 6625 |
| #11 | MeSH descriptor: [Wounds and Injuries] explode all trees | 34224 |
| #12 | (Trauma):ti,ab,kw | 18467 |
| #13 | (Burn?):ti,ab,kw | 5957 |
| #14 | (injur*):ti,ab,kw | 74035 |
| #15 | #11 OR #12 OR #13 OR #14 | 100220 |
| #16 | MeSH descriptor: [Multiple organ failure] explode all trees | 640 |
| #17 | (“Multiple organ failure”):ti,ab,kw | 1622 |
| #18 | (MOF):ti,ab,kw | 195 |
| #19 | (MODS):ti,ab,kw | 316 |
| #20 | (“Multiple organ dysfunction syndrome”):ti,ab,kw | 267 |
| #21 | #16 OR #17 OR #18 OR #19 OR #20 | 1934 |
| #22 | MeSH descriptor: [Sepsis] explode all trees | 6710 |
| #23 | MeSH descriptor: [Shock, Septic] explode all trees | 1236 |
| #24 | (sepsis):ti,ab,kw | 13042 |
| #25 | MeSH descriptor: [Shock] explode all trees | 3294 |
| #26 | (Shock):ti,ab,kw | 12827 |
| #27 | (septic):ti,ab,kw | 5344 |
| #28 | #22 OR #23 OR #24 OR #25 OR #26 OR #27 | 27170 |
| #29 | MeSH descriptor: [pancreatitis, acute necrotizing] explode all trees | 169 |
| #30 | (Severe):ti,ab,kw | 128365 |
| #31 | (pancreatitis):ti,ab,kw | 5266 |
| #32 | #30 AND #31 | 1442 |
| #33 | #29 OR #32 | 1518 |
| #34 | MeSH descriptor: [Ventilators, mechanical] explode all trees | 364 |
| #35 | MeSH descriptor: [respiration, artificial] explode all trees | 8195 |
| #36 | (Ventilator*):ti,ab,kw | 13398 |
| #37 | #34 OR #35 OR #36 | 18906 |
| #38 | #7 OR #10 OR #15 OR #21 OR #28 OR #33 OR #37 | 163321 |
| #39 | MeSH descriptor: [Arginine] explode all trees | 1658 |
| #40 | (Arginine):ti,ab,kw | 4909 |
| #41 | #39 OR #40 | 4987 |
| #42 | MeSH descriptor: [Immunonutrition diet] explode all trees | 2 |
| #43 | (Immunonutri*):ti,ab,kw | 389 |
| #44 | (“immune function”):ti,ab,kw | 3440 |
| #45 | (“immune enhancing”):ti,ab,kw | 181 |
| #46 | (**“**immune modulatory”):ti,ab,kw | 245 |
| #47 | #42 OR #43 OR #44 OR #45 OR #46 | 4163 |
| #48 | MeSH descriptor: [Enteral Nutrition] explode all trees | 2179 |
| #49 | (”Enteral Nutrition”):ti,ab,kw | 4963 |
| #50 | (”Enteral diet”):ti,ab,kw | 175 |
| #51 | #48 OR #49 OR #50 | 5022 |
| #52 | #41 OR #47 OR #51 | 13545 |
| #53 | #38 AND #52  (trials) | 2874  2835 |

Database

- Igaku Chuo Zasshi

Search formula and number of hits

Igaku Chuo Zasshi search strategy（Search date: May 1, 2023）

| #1 | (ICU/TH or "intensive care unit"/TA or ICU/TA) and (PT=会議録除く) | 55,530 |
| --- | --- | --- |
| #2 | (集中治療/TA) and (PT=会議録除く) | 12,421 |
| #3 | (クリティカルケア/TH or クリティカルケア/TA or クリティカル・ケア/TA) and (PT=会議録除く) | 7,498 |
| #4 | (重症/TA) and (PT=会議録除く) | 97,676 |
| #5 | (呼吸窮迫症候群/TH or 呼吸窮迫症候群/TA or ARDS/TA) and (PT=会議録除く) | 10,578 |
| #6 | (ショック_外傷性/TH or 外傷/TA) and (PT=会議録除く) | 64,030 |
| #7 | (熱傷/TH or 熱傷/TA) and (PT=会議録除く) | 11,630 |
| #8 | (創傷と損傷/TH or 損傷/AL) and (PT=会議録除く) | 269685 |
| #9 | (多臓器不全/TH or 多臓器不全/TA　or MOF/TA or MODS/TA) and (PT=会議録除く) | 8,227 |
| #10 | (ショック_敗血症性/TH or 敗血症/TH or 敗血症/TA) and (PT=会議録除く) | 27,283 |
| #11 | (膵炎-急性壊死性/TH or 急性膵炎/TA) and (PT=会議録除く) | 7233 |
| #12 | (人工呼吸/TH or 人工呼吸器/TH or 人工呼吸/TA) and (PT=会議録除く) | 38591 |
| #13 | #1 or #2 or #3 or #4 or #5 or #6 or #7 or #8 or #9 or #10 or #11 or #12 | 500664 |
| #14 | (アルギニン/TA or Arginine/TH or Arginine/TA) and (PT=会議録除く) | 7,178 |
| #15 | (免疫栄養食/TH or 免疫栄養/TA) and (PT=会議録除く) | 142 |
| #16 | (経腸栄養/TH or 経腸栄養/TA or 経腸栄養剤/TH or 経腸栄養剤/TA or 濃厚流動/TA or 注入栄養/TA or 経管栄養/TA or 経鼻胃管栄養/TA or 経鼻十二指腸栄養/TA) and (PT=会議録除く) | 17,045 |
| #17 | #14 or #15 or #16 | 24,036 |
| #18 | ランダム化比較試験/TH or 準ランダム化比較試験/TH or ランダム化/AL or 無作為化/AL or 比較試験/AL or 臨床試験/AL or プラセボ/AL or 対照/AL or コントロール/AL or 臨床研究/AL | 350,072 |
| #19 | #13 and #17 and #18 | 411 |

Flow diagram

ROB table

**Mortality**

**Length of ICU stay**

**Infectious Complication**

**Duration of Mechanical ventilation**

**Rate of Muscle Mass Loss**

**Adverse Events**

Forest Plot and Funnel Plot

**Mortality**

**Length of ICU stay**

**Infectious Complication**

**Duration of Mechanical ventilation**

**Rate of Muscle Mass Loss**

**Adverse Events**

**Mortality(Subgroup analysis)**

**Length of ICU stay (Subgroup analysis)**

**Infectious Complication(Subgroup analysis)**

**Duration of Mechanical ventilation(Subgroup analysis)**

**CQ2-5：Should high-fat, low-carbohydrate enteral nutrition be used for critically ill patients?**

Database

- MEDLINE (PubMed/Ovid)

Search formula and number of hits

PubMed search strategy (Search date: April 11, 2023)

| #1 | "Intensive Care Units" [Mesh] OR “intensive care"[tiab] OR "Critical Illness"[Mesh] OR "critical care"[Mesh] OR “critically ill”[tiab] OR "critical care"[tiab] OR “Respiration, Artificial”[Mesh] OR “Ventilators, Mechanical”[Mesh] | 377,774 |
| --- | --- | --- |
| #2 | “Diet, High-Fat”[Mesh] OR “high fat”[tiab] OR “fat based”[tiab] OR "fat modified"[tiab] | 57,124 |
| #3 | "Diet, Carbohydrate Restricted"[MeSH] OR "low carbohydrate"[ tiab] OR "low cho"[tiab] OR "diabetes specific"[tiab] OR “diabetic formula"[tiab] OR "glucose based"[tiab] OR "carbohydrate based"[tiab] | 10,712 |
| #4 | randomized controlled trial[pt] OR controlled clinical trial[pt] OR randomized[tiab] OR placebo[tiab] OR drug therapy[sh] OR randomly[tiab] OR trial[tiab] OR groups[tiab] NOT (animals [mh]  NOT humans [mh]) | 4,982,413 |
| #5 | #1 AND (#2 OR #3) AND #4 | 60 |

Database

- CENTRAL

Search formula and number of hits

CENTRAL search strategy（Search date: April 14, 2023）

| #1 | MeSH descriptor: [Intensive Care Units] explode all trees | 5225 |
| --- | --- | --- |
| #2 | ("intensive care unit*"):ti,ab,kw | 19846 |
| #3 | MeSH descriptor: [Critical Illness] explode all trees | 3218 |
| #4 | (“critical illness”):ti,ab,kw | 4441 |
| #5 | (“critically ill”):ti,ab,kw | 8214 |
| #6 | MeSH descriptor: [Critical Care] explode all trees | 2650 |
| #7 | ("critical care"):ti,ab,kw | 4859 |
| #8 | ("intensive care"):ti,ab,kw | 28987 |
| #9 | MeSH descriptor: [Respiration, Artificial] explode all trees | 8194 |
| #10 | MeSH descriptor: [Ventilators, Mechanical] explode all trees | 364 |
| #11 | #1 OR #2 OR #3 OR #4 OR #5 OR #6 OR #7 OR #8 OR #9 OR #10 | 40907 |
| #12 | MeSH descriptor: [Diet, High-Fat] explode all trees | 351 |
| #13 | ("high fat"):ti,ab,kw | 4334 |
| #14 | ("fat based"):ti,ab,kw | 63 |
| #15 | ("fat modified"):ti,ab,kw | 86 |
| #16 | #12 OR #13 OR #14 OR #15 | 4471 |
| #17 | MeSH descriptor: [Diet, Carbohydrate-Restricted] explode all trees | 661 |
| #18 | ("low carbohydrate"):ti,ab,kw | 1508 |
| #19 | ("low cho"):ti,ab,kw | 97 |
| #20 | ("diabetes specific"):ti,ab,kw | 463 |
| #21 | ("diabetic formula"):ti,ab,kw | 5 |
| #22 | ("glucose based"):ti,ab,kw | 195 |
| #23 | ("carbohydrate based"):ti,ab,kw | 83 |
| #24 | #17 OR #18 OR #19 OR #20 OR #21 OR #22 OR #23 | 2594 |
| #25 | #11 AND (#16 OR #24) | 64 |

Database

- Igaku Chuo Zasshi

Search formula and number of hits

Igaku Chuo Zasshi search strategy（Search date: April 23: 2023）

| #1 | (ICU/TH or “intensive care unit”/TA or ICU/TA) and (PT=会議録除く) | 55,436 |
| --- | --- | --- |
| #2 | (集中治療/TA) and (PT=会議録除く) | 12,382 |
| #3 | (クリティカルケア/TH or クリティカルケア/TA or クリティカル・ケア/TA) and (PT=会議録除く) | 7,487 |
| #4 | (重症患者/TA) and (PT=会議録除く) | 3,899 |
| #5 | (人工呼吸/TH) and (PT=会議録除く) | 26,414 |
| #6 | (人工呼吸器/TH) and (PT=会議録除く) | 7,082 |
| #7 | (陽圧呼吸/TH) and (PT=会議録除く) | 10,507 |
| #8 | #1 or #2 or #3 or #4 or #5 or #6 or #7 | 94,073 |
| #9 | ([高脂肪食]/TH) and (PT=会議録除く) | 1,155 |
| #10 | (高脂肪/TA) and (PT=会議録除く) | 2,352 |
| 11 | #9 or #10 | 2,778 |
| 12 | ([低炭水化物食]/TH) and (PT=会議録除く) | 1,001 |
| 13 | ([低炭水化物]/TA) and (PT=会議録除く) | 175 |
| 14 | (糖尿病食/TA) and (PT=会議録除く) | 877 |
| 15 | #11 or #12 or #13 or #14 | 4,604 |
| 16 | ランダム化比較試験/TH or 準ランダム化比較試験/TH or ランダム化/AL or 無作為化/AL or 比較試験/AL or 臨床試験/AL or プラセボ/AL or 対照/AL or コントロール/AL or 臨床研究/AL | 349,708 |
| 17 | #16 and #8 and (#11 or #15) | 14 |

Flow diagram

Risk of Bias

30-day mortality

Length of ICU stay

Duration of mechanical ventilation

Diarrhea

Gastric residual volume

Forest Plot and Funnel Plot

30-day mortality

Length of ICU stay

Duration of mechanical ventilation

Diarrhea

Gastric residual volume

**CQ2-6 ：Should intravenous lipid emulsions be administered to critically ill patients receiving parenteral nutrition?**

Search database

- MEDLINE (PubMed/Ovid)

Search Formula and Hit Count

PubMed search strategy（Date of Search: April 8, 2023）

| #1 | "Intensive Care Units"[Mesh] OR “intensive care"[tiab] OR "Critical Illness"[Mesh] OR "critical care"[Mesh] OR “critically ill”[tiab] OR "critical care"[tiab] | 307,654 |
| --- | --- | --- |
| #2 | "Parenteral Nutrition"[MeSH Terms] | 25,311 |
| #3 | "parenteral nutrition"[Title/Abstract] | 22,460 |
| #4 | "Fat Emulsions, Intravenous"[MeSH Terms] | 4,255 |
| #5 | "fat emulsion"[Title/Abstract] | 1,408 |
| #6 | "lipid emulsion"[Title/Abstract] | 2,572 |
| #7 | #2 OR #3 OR #4 OR #5 OR #6 | 38,073 |
| #8 | randomized controlled trial [pt] OR controlled clinical trial [pt] OR randomized [tiab] OR placebo [tiab] OR clinical trials as topic [mesh: noexp] OR randomly [tiab] OR trial [ti]NOT (animals [mh] NOT humans [mh]) | 1,488,611 |
| #9 | #1 AND #7 AND #8 | 822 |
|  |  |  |
|  |  |  |

Search database

- CENTRAL

Search Formula and Hit Count

CENTRAL search strategy（Date of Search: April 18, 2023）

| #1 | MeSH descriptor: [Intensive Care Units] explode all trees | 5,225 |
| --- | --- | --- |
| #2 | MeSH descriptor: [Critical Illness] explode all trees | 3,218 |
| #3 | (“critical illness”):ti,ab,kw | 4,441 |
| #4 | (“critically ill”):ti,ab,kw | 8,214 |
| #5 | MeSH descriptor: [Critical Care] explode all trees | 2,650 |
| #6 | ("critical care"):ti,ab,kw | 4,859 |
| #7 | ("intensive care"):ti,ab,kw | 28,987 |
| #8 | #1 OR #2 OR #3 OR #4 OR #5 OR #6 OR #7 | 32,413 |
| #9 | MeSH descriptor: [Parenteral Nutrition] explode all trees | 1,949 |
| #10 | ("parenteral nutrition"):ti,ab,kw | 4,450 |
| #11 | MeSH descriptor: [Fat Emulsions, Intravenous] explode all trees | 502 |
| #12 | ("fat emulsion"):ti,ab,kw | 321 |
| #13 | ("lipid emulsion"):ti,ab,kw | 630 |
| #14 | #9 OR #10 OR #11 OR #12 OR #13 | 5,046 |
| #15 | #8 AND #14 | 824 |

Search database

- Igaku Chuo Zasshi

Search Formula and Hit Count

Igaku Chuo Zasshi search strategy（Date of Search: April 20, 2023）

| #1 | (ICU/TH or “intensive care unit”/TA or ICU/TA) and (PT=会議録除く) | 55,436 |
| --- | --- | --- |
| #2 | (集中治療/TA) and (PT=会議録除く) | 12,382 |
| #3 | (クリティカルケア/TH or クリティカルケア/TA or クリティカル・ケア/TA) and (PT=会議録除く) | 7,487 |
| #4 | 重症患者/TA | 5,959 |
| #5 | #1 or #2 or #3 or #4 | 70,476 |
| #6 | (静注用脂肪乳剤/TH or 静注用脂肪乳剤/TA) and (PT=会議録除く) | 1,845 |
| #7 | (静脈栄養/TH or 静脈栄養/TA) and (PT=会議録除く) | 11,551 |
| #8 | #6 or #7 | 12,946 |
| #9 | #5 and #8 | 615 |
| #10 | (ランダム化比較試験/TH or 準ランダム化比較試験/TH or ランダム化/AL or 無作為化/AL or 比較試験/AL or 臨床試験/AL or プラセボ/AL or 対照/AL or コントロール/AL or 臨床研究/AL) | 349,708 |
| 11 | #9 and #10 | 56 |

Flow Diagram

Risk of Bias

**Hospital mortality**

**Length of ICU stay**

**Duration of mechanical ventilation**

**Infectious complication**

**All adverse event**

Forest Plot

**Hospital mortality**

**Hospital mortality（Sensitivity analysis：omega-3 vs non-omega-3）**

**Length of ICU stay**

**Length of ICU stay（Hospital mortality（Sensitivity analysis：omega-3 vs non-omega-3）**

**Duration of mechanical ventilation**

**Infectious complication**

**All adverse event**

**CQ2-7-1: Should prebiotics be administered in critically ill patients?**

Search database

- MEDLINE (PubMed/Ovid)

Search Formula and Hit Count

PubMed search strategy (Search Date: 30 April 2023)

| #1 | "Intensive Care Units"[Mesh] OR “intensive care"[tiab] OR "Critical Illness"[Mesh] OR "critical care"[Mesh] OR “critically ill”[tiab] OR "critical care"[tiab] OR Intensive care unit[Title/Abstract] OR ICU[Title/Abstract] | 324094 |
| --- | --- | --- |
| #2 | prebiotics[Mesh] | 4,773 |
| #3 | prebiotics[tiab] | 6,072 |
| #4 | “dietary fiber”[Mesh] | 23,477 |
| #5 | fiber[tiab] | 187,316 |
| #6 | roughage[tiab] | 1,460 |
| #7 | “resistant starch“[Mesh] | 244 |
| #8 | “resistant starch”[tiab] | 2491 |
| #9 | “indigestible dextrin”[tiab] | 26 |
| #10 | polysaccharide[tiab] | 54081 |
| #11 | polydextrose[tiab] | 299 |
| #12 | isomaltodextrin[tiab] | 16 |
| #13 | inulin[Mesh] | 7,390 |
| #14 | inulin[tiab] | 9566 |
| #15 | pectin[Mesh] | 6871 |
| #16 | pectin[tiab] | 10687 |
| #17 | “gum arabic”[Mesh] | 954 |
| #18 | “guar gum”[tiab] | 2002 |
| #19 | “acacia gum”[tiab] | 158 |
| #20 | glucan[Mesh] | 191055 |
| #21 | cellulose[Mesh] | 57,332 |
| #22 | lignin[Mesh] | 13,402 |
| #23 | psyllium[Mesh] | 731 |
| #24 | psyllium[tiab] | 888 |
| #25 | hemicellulose[tiab] | 6202 |
| #26 | glucomannan[tiab] | 1,248 |
| #27 | oat[tiab] | 10,098 |
| #28 | “wheat brans”[tiab] | 29 |
| #29 | oligosaccharides[Mesh] | 95,669 |
| #30 | oligosaccharides[tiab] | 26354 |
| #31 | fructooligosaccharides[tiab] | 1,698 |
| #32 | galactooligosaccharides[tiab] | 1,090 |
| #33 | xylooligosaccharide[tiab] | 325 |
| #34 | isomaltooligosaccharides[tiab] | 190 |
| #35 | lactulose[Mesh] | 2,355 |
| #36 | fructans[Mesh] | 8,450 |
| #37 | oligofructose[tiab] | 589 |
| #38 | lactosucrose[tiab] | 67 |
| #39 | raffinose[tiab] | 2854 |
| #40 | #2 OR #3 OR #4 OR #5 OR #6 OR #7 OR #8 OR #9 OR #10 OR #11 OR #12 OR #13 OR #14 OR #15 OR #16 OR #17 OR #18 OR #19 OR #20 OR #21 OR #22 OR #23 OR #24 OR #25 OR #26 OR #27 OR #28 OR #29 OR #30 OR #31 OR #32 OR #33 OR #34 OR #35 OR #36 OR #37 OR #38 OR #39 | 553526 |
| #41 | randomized controlled trial[pt] OR controlled clinical trial[pt] OR randomized[tiab] OR placebo[tiab] OR drug therapy[sh] OR randomly[tiab] OR trial[tiab] OR groups[tiab] NOT (animals [mh]  NOT humans [mh]) | 4,985,575 |
| #42 | #1 AND #40 AND #41 | 797 |

Search database

- CENTRAL

Search Formula and Hit Count

CENTRAL search strategy (Search Data: 15 April 2023)

| #1 | MeSH descriptor: [Intensive Care Units] explode all trees | 5225 |
| --- | --- | --- |
| #2 | MeSH descriptor: [Critical Illness] explode all trees | 3218 |
| #3 | (“critical illness”):ti,ab,kw | 4441 |
| #4 | (“critically ill”):ti,ab,kw | 8214 |
| #5 | MeSH descriptor: [Critical Care] explode all trees | 2650 |
| #6 | ("critical care"):ti,ab,kw | 4859 |
| #7 | ("intensive care"):ti,ab,kw | 28987 |
| #8 | #1 OR #2 OR #3 OR #4 OR #5 OR #6 OR #7 | 35462 |
| ＃9 | MeSH descriptor: [Prebiotics] explode all trees | 601 |
| ＃10 | (prebiotics):ti,ab,kw | 1238 |
| ＃11 | MeSH descriptor: [Dietary Fiber] explode all trees | 2514 |
| ＃12 | (fiber):ti,ab,kw | 12592 |
| ＃13 | (roughage):ti,ab,kw | 35 |
| ＃14 | MeSH descriptor: [Resistant Starch] explode all trees | 27 |
| ＃15 | (resistant starch):ti,ab,kw | 495 |
| #16 | (indigestible dextrin):ti,ab,kw | 37 |
| ＃17 | (polysaccharide):ti,ab,kw | 2044 |
| #18 | (polydextrose):ti,ab,kw | 137 |
| #19 | (isomaltodextrin):ti,ab,kw | 4 |
| ＃20 | MeSH descriptor: [Inulin] explode all trees | 284 |
| ＃21 | (inulin):ti,ab,kw | 1203 |
| ＃22 | MeSH descriptor: [Pectins] explode all trees | 135 |
| ＃23 | (pectin):ti,ab,kw | 347 |
| ＃24 | MeSH descriptor: [Gum Arabic] explode all trees | 35 |
| ＃25 | ("guar gum"):ti,ab,kw | 283 |
| #26 | (acacia gum):ti,ab,kw | 55 |
| #27 | MeSH descriptor: [Glucans] explode all trees | 4424 |
| #28 | MeSH descriptor: [Cellulose] explode all trees | 985 |
| #29 | MeSH descriptor: [Lignin] explode all trees | 12 |
| #30 | MeSH descriptor: [Psyllium] explode all trees | 205 |
| ＃31 | (psyllium):ti,ab,kw | 432 |
| #32 | (hemicellulose):ti,ab,kw | 33 |
| #33 | (glucomannan):ti,ab,kw | 112 |
| ＃34 | (oat):ti,ab,kw | 1038 |
| ＃35 | (wheat brans):ti,ab,kw | 6 |
| #36 | MeSH descriptor: [Oligosaccharides] explode all trees | 3721 |
| #37 | (oligosaccharides):ti,ab,kw | 1131 |
| #38 | (fructooligosaccharides):ti,ab,kw | 403 |
| #39 | (galactooligosaccharides):ti,ab,kw | 270 |
| #40 | (xylooligosaccharide):ti,ab,kw | 27 |
| ＃41 | (isomaltooligosaccharides):ti,ab,kw | 13 |
| #42 | MeSH descriptor: [Lactulose] explode all trees | 493 |
| #43 | MeSH descriptor: [Fructans] explode all trees | 306 |
| #44 | (oligofructose):ti,ab,kw | 193 |
| #45 | (lactosucrose):ti,ab,kw | 5 |
| #46 | (raffinose):ti,ab,kw | 99 |
| #47 | #9 OR #10 OR #11 OR #12 OR #13 OR #14 OR #15 OR #16 OR #17 OR #18 OR #19 OR #20 OR #21 OR #22 OR #23 OR #24 OR #25 OR #26 OR #27 OR #28 OR #29 OR #30 OR #31 OR #32 OR #33 OR #34 OR #35 OR #36 OR #37 OR #38 OR #39 OR #40 OR #41 OR #42 OR #43 OR #44 OR #45 OR #46 | 25319 |
|  | #8 AND #47 | 543 |

Search database

- Igaku Chuo Zasshi

Search Formula and Hit Count

Igaku Chuo Zasshi search strategy (Search Data: 22 April 2023)

| #1 | (ICU/TH or “intensive care unit”/TA or ICU/TA) and (PT=会議録除く) | 55436 |
| --- | --- | --- |
| #2 | (集中治療/TH or 集中治療/TA) and (PT=会議録除く) | 15549 |
| #3 | (クリティカルケア/TH or クリティカルケア/TA or クリティカル・ケア/TA) and (PT=会議録除く) | 7487 |
| #4 | 重症患者/TA | 5959 |
| #5 | #1 or #2 or #3 or #4 | 70476 |
| #6 | (((食物繊維/TH or 食物繊維/TA)) and (PT=会議録除く)) | 4459 |
| #7 | (((プレバイオティクス/TH or プレバイオティクス/TA)) and (PT=会議録除く)) | 1126 |
| #8 | ((((@Dextrins/TH and @難消化性デンプン/TH) or 難消化性デキストリン/TA))) and (PT=会議録除く) | 230 |
| #9 | (((Polydextrose/TH or ポリデキストロース/TA)) and (PT=会議録除く)) | 49 |
| #10 | (((non/TA and (デンプン/TH or starch/TA) and (Polysaccharides/TH or polysaccharides/TA) or 非デンプン多糖類/TA or 非でんぷん多糖類/TA) and (PT=会議録除く))) | 20 |
| #11 | (((Oligosaccharides/TH or オリゴ糖/TA)) and (PT=会議録除く)) | 7035 |
| #12 | (((Fructooligosaccharide/TH or フラクトオリゴ糖/TA)) and (PT=会議録除く)) | 217 |
| #13 | (((Galactooligosaccharide/TH or ガラクトオリゴ糖/TA)) and (PT=会議録除く)) | 87 |
| #14 | (((Xylooligosaccharide/TH or キシロオリゴ糖/TA)) and (PT=会議録除く)) | 39 |
| #15 | (((Lactosucrose/TH or ラクトスクロース/TA)) and (PT=会議録除く)) | 73 |
| #16 | (((Lactulose/TH or ラクツロース/TA)) and (PT=会議録除く)) | 578 |
| #17 | (((Inulin/TH or イヌリン/TA)) and (PT=会議録除く)) | 513 |
| #18 | (((Fructans/TH or フルクタン/TA)) and (PT=会議録除く)) | 403 |
| #19 | (((アラビアゴム/TH or アラビアガム/TA)) and (PT=会議録除く)) | 25 |
| #20 | ((("Guar Gum"/TH or グアーガム/TA)) and (PT=会議録除く)) | 160 |
| #21 | (((部分加水分解グアーガム/TA or PHGG/TA) and (PT=会議録除く))) | 47 |
| #22 | ((エンドウ/TH or pea/TA) and fiber/TA) and (PT=会議録除く) | 18 |
| #23 | (((Glucans/TH or グルカン/TA)) and (PT=会議録除く)) | 10901 |
| #24 | (((Psyllium/TH or サイリウム/TA)) and (PT=会議録除く)) | 104 |
| #25 | (((Hemicellulose/TH or ヘミセルロース/TA)) and (PT=会議録除く)) | 62 |
| #26 | (((Glucomannan/TH or グルコマンナン/TA)) and (PT=会議録除く)) | 77 |
| #27 | (((Pectins/TH or ペクチン/TA)) and (PT=会議録除く)) | 388 |
| #28 | (((Cellulose/TH or セルロース/TA)) and (PT=会議録除く)) | 3405 |
| #29 | (((Lignin/TH or リグニン/TA)) and (PT=会議録除く)) | 95 |
| #30 | (((soy/TA and (Polysaccharides/TH or polysaccharides/TA) or 大豆多糖類/TA) and (PT=会議録除く))) | 51 |
| #31 | (((難消化性デンプン/TH or レジスタントスターチ/TA)) and (PT=会議録除く)) | 265 |
| #32 | (((カラスムギ属/TH or oat/TA)) and (PT=会議録除く)) | 6045 |
| #33 | ((((Isomaltodextrin/TH or イソマルトデキストリン/TA)) and (PT=会議録除く))) | 17 |
| #34 | (((Raffinose/TH or ラフィノース/TA)) and (PT=会議録除く)) | 122 |
| #35 | #6 or #7 or #8 or #9 or #10 or #11 or #12 or #13 or #14 or #15 or #16 or #17 or #18 or #19 or #20 or #21 or #22 or #23 or #24 or #25 or #26 or #27 or #28 or #29 or #30 or #31 or #32 or #33 or #34 | 29589 |
| #36 | ランダム化比較試験/TH or 準ランダム化比較試験/TH or ランダム化/AL or 無作為化/AL or 比較試験/AL or 臨床試験/AL or プラセボ/AL or 対照/AL or コントロール/AL or 臨床研究/AL | 349708 |
| #37 | #5 and #35 and #36 | 72 |

**Flow diagram**

Risk of Bias

**In-hospital mortality**

**Length of ICU stay**

**Duration of mechanical ventilation**

**Infectious complication**

**All adverse events**

Forest Plot and Funnel Plot

**In-hospital mortality**

**Length of ICU stay**

**Duration of mechanical ventilation**

**Infectious complication**

**All adverse events**

**CQ2-7-2: Should probiotics be administered in critically ill patients?**

Search database

- MEDLINE (PubMed/Ovid)

Search expression

PubMed search strategy (Search Date: 30 April 2023)

| #1 | "Intensive Care Units"[Mesh] OR “intensive care"[tiab] OR "Critical Illness"[Mesh] OR "critical care"[Mesh] OR “critically ill”[tiab] OR "critical care"[tiab] | 307,6024 |
| --- | --- | --- |
| #2 | probiotics[Mesh] | 24,030 |
| #3 | bacillus subtilis[tiab] | 33,745 |
| #4 | bifidobacterium[tiab] | 11,563 |
| #5 | enterococcus[tiab] | 27,647 |
| #6 | fermented product[tiab] | 349 |
| #7 | gluconacetobacter[tiab] | 568 |
| #8 | leuconostocaceae[tiab] | 86 |
| #9 | lactobacillaceae[tiab] | 807 |
| #10 | lactobacillus[tiab] | 41,064 |
| #11 | lactococcus[tiab] | 7,838 |
| #12 | pediococcus[tiab] | 2,427 |
| #13 | probiotic agent[tiab] | 108 |
| #14 | probiotics[tiab] | 24,929 |
| #15 | #2 OR #3 OR #4 OR #5 OR #6 OR #7 OR #8 OR #9 OR #10 OR #11 OR #12 OR #13 OR #14 | 132,779 |
| #16 | randomized controlled trial[pt] OR controlled clinical trial[pt] OR randomized[tiab] OR placebo[tiab] OR drug therapy[sh] OR randomly[tiab] OR trial[tiab] OR groups[tiab] NOT (animals [mh]  NOT humans [mh]) | 4,980,236 |
| #17 | #1 AND #15 AND #16 | 644 |

Search database

- CENTRAL

Search Formula and Hit Count

CENTRAL search strategy (Search Data: 15 April 2023)

| #1 | MeSH descriptor: [Intensive Care Units] explode all trees | 5225 |
| --- | --- | --- |
| #2 | MeSH descriptor: [Critical Illness] explode all trees | 3218 |
| #3 | (“critical illness”):ti,ab,kw | 4441 |
| #4 | (“critically ill”):ti,ab,kw | 8214 |
| #5 | MeSH descriptor: [Critical Care] explode all trees | 2650 |
| #6 | ("critical care"):ti,ab,kw | 4859 |
| #7 | ("intensive care"):ti,ab,kw | 28987 |
| #8 | #1 OR #2 OR #3 OR #4 OR #5 OR #6 OR #7 | 35462 |
| #9 | MeSH descriptor: [Probiotics] explode all trees | 3004 |
| #10 | ("bacillus subtilis"):ti,ab,kw | 198 |
| #11 | (bifidobacterium):ti,ab,kw | 3384 |
| #12 | (enterococcus):ti,ab,kw | 1254 |
| #13 | ("fermented product"):ti,ab,kw | 111 |
| #14 | (gluconacetobacter):ti,ab,kw | 8 |
| #15 | (leuconostocaceae):ti,ab,kw | 3 |
| #16 | (lactobacillaceae):ti,ab,kw | 47 |
| #17 | (lactobacillus):ti,ab,kw | 6031 |
| #18 | (lactococcus):ti,ab,kw | 168 |
| #19 | (pediococcus):ti,ab,kw | 82 |
| #20 | ("probiotic agent"):ti,ab,kw | 1042 |
| #21 | (probiotics):ti,ab,kw | 6893 |
| #22 | #9 OR #10 OR #11 OR #12 OR #13 OR #14 OR #15 OR #16 OR #17 OR #18 OR #19 OR #20 OR #21 | 11919 |
| #23 | #8 AND #22 | 348 |

343 out of 348 cases Tria

Search databas

Search database

- Igaku Chuo Zasshi

rch Formula and Hit Count

Igaku Chuo Zasshi search strategy (Search Data: 22 April 2023)

| #1 | (ICU/TH or “intensive care unit”/TA or ICU/TA) and (PT=会議録18除く) | 55,436 |
| --- | --- | --- |
| #2 | (クリティカルケア/TH or 集中治療/TA) and (PT=会議録除く) | 15,549 |
| #3 | (クリティカルケア/TH or クリティカルケア/TA or クリティカル・ケア/TA) and (PT=会議録除く) | 7,487 |
| #4 | 重症患者/TA | 5,959 |
| #5 | #1 or #2 or #3 or #4 | 70,476 |
| #6 | "Bacillus subtilis"/TH | 1,159 |
| #7 | ((Bifidobacterium/TH or bifidobacterium/TA)) | 3,367 |
| #8 | "Enterococcus faecium"/TH | 511 |
| #9 | "Enterococcus faecalis"/TH | 2,060 |
| #10 | Enterococcus/TH | 4,536 |
| #11 | "Enterococcus hirae"/TH | 43 |
| #12 | 発酵食品/TH | 4,547 |
| #13 | Gluconacetobacter/TH | 20 |
| #14 | "Gluconacetobacter xylinus"/TH | 5 |
| #15 | Leuconostocaceae/TH | 118 |
| #16 | Lactobacillaceae/TH | 3,983 |
| #17 | Lactobacillus/TH | 3,910 |
| #18 | "Lactobacillus acidophilus"/TH | 251 |
| #19 | "Lactobacillus casei"/TH | 618 |
| #20 | "Lactobacillus brevis"/TH | 133 |
| #21 | "Lactobacillus delbrueckii"/TH | 123 |
| #22 | "Lactobacillus fermentum"/TH | 50 |
| #23 | "Lactobacillus helveticus"/TH | 87 |
| #24 | "Lactobacillus leichmannii"/TH | 3 |
| #25 | "Lactobacillus plantarum"/TH | 421 |
| #26 | "Lactobacillus reuteri"/TH | 100 |
| #27 | "Lactobacillus rhamnosus"/TH | 203 |
| #28 | "Lactobacillus paracasei"/TH | 124 |
| #29 | "Lactobacillus salivarius"/TH | 63 |
| #30 | "Lactobacillus gasseri"/TH | 379 |
| #31 | "Lactobacillus johnsonii"/TH | 40 |
| #32 | "Bifidobacterium bifidum"/TH | 122 |
| #33 | "Lactobacillus crispatus"/TH | 34 |
| #34 | "Lactobacillus sakei"/TH | 14 |
| #35 | "Lactobacillus pentosus"/TH | 24 |
| #36 | "Bacillus coagulans"/TH | 46 |
| #37 | Bifidobacterium/TH | 2,981 |
| #38 | "Lactococcus lactis"/TH | 248 |
| #39 | Lactococcus/TH | 339 |
| #40 | "Pediococcus pentosaceus"/TH | 7 |
| #41 | "Pediococcus acidilactici"/TH | 31 |
| #42 | Pediococcus/TH | 69 |
| #43 | プロバイオティクス/TH | 5,478 |
| #44 | #6 or #7 or #8 or #9 or #10 or #11 or #12 or #13 or #14 or #15 or #16 or #17 or #18 or #19 or #20 or #21 or #22 or #23 or #24 or #25 or #26 or #27 or #28 or #29 or #30 or #31 or #32 or #33 or #34 or #35 or #36 or #37 or #38 or #39 or #40 or #41 or #42 or #43 | 20,046 |
| #45 | ランダム化比較試験/TH or 準ランダム化比較試験/TH or ランダム化/AL or 無作為化/AL or 比較試験/AL or 臨床試験/AL orプラセボ/AL or 対照/AL or コントロール/AL or 臨床研究/AL | 349,708 |
| #46 | #5 AND #44 AND #45 | 71 |

**Flow diagram**

Risk of Bias

**In-hospital mortality**

**Length of ICU stay**

**Duration of mechanical ventilation**

**Infectious complication**

**All adverse events**

**Physical function**

Forest Plot and Funnel Plot

**In-hospital mortality**

**Length of ICU stay**

**Duration of mechanical ventilation**

**Infectious complication**

**All adverse events**

**Physical function**

**CQ2-7-3: Should synbiotics be administered in critically ill patients?**

Search database

- MEDLINE (PubMed/Ovid)

Search Formula and Hit Count

PubMed search strategy (Search Date: 30 April 2023)

| #1 | "Intensive Care Units"[MeSH Terms] OR "intensive care"[Title/Abstract] OR "Critical Illness"[MeSH Terms] OR "critical care"[MeSH Terms] OR "critically ill"[Title/Abstract] OR "critical care"[Title/Abstract] OR "burns"[MeSH Terms] OR "sepsis"[MeSH Terms] OR "respiratory distress syndrome"[MeSH Terms] OR "shock"[MeSH Terms] OR "multiple organ failure"[MeSH Terms] OR "transplantation"[MeSH Terms] OR "transplantation"[Title/Abstract] | [1,238,872](https://pubmed.ncbi.nlm.nih.gov/?term=%22Intensive+Care+Units%22%5BMeSH+Terms%5D+OR+%22intensive+care%22%5BTitle%2FAbstract%5D+OR+%22Critical+Illness%22%5BMeSH+Terms%5D+OR+%22critical+care%22%5BMeSH+Terms%5D+OR+%22critically+ill%22%5BTitle%2FAbstract%5D+OR+%22critical+care%22%5BTitle%2FAbstract%5D+OR+%22burns%22%5BMeSH+Terms%5D+OR+%22sepsis%22%5BMeSH+Terms%5D+OR+%22respiratory+distress+syndrome%22%5BMeSH+Terms%5D+OR+%22shock%22%5BMeSH+Terms%5D+OR+%22multiple+organ+failure%22%5BMeSH+Terms%5D+OR+transplantation%5BMeSH+Terms%5D+OR+transplantation%5BTitle%2FAbstract%5D&sort=) |
| --- | --- | --- |
| #2 | "prebiotics"[MeSH Terms] | 4,757 |
| #3 | "prebiotics"[Title/Abstract] | 6,054 |
| #4 | "dietary Fiber"[MeSH Terms] | 23,454 |
| #5 | "oligosaccharides"[MeSH Terms] | 95,633 |
| #6 | "oligofructose"[Title/Abstract] | 588 |
| #7 | "xylooligosaccharide"[Title/Abstract] | 325 |
| #8 | "galactooligosaccharide"[Title/Abstract] | 363 |
| #9 | "lactosucrose"[Title/Abstract] | 67 |
| #10 | "fructooligosaccharide"[Title/Abstract] | 712 |
| #11 | "dextrins"[MeSH Terms] | 17,363 |
| #12 | "polydextrose"[Title/Abstract] | 299 |
| #13 | "chitin"[Title/Abstract] | 12,721 |
| #14 | "Lignin"[Title/Abstract] | 20,039 |
| #15 | "glucans"[MeSH Terms] | 190,890 |
| #16 | "hemicellulose"[Title/Abstract] | 6,188 |
| #17 | "cellulose"[MeSH Terms] | 57,241 |
| #18 | "inulin"[MeSH Terms] | 7,385 |
| #19 | "dietary supplements"[MeSH Terms] | 98,822 |
| #20 | "polysaccharides"[MeSH Terms] | 635,119 |
| #21 | #2-20をORでつないだもの(prebiotics)  "prebiotics"[MeSH Terms] OR "prebiotics"[Title/Abstract] OR "dietary Fiber"[MeSH Terms] OR "oligosaccharides"[MeSH Terms] OR "oligofructose"[Title/Abstract] OR "xylooligosaccharide"[Title/Abstract] OR "galactooligosaccharide"[Title/Abstract] OR "lactosucrose"[Title/Abstract] OR "fructooligosaccharide"[Title/Abstract] OR "dextrins"[MeSH Terms] OR "polydextrose"[Title/Abstract] OR "chitin"[Title/Abstract] OR "Lignin"[Title/Abstract] OR "glucans"[MeSH Terms] OR "hemicellulose"[Title/Abstract] OR "cellulose"[MeSH Terms] OR "inulin"[MeSH Terms] OR "dietary supplements"[MeSH Terms] OR "polysaccharides"[MeSH Terms] | 770,632 |
| #22 | "probiotics"[MeSH Terms] | 24,031 |
| #23 | “probiotics”[Title/Abstract] | 24,938 |
| #24 | "Akkermansia"[Title/Abstract] | 2,727 |
| #25 | "Leuconostoc"[Title/Abstract] | 3,187 |
| #26 | "Pediococcus"[Title/Abstract] | 2,427 |
| #27 | "bacillus subtilis"[Title/Abstract] | 33,746 |
| #28 | "bacillus licheniformis"[Title/Abstract] | 3,105 |
| #29 | "bacillus coagulans"[Title/Abstract] | 721 |
| #30 | "bacillus clausii"[Title/Abstract] | 183 |
| #31 | "bacillus cereus"[Title/Abstract] | 9,727 |
| #32 | "Escherichia Coli"[Title/Abstract] | 298,359 |
| #33 | "enterococcus faecium"[Title/Abstract] | 6,861 |
| #34 | "enterococcus faecalis"[Title/Abstract] | 13,083 |
| #35 | "streptococcus thermophilus"[Title/Abstract] | 2,130 |
| #36 | "streptococcus salivarius"[Title/Abstract] | 1,258 |
| #37 | "Saccharomyces"[Title/Abstract] | 79,694 |
| #38 | "Bifidobacterium"[Title/Abstract] | 11,567 |
| #39 | "Lactobacillus"[Title/Abstract] | 41,072 |
| #40 | #22-39をORでつないだもの(probiotics)  "probiotics"[MeSH Terms] OR “probiotics”[Title/Abstract] OR "Lactobacillus"[Title/Abstract] OR "Bifidobacterium"[Title/Abstract] OR "Saccharomyces"[Title/Abstract] OR "streptococcus salivarius"[Title/Abstract] OR "streptococcus thermophilus"[Title/Abstract] OR "enterococcus faecalis"[Title/Abstract] OR "enterococcus faecium"[Title/Abstract] OR "Escherichia Coli"[Title/Abstract] OR "bacillus cereus"[Title/Abstract] OR "bacillus clausii"[Title/Abstract] OR "bacillus coagulans"[Title/Abstract] OR "bacillus licheniformis"[Title/Abstract] OR "bacillus subtilis"[Title/Abstract] OR "Pediococcus"[Title/Abstract] OR "Leuconostoc"[Title/Abstract] OR "Akkermansia"[Title/Abstract] | 486,472 |
| #41 | "synbiotics"[MeSH Terms] OR "synbiotics"[Title/Abstract] OR "pre and probiotics"[Title/Abstract] | [2,193](https://pubmed.ncbi.nlm.nih.gov/?term=%22synbiotics%22%5BMeSH+Terms%5D+OR+%22synbiotics%22%5BTitle%2FAbstract%5D+OR+%E2%80%9Cpre+and+probiotics%22%5BTitle%2FAbstract%5D&sort=) |
| #42 | #21 AND # 40 | [58,893](https://pubmed.ncbi.nlm.nih.gov/?term=%28%22prebiotics%22%5BMeSH+Terms%5D+OR+%22prebiotics%22%5BTitle%2FAbstract%5D+OR+%22dietary+Fiber%22%5BMeSH+Terms%5D+OR+%22oligosaccharides%22%5BMeSH+Terms%5D+OR+%22oligofructose%22%5BTitle%2FAbstract%5D+OR+%22xylooligosaccharide%22%5BTitle%2FAbstract%5D+OR+%22galactooligosaccharide%22%5BTitle%2FAbstract%5D+OR+%22lactosucrose%22%5BTitle%2FAbstract%5D+OR+%22fructooligosaccharide%22%5BTitle%2FAbstract%5D+OR+%22dextrins%22%5BMeSH+Terms%5D+OR+%22polydextrose%22%5BTitle%2FAbstract%5D+OR+%22chitin%22%5BTitle%2FAbstract%5D+OR+%22Lignin%22%5BTitle%2FAbstract%5D+OR+%22glucans%22%5BMeSH+Terms%5D+OR+%22hemicellulose%22%5BTitle%2FAbstract%5D+OR+%22cellulose%22%5BMeSH+Terms%5D+OR+%22inulin%22%5BMeSH+Terms%5D+OR+%22dietary+supplements%22%5BMeSH+Terms%5D+OR+%22polysaccharides%22%5BMeSH+Terms%5D%29+AND+%28%22probiotics%22%5BMeSH+Terms%5D+OR+%22probiotics%22%5BTitle%2FAbstract%5D+OR+%22Lactobacillus%22%5BTitle%2FAbstract%5D+OR+%22Bifidobacterium%22%5BTitle%2FAbstract%5D+OR+%22Saccharomyces%22%5BTitle%2FAbstract%5D+OR+%22streptococcus+salivarius%22%5BTitle%2FAbstract%5D+OR+%22streptococcus+thermophilus%22%5BTitle%2FAbstract%5D+OR+%22enterococcus+faecalis%22%5BTitle%2FAbstract%5D+OR+%22enterococcus+faecium%22%5BTitle%2FAbstract%5D+OR+%22Escherichia+Coli%22%5BTitle%2FAbstract%5D+OR+%22bacillus+cereus%22%5BTitle%2FAbstract%5D+OR+%22bacillus+clausii%22%5BTitle%2FAbstract%5D+OR+%22bacillus+coagulans%22%5BTitle%2FAbstract%5D+OR+%22bacillus+licheniformis%22%5BTitle%2FAbstract%5D+OR+%22bacillus+subtilis%22%5BTitle%2FAbstract%5D+OR+%22Pediococcus%22%5BTitle%2FAbstract%5D+OR+%22Leuconostoc%22%5BTitle%2FAbstract%5D+OR+%22Akkermansia%22%5BTitle%2FAbstract%5D%29&sort=) |
| #43 | #41 OR #42 | [59,200](https://pubmed.ncbi.nlm.nih.gov/?term=%28%28%22prebiotics%22%5BMeSH+Terms%5D+OR+%22prebiotics%22%5BTitle%2FAbstract%5D+OR+%22dietary+Fiber%22%5BMeSH+Terms%5D+OR+%22oligosaccharides%22%5BMeSH+Terms%5D+OR+%22oligofructose%22%5BTitle%2FAbstract%5D+OR+%22xylooligosaccharide%22%5BTitle%2FAbstract%5D+OR+%22galactooligosaccharide%22%5BTitle%2FAbstract%5D+OR+%22lactosucrose%22%5BTitle%2FAbstract%5D+OR+%22fructooligosaccharide%22%5BTitle%2FAbstract%5D+OR+%22dextrins%22%5BMeSH+Terms%5D+OR+%22polydextrose%22%5BTitle%2FAbstract%5D+OR+%22chitin%22%5BTitle%2FAbstract%5D+OR+%22Lignin%22%5BTitle%2FAbstract%5D+OR+%22glucans%22%5BMeSH+Terms%5D+OR+%22hemicellulose%22%5BTitle%2FAbstract%5D+OR+%22cellulose%22%5BMeSH+Terms%5D+OR+%22inulin%22%5BMeSH+Terms%5D+OR+%22dietary+supplements%22%5BMeSH+Terms%5D+OR+%22polysaccharides%22%5BMeSH+Terms%5D%29+AND+%28%22probiotics%22%5BMeSH+Terms%5D+OR+%22probiotics%22%5BTitle%2FAbstract%5D+OR+%22Lactobacillus%22%5BTitle%2FAbstract%5D+OR+%22Bifidobacterium%22%5BTitle%2FAbstract%5D+OR+%22Saccharomyces%22%5BTitle%2FAbstract%5D+OR+%22streptococcus+salivarius%22%5BTitle%2FAbstract%5D+OR+%22streptococcus+thermophilus%22%5BTitle%2FAbstract%5D+OR+%22enterococcus+faecalis%22%5BTitle%2FAbstract%5D+OR+%22enterococcus+faecium%22%5BTitle%2FAbstract%5D+OR+%22Escherichia+Coli%22%5BTitle%2FAbstract%5D+OR+%22bacillus+cereus%22%5BTitle%2FAbstract%5D+OR+%22bacillus+clausii%22%5BTitle%2FAbstract%5D+OR+%22bacillus+coagulans%22%5BTitle%2FAbstract%5D+OR+%22bacillus+licheniformis%22%5BTitle%2FAbstract%5D+OR+%22bacillus+subtilis%22%5BTitle%2FAbstract%5D+OR+%22Pediococcus%22%5BTitle%2FAbstract%5D+OR+%22Leuconostoc%22%5BTitle%2FAbstract%5D+OR+%22Akkermansia%22%5BTitle%2FAbstract%5D%29%29+OR+%28%22synbiotics%22%5BMeSH+Terms%5D+OR+%22synbiotics%22%5BTitle%2FAbstract%5D+OR+%22pre+and+probiotics%22%5BTitle%2FAbstract%5D%29&sort=) |
| #44 | ("randomized controlled trial"[Publication Type] OR "controlled clinical trial"[Publication Type] OR "randomized"[Title/Abstract] OR "placebo"[Title/Abstract] OR "drug therapy"[MeSH Subheading] OR "randomly"[Title/Abstract] OR "trial"[Title/Abstract] OR "groups"[Title/Abstract]) NOT ("animals"[MeSH Terms] NOT "humans"[MeSH Terms]) | [4,980,857](https://pubmed.ncbi.nlm.nih.gov/?term=%28%22randomized+controlled+trial%22%5BPublication+Type%5D+OR+%22controlled+clinical+trial%22%5BPublication+Type%5D+OR+%22randomized%22%5BTitle%2FAbstract%5D+OR+%22placebo%22%5BTitle%2FAbstract%5D+OR+%22drug+therapy%22%5BMeSH+Subheading%5D+OR+%22randomly%22%5BTitle%2FAbstract%5D+OR+%22trial%22%5BTitle%2FAbstract%5D+OR+%22groups%22%5BTitle%2FAbstract%5D%29+NOT+%28%22animals%22%5BMeSH+Terms%5D+NOT+%22humans%22%5BMeSH+Terms%5D%29&sort=) |
| #45 | #1 AND #43 AND #44 | 676 |

Search database

- CENTRAL

Search Formula and Hit Count

CENTRAL search strategy (Search Data: 15 April 2023)

| #1 | MeSH descriptor: [Intensive Care Units] explode all trees | 5225 |
| --- | --- | --- |
| #2 | (“intensive care unit”):ti,ab,kw | 19845 |
| #3 | MeSH descriptor: [Critical Illness] explode all trees | 3218 |
| #4 | (“Critical Illness”):ti,ab,kw | 4441 |
| #5 | (“Critically ill”):ti,ab,kw | 8214 |
| #6 | MeSH descriptor: [Critical Care] explode all trees | 2650 |
| #7 | ("critical care"):ti,ab,kw | 4859 |
| #8 | ("intensive care"):ti,ab,kw | 28986 |
| #9 | MeSH descriptor: [Burns] explode all trees | 2093 |
| #10 | (burns):ti,ab,kw | 3611 |
| #11 | MeSH descriptor: [Sepsis] explode all trees | 6710 |
| #12 | (sepsis):ti,ab,kw | 13041 |
| #13 | MeSH descriptor: [Respiratory Distress Syndrome] explode all trees | 3262 |
| #14 | (“respiratory distress syndrome”):ti,ab,kw | 6514 |
| #15 | MeSH descriptor: [Shock] explode all trees | 3294 |
| #16 | (shock):ti,ab,kw | 12827 |
| #17 | MeSH descriptor: [Multiple Organ Failure] explode all trees | 640 |
| #18 | (“multiple organ failure”):ti,ab,kw | 1622 |
| #19 | MeSH descriptor: [Transplantation] explode all trees | 16525 |
| #20 | (transplantation):ti,ab,kw | 36823 |
| #21 | #1 OR #2 OR #3 OR #4 OR #5 OR #6 OR #7 OR #8 OR #9 OR #10 OR #11 OR #12 OR #13 OR #14 OR #15 OR #16 OR #17 OR #18 OR #19 OR #20 | 98076 |
| #22 | MeSH descriptor: [Prebiotics] explode all trees | 601 |
| #23 | (prebiotics):ti,ab,kw | 1238 |
| #24 | MeSH descriptor: [Dietary Fiber] explode all trees | 2514 |
| #25 | (dietary Fiber):ti,ab,kw | 5243 |
| #26 | MeSH descriptor: [Oligosaccharides] explode all trees | 3721 |
| #27 | (oligosaccharides):ti,ab,kw | 1131 |
| #28 | (oligofructose):ti,ab,kw | 193 |
| #29 | (xylooligosaccharide):ti,ab,kw | 27 |
| #30 | (galactooligosaccharide):ti,ab,kw | 99 |
| #31 | (lactosucrose):ti,ab,kw | 5 |
| #32 | (fructooligosaccharide):ti,ab,kw | 202 |
| #33 | MeSH descriptor: [Dextrins] explode all trees | 346 |
| #34 | (dextrins):ti,ab,kw | 45 |
| #35 | (polydextrose):ti,ab,kw | 137 |
| #36 | MeSH descriptor: [Chitin] explode all trees | 239 |
| #37 | (chitin):ti,ab,kw | 98 |
| #38 | MeSH descriptor: [Lignin] explode all trees | 12 |
| #39 | (Lignin):ti,ab,kw | 49 |
| #40 | MeSH descriptor: [Glucans] explode all trees | 4424 |
| #41 | (glucans):ti,ab,kw | 499 |
| #42 | (hemicellulose):ti,ab,kw | 33 |
| #43 | MeSH descriptor: [Cellulose] explode all trees | 985 |
| #44 | (cellulose):ti,ab,kw | 2165 |
| #45 | MeSH descriptor: [Inulin] explode all trees | 284 |
| #46 | (inulin):ti,ab,kw | 1203 |
| #47 | MeSH descriptor: [Dietary Supplements] explode all trees | 16750 |
| #48 | (dietary supplements):ti,ab,kw | 17055 |
| #49 | MeSH descriptor: [Polysaccharides] explode all trees | 19061 |
| #50 | (polysaccharides):ti,ab,kw | 1459 |
| #51 | #22 OR #23 OR #24 OR #25 OR #26 OR #27 OR #28 OR #29 OR #30 OR #31 OR #32 OR #33 OR #34 OR #35 OR #36 OR #37 OR #38 OR #39 OR #40 OR #41 OR #42 OR #43 OR #44 OR #45 OR #46 OR #47 OR #48 OR #49 OR #50 | 45414 |
| #52 | MeSH descriptor: [Probiotics] explode all trees | 3004 |
| #53 | (probiotics):ti,ab,kw | 6892 |
| #54 | MeSH descriptor: [Akkermansia] explode all trees | 11 |
| #55 | (Akkermansia):ti,ab,kw | 153 |
| #56 | MeSH descriptor: [Leuconostoc] explode all trees | 9 |
| #57 | (Leuconostoc):ti,ab,kw | 36 |
| #58 | MeSH descriptor: [Pediococcus] explode all trees | 20 |
| #59 | (Pediococcus):ti,ab,kw | 82 |
| #60 | MeSH descriptor: [Bacillus subtilis] explode all trees | 73 |
| #61 | (bacillus subtilis):ti,ab,kw | 201 |
| #62 | MeSH descriptor: [Bacillus licheniformis] explode all trees | 5 |
| #63 | (bacillus licheniformis):ti,ab,kw | 31 |
| #64 | MeSH descriptor: [Bacillus coagulans] explode all trees | 30 |
| #65 | (bacillus coagulans):ti,ab,kw | 158 |
| #66 | MeSH descriptor: [Bacillus clausii] explode all trees | 9 |
| #67 | (bacillus clausii):ti,ab,kw | 64 |
| #68 | MeSH descriptor: [Bacillus cereus] explode all trees | 8 |
| #69 | (bacillus cereus):ti,ab,kw | 38 |
| #70 | MeSH descriptor: [Escherichia coli] explode all trees | 1005 |
| #71 | (Escherichia Coli):ti,ab,kw | 3212 |
| #72 | MeSH descriptor: [Enterococcus faecium] explode all trees | 57 |
| #73 | (enterococcus faecium):ti,ab,kw | 184 |
| #74 | MeSH descriptor: [Enterococcus faecalis] explode all trees | 224 |
| #75 | (enterococcus faecalis):ti,ab,kw | 531 |
| #76 | MeSH descriptor: [Streptococcus thermophilus] explode all trees | 86 |
| #77 | (streptococcus thermophilus):ti,ab,kw | 343 |
| #78 | MeSH descriptor: [Streptococcus salivarius] explode all trees | 24 |
| #79 | (streptococcus salivarius):ti,ab,kw | 208 |
| #80 | MeSH descriptor: [Saccharomyces] explode all trees | 256 |
| #81 | (Saccharomyces):ti,ab,kw | 628 |
| #82 | MeSH descriptor: [Bifidobacterium] explode all trees | 1113 |
| #83 | (Bifidobacterium):ti,ab,kw | 3384 |
| #84 | MeSH descriptor: [Lactobacillus] explode all trees | 1539 |
| #85 | (Lactobacillus):ti,ab,kw | 6031 |
| #86 | #52 OR #53 OR #54 OR #55 OR #56 OR #57 OR #58 OR #59 OR #60 OR #61 OR #62 OR #63 OR #64 OR #65 OR #66 OR #67 OR #68 OR #69 OR #70 OR #71 OR #72 OR #73 OR #74 OR #75 OR #76 OR #77 OR #78 OR #79 OR #80 OR #81 OR #82 OR #83 OR #84 OR #85 | 14681 |
| #87 | MeSH descriptor: [Synbiotics] explode all trees | 237 |
| #88 | (synbiotics):ti,ab,kw | 584 |
| #89 | (pre and probiotics):ti,ab,kw | 496 |
| #90 | #87 OR #88 OR #89 | 1036 |
| #91 | #51 AND #86 | 4637 |
| #92 | #90 OR #91 | 5029 |
| #93 | #22 AND #92 | 370 |

Search database

- Igaku Chuo Zasshi

Search Formula and Hit Count

Igaku Chuo Zasshi search strategy (Search Data: 22 April 2023)

| #1 | (ICU/TH or “intensive care unit”/TA or ICU/TA) and (PT=会議録除く) | 55,436 |
| --- | --- | --- |
| #2 | (クリティカルケア/TH or 集中治療/TA) and (PT=会議録除く) | 15,549 |
| #3 | (クリティカルケア/TH or クリティカルケア/TA or クリティカル・ケア/TA) and (PT=会議録除く) | 7,487 |
| #4 | 重症患者/TA | 5,959 |
| #5 | (敗血症/TH or 敗血症/TA) and (PT=会議録除く) | 27,247 |
| #6 | (熱傷/TH or 熱傷/TA) and (PT=会議録除く) | 11,626 |
| #7 | (多臓器不全/TH or 多臓器不全/TA) and (PT=会議録除く) | 6,918 |
| #8 | (呼吸窮迫症候群/TH or 呼吸窮迫症候群/TA) and (PT=会議録除く) | 6,986 |
| #9 | (移植/TH or 移植/TA) and (PT=会議録除く) | 128,854 |
| #10 | ((ショック/TH or ショック/TA)) and (PT=会議録除く) | 37,642 |
| #11 | #1 or #2 or #3 or #4 or #5 or #6 or #7 or #8 or #9 or #10 | 260,942 |
| #12 | (プレバイオティクス/TH or プレバイオティクス/TA) and (PT=会議録除く) | 1,126 |
| #13 | (食物繊維/TH or 食物繊維/TA) and (PT=会議録除く) | 4,459 |
| #14 | (Oligosaccharides/TH or oligosaccharides/TA) and (PT=会議録除く) | 6,490 |
| #15 | (Xylooligosaccharide/TH or xylooligosaccharide/TA) and (PT=会議録除く) | 22 |
| #16 | (Galactooligosaccharide/TH or galactooligosaccharide/TA) and (PT=会議録除く) | 70 |
| #17 | (Lactosucrose/TH or lactosucrose/TA) and (PT=会議録除く) | 70 |
| #18 | (Fructooligosaccharide/TH or fructooligosaccharide/TA) and (PT=会議録除く) | 190 |
| #19 | (Dextrins/TH or dextrins/TA) and (PT=会議録除く) | 1,603 |
| #20 | (Polydextrose/TH or polydextrose/TA) and (PT=会議録除く) | 38 |
| #21 | (Chitin/TH or chitin/TA) and (PT=会議録除く) | 1,110 |
| #22 | (Lignin/TH or Lignin/TA) and (PT=会議録除く) | 117 |
| #23 | (Glucans/TH or glucans/TA) and (PT=会議録除く) | 10,013 |
| #24 | (Hemicellulose/TH or hemicellulose/TA) and (PT=会議録除く) | 65 |
| #25 | (Cellulose/TH or cellulose/TA) and (PT=会議録除く) | 2,448 |
| #26 | (Inulin/TH or inulin/TA) and (PT=会議録除く) | 480 |
| #27 | (((Polysaccharides/TH or Polysaccharides/TA)) and (PT=会議録除く)) | 46,708 |
| #28 | #12 or #13 or #14 or #15 or #16 or #17 or #18 or #19 or #20 or #21 or #22 or #23 or #24 or #25 or #26 or #27 | 51,282 |
| #29 | (プロバイオティクス/TH or プロバイオティクス/TA) and (PT=会議録除く) | 3,727 |
| #30 | ((Akkermansia/TH or Akkermansia/TA) and (PT=会議録除く)) | 58 |
| #31 | ((Leuconostoc/TH or Leuconostoc/TA) and (PT=会議録除く)) | 120 |
| #32 | ((Pediococcus/TH or Pediococcus/TA) and (PT=会議録除く)) | 123 |
| #33 | ((("Bacillus subtilis"/TH or "Bacillus subtilis"/TA)) and (PT=会議録除く)) | 796 |
| #34 | ((("Bacillus licheniformis"/TH or "Bacillus licheniformis"/TA)) and (PT=会議録除く)) | 21 |
| #35 | ((("Bacillus coagulans"/TH or "Bacillus coagulans"/TA)) and (PT=会議録除く)) | 36 |
| #36 | ((("Bacillus clausii"/TH or "Bacillus clausii"/TA)) and (PT=会議録除く)) | 2 |
| #37 | ((("Bacillus cereus"/TH or "Bacillus cereus"/TA)) and (PT=会議録除く)) | 642 |
| #38 | ((("Escherichia coli"/TH or "Escherichia Coli"/TA)) and (PT=会議録除く)) | 11,674 |
| #39 | (("Enterococcus faecium"/TH or "enterococcus faecium"/TA) and (PT=会議録除く)) | 411 |
| #40 | (("Enterococcus faecalis"/TH or "enterococcus faecalis"/TA) and (PT=会議録除く)) | 1,681 |
| #41 | (("Streptococcus thermophilus"/TH or "streptococcus thermophilus"/TA) and (PT=会議録除く)) | 60 |
| #42 | (("Streptococcus salivarius"/TH or "streptococcus salivarius"/TA) and (PT=会議録除く)) | 123 |
| #43 | (Saccharomyces/TH or Saccharomyces/TA) and (PT=会議録除く) | 1,130 |
| #44 | (Bifidobacterium/TH or Bifidobacterium/TA) and (PT=会議録除く) | 1,941 |
| #45 | (Lactobacillus/TH or Lactobacillus/TA) and (PT=会議録除く) | 2,781 |
| #46 | ("Clostridium butyricum"/TH or "Clostridium butyricum"/TA) and (PT=会議録除く) | 250 |
| #47 | #29 or #30 or #31 or #32 or #33 or #34 or #35 or #36 or #37 or #38 or #39 or #40 or #41 or #42 or #43 or #44 or #45 or #46 | 21,967 |
| #48 | (((シンバイオティクス/TH or シンバイオティクス/TA)) and (PT=会議録除く)) | 338 |
| #49 | #28 and #47 | 2,100 |
| #50 | #48 or #49 | 2,126 |
| #51 | (ランダム化比較試験/TH) or (準ランダム化比較試験/TH) or (ランダム化/AL) or (無作為化/AL) or (比較試験/AL) or (臨床試験/AL) or (プラセボ/AL) or (対照/AL) or (コントロール/AL) or (臨床研究/AL) | 349,708 |
| #52 | #11 and #50 and #51 | 49 |

**Flow diagram**

Risk of Bias

**In-hospital mortality**

**Length of ICU stay**

**Duration of mechanical ventilation**

**Infectious complication**

**All adverse events**

Forest Plot and Funnel Plot

**In-hospital mortality**

**Length of ICU stay**

**Duration of mechanical ventilation**

**Infectious complication**

**All adverse events**

**CQ2-8：What is the concept of vitamin and trace element supplementation in nutrition therapy for critically ill patients?**

Search database

- MEDLINE (PubMed/Ovid)

Search Formula and Hit Count

PubMed search strategy (Search Date: 30 April 2023)

| #1 | "Intensive Care Units"[Mesh] OR “intensive care"[tiab] OR "Critical Illness"[Mesh] OR "critical care"[Mesh] OR “critically ill”[tiab] OR "critical care"[tiab] | 308,639 |
| --- | --- | --- |
| #2 | sepsis[Mesh] OR sepsis[tiab] | 208,937 |
| #3 | trauma[tiab] | 281,331 |
| #4 | burns[Mesh] OR burn[tiab] | 76,620 |
| #5 | “respiratory distress syndrome”[Mesh] OR “respiratory distress syndrome”[tiab] OR ARDS[tiab] OR “acute respiratory distress syndrome”[tiab] OR “adult respiratory distress syndrome”[tiab] | 59,978 |
| #6 | #1 OR #2 OR #3 OR #4 OR #5 | 851,693 |
| #7 | “vitamin B1”[tiab] OR “Vit B1”[tiab] | 3,431 |
| #8 | thiamine[Mesh] OR thiamine[tiab] | 17,422 |
| #9 | “vitamin B”[tiab] OR “Vit B”[tiab] | 14,859 |
| #10 | “vitamins”[Mesh] OR “vitamin”[tiab] | 247,769 |
| #11 | #7 OR #8 OR #9 OR #10 | 258,975 |
| #12 | randomized controlled trial[pt] OR controlled clinical trial[pt] OR randomized[tiab] OR placebo[tiab] OR drug therapy[sh] OR randomly[tiab] OR trial[tiab] OR groups[tiab] NOT (animals [mh]  NOT humans [mh]) | 4,993,938 |
| #13 | #6 AND #11 AND #12 | 1,718 |

Search database

- CENTRAL

Search Formula and Hit Count

CENTRAL search strategy (searched in April 28th, 2023)

| #1 | MeSH descriptor: [Intensive Care Units] explode all trees | 5225 |
| --- | --- | --- |
| #2 | ("intensive care"):ti,ab,kw | 28987 |
| #3 | MeSH descriptor: [Critical Illness] explode all trees | 3218 |
| #4 | MeSH descriptor: [Critical Care] explode all trees | 2650 |
| #5 | ("critically ill"):ti,ab,kw | 8214 |
| #6 | (“critical illness”):ti,ab,kw | 4441 |
| #7 | ("critical care"):ti,ab,kw | 4859 |
| #8 | MeSH descriptor: [Sepsis] explode all trees | 6710 |
| #9 | (sepsis):ti,ab,kw | 13042 |
| #10 | (trauma):ti,ab,kw | 18467 |
| #11 | MeSH descriptor: [Burns] explode all trees | 2093 |
| #12 | (burn):ti,ab,kw | 4696 |
| #13 | MeSH descriptor: [Respiratory Distress Syndrome] explode all trees | 3262 |
| #14 | ("respiratory distress syndrome"):ti,ab,kw | 6514 |
| #15 | (ARDS):ti,ab,kw | 2543 |
| #16 | ("acute respiratory distress syndrome"):ti,ab,kw | 2247 |
| #17 | ("adult respiratory distress syndrome"):ti,ab,kw | 1544 |
| #18 | #1 OR #2 OR #3 OR #4 OR #5 OR #6 OR #7 OR #8 OR #9 OR #10 OR #11 OR #12 OR #13 OR #14 OR #15 OR #16 OR #17 | 72600 |
| #19 | ("vitamin B1"):ti,ab,kw | 342 |
| #20 | ("Vit B1"):ti,ab,kw | 23 |
| #21 | MeSH descriptor: [Thiamine] explode all trees | 345 |
| #22 | (thiamine):ti,ab,kw | 917 |
| #23 | ("vitamin B"):ti,ab,kw | 2745 |
| #24 | ("Vit B"):ti,ab,kw | 26 |
| #25 | MeSH descriptor: [Vitamins] explode all trees | 5667 |
| #26 | (vitamin):ti,ab,kw | 33828 |
| #27 | #19 OR #20 OR #21 OR #22 OR #23 OR #24 OR #25 OR #26 | 34800 |
| #28 | #18 AND #27 | 1174 |

Search database

- Igaku Chuo Zasshi

Search Formula and Hit Count

Igaku Chuo Zasshi search strategy (Search Data: 1 May 2023)

| #1 | (ICU/TH or "intensive care unit"/TA or ICU/TA) and (PT=会議録除く) | 55,530 |
| --- | --- | --- |
| #2 | (クリティカルケア/TH or 集中治療/TA) and (PT=会議録除く) | 15,594 |
| #3 | (クリティカルケア/TH or クリティカルケア/TA or クリティカル・ケア/TA) and (PT=会議録除く) | 7,498 |
| #4 | (重症患者/TA) and (PT=会議録除く) | 3,910 |
| #5 | (敗血症/TH or 敗血症/TA) and (PT=会議録除く) | 27,283 |
| #6 | (外傷/TA) and (PT=会議録除く) | 64,030 |
| #7 | (熱傷/TH or 熱傷/TA) and (PT=会議録除く) | 11,630 |
| #8 | (呼吸窮迫症候群/TH or 呼吸窮迫症候群/TA or ARDS/TA or 急性呼吸窮迫症候群/TA or 成人呼吸窮迫症候群/TA) and (PT=会議録除く) | 10,578 |
| #9 | #1 or #2 or #3 or #4 or #5 or #6 or #7 or #8 | 172,221 |
| #10 | (thiamine/TH or thiamine/TA or チアミン/TA) and (PT=会議録除く) | 2,278 |
| #11 | ("vitamin B1"/TA or "Vit B1"/TA or ビタミンB1/TA) and (PT=会議録除く) | 3,461 |
| #12 | ("vitamin B"/TA or "vit B"/TA or ビタミンB/TA) and (PT=会議録除く) | 5,156 |
| #13 | (vitamins/TH or vitamin/TA or ビタミン/TA) and (PT=会議録除く) | 63,228 |
| #14 | #10 or #11 or #12 or #13 | 63,730 |
| #15 | #9 and #14 | 1,086 |
| #16 | ランダム化比較試験/TH or 準ランダム化比較試験/TH or ランダム化/AL or 無作為化/AL or 比較試験/AL or 臨床試験/AL or プラセボ/AL or 対照/AL or コントロール/AL or 臨床研究/AL | 350,072 |
| #17 | #15 and #16 | 113 |

**Flow diagram**

Risk of bias summary

**Mortality**

**Length of ICU stay**

**Infectious complication**

**Length of mechanical ventilation**

**Short Form Health Survey (SF-36/8)**

**SOFA scores (at final record)**

**Changes in SOFA score**

**Delirium**

**All adverse events**

**Shock duration including vasopressors duration (hours)**

**Lactate level (mmol/L)**

Forest and Funnel Plots

**Mortality**

**Length of ICU stay**

**Infectious complication**

**Length of mechanical ventilation**

**Short Form Health Survey (SF-36/8)**

**SOFA scores (at final record)**

**Changes in SOFA score**

**Delirium**

**All adverse events**

**Shock duration including vasopressors duration (hours)**

**Lactate level (mmol/L)**

CQ2-8-Supplement：What is the concept of vitamin and trace element supplementation in nutrition therapy for critically ill patients?

In this guideline, information on vitamins and trace elements is presented as background question (BQ). In this supplement, we present the results of the systematic review and meta-analysis conducted by the guideline committee on vitamin B1, as well as recent guidelines and systematic review/meta-analyses on other vitamins and trace elements. (Provision of information for background question)

1. Vitamin B1

The guideline committee conducted a systematic review on the effect of vitamin B1 administration in critically ill patients and performed a meta-analysis using 35 RCTs [1-35]. As favorable effects, mortality was 36 fewer per 1000 patients (81 fewer to 19 more), the duration of shock, including the duration of vasoconstrictor administration, was mean difference (MD) of 11.4 hours shorter (20.2 hours shorter to 2.7 hours shorter) , blood lactate level was MD of 0.3 mmol/L lower (0.6 mmol/L lower to 0.05 mmol/L lower), SOFA score was MD of 1.3 points lower (1.9 points lower to 0.7 points lower), incidence of delirium was 67 fewer per 1000 patients (132 fewer to 26 more), and incidence of infectious complications was 45 fewer (101 fewer to 71 more). As unfavorable effects, length of ICU stay was MD of 0.4 days longer (0.01 days longer to 0.8 days longer), duration of mechanical ventilation was MD of 0.02 days longer (0.1 days shorter to 0.2 days longer), adverse events were 39 more per 1000 patients (2 fewer to 148 more), and Short Form Health Survey score was MD of 3.8 points lower (8.1 points lower to 0.5 points higher).

In the systematic review and meta-analysis, vitamin B1 administration in critically ill patients was significantly effective in improving shock and organ dysfunction. The favorable effects on the incidence of delirium and infectious complication were small. The unfavorable effect was a prolongation of ICU stay and duration of mechanical ventilation, but the difference was small. Only one study evaluated physical function, so future studies are needed.

2. Vitamin C

After the publication of the Japanese Clinical Guidelines for Management of Sepsis and Septic Shock 2020 (J-SSCG 2020) [36], several RCTs on vitamin C administration have been reported. The guideline committee of J-SSCG 2020 performed and updated systematic review in 2022. They identified 12 new RCTs and conducted an updated meta-analysis using 23 RCTs [37]. As a favorable effect, the length of ICU stay was MD of 0.3 days shorter (0.7 days shorter to 0.2 days longer). On the other hand, as unfavorable effects, long-term mortality was 42 more per 1000 patients (8 fewer to 83 more), the incidence of acute kidney injury was 6 more per 1000 patients (20 fewer to 38 more), and the length of hospital stay was MD of 0.2 days longer (1.0 days shorter to 1.5 days longer). As a result of the meta-analysis, the recommendation was revised to “We suggest against administering vitamin C to septic patients”.

A meta-analysis of 18 RCTs (n = 3364) on vitamin C administration to patients with sepsis reported in 2023 [38] showed SOFA score improvement (MD: -0.62, 95% CI: -1.00 to -0.25) and shorter duration of vasoconstrictor use (MD: -15.07, 95% CI: -21.59 to -8.55). However, there was no benefit in short-term mortality (odds ratio (OR): 0.89, 95% CI: 0.77 to 1.04), and adverse events such as organ failure or dysglycemia were significantly higher with vitamin C administration (OR: 1.98, 95% CI: 1.06 to 3.68). Subgroup analysis showed a significant improvement in SOFA score (MD: -0.9, 95% CI: -1.2 to -0.5) and a significant reduction in short-term mortality (OR: 0.80, 95% CI: 0.65 to 0.97).

A meta-analysis of 16 RCTs (n = 2130) has been conducted on vitamin C alone in critically ill patient [39]. The favorable effects included a reduction in overall mortality (risk ratio (RR): 0.73, 95% CI: 0.60 to 0.89) and 28-day mortality (RR: 0.70, 95% CI: 0.53 to 0.95), a shorter duration of vasoconstrictor administration (MD: -0.8 days, 95% CI: -1.2 to -0.3). On the other hand, long-term (longest observation period of 60 days or longer) mortality (RR: 0.98, 95% CI: 0.82 to 1.19), duration of mechanical ventilation (MD: -1.7 days, 95% CI: -5.9to 2.5), length of ICU stay (MD: -0.6 days, 95% CI: -2.0 to 0.75), length of hospital stay (MD: -0.6 days, 95% CI: -4.0 to 2.9), incidence of acute kidney injury (RR: 0.95, 95% CI: 0.81 to 1.11), renal replacement therapy (RR: 2.00, 95 CI: 0.59 to 6.77), SOFA score at 96 hours (MD: -0.8 points, 95% CI: -2.0 to 0.14), vasoconstrictor dose (standardized MD: -0.3, 95% CI: -0.8 to 0.3), and adverse events (RR: 1.04, 95% CI: 0.90 to 1.20) were not affected by administration of vitamin C alone.

3. Vitamin D

J-SSCG 2020 presents the results of a meta-analysis of 11 RCTs on the effects of vitamin D [36]. As favorable effects, 28- or 30-day mortality was 8 fewer per 1000 patients (50 fewer to 46 more), 90-day mortality was 28 more per 1000 patients (18 fewer to 85 more), and in-hospital mortality was 95 fewer per 1000 patients (180 fewer to 41 more), and the length of hospital stay was MD of 0.3 days shorter (2.2 days shorter to 1.5 days longer; 16 RCTs, 3,534 patients). As Unfavorable effects, long-term mortality was 42 more per 1000 patients (8 more to 83 more), incidence of acute kidney injury was 6 more per 1000 patients (20 fewer to 38 more), and length of hospital stay ways MD of 0.2 days longer (1.0 days shorter to 1.5 days longer), and hypercalcemia was 7 more per 1000 patients (20 fewer to 65 more). In a subgroup analysis of vitamin D-deficient patients, 28- or 30-day mortality was also 3 fewer per 1000 patients (60 fewer to 82 more), and 90-day mortality was 29 more per 1000 patients (19 fewer to 89 more). Based on these results, the J-SSCG 2020 stated "We suggest against providing vitamin D in septic patients”.

A meta-analysis of 16 RCTs (n = 2449) on vitamin D administration in critically ill patients was reported in 2022 [40]. Although the route of administration (intravenous, intravenous, or intramuscular) and dose (120,000-540,000 IU) varied, the desirable effects of vitamin D administration included reduced mortality (RR: 0.78, 95% CI: 0.62 to 0.97), shorter length of ICU stay (MD: -3.1 days, 95% CI: -5.4 to -0.9) and shorter duration of mechanical ventilation (MD: -5.1 days, 95% CI: -7.4 to -2.7). Subgroup analysis suggested that intravenous administration may be more useful than oral or enteral administration, but the risk of bias in each study was considered high.

4. Trace elements

Systematic review and meta-analyses on trace element administration in critically ill patients have been conducted mainly on selenium. A meta-analysis of 24 RCTs was reported in 2023 [41], administration of selenium reduced all-cause mortality (RR: 0.83, 95% CI: 0.71 to 0.98) and incidence of acute kidney injury (RR: 0.67, 95% CI: 0. 46 to 0.98). However, there was no favorable effect of selenium administration on 28-day mortality, risk of infection, pneumonia, length of ICU stay, length of hospital stay, or duration of mechanical ventilation.

For zinc, a meta-analysis of two RCTs (n = 168) has been reported, but no effect of zinc on improving mortality (RR: 0.73, 95% CI: 0.41 to 1.28) was presented [42]. A meta-analysis using 7 RCTs has been reported on the effect of zinc administration in trauma patients [43]. There was no benefit in mortality (OR: 0.76, 95% CI: 0.49 to 1.16) and length of hospital stay (standardized MD: -0.2 days, 95% CI: -0.5 to 0.1), but it reduced the incidence of pneumonia (OR: 0.51, 95% CI: 0.29 to 0.88).

References

1	Luger M, Hiesmayr M, Koppel P, Sima B, Ranz I, Weiss C, et al. Influence of intravenous thiamine supplementation on blood lactate concentration prior to cardiac surgery: A double-blinded, randomised controlled pilot study. Eur J Anaesthesiol. 2015;32:543-8.

2	Andersen LW, Holmberg MJ, Berg KM, Chase M, Cocchi MN, Sulmonte C, et al. Thiamine as an adjunctive therapy in cardiac surgery: a randomized, double-blind, placebo-controlled, phase II trial. Crit Care. 2016;20:92.

3	Donnino MW, Andersen LW, Chase M, Berg KM, Tidswell M, Giberson T, et al. Randomized, Double-Blind, Placebo-Controlled Trial of Thiamine as a Metabolic Resuscitator in Septic Shock: A Pilot Study. Crit Care Med. 2016;44:360-7.

4	Moskowitz A, Andersen LW, Cocchi MN, Karlsson M, Patel PV, Donnino MW. Thiamine as a Renal Protective Agent in Septic Shock. A Secondary Analysis of a Randomized, Double-Blind, Placebo-controlled Trial. Ann Am Thorac Soc. 2017;14:737-41.

5	Balakrishnan M, Gandhi H, Shah K, Pandya H, Patel R, Keshwani S, et al. Hydrocortisone, Vitamin C and thiamine for the treatment of sepsis and septic shock following cardiac surgery. Indian J Anaesth. 2018;62:934-9.

6	Harun NF, Cheah SK, Yusof AM, Lau CL, Masdar A, Nidzwani S, et al. Intravenous thiamine as adjuvant therapy for hyperlactatemia in septic shock patients. Crit Care Shock. 2019;22:288-98.

7	Chang P, Liao Y, Guan J, Guo Y, Zhao M, Hu J, et al. Combined Treatment With Hydrocortisone, Vitamin C, and Thiamine for Sepsis and Septic Shock: A Randomized Controlled Trial. Chest. 2020;158:174-82.

8	Grossestreuer AV, Moskowitz A, Andersen LW, Holmberg MJ, Konacki V, Berg KM, et al. Effect of Ascorbic Acid, Corticosteroids, and Thiamine on Health-Related Quality of Life in Sepsis. Crit Care Explor. 2020;2:e0270.

9	Hwang SY, Ryoo SM, Park JE, Jo YH, Jang DH, Suh GJ, et al. Combination therapy of vitamin C and thiamine for septic shock: a multi-centre, double-blinded randomized, controlled study. Intensive Care Med. 2020;46:2015-25.

10	Iglesias J, Vassallo AV, Patel VV, Sullivan JB, Cavanaugh J, Elbaga Y. Outcomes of Metabolic Resuscitation Using Ascorbic Acid, Thiamine, and Glucocorticoids in the Early Treatment of Sepsis: The ORANGES Trial. Chest. 2020;158:164-73.

11	Mohamed ZU, Prasannan P, Moni M, Edathadathil F, Prasanna P, Menon A, et al. Vitamin C Therapy for Routine Care in Septic Shock (ViCTOR) Trial: Effect of Intravenous Vitamin C, Thiamine, and Hydrocortisone Administration on Inpatient Mortality among Patients with Septic Shock. Indian J Crit Care Med. 2020;24:653-61.

12	Moskowitz A, Huang DT, Hou PC, Gong J, Doshi PB, Grossestreuer AV, et al. Effect of Ascorbic Acid, Corticosteroids, and Thiamine on Organ Injury in Septic Shock: The ACTS Randomized Clinical Trial. JAMA. 2020;324:642-50.

13	Moslemi R, Khalili H, Mohammadi M, Mehrabi Z, Mohebbi N. Thiamine for Prevention of Postoperative Delirium in Patients Undergoing Gastrointestinal Surgery: A Randomized Clinical Trial. J Res Pharm Pract. 2020;9:30-5.

14	Nasution AH, Yulianda RS. The Effect of Thiamine Administration on Interleukin-6 (Il-6) Enzyem, Lactate and Sequential Organ Failure Assessment (SOFA) Score in Patients with Sepsis. J Drug Alcohol Res. 2020;9:1-5.

15	Petsakul S, Morakul S, Tangsujaritvijit V, Kunawut P, Singhatas P, Sanguanwit P. Effects of thiamine on vasopressor requirements in patients with septic shock: a prospective randomized controlled trial. BMC Anesthesiol. 2020;20:280.

16	Reddy PR, Samavedam S, Aluru N, Yelle S, Rajyalakshmi B. Metabolic Resuscitation Using Hydrocortisone, Ascorbic Acid, and Thiamine: Do Individual Components Influence Reversal of Shock Independently? Indian J Crit Care Med. 2020;24:649-52.

17	Wani SJ, Mufti SA, Jan RA, Shah SU, Qadri SM, Khan UH, et al. Combination of vitamin C, thiamine and hydrocortisone added to standard treatment in the management of sepsis: results from an open label randomised controlled clinical trial and a review of the literature. Infect Dis (Lond). 2020;52:271-8.

18	Berg KM, Grossestreuer AV, Andersen LW, Liu X, Donnino MW. The Effect of a Single Dose of Thiamine on Oxygen Consumption in Patients Requiring Mechanical Ventilation for Acute Illness: A Phase II, Randomized, Double-Blind, Placebo-Controlled Trial. Crit Care Explor. 2021;3:e0579.

19	Deane AM, Jiang A, Tascone B, Clancy A, Finnis ME, Collie JT, et al. A multicenter randomized clinical trial of pharmacological vitamin B1 administration to critically ill patients who develop hypophosphatemia during enteral nutrition (The THIAMINE 4 HYPOPHOSPHATEMIA trial). Clin Nutr. 2021;40:5047-52.

20	Hussein AA, Sabry NA, Abdalla MS, Farid SF. A prospective, randomised clinical study comparing triple therapy regimen to hydrocortisone monotherapy in reducing mortality in septic shock patients. Int J Clin Pract. 2021;75:e14376.

21	Jamshidi MR, Zeraati MR, Forouzanfar B, Tahrekhani M, Motamed N. Effects of triple combination of hydrocortisone, thiamine, and Vitamin C on clinical outcome in patients with septic shock: A single-center randomized controlled trial. J Res Med Sci. 2021;26:47.

22	Kamel NA, Soliman MM, Abo-Zeid MA, Shaaban MI. Effect of Anti-Inflammatory and Antimicrobial Cosupplementations on Sepsis Prevention in Critically Ill Trauma Patients at High Risk for Sepsis. Front Pharmacol. 2021;12:792741.

23	Lubis B, Ganie RA, Lelo A, Wijaya DW, Munir D, Nasution AR. Combination Therapy of Vitamin C and Thiamine on Matrix Metalloproteinases-9 (Mmp-9) for Septic in ICU. J Drug Alcohol Res. 2021;10:1-5.

24	Lubis B, Lelo A, Amelia P, Nasution AH, Hamdi T. Effects of Thiamine on Balance between Matrix Metalloproteinas-es-9 (Mmp-9) and Tissue Inhibitors of Metalloproteinases-1 (TIMP-1). J Drug Alcohol Res. 2021;10:1-4.

25	Raghu K, Ramalingam K. Safety and Efficacy of Vitamin C, Vitamin B1, and Hydrocortisone in clinical outcome of septic shock receiving standard care: A quasi experimental randomized open label two arm parallel group study. Eur J Mol Clin Med. 2021;8:873-91.

26	Sevransky JE, Rothman RE, Hager DN, Bernard GR, Brown SM, Buchman TG, et al. Effect of Vitamin C, Thiamine, and Hydrocortisone on Ventilator- and Vasopressor-Free Days in Patients With Sepsis: The VICTAS Randomized Clinical Trial. JAMA. 2021;325:742-50.

27	Ap GR, Daga MK, Mawari G, Koner BC, Singh VK, Kumar N, et al. Effect of Supplementation of Vitamin C and Thiamine on the Outcome in Sepsis: South East Asian Region. J Assoc Physicians India. 2022;70:11-2.

28	Lubis B, Lelo A, Amelia P, Prima A. The Effect of Thiamine, Ascorbic Acid, and the Combination of Them on the Levels of Matrix Metalloproteinase-9 (MMP-9) and Tissue Inhibitor of Matrix Metalloproteinase-1 (TIMP-1) in Sepsis Patients. Infect Drug Resist. 2022;15:5741-51.

29	Lyu QQ, Zheng RQ, Chen QH, Yu JQ, Shao J, Gu XH. Early administration of hydrocortisone, vitamin C, and thiamine in adult patients with septic shock: a randomized controlled clinical trial. Crit Care. 2022;26:295.

30	Nandhini N, Malviya D, Parashar S, Pandey C, Nath SS, Tripathi M. Comparison of the effects of vitamin C and thiamine on refractory hypotension in patients with sepsis: A randomized controlled trial. Int J Crit Illn Inj Sci. 2022;12:138-45.

31	Park JE, Jo YH, Hwang SY, Kim WY, Ryoo SM, Jang DH, et al. Biomarker Analysis for Combination Therapy of Vitamin C and Thiamine in Septic Shock: A Post-Hoc Study of the ATESS Trial. Shock. 2022;57:81-7.

32	Collie JTB, Jiang A, Abdelhamid YA, Ankravs M, Bellomo R, Byrne KM, et al. Relationship of blood thiamine pyrophosphate to plasma phosphate and the response to enteral nutrition plus co-administration of intravenous thiamine during critical illness. J Hum Nutr Diet. 2023;36:1214-24.

33	Mohamed A, Abdelaty M, Saad MO, Shible A, Mitwally H, Akkari AR, et al. Evaluation of Hydrocortisone, Vitamin C, and Thiamine for the Treatment of Septic Shock: A Randomized Controlled Trial (the Hyvits Trial). Shock. 2023;59:697-701.

34	Roberson SW, Nwosu S, Collar EM, Kiehl AL, Harrison FE, Bastarache J, et al. Association of Vitamin C, Thiamine, and Hydrocortisone Infusion With Long-term Cognitive, Psychological, and Functional Outcomes in Sepsis Survivors: A Secondary Analysis of the Vitamin C, Thiamine, and Steroids in Sepsis Randomized Clinical Trial. JAMA Netw Open. 2023;6:e230380.

35	Wang J, Song Q, Yang S, Wang H, Meng S, Huang L, et al. Effects of hydrocortisone combined with vitamin C and vitamin B1 versus hydrocortisone alone on microcirculation in septic shock patients: A pilot study. Clin Hemorheol Microcirc. 2023;84:111-23.

36	Egi M, Ogura H, Yatabe T, Atagi K, Inoue S, Iba T, et al. The Japanese Clinical Practice Guidelines for Management of Sepsis and Septic Shock 2020 (J-SSCG 2020). J Intensive Care. 2021;9:53.

37	Guideline committee of The Japanese Clinical Practice Guidelines for Management of Sepsis and Septic Shock 2020, Japanese Society of Intensive Care Medicine & Japanese Association for Acute Medicine. The revised recommendation for administering vitamin C in septic patients: the Japanese Clinical Practice Guidelines for Management of Sepsis and Septic Shock 2020. J Intensive Care. 2022;10(1):50.

38	Liang B, Su J, Shao H, Chen H, Xie B. The outcome of IV vitamin C therapy in patients with sepsis or septic shock: a meta-analysis of randomized controlled trials. Crit Care. 2023;27:109.

39	Lee ZY, Ortiz-Reyes L, Lew CCH, Hasan MS, Ke L, Patel JJ, et al. Intravenous vitamin C monotherapy in critically ill patients: a systematic review and meta-analysis of randomized controlled trials with trial sequential analysis. Ann Intensive Care. 2023;13:14.

40	Ministry of Health, Labour and Welfare. Dietary Reference Intakes for Japanese (2020). https://www.mhlw.go.jp/content/10900000/001150922.pdf (Cited 2023 Jun 14)

41	Jaff S, Zeraattalab-Motlagh S, Amiri Khosroshahi R, Gubari M, Mohammadi H, Djafarian K. The effect of selenium therapy in critically ill patients: an umbrella review of systematic reviews and meta-analysis of randomized controlled trials. Eur J Med Res. 2023;28:104.

42	Vesterlund GK, Jensen TS, Ellekjaer KL, Moller MH, Thomsen T, Perner A. Effects of magnesium, phosphate, or zinc supplementation in intensive care unit patients-A systematic review and meta-analysis. Acta Anaesthesiol Scand. 2023;67:264-76.

43	Huang SY, Huang JF, Chan SY, Ou Yang CH, Cheng CT, Wang CC, et al. Effect of zinc supplement on patients with trauma: A systematic review and meta-analysis. JPEN J Parenter Enteral Nutr. 2023; 47:595-602.

**CQ3-2: Should indirect calorimetry be used to estimate energy expenditure in critically ill patients?**

Search Database

- MEDLINE (PubMed/Ovid)

Search Strategy and Hits:

PubMed search strategy （Search Date: March 31, 2023）

| #1 | "Critical Illness"[MeSH Terms] OR "critical care"[MeSH Terms] OR "critical*"[Title/Abstract] OR "severe"[Title/Abstract] OR "intensive care units"[MeSH Terms] OR "intensive"[Title/Abstract] OR "ICU"[Title/Abstract] OR "CCU"[Title/Abstract] OR "coronary care unit*"[Title/Abstract] OR "HDU"[Title/Abstract] OR "RCU"[Title/Abstract] OR "respiratory care unit*"[Title/Abstract] OR "ITU"[Title/Abstract] OR "burn unit*"[Title/Abstract] OR "burn center*"[Title/Abstract] OR "recovery room*"[Title/Abstract] OR "respiration, artificial"[MeSH Terms] OR "Artificial Respiration"[Title/Abstract:~3] OR "Artificial Respirations"[Title/Abstract:~3] OR "Ventilation Mechanical"[Title/Abstract:~3] OR "Ventilations Mechanical"[Title/Abstract:~3] OR "shock"[MeSH Terms] OR "circulatory failure"[Title/Abstract:~3] OR "circulatory collapse"[Title/Abstract:~3] OR "multiple organ"[Title/Abstract] OR "MODS"[Title/Abstract] OR "Respiratory Insufficiency"[MeSH Terms] OR "respiratory failure"[Title/Abstract:~3] OR "respiratory failures"[Title/Abstract:~3] OR "respiratory depression"[Title/Abstract:~3] OR "ventilatory depression"[Title/Abstract:~3] OR "sepsis"[MeSH Terms] OR "sepsis"[Title/Abstract] OR "septic"[Title/Abstract] OR  "Wounds and Injuries"[mesh] OR burn*[tiab] OR trauma*[tiab] OR Injur*[tiab] OR wound*[tiab] | 4,537,953 |
| --- | --- | --- |
| #2 | "calorimetry, indirect"[MeSH Terms] OR "Indirect Calorimetry"[Title/Abstract:~3] OR "indirect calorimet*"[Title/Abstract] OR "Calorimetry Respiration"[Title/Abstract:~3] OR "calorimetry respiration*"[Title/Abstract] OR "metabolic chamber"[Title/Abstract] OR "indirect energy measurement"[Title/Abstract:~3] OR "indirect calorie measurement"[Title/Abstract:~3] | 9,078 |
| #3 | "randomized controlled trial"[Publication Type] OR "controlled clinical trial"[Publication Type] OR "randomized"[Title/Abstract] OR "placebo"[Title/Abstract] OR "drug therapy"[MeSH Subheading] OR "randomly"[Title/Abstract] OR "trial"[Title/Abstract] OR "groups"[Title/Abstract]) NOT ("animals"[MeSH Terms] NOT "humans"[MeSH Terms] | 4,988,719 |
| #4 | #1 and #2 and #3 | 421 |
| #5 | "1940/01/01"[Date - Create] : "2023/03/31"[Date - Create]) and #4 | 421 |

Search Database

- CENTRAL

Search Strategy and Hits:

CENTRAL search strategy （Search Date: March 31, 2023）

| #1 | [mh "Critical Illness"] or [mh "critical care"] or [mh "intensive care units"] or [mh "respiration, artificial"] or [mh shock] or [mh "respiratory insufficiency"] or [mh sepsis] or [mh "Wounds and Injuries"] | 58781 |
| --- | --- | --- |
| #2 | (critical*):ti,ab,kw or (severe):ti,ab,kw or (ICU):ti,ab,kw or (CCU):ti,ab,kw or (HDU):ti,ab,kw or (ITU):ti,ab,kw or (burn near/3 (unit* or center*)):ti,ab,kw or (recovery room*):ti,ab,kw or (artificial near/3 respirat*):ti,ab,kw or (ventilat* near/3 mechanical):ti,ab,kw or (circulat* near/3 (failure* or collapse*)):ti,ab,kw or ("multiple organ"):ti,ab,kw or (MODS):ti,ab,kw or (respirat* near/3 (failure* or depression*)):ti,ab,kw or (ventilat* near/3 (failure* or depression*)):ti,ab,kw or (sepsis):ti,ab,kw or (septic):ti,ab,kw or ("coronary care" unit*):ti,ab,kw or (RCU):ti,ab,kw or ("respiratory care" unit*):ti,ab,kw (burn*):ti,ab,kw or (trauma*):ti,ab,kw or (Injur*):ti,ab,kw or (wound*):ti,ab,kw (Word variations have been searched) | 428503 |
| #3 | (intensive):ti,ab,kw | 50902 |
| #4 | {OR #1-#3} | 466397 |
| #5 | [mh "calorimetry, indirect"] | 522 |
| #6 | (indirect* near/3 calorimetr*):ti,ab,kw or (calorimetr* near/3 respirat*):ti,ab,kw or (metabolic chamber):ti,ab,kw or (indirect near/2 energy near/2 measurement):ti,ab,kw or (indirect near/2 calorie near/2 measurement):ti,ab,kw (Word variations have been searched) | 2742 |
| #7 | {OR #5-#6} | 2742 |
| #8 | #4 and #7 in Trials | 532 |

**Search Database**

■Igaku Chuo Zasshi

**Search Strategy and Hits:**

Igaku Chuo Zasshi search strategy（Search Date: March 31, 2023）

| #1 | (ICU/TH or “intensive care unit”/TA or ICU/TA) and (PT=会議録除く) | 55436 |
| --- | --- | --- |
| #2 | (クリティカルケア/TH or クリティカルケア/TA or クリティカル・ケア/TA or 集中治療/TA) and (PT=会議録除く) | 16525 |
| #3 | 重症患者/TA | 5959 |
| #4 | (熱傷/TH or 熱傷/TA) and (PT=会議録除く) | 11626 |
| #5 | (創傷と損傷/TH or 外傷/TA) and (PT=会議録除く) | 256932 |
| #6 | (膵炎/TH or 膵炎/TA) and (PT=会議録除く) | 21529 |
| #7 | #1 or #2 or #3 or #4 or #5 or #6 | 346279 |
| #8 | ランダム化比較試験/TH or 準ランダム化比較試験/TH or ランダム化/AL or 無作為化/AL or 比較試験/AL or 臨床試験/AL or プラセボ/AL or 対照/AL or コントロール/AL or 臨床研究/AL | 349708 |
| #9 | (経腸栄養/TH or 経腸栄養/AL or 経腸栄養/TA or 経鼻栄養/AL or 経鼻栄養/TA) and (PT=会議録除く) | 15957 |
| #10 | (胃/TH or 胃/AL or 胃/TA) and (PT=会議録除く) | 200909 |
| #11 | (十二指腸/TH or 十二指腸/AL or 十二指腸/TA or 小腸/TH or 小腸/AL or 小腸/TA or 空腸/TH or 空腸/AL or 空腸/TA or 幽門/TH or 幽門/AL or 幽門/TA) and (PT=会議録除く) | 92751 |
| #12 | #7 and #8 and #9 and #10 and #11 | 15 |

Flow Diagram

Risk of Bias

Short-term mortality

Length of ICU stay

Duration of mechanical ventilation

All infections

Ventilator-associated pneumonia

Adverse events (kidney)

Adverse events (liver)

Forest Plot and Funnel Plot

Short-term mortality

Length of ICU stay

Duration of mechanical ventilation

All infections

Ventilator-associated pneumonia

Adverse events (kidney)

Adverse events (liver)

**CQ4-3: Should higher than standard protein doses (>2.0 g/kg/day) be given to critically ill pediatric patients?**

Search database

- MEDLINE (PubMed/Ovid)

Search Strategy and Hits:

PubMed search strategy （Search Date: April 26, 2023）

| #1 | (“child”[MeSH Terms] OR “adolescent”[MeSH Terms] OR “adolescent, hospitalized”[MeSH Terms] OR “infant”[MeSH Terms] OR “child, hospitalized”[MeSH Terms] OR “neonate”[Title/Abstract] OR “newborn”[Title/Abstract] OR “infant*“[Title/Abstract] OR “toddler”[Title/Abstract] OR “child*“[Title/Abstract] OR “adolescent*“[Title/Abstract] OR paediatric*[Title/Abstract] OR pediatric*[Title/Abstract] OR “pediatrics”[MeSH Terms]) NOT (“infant, premature”[MeSH Terms] OR “infant, very low birth weight”[MeSH Terms]) | 4,508,827 |
| --- | --- | --- |
| #2 | "intensive care units"[MeSH Terms] OR "intensive care"[Title/Abstract] OR "critical illness"[MeSH Terms] OR "critical care"[MeSH Terms] OR "critically ill"[Title/Abstract] OR "critical care"[Title/Abstract] OR "PICU"[Title/Abstract] OR "PCCU"[Title/Abstract] OR "heart defects, congenital"[MeSH Terms] OR "cardiopulmonary bypass"[MeSH Terms] OR "thoracic surgery"[MeSH Terms] OR "cardiac surgical procedures"[MeSH Terms] OR "congenital heart disease"[Title/Abstract] OR "congenital heart surgery"[Title/Abstract] | 713,120 |
| #3 | "diet, high protein"[MeSH Terms] OR "dietary proteins"[MeSH Terms] OR "dietary supplements"[MeSH Terms] OR "amino acids"[MeSH Terms] OR "nitrogen"[MeSH Terms] OR "nutritional support"[MeSH Terms] OR "infant formula"[MeSH Terms] OR "energy intake"[MeSH Terms] OR "protein deficiency"[MeSH Terms] OR "high protein diet"[Title/Abstract] OR "dietary proteins"[Title/Abstract] OR "dietary supplements"[Title/Abstract] OR "nutritional support"[Title/Abstract] OR "enteral nutrition"[Title/Abstract] OR "infant formula"[Title/Abstract] OR "energy intake"[Title/Abstract] OR "protein deficiency"[Title/Abstract] OR "protein deficient"[Title/Abstract] | 1,236,084 |
| #4 | ("randomized controlled trial"[Publication Type] OR "controlled clinical trial"[Publication Type] OR "randomized"[Title/Abstract] OR "placebo"[Title/Abstract] OR "drug therapy"[MeSH Subheading] OR "randomly"[Title/Abstract] OR "trial"[Title/Abstract] OR "groups"[Title/Abstract]) NOT ("animals"[MeSH Terms] NOT "humans"[MeSH Terms]) | 4,992,902 |
| #5 | #1 AND #2 AND #3 AND #4 | 1,187 |

Search database

- CENTRAL

Search Strategy and Hits:

CENTRAL search strategy （Search Date: April 20, 2023）

| #1 | MeSH descriptor: [Child] explode all trees | 77476 |
| --- | --- | --- |
| #2 | MeSH descriptor: [Adolescent] explode all trees | 125165 |
| #3 | MeSH descriptor: [Adolescent, Hospitalized] explode all trees | 9 |
| #4 | MeSH descriptor: [Infant] explode all trees | 41440 |
| #5 | MeSH descriptor: [Child, Hospitalized] explode all trees | 172 |
| #6 | MeSH descriptor: [Pediatrics] explode all trees | 1178 |
| #7 | (neonate):ti,ab,kw | 2245 |
| #8 | (newborn):ti,ab,kw | 31652 |
| #9 | (infant):ti,ab,kw | 59253 |
| #10 | (toddler):ti,ab,kw | 1095 |
| #11 | (child):ti,ab,kw | 174132 |
| #12 | (adolescent):ti,ab,kw | 147831 |
| #13 | (paediatric):ti,ab,kw | 38857 |
| #14 | (pediatric):ti,ab,kw | 38865 |
| #15 | (pediatrics):ti,ab,kw | 4830 |
| #16 | #1 OR #2 OR #3 OR #4 OR #5 OR #6 OR #7 OR #8 OR #9 OR #10 OR #11 OR #12 OR #13 OR #14 OR #15 | 310024 |
| #17 | MeSH descriptor: [Infant, Premature] explode all trees | 4840 |
| #18 | MeSH descriptor: [Infant, Very Low Birth Weight] explode all trees | 1171 |
| #19 | #17 OR #18 | 5343 |
| #20 | #16 NOT #19 | 304681 |
| #21 | MeSH descriptor: [Intensive Care Units] explode all trees | 5225 |
| #22 | MeSH descriptor: [Critical Illness] explode all trees | 3218 |
| #23 | MeSH descriptor: [Critical Care] explode all trees | 2650 |
| #24 | MeSH descriptor: [Heart Defects, Congenital] explode all trees | 2833 |
| #25 | MeSH descriptor: [Cardiopulmonary Bypass] explode all trees | 3314 |
| #26 | MeSH descriptor: [Thoracic Surgery] explode all trees | 198 |
| #27 | MeSH descriptor: [Cardiac Surgical Procedures] explode all trees | 15455 |
| #28 | (“intensive care”):ti,ab,kw | 28987 |
| #29 | (“critically ill”):ti,ab,kw | 8214 |
| #30 | (“critical illness”):ti,ab,kw | 4441 |
| #31 | (“critical care”):ti,ab,kw | 4859 |
| #32 | (PICU):ti,ab,kw | 1009 |
| #33 | (PCCU):ti,ab,kw | 4 |
| #34 | (“Congenital heart disease”):ti,ab,kw | 1701 |
| #35 | (“Congenital heart surgery”):ti,ab,kw | 160 |
| #36 | #21 OR #22 OR #23 OR #24 OR #25 OR #26 OR #27 OR #28 OR #29 OR #30 OR #31 OR #32 OR #33 OR #34 OR #35 | 54161 |
| #37 | MeSH descriptor: [Diet, High-Protein] explode all trees | 136 |
| #38 | MeSH descriptor: [Dietary Proteins] explode all trees | 4782 |
| #39 | MeSH descriptor: [Dietary Supplements] explode all trees | 16750 |
| #40 | MeSH descriptor: [Amino Acids] explode all trees | 25168 |
| #41 | MeSH descriptor: [Nitrogen] explode all trees | 1039 |
| #42 | MeSH descriptor: [Nutritional Support] explode all trees | 4156 |
| #43 | MeSH descriptor: [Infant Formula] explode all trees | 765 |
| #44 | MeSH descriptor: [Energy Intake] explode all trees | 6440 |
| #45 | MeSH descriptor: [Protein Deficiency] explode all trees | 311 |
| #46 | (high-protein NEAR/3 diet*):ti,ab,kw | 951 |
| #47 | (dietary-protein*):ti,ab,kw | 3722 |
| #48 | (dietary-supplement*):ti,ab,kw | 19027 |
| #49 | (nutritional-support*):ti,ab,kw | 2286 |
| #50 | (enteral-nutrition*):ti,ab,kw | 5024 |
| #51 | (infant-formula*):ti,ab,kw | 1740 |
| #52 | (energy-intak*):ti,ab,kw | 9316 |
| #53 | (protein-deficien*):ti,ab,kw | 118 |
| #54 | #37 OR #38 OR #39 OR #40 OR #41 OR #42 OR #43 OR #44 OR #45 OR #46 OR #47 OR #48 OR #49 OR #50 OR #51 OR #52 OR #53 | 65694 |
| #55 | #20 AND #36 AND #54 | 607 |

Search database

■Igaku Chuo Zasshi

Search Strategy and Hits:

Igaku Chuo Zasshi search strategy （Search Date: April 22, 2023）

| #1 | (新生児/TH or 新生児/TA) and (PT=会議録除く) | 61228 |
| --- | --- | --- |
| #2 | (乳児/TH or 乳児/TA) and (PT=会議録除く) | 41265 |
| #3 | (幼児/TH or 幼児/TA) and (PT=会議録除く) | 29011 |
| #4 | (小児/TH or 小児/TA) and (PT=会議録除く) | 207930 |
| #5 | (思春期/TH or 思春期/TA) and (PT=会議録除く) | 16907 |
| #6 | (青年/TH or 青年/TA) and (PT=会議録除く) | 8538 |
| #7 | (小学生/TH or 小学/TA) and (PT=会議録除く) | 12588 |
| #8 | (中学生/TH or 中学/TA) and (PT=会議録除く) | 12412 |
| #9 | (高校生/TH or 高校/TA or 高等学校/TA) and (PT=会議録除く) | 9985 |
| #10 | (学童/TA) and (PT=会議録除く) | 6951 |
| #11 | #1 or #2 or #3 or #4 or #5 or #6 or #7 or #8 or #9 or #10 | 319301 |
| #12 | (極低出生体重児/TH) and (PT=会議録除く) | 2964 |
| #13 | (未熟児/TH) and (PT=会議録除く) | 4531 |
| #14 | #12 or #13 | 7261 |
| #15 | #11 not #14 | 312040 |
| #16 | (小児ICU/TH or 新生児ICU/TH or PICU/TA) and (PT=会議録除く) | 8343 |
| #17 | (PCCU/TA or 小児心臓ケアユニット/TA) and (PT=会議録除く) | 3 |
| #18 | (心臓疾患-先天性/TH or 先天性心疾患/TA) and (PT=会議録除く) | 33433 |
| #19 | (心肺バイパス術/TH or 心肺バイパス術/TA) and (PT=会議録除く) | 4743 |
| #20 | (胸部外科/TH or 胸部外科/TA) and (PT=会議録除く) | 83833 |
| #21 | (心臓外科/TH or 心臓外科/TA) and (PT=会議録除く) | 43885 |
| #22 | (先天性心臓手術/TA) and (PT=会議録除く) | 15 |
| #23 | (ICU/TH or “intensive care unit”/TA or ICU/TA) and (PT=会議録除く) | 55436 |
| #24 | （集中治療/TA) and (PT=会議録除く) | 12382 |
| #25 | (クリティカルケア/TH or クリティカルケア/TA or クリティカル・ケア/TA) and (PT=会議録除く) | 7487 |
| #26 | (重症患者/TA) and (PT=会議録除く) | 3899 |
| #27 | #16 or #17 or #18 or #19 or #20 or #21 or #22 or #23 or #24 or #25 or #26 | 178341 |
| #28 | (高タンパク食/TH or 高タンパク食/TA) and (PT=会議録除く) | 520 |
| #29 | (食事性タンパク質/TH or 食事性タンパク質/TA) and (PT=会議録除く) | 6799 |
| #30 | (栄養補助食品/TH or 栄養補助食品/TA) and (PT=会議録除く) | 10093 |
| #31 | ("Amino Acids"/TH or アミノ酸/TA) and (PT=会議録除く) | 71131 |
| #32 | (窒素/TH or 窒素/TA) and (PT=会議録除く) | 16983 |
| #33 | (栄養補助/TH or 栄養補助/TA) and (PT=会議録除く) | 23076 |
| #34 | (乳児用調整乳/TH or 乳児用調整乳/TA) and (PT=会議録除く) | 1084 |
| #35 | (エネルギー摂取量/TH or エネルギー摂取量/TA) and (PT=会議録除く) | 8334 |
| #36 | (タンパク質欠乏症/TH or タンパク質欠乏症/TA) and (PT=会議録除く) | 856 |
| #37 | #28 or #29 or #30 or #31 or #32 or #33 or #34 or #35 or #36 | 129097 |
| #38 | ランダム化比較試験/TH or 準ランダム化比較試験/TH or ランダム化/AL or 無作為化/AL or 比較試験/AL or 臨床試験/AL or プラセボ/AL or 対照/AL or コントロール/AL or 臨床研究/AL | 349708 |
| #39 | #15 and #27 and #37 and #38 | 28 |

Flow Diagram

Risk of bias

Mortality

Length of ICU stay

Duration of mechanical ventilation

Diarrhea

Infection complications

Muscle mass change

Hyperproteinemia

Forest plot & Funnel plot

Mortality

Length of ICU stay

Duration of mechanical ventilation

Diarrhea

Infection complications

Muscle mass change

Hyperproteinemia

**CQ4-4: Should enteral nutrition be initiated within 48 hours of starting treatment for critically ill pediatric patients?**

Search Database

■MEDLINE (PubMed/Ovid)

Search Strategy and Hits:

PubMed search strategy （Search Date: April 25, 2023）

| #1 | "Enteral Nutrition"[mh] OR "Enteral Feed*"[tiab] OR "Force Feed*"[tiab] OR "Tube Feed*"[tiab] OR "Gastric Feed*"[tiab] OR eating[tiab] OR feed*[tiab] OR "milk feed*"[tiab] OR "breastfeed*"[tiab] OR "Nutritional Support"[tiab] OR nasogastric[tiab] OR nasoenteral[tiab] OR "percutaneous tube"[tiab] OR "oral feed*"[tiab] OR "Enteral Nutrition*"[tiab] OR "enteral support*"[tiab] OR "ED tube*"[tiab] OR "Duodenal Tube*"[tiab] OR "Postpyloric Tube*"[tiab] | 657,573 |
| --- | --- | --- |
| #2 | early[tiab] OR delayed[tiab] OR gap[tiab] OR interpose[tiab] OR "Time Factors"[mh] | 3,334,814 |
| #3 | ("child"[mh] OR "adolescent"[mh] OR "adolescent, hospitalized"[mh] OR "infant"[mh] OR "child, hospitalized"[mh] OR "neonate"[tiab] OR "newborn"[tiab] OR "infant*"[tiab] OR "toddler"[tiab] OR "child*"[tiab] OR "adolescent*"[tiab] OR "paediatric*"[tiab] OR "pediatric*"[tiab] OR "pediatrics"[mh]) NOT ("infant, premature"[mh] OR "infant, very low birth weight"[mh]) | 4,508,446 |
| #4 | "intensive care units"[mh] OR "intensive care"[tiab] OR "critical illness"[mh] OR "critical care"[mh] OR "critically ill"[tiab] OR "critical care"[tiab] OR "PICU"[tiab] OR "PCCU"[tiab] OR "heart defects, congenital"[mh] OR "cardiopulmonary bypass"[mh] OR "thoracic surgery"[mh] OR "cardiac surgical procedures"[mh] OR "congenital heart disease"[tiab] OR "congenital heart surgery"[tiab] | 713,007 |
| #5 | ("randomized controlled trial"[pt] OR "controlled clinical trial"[pt] OR "randomized"[tiab] OR "placebo"[tiab] OR "drug therapy"[sh] OR "randomly"[tiab] OR "trial"[tiab] OR "groups"[tiab]) NOT ("animals"[mh] NOT "humans"[mh]) | 4,992,267 |
| #6 | #1 AND #2 AND #3 AND #4 AND #5 | 377 |

Search Database

■CENTRAL

Search Strategy and Hits:

CENTRAL search strategy （Search Date: April 20, 2023）

| #1 | MeSH descriptor: [Enteral Nutrition] explode all trees | 2179 |
| --- | --- | --- |
| #2 | MeSH descriptor: [Nutritional Support] explode all trees | 4156 |
| #3 | MeSH descriptor: [Eating] explode all trees | 5190 |
| #4 | ("Enteral Nutrition"):ti,ab,kw | 5026 |
| #5 | ("Enteral Feed*"):ti,ab,kw | 3320 |
| #6 | ("Force Feed*"):ti,ab,kw | 13 |
| #7 | ("Tube Feed*"):ti,ab,kw | 924 |
| #8 | ("Gastric Feed*"):ti,ab,kw | 95 |
| #9 | (eating):ti,ab,kw | 21564 |
| #10 | (feed*):ti,ab,kw | 51361 |
| #11 | ("milk feed*"):ti,ab,kw | 321 |
| #12 | (breastfeed*):ti,ab,kw | 7308 |
| #13 | ("Nutritional Support"):ti,ab,kw | 2759 |
| #14 | (nasogastric):ti,ab,kw | 2592 |
| #15 | (nasoenteral):ti,ab,kw | 109 |
| #16 | ("percutaneous tube"):ti,ab,kw | 6 |
| #17 | ("oral feed*"):ti,ab,kw | 1172 |
| #18 | ("Enteral Nutrition*"):ti,ab,kw | 5026 |
| #19 | ("Enteral support*"):ti,ab,kw | 23 |
| #20 | ("ED tube*"):ti,ab,kw | 2 |
| #21 | ("Duodenal Tube*"):ti,ab,kw | 34 |
| #22 | ("Postpyloric Tube*"):ti,ab,kw | 26 |
| #23 | #1 OR #2 OR #3 OR #4 OR #5 OR #6 OR #7 OR #8 OR #9 OR #10 OR #11 OR #12 OR #13 OR #14 OR #15 OR #16 OR #17 OR #18 OR #19 OR #20 OR #21 OR #22 | 78141 |
| #24 | MeSH descriptor: [Time Factors] explode all trees | 72994 |
| #25 | (early):ti,ab,kw | 140702 |
| #26 | (delayed):ti,ab,kw | 53662 |
| #27 | (gap):ti,ab,kw | 10183 |
| #28 | (interpose):ti,ab,kw | 178 |
| #29 | #24 OR #25 OR #26 OR #27 OR #28 | 254307 |
| #30 | MeSH descriptor: [Child] explode all trees | 77476 |
| #31 | MeSH descriptor: [Adolescent] explode all trees | 125165 |
| #32 | MeSH descriptor: [Adolescent, Hospitalized] explode all trees | 9 |
| #33 | MeSH descriptor: [Infant] explode all trees | 41440 |
| #34 | MeSH descriptor: [Child, Hospitalized] explode all trees | 172 |
| #35 | MeSH descriptor: [Pediatrics] explode all trees | 1178 |
| #36 | (neonate):ti,ab,kw | 26449 |
| #37 | (newborn):ti,ab,kw | 33194 |
| #38 | (infant):ti,ab,kw | 70368 |
| #39 | (toddler):ti,ab,kw | 2101 |
| #40 | (child):ti,ab,kw | 174232 |
| #41 | (adolescent):ti,ab,kw | 158278 |
| #42 | (paediatric):ti,ab,kw | 41381 |
| #43 | (pediatric):ti,ab,kw | 41389 |
| #44 | (pediatrics):ti,ab,kw | 41380 |
| #45 | #30 OR #31 OR #32 OR #33 OR #34 OR #35 OR #36 OR #37 OR #38 OR #39 OR #40 OR #41 OR #42 OR #43 OR #44 | 329290 |
| #46 | MeSH descriptor: [Infant, Premature] explode all trees | 4840 |
| #47 | MeSH descriptor: [Infant, Very Low Birth Weight] explode all trees | 1171 |
| #48 | #46 OR #47 | 5343 |
| #49 | #45 NOT #48 | 323947 |
| #50 | MeSH descriptor: [Intensive Care Units] explode all trees | 5225 |
| #51 | MeSH descriptor: [Critical Illness] explode all trees | 3218 |
| #52 | MeSH descriptor: [Critical Care] explode all trees | 2650 |
| #53 | MeSH descriptor: [Heart Defects, Congenital] explode all trees | 2833 |
| #54 | MeSH descriptor: [Cardiopulmonary Bypass] explode all trees | 3314 |
| #55 | MeSH descriptor: [Thoracic Surgery] explode all trees | 198 |
| #56 | MeSH descriptor: [Cardiac Surgical Procedures] explode all trees | 15455 |
| #57 | ("intensive care"):ti,ab,kw | 29024 |
| #58 | ("critically ill"):ti,ab,kw | 9739 |
| #59 | (“critical illness”):ti,ab,kw | 9741 |
| #60 | ("critical care"):ti,ab,kw | 4862 |
| #61 | (PICU):ti,ab,kw | 1009 |
| #62 | (PCCU):ti,ab,kw | 4 |
| #63 | ("Congenital heart disease"):ti,ab,kw | 1778 |
| #64 | ("Congenital heart surgery"):ti,ab,kw | 164 |
| #65 | #50 OR #51 OR #52 OR #53 OR #54 OR #55 OR #56 OR #57 OR #58 OR #59 OR #60 OR #61 OR #62 OR #63 OR #64 | 54299 |
| #66 | #23 AND #29 AND #49 AND #65 | 434 (trial 419) |

Search Database

■Igaku Chuo Zasshi

Search Strategy and Hits:

Igaku Chuo Zasshi search strategy （Search Date: April 22, 2023）

| #1 | (経腸栄養/TH or 経腸栄養/TA) and (PT=会議録除く) | 14,354 |
| --- | --- | --- |
| #2 | (食事/TH or 食事/TA) and (PT=会議録除く) | 68,069 |
| #3 | (母乳/TH or 母乳/TA) and (PT=会議録除く) | 9,442 |
| #4 | (乳/TH or 乳/TA) and (PT=会議録除く) | 201,718 |
| #5 | (摂食/TH or 摂食/TA) and (PT=会議録除く) | 31,865 |
| #6 | (栄養補助/TH or 栄養補助/TA) and (PT=会議録除く) | 23,076 |
| #7 | ((胃ゾンデ/TH or 胃ゾンデ/TA or 胃管/TA) and (PT=会議録除く)) | 5,342 |
| #8 | ((十二指腸ゾンデ/TH or 十二指腸ゾンデ/TA or 十二指腸チューブ/TA or EDチューブ/TA) and (PT=会議録除く)) | 232 |
| #9 | #1 or #2 or #3 or #4 or #5 or #6 or #7 or #8 | 314,921 |
| #10 | (時間/TH or 時間/TA) and (PT=会議録除く) | 251,324 |
| #11 | (早期/TA) and (PT=会議録除く) | 154,682 |
| #12 | (晩期/TA) and (PT=会議録除く) | 6,000 |
| #13 | ((タイミング/TH or タイミング/TA)) and (PT=会議録除く) | 11,183 |
| #14 | #10 or #11 or #12 or #13 | 403,799 |
| #15 | (新生児/TH or 新生児/TA) and (PT=会議録除く) | 61,228 |
| #16 | (乳児/TH or 乳児/TA) and (PT=会議録除く) | 41,265 |
| #17 | (幼児/TH or 幼児/TA) and (PT=会議録除く) | 29,011 |
| #18 | (小児/TH or 小児/TA) and (PT=会議録除く) | 207,930 |
| #19 | (思春期/TH or 思春期/TA) and (PT=会議録除く) | 16,907 |
| #20 | (青年/TH or 青年/TA) and (PT=会議録除く) | 8,538 |
| #21 | (小学生/TH or 小学/TA) and (PT=会議録除く) | 12,588 |
| #22 | (中学生/TH or 中学/TA) and (PT=会議録除く) | 12,412 |
| #23 | (高校生/TH or 高校/TA or 高等学校/TA) and (PT=会議録除く) | 9,985 |
| #24 | (学童/TA) and (PT=会議録除く) | 6,951 |
| #25 | #15 or #16 or #17 or #18 or #19 or #20 or #21 or #22 or #23 or #24 | 319,301 |
| #26 | (極低出生体重児/TH) and (PT=会議録除く) | 2,964 |
| #27 | (未熟児/TH) and (PT=会議録除く) | 4,531 |
| #28 | #26 or #27 | 7,261 |
| #29 | #25 not #28 | 312,040 |
| #30 | (小児ICU/TH or 新生児ICU/TH or PICU/TA) and (PT=会議録除く) | 8,343 |
| #31 | (PCCU/TA or 小児心臓ケアユニット/TA) and (PT=会議録除く) | 3 |
| #32 | (心臓疾患-先天性/TH or 先天性心疾患/TA) and (PT=会議録除く) | 33,433 |
| #33 | (心肺バイパス術/TH or 心肺バイパス術/TA) and (PT=会議録除く) | 4,743 |
| #34 | (胸部外科/TH or 胸部外科/TA) and (PT=会議録除く) | 83,833 |
| #35 | (心臓外科/TH or 心臓外科/TA) and (PT=会議録除く) | 43,885 |
| #36 | (先天性心臓手術/TA) and (PT=会議録除く) | 15 |
| #37 | (ICU/TH or "intensive care unit"/TA or ICU/TA) and (PT=会議録除く) | 55,436 |
| #38 | (集中治療/TA) and (PT=会議録除く) | 12,382 |
| #39 | (クリティカルケア/TH or クリティカルケア/TA or クリティカル・ケア/TA) and (PT=会議録除く) | 7,487 |
| #40 | 重症患者/TA and (PT=会議録除く) | 3,899 |
| #41 | #30 or #31 or #32 or #33 or #34 or #35 or #36 or #37 or #38 or #39 or #40 | 178,341 |
| #42 | ランダム化比較試験/TH or 準ランダム化比較試験/TH or ランダム化/AL or 無作為化/AL or 比較試験/AL or 臨床試験/AL or プラセボ/AL or 対照/AL or コントロール/AL or 臨床研究/AL | 349,708 |
| #43 | #9 and #14 and #29 and #41 and #42 | 46 |

Flow diagram

Risk of Bias

Length of ICU stay

Adverse events

Forest Plot &　Funnel Plot

Length of ICU stay

Adverse events

**Q4-6: Should post-pyloric feeding rather than gastric feeding be used for enteral nutrition of critically ill pediatric patients?**

Search Database

- MEDLINE (PubMed/Ovid)

Search Strategy and Hits:

PubMed search strategy （Search Date: April 26, 2023）

| #1 | ("child"[MeSH Terms] OR "adolescent"[MeSH Terms] OR "adolescent, hospitalized"[MeSH Terms] OR "infant"[MeSH Terms] OR "child, hospitalized"[MeSH Terms] OR "neonate"[Title/Abstract] OR "newborn"[Title/Abstract] OR "infant*"[Title/Abstract] OR "toddler"[Title/Abstract] OR "child*"[Title/Abstract] OR "adolescent*"[Title/Abstract] OR "paediatric*"[Title/Abstract] OR "pediatric*"[Title/Abstract] OR "pediatrics"[MeSH Terms]) NOT ("infant, premature"[MeSH Terms] OR "infant, very low birth weight"[MeSH Terms]) | 4,508,827 |
| --- | --- | --- |
| #2 | "intensive care units"[MeSH Terms] OR "intensive care"[Title/Abstract] OR "critical illness"[MeSH Terms] OR "critical care"[MeSH Terms] OR "critically ill"[Title/Abstract] OR "critical care"[Title/Abstract] OR "PICU"[Title/Abstract] OR "PCCU"[Title/Abstract] OR "heart defects, congenital"[MeSH Terms] OR "cardiopulmonary bypass"[MeSH Terms] OR "thoracic surgery"[MeSH Terms] OR "cardiac surgical procedures"[MeSH Terms] OR "congenital heart disease"[Title/Abstract] OR "congenital heart surgery"[Title/Abstract] | 713,120 |
| #3 | ("randomized controlled trial"[Publication Type] OR "controlled clinical trial"[Publication Type] OR "randomized"[Title/Abstract] OR "placebo"[Title/Abstract] OR "drug therapy"[MeSH Subheading] OR "randomly"[Title/Abstract] OR "trial"[Title/Abstract] OR "groups"[Title/Abstract]) NOT ("animals"[MeSH Terms] NOT "humans"[MeSH Terms]) | 4,992,902 |
| #4 | "enteral nutrition"[MeSH Terms] OR "nutrition*"[Title/Abstract] OR "fed"[Title/Abstract] OR "feed*"[Title/Abstract] OR "tube*"[Title/Abstract] OR "intub*"[Title/Abstract] OR "milk*"[Title/Abstract] | 1,636,772 |
| #5 | "intubation, gastrointestinal"[MeSH Terms] OR "gastrostomy"[MeSH Terms] OR "g tube*"[Title/Abstract] OR "ng tube*"[Title/Abstract] OR "peg"[Title/Abstract] OR "gastr*"[Title/Abstract] OR "nasogastr*"[Title/Abstract] OR "orogastr*"[Title/Abstract] OR "stomach"[Title/Abstract] OR "gavage"[Title/Abstract] OR "oral"[Title/Abstract] OR "breastfeed*"[Title/Abstract] | 1,542,679 |
| #6 | "enterostomy"[MeSH Terms] OR "duodenostomy"[MeSH Terms] OR "jejunostomy"[MeSH Terms] OR "j tube*"[Title/Abstract] OR "nj tube*"[Title/Abstract] OR "pej"[Title/Abstract] OR "enter*"[Title/Abstract] OR "nasoenter*"[Title/Abstract] OR "oroenter*"[Title/Abstract] OR "duoden*"[Title/Abstract] OR "nasoduoden*"[Title/Abstract] OR "oroduoden*"[Title/Abstract] OR "jejun*"[Title/Abstract] OR "nasojejun*"[Title/Abstract] OR "orojejun*"[Title/Abstract] OR "bowel*"[Title/Abstract] OR "intestine*"[Title/Abstract] OR "postpylor*"[Title/Abstract] OR "post pylor*"[Title/Abstract] OR "transpylor*"[Title/Abstract] OR "trans pylor*"[Title/Abstract] | 898,355 |
| #7 | #1 AND #2 AND #3 AND #4 AND #5 AND #6 | 240 |

Search Database

- CENTRAL

Search Strategy and Hits:

CENTRAL search strategy （Search Date: April 21, 2023）

| #1 | MeSH descriptor: [Child] explode all trees | 77476 |
| --- | --- | --- |
| #2 | MeSH descriptor: [Adolescent] explode all trees | 125165 |
| #3 | MeSH descriptor: [Adolescent, Hospitalized] explode all trees | 9 |
| #4 | MeSH descriptor: [Infant] explode all trees | 41440 |
| #5 | MeSH descriptor: [Child, Hospitalized] explode all trees | 172 |
| #6 | MeSH descriptor: [Pediatrics] explode all trees | 1178 |
| #7 | (neonate):ti,ab,kw | 2246 |
| #8 | (newborn):ti,ab,kw | 31652 |
| #9 | (infant):ti,ab,kw | 59253 |
| #10 | (toddler):ti,ab,kw | 1095 |
| #11 | (child):ti,ab,kw | 174134 |
| #12 | (adolescent):ti,ab,kw | 147831 |
| #13 | (paediatric):ti,ab,kw | 38857 |
| #14 | (pediatric):ti,ab,kw | 38865 |
| #15 | (pediatrics):ti,ab,kw | 4830 |
| #16 | #1 or #2 or #3 or #4 or #5 or #6 or #7 or #8 or #9 or #10 or #11 or #12 or #13 or #14 or #15 | 310026 |
| #17 | MeSH descriptor: [Infant, Premature] explode all trees | 4840 |
| #18 | MeSH descriptor: [Infant, Very Low Birth Weight] explode all trees | 1171 |
| #19 | #17 or #18 | 5343 |
| #20 | #16 not #19 | 304683 |
| #21 | MeSH descriptor: [Intensive Care Units] explode all trees | 5225 |
| #22 | MeSH descriptor: [Critical Illness] explode all trees | 3218 |
| #23 | MeSH descriptor: [Critical Care] explode all trees | 2650 |
| #24 | MeSH descriptor: [Heart Defects, Congenital] explode all trees | 2833 |
| #25 | MeSH descriptor: [Cardiopulmonary Bypass] explode all trees | 3314 |
| #26 | MeSH descriptor: [Thoracic Surgery] explode all trees | 198 |
| #27 | MeSH descriptor: [Cardiac Surgical Procedures] explode all trees | 15455 |
| #28 | ("intensive care"):ti,ab,kw | 28987 |
| #29 | ("critically ill"):ti,ab,kw | 8214 |
| #30 | ("critical illness"):ti,ab,kw | 4441 |
| #31 | ("critical care"):ti,ab,kw | 4859 |
| #32 | (PICU):ti,ab,kw | 1009 |
| #33 | (PCCU):ti,ab,kw | 4 |
| #34 | ("congenital heart disease"):ti,ab,kw | 1701 |
| #35 | ("congenital heart surgery"):ti,ab,kw | 160 |
| #36 | #21 or #22 or #23 or #24 or #25 or #26 or #27 or #28 or #29 or #30 or #31 or #32 or #33 or #34 or #35 | 54161 |
| #37 | MeSH descriptor: [Enteral Nutrition] explode all trees | 2179 |
| #38 | (nutrition*):ti,ab,kw | 51270 |
| #39 | (fed):ti,ab,kw | 10688 |
| #40 | (feed*):ti,ab,kw | 51360 |
| #41 | (tube*):ti,ab,kw | 31726 |
| #42 | (intub*):ti,ab,kw | 23484 |
| #43 | (milk*):ti,ab,kw | 13433 |
| #44 | #37 or #38 or #39 or #40 or #41 or #42 or #43 | 150640 |
| #45 | MeSH descriptor: [Intubation, Gastrointestinal] explode all trees | 752 |
| #46 | MeSH descriptor: [Gastrostomy] explode all trees | 245 |
| #47 | (g-tube*):ti,ab,kw | 80 |
| #48 | (ng-tube*):ti,ab,kw | 233 |
| #49 | (peg):ti,ab,kw | 5838 |
| #50 | (gastr*):ti,ab,kw | 89160 |
| #51 | (nasogastr*):ti,ab,kw | 2601 |
| #52 | (orogastr*):ti,ab,kw | 327 |
| #53 | (stomach):ti,ab,kw | 21168 |
| #54 | (gavage):ti,ab,kw | 371 |
| #55 | (oral):ti,ab,kw | 194847 |
| #56 | (breastfeed*):ti,ab,kw | 7308 |
| #57 | #45 or #46 or #47 or #48 or #49 or #50 or #51 or #52 or #53 or #54 or #55 or #56 | 282473 |
| #58 | MeSH descriptor: [Enterostomy] explode all trees | 649 |
| #59 | MeSH descriptor: [Duodenostomy] explode all trees | 18 |
| #60 | MeSH descriptor: [Jejunostomy] explode all trees | 116 |
| #61 | (j-tube*):ti,ab,kw | 21 |
| #62 | (nj-tube*):ti,ab,kw | 19 |
| #63 | (pej):ti,ab,kw | 10 |
| #64 | (enter*):ti,ab,kw | 50250 |
| #65 | (nasoenter*):ti,ab,kw | 117 |
| #66 | (oroenter*):ti,ab,kw | 8 |
| #67 | (duoden*):ti,ab,kw | 8252 |
| #68 | (nasoduoden*):ti,ab,kw | 112 |
| #69 | (oroduoden*):ti,ab,kw | 4 |
| #70 | (jejun*):ti,ab,kw | 1857 |
| #71 | (nasojejun*):ti,ab,kw | 240 |
| #72 | (orojejun*):ti,ab,kw | 3 |
| #73 | (bowel*):ti,ab,kw | 22575 |
| #74 | (intestine*):ti,ab,kw | 14536 |
| #75 | (postpylor*):ti,ab,kw | 114 |
| #76 | (post-pylor*):ti,ab,kw | 72 |
| #77 | (transpylor*):ti,ab,kw | 65 |
| #78 | (trans-pylor*):ti,ab,kw | 7 |
| #79 | #58 or #59 or #60 or #61 or #62 or #63 or #64 or #65 or #66 or #67 or #68 or #69 or #70 or #71 or #72 or #73 or #74 or #75 or #76 or #77 or #78 | 85638 |
| #80 | #20 and #36 and #44 and #57 and #79 | 332 |

Search Database

■Igaku Chuo Zasshi

Search Strategy and Hits:

Igaku Chuo Zasshi search strategy （Search Date: April 21, 2023）

| #1 | (新生児/TH or 新生児/TA) and (PT=会議録除く) | 61,228 |
| --- | --- | --- |
| #2 | (乳児/TH or 乳児/TA) and (PT=会議録除く) | 41,265 |
| #3 | (幼児/TH or 幼児/TA) and (PT=会議録除く) | 29,011 |
| #4 | (小児/TH or 小児/TA) and (PT=会議録除く) | 207,930 |
| #5 | (思春期/TH or 思春期/TA) and (PT=会議録除く) | 16,907 |
| #6 | (青年/TH or 青年/TA) and (PT=会議録除く) | 8,538 |
| #7 | (小学生/TH or 小学/TA) and (PT=会議録除く) | 12,588 |
| #8 | (中学生/TH or 中学/TA) and (PT=会議録除く) | 12,412 |
| #9 | (高校生/TH or 高校/TA or 高等学校/TA) and (PT=会議録除く) | 9,985 |
| #10 | (学童/TA) and (PT=会議録除く) | 6,951 |
| #11 | #1 or #2 or #3 or #4 or #5 or #6 or #7 or #8 or #9 or #10 | 319,301 |
| #12 | (極低出生体重児/TH) and (PT=会議録除く) | 2,964 |
| #13 | (未熟児/TH) and (PT=会議録除く) | 4,531 |
| #14 | #12 or #13 | 7,261 |
| #15 | #11 not #14 | 312,040 |
| #16 | (小児ICU/TH or 新生児ICU/TH or PICU/TA) and (PT=会議録除く) | 8,343 |
| #17 | (PCCU/TA or 小児心臓ケアユニット/TA) and (PT=会議録除く) | 3 |
| #18 | (心臓疾患-先天性/TH or 先天性心疾患/TA) and (PT=会議録除く) | 33,433 |
| #19 | (心肺バイパス術/TH or 心肺バイパス術/TA) and (PT=会議録除く) | 4,743 |
| #20 | (胸部外科/TH or 胸部外科/TA) and (PT=会議録除く) | 83,833 |
| #21 | (心臓外科/TH or 心臓外科/TA) and (PT=会議録除く) | 43,885 |
| #22 | (先天性心臓手術/TA) and (PT=会議録除く) | 15 |
| #23 | (ICU/TH or "intensive care unit"/TA or ICU/TA) and (PT=会議録除く) | 55,436 |
| #24 | (集中治療/TA) and (PT=会議録除く) | 12,382 |
| #25 | (クリティカルケア/TH or クリティカルケア/TA or クリティカル・ケア/TA) and (PT=会議録除く) | 7,487 |
| #26 | (重症患者/TA) and (PT=会議録除く) | 3,899 |
| #27 | #16 or #17 or #18 or #19 or #20 or #21 or #22 or #23 or #24 or #25 or #26 | 178,341 |
| #28 | ランダム化比較試験/TH or 準ランダム化比較試験/TH or ランダム化/AL or 無作為化/AL or 比較試験/AL or 臨床試験/AL or プラセボ/AL or 対照/AL or コントロール/AL or 臨床研究/AL | 349,708 |
| #29 | (栄養生理学的現象/TH or "食事, 食物および栄養"/TH or 経腸栄養/TH or 経腸/TA or 栄養/TA) and (PT=会議録除く) | 165,236 |
| #30 | (経管/TA or チューブ/TA or カテーテル/TA) and (PT=会議録除く) | 62,390 |
| #31 | (母乳/TH or 母乳/TA or 乳児用調整乳/TH or 乳児用調整乳/TA or 人工乳/TA or ミルク/TA) and (PT=会議録除く) | 11,896 |
| #32 | #29 or #30 or #31 | 225,463 |
| #33 | (経胃/TA) and (PT=会議録除く) | 588 |
| #34 | (胃ゾンデ/TH or 胃ゾンデ/TA or 胃管/TA or 胃チューブ/TA or 胃カテーテル/TA or NGチューブ/TA or NGカテーテル/TA) and (PT=会議録除く) | 5,567 |
| #35 | (胃造瘻術/TH or 胃造瘻術/TA or 胃瘻/TH or 胃瘻/TA or PEG/TA) and (PT=会議録除く) | 10,635 |
| #36 | (経皮経食道胃管挿入術/TH or 経皮経食道胃管挿入術/TA) and (PT=会議録除く) | 321 |
| #37 | (経口/TA) and (PT=会議録除く) | 67,055 |
| #38 | (食事/TH or 食事/TA or 摂食/TH or 摂食/TA) and (PT=会議録除く) | 94,260 |
| #39 | (母乳栄養/TH or 授乳/TA or 哺乳びん栄養補給/TH or 哺乳/TA) and (PT=会議録除く) | 23,295 |
| #40 | #33 or #34 or #35 or #36 or #37 or #38 or #39 | 189,454 |
| #41 | (幽門後/TA) and (PT=会議録除く) | 27 |
| #42 | (腸造瘻術/TH or 腸造瘻術/TA or 腸瘻/TA) and (PT=会議録除く) | 10,977 |
| #43 | (十二指腸ゾンデ/TH or 十二指腸造瘻術/TH or 十二指腸/TA) and (PT=会議録除く) | 38,314 |
| #44 | (空腸造瘻術/TH or 空腸/TA) and (PT=会議録除く) | 13,035 |
| #45 | (回腸造瘻術/TH or 回腸/TA) and (PT=会議録除く) | 13,936 |
| #46 | (NJチューブ/TA or NJカテーテル/TA or PEJ/TA or PEG-J/TA or PEGJ/TA) and (PT=会議録除く) | 107 |
| #47 | #41 or #42 or #43 or #44 or #45 or #46 | 66,892 |
| #48 | #15 and #27 and #32 and #40 and #47 | 19 |

Flow diagram

Risk of bias

Mortality

Length of hospital stay

Duration of mechanical ventilation

Ventilator-associated pneumonia

Aspiration

Emesis

Enteral nutrition initiation time

Forrest plot & Funnel plot

Mortality

Length of hospital stay

Duration of mechanical ventilation

Ventilator-associated pneumonia

Aspiration

Emesis

Enteral nutrition initiation time

**CQ4-7: Should bolus feeding rather than continuous feeding be used in critically ill pediatric patients undergoing gastric feeding?**

Search Database

- MEDLINE (PubMed/Ovid)

Search Strategy and Hits:

PubMed search strategy （Search Date: April 26, 2023）

| #1 | (“child”[MeSH Terms] OR “adolescent”[MeSH Terms] OR “adolescent, hospitalized”[MeSH Terms] OR “infant”[MeSH Terms] OR “child, hospitalized”[MeSH Terms] OR “neonate”[Title/Abstract] OR “newborn”[Title/Abstract] OR “infant*“[Title/Abstract] OR “toddler”[Title/Abstract] OR “child*“[Title/Abstract] OR “adolescent*“[Title/Abstract] OR paediatric*[Title/Abstract] OR pediatric*[Title/Abstract] OR “pediatrics”[MeSH Terms]) NOT (“infant, premature”[MeSH Terms] OR “infant, very low birth weight”[MeSH Terms]) | 4,508,827 |
| --- | --- | --- |
| #2 | "intensive care units"[MeSH Terms] OR "intensive care"[Title/Abstract] OR "critical illness"[MeSH Terms] OR "critical care"[MeSH Terms] OR "critically ill"[Title/Abstract] OR "critical care"[Title/Abstract] OR "PICU"[Title/Abstract] OR "PCCU"[Title/Abstract] OR "heart defects, congenital"[MeSH Terms] OR "cardiopulmonary bypass"[MeSH Terms] OR "thoracic surgery"[MeSH Terms] OR "cardiac surgical procedures"[MeSH Terms] OR "congenital heart disease"[Title/Abstract] OR "congenital heart surgery"[Title/Abstract] | 713,120 |
| #3 | ("randomized controlled trial"[Publication Type] OR "controlled clinical trial"[Publication Type] OR "randomized"[Title/Abstract] OR "placebo"[Title/Abstract] OR "drug therapy"[MeSH Subheading] OR "randomly"[Title/Abstract] OR "trial"[Title/Abstract] OR "groups"[Title/Abstract]) NOT ("animals"[MeSH Terms] NOT "humans"[MeSH Terms]) | 4,992,902 |
| #4 | Enteral Nutrition [mh] OR “Milk, Human” [mh] OR “Food, Formulated” [mh] OR “enteral nutrition” [tiab] OR “nutrition enteral” [tiab] OR “enteral feed*” [tiab] OR “tube feed*” [tiab] OR “feeding tube” [tiab] OR “artificial feed*” [tiab] OR “artificial nutrition” [tiab] OR “force feeding” [tiab] OR “nutrition support” [tiab] OR “nutritional support” [tiab] OR “intragastric feed*” [tiab] OR “gastric feed*” [tiab] OR “gastric tube” [tiab] OR “nasogastric” [tiab] OR “nasoenteral” [tiab] OR “percutaneous tube” [tiab] OR “gastrostomy” [tiab] OR “gavage” [tiab] OR “gastrogavage” [tiab] OR “milk” [tiab] OR “food formulated” [tiab] OR “enteral diet” [tiab] OR “enteral formula” [tiab] | 233,491 |
| #5 | “bolus” [tiab] OR “intermittent*” [tiab] | 156,758 |
| #6 | “continuous*” [tiab] OR “continued” [tiab] OR “constant” [tiab] | 1,131,329 |
| #7 | #1 AND #2 AND #3 AND #4 AND (#5 OR #6) | 113 |

Search Database

- CENTRAL

Search Strategy and Hits:

CENTRAL search strategy （Search Date: April 20, 2023）

| #1 | MeSH descriptor: [Child] explode all trees | 77,476 |
| --- | --- | --- |
| #2 | MeSH descriptor: [Adolescent] explode all trees | 125,165 |
| #3 | MeSH descriptor: [Adolescent, Hospitalized] explode all trees | 9 |
| #4 | MeSH descriptor: [Infant] explode all trees | 41,440 |
| #5 | MeSH descriptor: [Child, Hospitalized] explode all trees | 172 |
| #6 | MeSH descriptor: [Pediatrics] explode all trees | 1,178 |
| #7 | (neonate):ti,ab,kw | 2,245 |
| #8 | (newborn):ti,ab,kw | 31,652 |
| #9 | (infant):ti,ab,kw | 59,253 |
| #10 | (toddler):ti,ab,kw | 1,095 |
| #11 | (child):ti,ab,kw | 174,132 |
| #12 | (adolescent):ti,ab,kw | 147,831 |
| #13 | (paediatric):ti,ab,kw | 38,857 |
| #14 | (pediatric):ti,ab,kw | 38,865 |
| #15 | (pediatrics):ti,ab,kw | 4,830 |
| #16 | #1 or #2 or #3 or #4 or #5 or #6 or #7 or #8 or #9 or #10 or #11 or #12 or #13 or #14 or #15 | 310,024 |
| #17 | MeSH descriptor: [Infant, Premature] explode all trees | 4,840 |
| #18 | MeSH descriptor: [Infant, Very Low Birth Weight] explode all trees | 1,171 |
| #19 | #17 or #18 | 5,343 |
| #20 | #16 not #19 | 304,681 |
| #21 | MeSH descriptor: [Intensive Care Units] explode all trees | 5,225 |
| #22 | MeSH descriptor: [Critical Illness] explode all trees | 3,218 |
| #23 | MeSH descriptor: [Critical Care] explode all trees | 2,650 |
| #24 | MeSH descriptor: [Heart Defects, Congenital] explode all trees | 2,833 |
| #25 | MeSH descriptor: [Cardiopulmonary Bypass] explode all trees | 3,314 |
| #26 | MeSH descriptor: [Thoracic Surgery] explode all trees | 198 |
| #27 | MeSH descriptor: [Cardiac Surgical Procedures] explode all trees | 15,455 |
| #28 | ("intensive care"):ti,ab,kw | 28,987 |
| #29 | ("critically ill"):ti,ab,kw | 8,214 |
| #30 | ("critical illness"):ti,ab,kw | 4,441 |
| #31 | ("critical care"):ti,ab,kw | 4,859 |
| #32 | (PICU):ti,ab,kw | 1,009 |
| #33 | (PCCU):ti,ab,kw | 4 |
| #34 | ("congenital heart disease"):ti,ab,kw | 1,701 |
| #35 | ("congenital heart surgery"):ti,ab,kw | 160 |
| #36 | #21 or #22 or #23 or #24 or #25 or #26 or #27 or #28 or #29 or #30 or #31 or #32 or #33 or #34 or #35 | 54,161 |
| #37 | MeSH descriptor: [Enteral Nutrition] explode all trees | 2,179 |
| #38 | MeSH descriptor: [Milk, Human] explode all trees | 1,277 |
| #39 | MeSH descriptor: [Food, Formulated] explode all trees | 1,583 |
| #40 | (enteral nutrition):ti,ab,kw | 5,603 |
| #41 | (enteral feed*):ti,ab,kw | 4,127 |
| #42 | (tube feed*):ti,ab,kw | 2,645 |
| #43 | (artificial feed*):ti,ab,kw | 1,499 |
| #44 | (artificial nutrition):ti,ab,kw | 980 |
| #45 | (force feed*):ti,ab,kw | 711 |
| #46 | (nutrition support):ti,ab,kw | 4,688 |
| #47 | (nutritional support):ti,ab,kw | 4,495 |
| #48 | (intragastric feed*):ti,ab,kw | 162 |
| #49 | (gastric feed*):ti,ab,kw | 1,721 |
| #50 | (gastric tube):ti,ab,kw | 2,092 |
| #51 | (nasogastric):ti,ab,kw | 2,573 |
| #52 | (nasoenteral):ti,ab,kw | 54 |
| #53 | (percutaneous tube):ti,ab,kw | 699 |
| #54 | (gastrostomy):ti,ab,kw | 764 |
| #55 | (gavage):ti,ab,kw | 371 |
| #56 | (gastrogavage):ti,ab,kw | 1 |
| #57 | (milk):ti,ab,kw | 12,926 |
| #58 | (food formulated):ti,ab,kw | 1,245 |
| #59 | (enteral diet):ti,ab,kw | 1,317 |
| #60 | (enteral formula):ti,ab,kw | 1,116 |
| #61 | #37 or #38 or #39 or #40 or #41 or #42 or #43 or #44 or #45 or #46 or #47 or #48 or #49 or #50 or #51 or #52 or #53 or #54 or #55 or #56 or #57 or #58 or #59 or #60 | 32,136 |
| #62 | (bolus):ti,ab,kw | 18,791 |
| #63 | (intermittent*):ti,ab,kw | 17,657 |
| #64 | #62 or #63 | 35,435 |
| #65 | (continuous*):ti,ab,kw | 72,829 |
| #66 | (continued):ti,ab,kw | 39,348 |
| #67 | (constant):ti,ab,kw | 15,198 |
| #68 | #65 or #66 or #67 | 122,745 |
| #69 | #20 and #36 and #61 and (#64 or #68) | 192 |

Search Database

■Igaku Chuo Zasshi

Search Strategy and Hits:

Igaku Chuo Zasshi search strategy （Search Date: April 21, 2023）

| #1 | (新生児/TH or 新生児/TA) and (PT=会議録除く) | 61228 |
| --- | --- | --- |
| #2 | (乳児/TH or 乳児/TA) and (PT=会議録除く) | 41265 |
| #3 | (幼児/TH or 幼児/TA) and (PT=会議録除く) | 29011 |
| #4 | (小児/TH or 小児/TA) and (PT=会議録除く) | 207930 |
| #5 | (思春期/TH or 思春期/TA) and (PT=会議録除く) | 16907 |
| #6 | (青年/TH or 青年/TA) and (PT=会議録除く) | 8538 |
| #7 | (小学生/TH or 小学/TA) and (PT=会議録除く) | 12588 |
| #8 | (中学生/TH or 中学/TA) and (PT=会議録除く) | 12412 |
| #9 | (高校生/TH or 高校/TA or 高等学校/TA) and (PT=会議録除く) | 9985 |
| #10 | (学童/TA) and (PT=会議録除く) | 6951 |
| #11 | #1 or #2 or #3 or #4 or #5 or #6 or #7 or #8 or #9 or #10 | 319301 |
| #12 | (極低出生体重児/TH) and (PT=会議録除く) | 2964 |
| #13 | (未熟児/TH) and (PT=会議録除く) | 4531 |
| #14 | #12 or #13 | 7261 |
| #15 | #11 not #14 | 312040 |
| #16 | (小児ICU/TH or 新生児ICU/TH or PICU/TA) and (PT=会議録除く) | 8343 |
| #17 | (PCCU/TA or 小児心臓ケアユニット/TA) and (PT=会議録除く) | 3 |
| #18 | (心臓疾患-先天性/TH or 先天性心疾患/TA) and (PT=会議録除く) | 33433 |
| #19 | (心肺バイパス術/TH or 心肺バイパス術/TA) and (PT=会議録除く) | 4743 |
| #20 | (胸部外科/TH or 胸部外科/TA) and (PT=会議録除く) | 83833 |
| #21 | (心臓外科/TH or 心臓外科/TA) and (PT=会議録除く) | 43885 |
| #22 | (先天性心臓手術/TA) and (PT=会議録除く) | 15 |
| #23 | (ICU/TH or “intensive care unit”/TA or ICU/TA) and (PT=会議録除く) | 55436 |
| #24 | （集中治療/TA) and (PT=会議録除く) | 12382 |
| #25 | (クリティカルケア/TH or クリティカルケア/TA or クリティカル・ケア/TA) and (PT=会議録除く) | 7487 |
| #26 | (重症患者/TA) and (PT=会議録除く) | 3899 |
| #27 | #16 or #17 or #18 or #19 or #20 or #21 or #22 or #23 or #24 or #25 or #26 | 178341 |
| #28 | (ランダム化比較試験/TH or 準ランダム化比較試験/TH or ランダム化/AL or 無作為化/AL or 比較試験/AL or 臨床試験/AL or プラセボ/AL or 対照/AL or コントロール/AL or 臨床研究/AL) | 349708 |
| #29 | (経腸栄養/TH) and (PT=会議録除く) | 12201 |
| #30 | ((経腸/TA or 経管/TA or 経胃/TA or 胃内/TA or 経鼻/TA or 鼻腔/TA or 強制/TA or 人工/TA or チューブ/TA) and 栄養/TA) and (PT=会議録除く) | 14347 |
| #31 | ((経腸/TA or 経管/TA or 経胃/TA or 胃内/TA or 経鼻/TA) and 投与/TA) and (PT=会議録除く) | 6725 |
| #32 | (フィーディング/TA and チューブ/TA) and (PT=会議録除く) | 30 |
| #33 | (胃管/TA) and (PT=会議録除く) | 4437 |
| #34 | (胃瘻/TH or 胃瘻/TA) and (PT=会議録除く) | 5033 |
| #35 | (胃造瘻術/TH or 胃造瘻術/TA or 胃瘻造設/TA) and (PT=会議録除く) | 6516 |
| #36 | (栄養/TA and (補給/TA or 療法/TA or 投与/TA)) and (PT=会議録除く) | 22978 |
| #37 | (EN/TA or "Enteral nutrition"/TA or "Enteral feed"/TA) and (PT=会議録除く) | 602483 |
| #38 | (母乳/TH or 母乳/TA) and (PT=会議録除く) | 9442 |
| #39 | (ミルク/TA or 人工乳/TA or 治療乳/TA) and (PT=会議録除く) | 2989 |
| #40 | (経腸栄養剤/TH or 経腸栄養剤/TA) and (PT=会議録除く) | 4190 |
| #41 | #29 or #30 or #31 or #32 or #33 or #34 or #35 or #36 or #37 or #38 or #39 or #40 | 653946 |
| #42 | (間欠/TA or 間歇/TA or ボーラス/TA or 断続/TA or 非持続/TA) and (PT=会議録除く) | 16562 |
| #43 | (持続/TA or 連続/TA or 非間欠/TA) and (PT=会議録除く) | 127419 |
| #44 | #15 and #27 and #28 and #41 and (#42 or #43) | 29 |

Flow diagram

Risk of bias

Length of ICU stay

Duration of mechanical ventilation

Ventilator-associated infections

Emesis

Diarrhea

Gastric residual volume

Time to target feeding goal

Funnel plot & Forrest plot

Length of ICU stay

Duration of mechanical ventilation

Ventilator-associated infections

Emesis

Diarrhea

Gastric residual volume

Time needed to target feeding goal

**CQ4-8: Should energy/protein-dense formulas be administered to critically ill pediatric patients who are receiving enteral nutrition?**

Search Database

- MEDLINE (PubMed/Ovid)

Search Strategy and Hits:

PubMed search strategy （Search Date: April 25, 2023）

| 1 | ("child"[MeSH Terms] OR "adolescent"[MeSH Terms] OR "adolescent, hospitalized"[MeSH Terms] OR "infant"[MeSH Terms] OR "child, hospitalized"[MeSH Terms] OR "neonate"[Title/Abstract] OR "newborn"[Title/Abstract] OR "infant*"[Title/Abstract] OR "toddler"[Title/Abstract] OR "child*"[Title/Abstract] OR "adolescent*"[Title/Abstract] OR paediatric*[Title/Abstract] OR pediatric*[Title/Abstract] OR "pediatrics"[MeSH Terms]) NOT ("infant, premature"[MeSH Terms] OR "infant, very low birth weight"[MeSH Terms]) | 4,508,645 |
| --- | --- | --- |
| 2 | "intensive care units"[mh] OR "intensive care"[tiab] OR "critical illness"[mh] OR "critical care"[mh] OR "critically ill"[tiab] OR "critical care"[tiab] OR PICU[tiab] OR PCCU[tiab] OR "heart defects, congenital"[mh] OR "cardiopulmonary bypass"[mh] OR "thoracic surgery"[mh] OR "cardiac surgical procedures"[mh] OR "congenital heart disease"[tiab] OR "congenital heart surgery"[tiab] | 713,092 |
| 3 | ("randomized controlled trial"[Publication Type] OR "controlled clinical trial"[Publication Type] OR randomized[tiab] OR placebo[tiab] OR "drug therapy"[MeSH Subheading] OR randomly[tiab] OR trial[tiab] OR groups[tiab]) NOT (animals[mh] NOT humans[mh]) | 4,992,614 |
| 4 | "infant formula"[mh] OR "milk, human"[mh] OR "enteral nutrition"[mh] OR "food, formulated"[mh] OR nutrients[mh] OR "milk substitutes"[mh] OR "nutritional status"[mh] OR "infant nutritional physiological phenomena"[mh] OR formula*[tiab] OR milk[tiab] OR nutri* [tiab] OR feed*[tiab] OR nasogastric[tiab] OR nasoenteral[tiab] | 1,546,570 |
| 5 | concentrat*[tiab] OR dens*[tiab] OR enrich*[tiab] OR energy[tiab] OR high*[tiab] | 10,943,344 |
| 6 | #1 AND #2 AND #3 AND #4 AND #5 | 1,112 |

Search Database

- CENTRAL

Search Strategy and Hits:

CENTRAL search strategy （Search Date: April 19, 2023）

| #1 | MeSH descriptor: [Child] explode all trees | 77476 |
| --- | --- | --- |
| #2 | MeSH descriptor: [Adolescent] explode all trees | 125165 |
| #3 | MeSH descriptor: [Adolescent, Hospitalized] explode all trees | 9 |
| #4 | MeSH descriptor: [Infant] explode all trees | 41440 |
| #5 | MeSH descriptor: [Child, Hospitalized] explode all trees | 172 |
| #6 | MeSH descriptor: [Pediatrics] explode all trees | 1178 |
| #7 | (neonate):ti,ab,kw | 2245 |
| #8 | (newborn):ti,ab,kw | 31652 |
| #9 | (infant):ti,ab,kw | 59253 |
| #10 | (toddler):ti,ab,kw | 1095 |
| #11 | (child):ti,ab,kw | 174132 |
| #12 | (adolescent):ti,ab,kw | 147831 |
| #13 | (paediatric):ti,ab,kw | 38857 |
| #14 | (pediatric):ti,ab,kw | 38865 |
| #15 | (pediatrics):ti,ab,kw | 4830 |
| #16 | #1 OR #2 OR #3 OR #4 OR #5 OR #6 OR #7 OR #8 OR #9 OR #10 OR #11 OR #12 OR #13 OR #14 OR #15 | 310024 |
| #17 | MeSH descriptor: [Infant, Premature] explode all trees | 4840 |
| #18 | MeSH descriptor: [Infant, Very Low Birth Weight] explode all trees | 1171 |
| #19 | #17 OR #18 | 5343 |
| #20 | #16 NOT #19 | 304681 |
| #21 | MeSH descriptor: [Intensive Care Units] explode all trees | 5225 |
| #22 | MeSH descriptor: [Critical Illness] explode all trees | 3218 |
| #23 | MeSH descriptor: [Critical Care] explode all trees | 2650 |
| #24 | MeSH descriptor: [Heart Defects, Congenital] explode all trees | 2833 |
| #25 | MeSH descriptor: [Cardiopulmonary Bypass] explode all trees | 3314 |
| #26 | MeSH descriptor: [Thoracic Surgery] explode all trees | 198 |
| #27 | MeSH descriptor: [Cardiac Surgical Procedures] explode all trees | 15455 |
| #28 | ("intensive care"):ti,ab,kw | 28987 |
| #29 | ("critically ill"):ti,ab,kw | 8214 |
| #30 | (“critical illness”):ti,ab,kw | 4441 |
| #31 | ("critical care"):ti,ab,kw | 4859 |
| #32 | (PICU):ti,ab,kw | 1009 |
| #33 | (PCCU):ti,ab,kw | 4 |
| #34 | ("congenital heart disease"):ti,ab,kw | 1701 |
| #35 | ("congenital heart surgery"):ti,ab,kw | 160 |
| #36 | #21 OR #22 OR #23 OR #24 OR #25 OR #26 OR #27 OR #28 OR #29 OR #30 OR #31 OR #32 OR #33 OR #34 OR #35 | 54161 |
| #37 | #20 AND #36 | 11300 |
| #38 | MeSH descriptor: [Infant Formula] explode all trees | 765 |
| #39 | MeSH descriptor: [Milk, Human] explode all trees | 1277 |
| #40 | MeSH descriptor: [Enteral Nutrition] explode all trees | 2179 |
| #41 | MeSH descriptor: [Food, Formulated] explode all trees | 1583 |
| #42 | MeSH descriptor: [Nutrients] explode all trees | 6298 |
| #43 | MeSH descriptor: [Milk Substitutes] explode all trees | 867 |
| #44 | MeSH descriptor: [Nutritional Status] explode all trees | 3569 |
| #45 | MeSH descriptor: [Infant Nutritional Physiological Phenomena] explode all trees | 4001 |
| #46 | MeSH descriptor: [Nutritional Support] explode all trees | 4156 |
| #47 | (formula*):ti,ab,kw | 52037 |
| #48 | (milk):ti,ab,kw | 12929 |
| #49 | (nutri*):ti,ab,kw | 57043 |
| #50 | (feed*):ti,ab,kw | 51360 |
| #51 | (nasogastric):ti,ab,kw | 2573 |
| #52 | (nasoenteral):ti,ab,kw | 54 |
| #53 | #38 OR #39 OR #40 OR #41 OR #42 OR #43 OR #44 OR #45 OR #46 OR #47 OR #48 OR #49 OR #50 OR #51 OR #52 | 154509 |
| #54 | (concentrat*):ti,ab,kw | 160105 |
| #55 | (dens*):ti,ab,kw | 50066 |
| #56 | (enrich*):ti,ab,kw | 9649 |
| #57 | (energy):ti,ab,kw | 40994 |
| #58 | (high*):ti,ab,kw | 553172 |
| #59 | #54 OR #55 OR #56 OR #57 OR #58 | 687066 |
| #60 | #53 AND #59 | 77747 |
| #61 | #37 AND #60 | 905 |

Search Database

■Igaku Chuo Zasshi

Search Strategy and Hits:

Igaku Chuo Zasshi search strategy （Search Date: April 19, 2023）

| #1 | ((新生児/TH or 新生児/TA)) and (PT=会議録除く) | 61228 |
| --- | --- | --- |
| #2 | ((乳児/TH or 乳児/TA)) and (PT=会議録除く) | 41265 |
| #3 | ((幼児/TH or 幼児/TA)) and (PT=会議録除く) | 29011 |
| #4 | ((小児/TH or 小児/TA)) and (PT=会議録除く) | 207930 |
| #5 | ((思春期/TH or 思春期/TA)) and (PT=会議録除く) | 16907 |
| #6 | ((青年/TH or 青年/TA)) and (PT=会議録除く) | 8538 |
| #7 | ((小学生/TH or 小学/TA)) and (PT=会議録除く) | 12588 |
| #8 | ((中学生/TH or 中学/TA)) and (PT=会議録除く) | 12412 |
| #9 | ((高校生/TH or 高校/TA or 高等学校/TA)) and (PT=会議録除く) | 9985 |
| #10 | (学童/TA) and (PT=会議録除く) | 6951 |
| #11 | #1 or #2 or #3 or #4 or #5 or #6 or #7 or #8 or #9 or #10 | 319301 |
| #12 | (極低出生体重児/TH) and (PT=会議録除く) | 2964 |
| #13 | (未熟児/TH) and (PT=会議録除く) | 4531 |
| #14 | #12 or #13 | 7261 |
| #15 | #11 not #14 | 312040 |
| #16 | (ICU/TH or “intensive care unit”/TA or ICU/TA) and (PT=会議録除く) | 55436 |
| #17 | (クリティカルケア/TH or クリティカルケア/TA or クリティカル・ケア/TA or 集中治療/TA) and (PT=会議録除く) | 16525 |
| #18 | (重症患者/TA) and (PT=会議録除く) | 3899 |
| #19 | (小児ICU/TH or 小児ICU/TA or PICU/TA) and (PT=会議録除く) | 8404 |
| #20 | (新生児ICU/TH or 新生児ICU/TA) and (PT=会議録除く) | 7114 |
| #21 | ((PCCU/TA or 小児心臓ケアユニット/TA)) and (PT=会議録除く) | 3 |
| #22 | ((心臓疾患-先天性/TH or 先天性心疾患/TA)) and (PT=会議録除く) | 33433 |
| #23 | ((心肺バイパス術/TH or 心肺バイパス術/TA)) and (PT=会議録除く) | 4743 |
| #24 | ((胸部外科/TH or 胸部外科/TA)) and (PT=会議録除く) | 83833 |
| #25 | ((心臓外科/TH or 心臓外科/TA)) and (PT=会議録除く) | 43885 |
| #26 | (先天性心臓手術/TA) and (PT=会議録除く) | 15 |
| #27 | #16 or #17 or #18 or #19 or #20 or #21 or #22 or #23 or #24 or #25 or #26 | 178341 |
| #28 | #15 and #27 | 18650 |
| #29 | ランダム化比較試験/TH or 準ランダム化比較試験/TH or ランダム化/AL or 無作為化/AL or 比較試験/AL or 臨床試験/AL orプラセボ/AL or 対照/AL or コントロール/AL or 臨床研究/AL | 223559 |
| #30 | #28 and #29 | 551 |
| #31 | ((乳児用調整乳/TH or 乳児用調製粉乳/TA or 調製乳/AL or 粉乳/AL)) and (PT=会議録除く) | 1925 |
| #32 | ((母乳/TH or 母乳/TA)) and (PT=会議録除く) | 9442 |
| #33 | ((経腸栄養/TH or 経管栄養/TA or 経腸栄養/TA)) and (PT=会議録除く) | 16142 |
| #34 | (フォーミュラー食品/TH) and (PT=会議録除く) | 4765 |
| #35 | ((哺乳びん栄養補給/TH or 人工栄養/TA or 人工乳/TA)) and (PT=会議録除く) | 2406 |
| #36 | ((乳/TH or ミルク/TA)) and (PT=会議録除く) | 8106 |
| #37 | ((代用乳/TH or 代用乳/TA)) and (PT=会議録除く) | 1399 |
| #38 | ((母乳栄養/TH or 哺乳/TA)) and (PT=会議録除く) | 18444 |
| #39 | (乳児栄養生理学的現象/TH) and (PT=会議録除く) | 9713 |
| #40 | (栄養素/TH ) and (PT=会議録除く) | 64192 |
| #41 | (栄養補助/TH) and (PT=会議録除く) | 22524 |
| #42 | (栄養状態/TH) and (PT=会議録除く) | 3231 |
| #43 | (栄養/TA) and (PT=会議録除く) | 95117 |
| #44 | ((胃ゾンデ/TH or 胃管/TA)) and (PT=会議録除く) | 5271 |
| #45 | #31 or #32 or #33 or #34 or #35 or #36 or #37 or #38 or #39 or #40 or #41 or #42 or #43 or #44 | 175284 |
| #46 | ((濃度/TH or 濃度/TA)) and (PT=会議録除く) | 132,604 |
| #47 | ((濃縮/TH or 濃縮/TA)) and (PT=会議録除く) | 5,756 |
| #48 | ((密度/TH or 密度/TA)) and (PT=会議録除く) | 23483 |
| #49 | (高/TA) and (PT=会議録除く) | 862,924 |
| #50 | ((高カロリー/TA)) and (PT=会議録除く) | 3372 |
| #51 | ((高エネルギー/TA)) and (PT=会議録除く) | 1634 |
| #52 | #46 or #47 or #48 or #49 or #50 or #51 | 950149 |
| #53 | #45 and #52 | 48752 |
| #54 | #30 and #53 | 22 |

Flow diagram

Risk of bias

Mortality

Length of ICU stay

Duration of mechanical ventilation

Emesis

Diarrhea

Gastrointestinal bleeding

Weight for age Z score

Forest plot & Funnel plot

Mortality

Length of ICU stay

Duration of mechanical ventilation

Emesis

Diarrhea

Gastrointestinal bleeding

Weight for age Z score

Sensitivity analysis

Mortality

Length of ICU stay

Duration of mechanical ventilation

Emesis

Diarrhea

Gastrointestinal bleeding

Weight for age Z score

| **Committee for the Japanese Critical Care Nutrition Guideline 2024 -Members’ names, conflicts of interest (COI), and roles-** | | | | | | | | | | | | | | | | | | | |
| --- | --- | --- | --- | --- | --- | --- | --- | --- | --- | --- | --- | --- | --- | --- | --- | --- | --- | --- | --- |
| **Panelists, Organizers** | |  |  |  |  |  |  |  |  |  |  |  |  |  |  |  |  |  |  |
| Name | Workplace | Financial COI | | | | | | | | | | | | | | Academic COI | | Position | Area of GL (◎ leader) |
|  |  | A-1 | A-2 | A-3 | A-4 | A-5 | A-6 | A-7 | A-8 | A-9 | B-1 | B-2 | B-3 | C-1 | C-2 | Position in academic societies | Contribution to other guidelines |  |  |
| Joji Kotani | Division of Disaster and Emergency Medicine, Department of Surgery Related, Kobe University Graduate School of Medicine, Hyogo, Japan | no | no | no | no | no | no | no | no | No | no | no | no | no | no | JSCNM (Director), Japanese society for surgical metabolism and nutrition (Director), Cytokine research in surgery and critical care (Chief Organizer), Japan endotoxin and innate immunity society (Chief Director), Japan society for critical care medicine (Director), International Association for Surgical Nutrition and Metabolism (Secretary General), Asian Association for Surgical Metabolism and Nutrition (Organizing Committee) | Clinical guidelines for the nutrition therapy of patients with cancer | Chairman |  |
| Kensuke Nakamura | Department of Critical Care Medicine, Yokohama City University Hospital, Kanagawa, Japan | no | no | no | no | no | no | no | no | No | no | no | no | no | no | Japanese society for early mobilization (Director), Japan society for homecare and emergency medicine (Director) | J-ReCIP2023 J-SSCG2020 | Vice-chairman |  |
| Naoki Higashibeppu | Department of Anesthesia and Critical Care, Kobe City Medical Center General Hospital, Kobe, Japan | no | no | no | no | no | no | no | no | No | no | no | no | no | no | No | Clinical guidelines for the nutrition therapy of patients with cancer, J-SSCG2016/2020, JCCNG2016/2017 | committee member | ◎WG1 |
| Hiroo Izumino | Acute and Critical Care Center, Nagasaki University Hospital, Nagasaki, Japan | no | no | no | Nestlé Japan Ltd. | no | no | no | no | No | no | no | no | no | no | No | no | committee member |  |
| Taku Oshima | Department of Emergency and Critical Care Medicine, Chiba University Graduate School of Medicine, Chiba City, Japan | no | no | no | no | no | no | no | no | No | no | no | no | no | Nihon Kohden | No | no | committee member |  |
| Yoshiyuki Shimizu | Department of Intensive Care Medicine, Osaka Women’s and Children’s Hospital, Osaka, Japan | no | no | no | no | no | no | no | no | No | no | no | no | no | no | No | JCCNG2016/2017 | committee member | ◎WG4 |
| Hiroomi Tatsumi | Department of Intensive Care Medicine, Sapporo Medical University School of Medicine, Sapporo, Japan | no | no | no | Otsuka Pharmaceutical Factory, Inc. | no | Otsuka Pharmaceutical Factory, Inc. | no | no | No | no | no | no | no | no | No | J-SSCG2020 | committee member | ◎WG3 |
| Junji Hatakeyama | Department of Emergency and Critical Care Medicine, Osaka Medical and Pharmaceutical University, Osaka, Japan | no | no | no | no | no | no | no | no | No | no | no | no | no | no | No | J-SSCG2024 | committee member |  |
| Minoru Yoshida | Department of Emergency and Critical Care Medicine, St. Marianna University School of Medicine, Kanagawa, Japan | no | no | no | no | no | no | no | no | no | no | no | no | Eisai Co., Ltd. | no | No | no | committee member | ◎WG2 |
| Akira Ouchi | Department of Adult Health Nursing, College of Nursing, Ibaraki Christian University, Hitachi, Japan | no | no | no | no | no | no | no | no | no | no | no | no | no | no | No | J-ReCIP2023 J-SSCG2024 | committee member | WG |
| Rie Tsutsumi | Department of Anesthesiology and Critical Care, Hiroshima University Hospital, Hiroshima, Japan | no | Citlian, Inc. | no | Nippon Television Holdings, Inc. | no | Nestlé Japan Ltd. | IKEDA YAKUSOU | no | no | no | no | no | no | no | JSPEN(Director), Japanese society for child nutrition research (Director) | Clinical guidelines for the nutrition therapy of patients with cancer | committee member |  |
| Norihiko Tsuboi | Department of Critical Care Medicine and Anesthesia, National Center for Child Health and Development, Tokyo, Japan | no | no | no | no | no | no | no | no | no | no | no | no | no | no | No | no | committee member |  |
| Natsuhiro Yamamoto | Department of Anesthesiology and Critical Care Medicine, Yokohama City University School of Medicine, Kanagawa, Japan | no | no | no | no | no | no | no | no | no | no | no | no | no | no | No | J-ReCIP2023 | committee member | WG |
| Ayumu Nozaki | Department of Pharmacy, Kyoto-Katsura Hospital, Kyoto, Japan | no | no | no | no | no | no | no | no | no | no | no | no | no | no | No | ARDS Clinical Practice Guideline2016 | committee member |  |
| Muneyuki Takeuchi | Department of Critical Care Medicine, National Cerebral and Cardiovascular Center, Osaka, Japan | no | no | no | no | no | no | no | no | no | no | no | no | no | no | JSICM(Director), JSPICC (Chief Director) | ARDS Clinical Practice Guideline | Director in charge |  |
|  |  |  |  |  |  |  |  |  |  |  |  |  |  |  |  |  |  |  |  |
| **Academic Guidelines Promotion Team members** | | | |  |  |  |  |  |  |  |  |  |  |  |  |  |  |  |  |
| Name | Workplace | Financial COI | | | | | | | | | | | | | | Academic COI | | Position (◎ leader) | Area of GL |
|  |  | A-1 | A-2 | A-3 | A-4 | A-5 | A-6 | A-7 | A-8 | A-9 | B-1 | B-2 | B-3 | C-1 | C-2 | Position in academic societies | Contribution to other guidelines |  |  |
| Ryo Yamamoto | Department of Emergency and Critical Care Medicine, Keio University School of Medicine, Tokyo, Japan | no | no | no | no | no | no | no | no | no | no | no | no | no | no | No | J-SSCG2024 | ◎AC |  |
| Sadaharu Asami | Department of Cardiology, Musashino Tokushukai Hospital, Tokyo, Japan | no | no | no | no | no | no | no | no | no | no | no | no | no | no | No | no | AC |  |
| Yudai Takatani | Department of Primary Care and Emergency Medicine, Kyoto University Hospital, Kyoto, Japan. | no | no | no | no | no | no | no | no | no | no | no | no | no | no | No | DIC Clinical Practice Guideline2024 | AC |  |
| Kohei Yamada | Department of Traumatology and Critical Care, National Defense Medical College Hospital, Saitama, Japan | no | no | no | no | no | no | no | no | no | no | no | no | no | no | No | J-SSCG2020/2024, ARDS Clinical Practice Guideline2021, Neurointensive care Clinical Practice Guideline2024 | AC |  |
| Yujiro Matsuishi | Adult and elderly nursing, Faculty of Nursing, Tokyo University of Information Science, Chiba, Japan | no | no | no | no | no | no | no | no | no | no | no | no | no | no | No | J-ReCIP2023 | AC |  |
|  |  |  |  |  |  |  |  |  |  |  |  |  |  |  |  |  |  |  |  |
|  |  |  |  |  |  |  |  |  |  |  |  |  |  |  |  |  |  |  |  |
| **Working group members** | |  |  |  |  |  |  |  |  |  |  |  |  |  |  |  |  |  |  |
| Name | Workplace | Financial COI | | | | | | | | | | | | | | Academic COI | | Position | Area of GL |
|  |  | A-1 | A-2 | A-3 | A-4 | A-5 | A-6 | A-7 | A-8 | A-9 | B-1 | B-2 | B-3 | C-1 | C-2 | Position in academic societies | Contribution to other guidelines |  |  |
| Shuhei Takauji | Department of Emergency Medicine, Hokkaido University Hospital, Sapporo, Japan | no | no | no | no | no | no | no | no | no | no | no | no | no | no | No | no | WG | WG1 |
| Akihito Tampo | Department of Emergency Medicine, Asahikawa Medical University, Asahikawa, Japan | no | no | no | no | no | no | no | no | no | no | no | no | no | no | No | J-SSCG2020/2024, DIC Clinical Practice Guideline2024 | WG | WG2 |
| Yusuke Terasaka | Department of Emergency Medicine, Kyoto Katsura Hospital, Kyoto, Japan | no | no | no | Nippon BD Co., Ltd. | no | no | no | no | no | no | no | no | no | no | No | JRC Resuscitation Guidelines2020 | WG | WG1 |
| Takeaki Sato | Tohoku University Hospital Emergency Center, Miyagi, Japan | no | no | no | no | no | no | no | no | no | no | no | no | no | no | No | no | WG | WG3 |
| Saiko Okamoto | Department of Nursing, Hitachi General Hospital, Hitachi, Japan | no | no | no | no | no | no | no | no | no | no | no | no | no | no | No | J-ReCIP2023 | WG | WG1 |
| Hideaki Sakuramoto | Department of Acute Care Nursing, Japanese Red Cross Kyushu International College of Nursing, Munakata, Japan | no | no | no | no | no | no | no | NEW NURSING Inc | no | no | no | no | no | no | No | J-ReCIP2023 | WG | WG2 |
| Tomoka Miyagi | Anesthesiology and Critical Care Medicine, Master's Degree Program, Graduate School of Medicine, Yokohama City University, Kanagawa, Japan | no | no | no | no | no | no | no | no | no | no | no | no | no | no | No | no | WG | WG3 |
| Keisei Aki | Department of Pharmacy, Kokura memorial Hospital, Fukuoka, Japan | no | no | no | no | no | no | no | no | no | no | no | no | no | no | No | no | WG | WG3 |
| Hidehito Ota | Department of Pediatrics, School of Medicine, The University of Tokyo, Tokyo, Japan | no | no | no | no | no | no | no | no | no | no | no | no | no | no | No | no | WG | WG4 |
| Taro Watanabe | Department of Intensive Care Medicine, Showa University School of Medicine, Tokyo, Japan | no | no | no | no | no | no | no | no | no | no | no | no | no | no | No | no | WG | WG4 |
| Nobuto Nakanishi | Department of Disaster and Emergency Medicine, Graduate School of Medicine, Kobe University, Kobe, Japan | no | no | no | no | no | no | no | no | no | no | no | no | no | no | No | J-ReCIP2023 J-SSCG2024 | WG | WG2 |
| Hiroyuki Ohbe | Department of Emergency and Critical Care Medicine, Tohoku University Hospital, Sendai, Japan | no | no | no | no | no | no | no | no | no | no | no | no | no | no | No | no | WG | WG2 |
| Chihiro　Narita | Department of Emergency Medicine, Shizuoka General Hospital, Shizuoka, Japan | no | no | no | no | no | no | no | no | no | no | no | no | no | no | No | J-ReCIP2023 ARDS Clinical Practice Guideline2026, J-SSCG2020 | WG | WG2 |
| Jun Takeshita | Department of Anesthesiology, Osaka Women's and Children's Hospital, Izumi, Japan | no | no | no | no | no | no | no | no | no | no | no | no | no | no | No | J-SSCG2020/2024 ARDS Clinical Practice Guideline2021 | WG | WG4 |
| Masano Sagawa | Department of Surgery, Tokyo Women’s Medical University Adachi Medical Center, Tokyo, Japan | no | no | no | no | no | no | no | no | no | no | no | no | no | no | No | Clinical guidelines for the nutrition therapy of patients with cancer, Care Guidelines for the Collaboration of Rehabilitation, Nutrition, and Oral Care in the Chronic Phase | WG | WG1 |
| Takefumi Tsunemitsu | Department of Preventive Services, Graduate School of Medicine, Kyoto University, Kyoto, Japan | no | no | no | no | no | no | no | no | no | no | no | no | HealthTech.Lab Inc. | Ritsuan /ZOLL Medical | No | J-SSCG2024 | WG | WG1 |
| Shinya Matsushima | Department of Physical Therapy, Faculty of Health Science, Kyorin University, Tokyo, Japan | no | no | no | no | no | no | no | no | no | no | no | no | no | no | No | J-ReCIP2023 | WG | WG1 |
| Yorihide Yanagita | Department of Health Sciences, Institute of Biomedical Sciences, Nagasaki University, Nagasaki, Japan | no | no | no | no | no | no | no | no | no | no | no | no | no | no | No | The clinical practice guidelines on tracheal suctioning 2023 （3rd edition） | WG | WG1 |
| Shinichi Watanabe | Department of Physical Therapy, Faculty of Rehabilitation, Gifu University of Health Science, 2-92 Higashiuzura, Gifu, 500-8281, Japan. | no | no | no | no | no | no | no | no | no | no | no | no | no | no | No | J-SSCG J-ReCIP2023 | SR WG | WG3 |
| Hiroyasu Murata | Department of Rehabilitation Medicine, Kyorin University Hospital, Tokyo, Japan | no | no | no | no | no | no | no | no | no | no | no | no | no | no | No | no | SR WG | WG2 |
| Akihisa Taguchi | Department of Anesthesia, Kyoto University Hospital, Kyoto, Japan | no | no | no | no | no | no | no | no | no | no | no | no | no | no | No | no | SR WG | WG1 |
| Takuya Hiramoto | Department of Internal Medicine, Tokyo Bay Urayasu Ichikawa Medical Center, Urayasu, Japan | no | no | no | no | no | no | no | no | no | no | no | no | no | no | No | no | SR WG | WG4 |
| Daisuke Kobashi | Department of Critical Care and Emergency Medicine, Japanese Red Cross Maebashi Hospital, Gunma, Japan | no | no | no | no | no | no | no | no | no | no | no | no | no | no | No | no | WG | WG1 |
|  |  |  |  |  |  |  |  |  |  |  |  |  |  |  |  |  |  |  |  |
| **Systematic review members** | |  |  |  |  |  |  |  |  |  |  |  |  |  |  |  |  |  |  |
| Name | Workplace | Financial COI | | | | | | | | | | | | | | Academic COI | | Position | Area of GL |
|  |  | A-1 | A-2 | A-3 | A-4 | A-5 | A-6 | A-7 | A-8 | A-9 | B-1 | B-2 | B-3 | C-1 | C-2 | Position in academic societies | Contribution to other guidelines |  |  |
| Atsuhiro Ijiri | Department of Traumatology and Critical Care Medicine, National Defense Medical College, Saitama, Japan | no | no | no | no | no | no | no | no | no | no | no | no | no | no | No | J-SSCG2024 | SR | WG1 |
| Ken-ichi Kano | Department of Pharmacoepidemiology, Graduate School of Medicine, Kyoto University, Kyoto, Japan | no | no | no | no | no | no | no | no | no | no | no | no | no | no | No | Heat Stroke Guideline2024, Practice Guidelines for Primary Care of Acute Abdomen2025, J-SSCG2024, Neurointensive care Clinical Practice Guideline2024 | SR | WG1 |
| Satoshi Nakano | Department of Pediatric Emergency and Critical Care, Saitama Children’s Medical Center, Saitama, Japan | no | no | no | no | no | no | no | no | no | no | no | no | no | no | No | no | SR | WG4 |
| Hitoshi Kikuchi | Department of Emergency and Critical Care Medicine, Fujisawa City Hospital. Kanagawa, Japan | no | no | no | no | no | no | no | no | no | no | no | no | no | no | No | J-SSCG2024 | SR | WG4 |
| Mahoko Taito | Department of Nursing, Hiroshima University Hospital, Hiroshima, Japan | no | no | no | no | no | no | no | no | no | no | no | no | no | no | No | J-SSCG2024 | SR | WG1 |
| Takaaki Maruhashi | Department of Emergency and Critical Care Medicine, Kitasato University School of Medicine, Sagamihara, Japan | no | no | no | no | no | no | no | no | no | no | no | no | no | no | No | ARDS Clinical Practice Guideline2021 JRC Resuscitation Guidelines2020 | SR | WG1 |
| Kenzo Ishii | Department of Anesthesiology, Intensive Care Unit, Fukuyama City Hospital, Fukuyama, Hiroshima, Japan | no | no | no | no | no | no | no | no | no | no | no | no | no | no | No | ARDS Clinical Practice Guideline2021 JRC Resuscitation Guidelines2025 | SR | WG1 |
| Junko Tatsuno | Department of Nursing, Kokura Memorial Hospital, Fukuoka, Japan | no | no | no | no | no | no | no | no | no | no | no | no | no | no | No | J-ReCIP2023 | SR | WG2 |
| Hiroshi Sugimoto | Department of Internal Medicine, NHO Kinki-Chuo Chest Medical Center, Osaka, Japan | no | no | no | no | no | no | no | no | no | no | no | no | no | no | No | ARDS Clinical Practice Guideline2021, The JRS Guidelines for the Management of Pneumonia in Adults2024, J-SSCG2024 | SR | WG1 |
| Hiromichi Naito | Department of Emergency and Critical Care Medicine, Okayama University Hospital, Okayama, Japan | no | no | no | no | no | no | no | no | no | no | no | no | no | no | No | J-SSCG2020 JATEC | SR | WG1 |
| Minoru Hayashi | Department of Emergency Medicine, Fukui Prefectural Hospital, Fukui, Japan | no | no | no | no | no | no | no | no | no | no | no | no | no | no | No | no | SR | WG1 |
| Kota Yamauchi | Department of Rehabilitation, Steel Memorial Yawata Hospital, Kitakyushu, Japan | no | no | no | no | no | no | no | no | no | no | no | no | no | no | No | no | SR | WG2 |
| Yoji Kokura | Department of Nutritional Management, Keiju Hatogaoka Integrated Facility for Medical and Long-Term Care, Anamizu, Japan | no | no | no | no | no | no | no | no | no | no | no | no | no | no | No | no | SR | WG2 |
| Takahiro Suzuki | Department of Cardiovascular Medicine, St. Luke’s International Hospital, Tokyo, Japan | no | no | no | no | no | no | no | no | no | no | no | no | no | no | No | Guideline on Cardiovascular Practice with Consideration for Diversity, Equity, and Inclusion | SR | WG3 |
| Katsuaki Honda | Department of Pharmacy, Seirei Hamamatsu General Hospital, Hamamatsu, Japan | no | no | no | no | no | no | no | no | no | no | no | no | no | no | No | Japanese Preterm PDA guideline Clinical Practice Guidelines for Prevention and Treatment of Chronic Lung Disease of Preterm Infants, Neonatal pain management care guideline in Japanese NICUs, ARDS Clinical Practice Guideline2026 | SR | WG4 |
| Maki Murata | Department of Emergency Medicine and Critical Care, NHO Kyoto Medical Center | no | no | no | no | no | no | no | no | no | no | no | no | no | no | No | ARDS Clinical Practice Guideline2021 | SR | WG2 |
| Kazuya Okada | Department of Critical Care Medicine, Tokyo Metropolitan Bokutoh Hospital, Tokyo, Japan | no | no | no | no | no | no | no | no | no | no | no | no | no | no | No | ARDS Clinical Practice Guideline2021 | SR | WG2 |
| Kazuki Kikuyama | Department of Intensive Care Medicine, Showa University Hospital, Tokyo, Japan | no | no | no | no | no | no | no | no | no | no | no | no | no | no | No | ARDS Clinical Practice Guideline2021 | SR | WG4 |
| Yoshihisa Fujinami | Department of Emergency Medicine, Kakogawa Central City Hospital, Hyogo, Japan | no | no | no | no | no | no | no | no | no | no | no | no | no | no | No | J-SSCG2024 | SR | WG2 |
| Yoshihiro Suido | Department of Infectious diseases, Shonan Kamakura General Hospital, Kanagawa, Japan | no | no | no | no | no | no | no | no | no | no | no | no | no | no | No | ARDS Clinical Practice Guideline2021 | SR | WG2 |
| Miki Sato | Division of Pharmacotherapeutics, Department of Clinical Pharmacy, School of Pharmacy, Showa University, Tokyo, Japan | no | no | no | no | no | no | no | no | no | no | no | no | no | no | No | no | SR | WG4 |
| Takumi Nagao | Department of Nursing, Sakakibara Heart Institute, Sakakibara Heart Foundation, Tokyo, Japan | no | no | no | no | no | no | no | no | no | no | no | no | no | no | No | ARDS Clinical Practice Guideline2021 | SR | WG4 |
| Yoshinobu Abe | Division of Emergency and Disaster Medicine, Tohoku Medical and Pharmaceutical University, Sendai, Japan | no | no | no | no | no | no | no | no | no | no | no | no | no | no | No | ARDS Clinical Practice Guideline2021 | SR | WG2 |
| Keiichiro Shimoyama | Department of Neurointensive Care and Coma Science Center, Toda Medical Group, Asaka Medical Center, Saitama, Japan | no | no | no | no | no | no | no | no | no | no | no | no | no | no | No | no | SR | WG4 |
| Taiki Yamamoto | Department of Intensive Care Nursing, Naniwa Ikuno Hospital, Osaka, Japan | no | no | no | no | no | no | no | no | no | no | no | no | no | no | No | no | SR | WG4 |
| Yoshihiro Nishita | Department of Pharmacy, Kanazawa Medical University Hospital, Ishikawa, Japan | no | no | no | no | no | no | no | no | no | no | no | no | no | no | No | no | SR | WG1 |
| Misako Mori | Department of Nursing, St. Marianna University School of Medicine, Kanagawa, Japan | no | no | no | no | no | no | no | no | no | no | no | no | no | no | No | no | SR | WG1 |
| Satomi Ichimaru | Food and Nutrition Service Department, Fujita Health University Hospital, Toyoake, Japan | no | no | no | no | no | no | no | no | no | no | no | no | no | no | No | no | SR | WG2 |
| Yasutaka Hamai | Department of Preventive Services, Graduate School of Medicine, Kyoto University, Kyoto, Japan | no | no | no | no | no | no | no | no | no | no | no | no | no | no | No | J-SSCG2024 | SR | WG1 |
| Hidenori Kasuya | Department of Nursing, Daido Hospital Kojunkai, Social Medical Corporation, Aichi, Japan | no | no | no | no | no | no | no | no | no | no | no | no | no | no | No | no | SR | WG1 |
| Fumiaki Ishikawa | Department of Nutrition Japanese Red Cross Medical Center, Tokyo, Japan | no | no | no | no | no | no | no | no | no | no | no | no | no | no | No | no | SR | WG1 |
| Aya Tampo | Department of Anesthesia, Asahikawa City Hospital, Asahikawa, Japan | no | no | no | no | no | no | no | no | no | no | no | no | no | no | No | no | SR | WG4 |
| Kazuki Nagatomo | Department of Emergency and Critical Care Medicine, University of Tsukuba Hospital, Ibaraki, Japan | no | no | no | no | no | no | no | no | no | no | no | no | no | no | No | no | SR | WG2 |
| Kanako Takahashi | Department of Nephrology, Sapporo Hokushin Hospital, Sapporo, Japan | no | no | no | no | no | no | no | no | no | no | no | no | no | no | No | no | SR | WG2 |
| Noritomo Fujisaki | Department of Emergency Medicine, Hiroshima City Hiroshima Citizens Hospital, Hiroshima, Japan | no | no | no | no | no | no | no | no | no | no | no | no | no | no | No | no | SR | WG2 |
| Isao Myajima | Department of Clinical Nutrition, Chikamori Hospital, Kochi, Japan | no | no | no | no | no | no | no | no | no | no | no | no | no | no | No | ARDS Clinical Practice Guideline2026 | SR | WG3 |
| Masayuki Kaku | Department of Nutrition, NHO Kumamoto Medical Center, Kumamoto, Japan | no | no | no | no | no | no | no | no | no | no | no | no | no | no | No | no | SR | WG2 |
| Takaaki Inoue | Department of Nursing, Fukushima Medical University Hospital, Fukushima, Japan | no | no | no | no | no | no | no | no | no | no | no | no | no | no | No | no | SR | WG2 |
| Naoya Hashimoto | Department of Nursing, Hokkaido University of Science, Sapporo, Japan | no | no | no | no | no | no | no | no | no | no | no | no | no | no | No | no | SR | WG2 |
| Yusuke Miyazaki | Department of Emergency and Critical Care Medicine, Konan Medical Center, Kobe, Japan | no | no | no | no | no | no | no | no | no | no | no | no | no | no | No | no | SR | WG2 |
| Mizue Matsuo | Emergency and Critical Care Center, Kitakyushu General Hospital, Kitakyushu, Japan | no | no | no | no | no | no | no | no | no | no | no | no | no | no | No | no | SR | WG2 |
| Tomoki Yamada | Emergency and Critical Care Medical Center, Osaka Keisatsu Hospital, Osaka, Japan | no | no | no | no | no | no | no | no | no | no | no | no | no | no | No | no | SR | WG1 |
| Ichiro Hirayama | Department of Emergency Medicine, National Hospital Organization Saitama Hospital, Saitama, Japan | no | no | no | no | no | no | no | no | no | no | no | no | no | no | No | no | SR | WG1 |
| Jiro Ito | Department of Anesthesia and Critical Care, Kobe City Medical Center General Hospital, Kobe, Japan | no | no | no | no | no | no | no | no | no | no | no | no | no | no | No | no | SR | WG1 |
| Hiroyuki Tanaka | Department of Anesthesiology and Critical Care Medicine, Yokohama City University Graduate School of Medicine, Yokohama, Japan | no | no | no | no | no | no | no | no | no | no | no | no | no | no | No | no | SR | WG1 |
| Akihito Takemae | Department of Intensive Care, Gunma University Hospital, Maebashi, Japan | no | no | no | no | no | no | no | no | no | no | no | no | no | no | No | no | SR | WG1 |
| Kazushige Inoue | Department of Critical Care Medicine and Trauma, National Hospital Organization Disaster Medical Center, Tokyo, Japan | no | no | no | no | no | no | no | no | no | no | no | no | no | no | No | no | SR | WG2 |
| Kazushi Takayama | Department of Disaster and Emergency Medicine, Graduate School of Medicine, Kobe University, Kobe, Japan | no | no | no | no | no | no | no | no | no | no | no | no | no | no | No | no | SR | WG1 |
| Takahiro Hiruma | Department of Emergency and Critical Care Medicine, Southern Tohoku General Hospital, Fukushima, Japan | no | no | no | no | no | no | no | no | no | no | no | no | no | no | No | no | SR | WG1 |
| Takahiro Kawaji | Department of Anesthesiology and Critical Care Medicine, Fujita Health University, School of Medicine, Toyoake, Japan | no | no | no | no | no | no | no | no | no | no | no | no | no | no | No | no | SR | WG1 |
| Takayuki Irahara | Department of Emergency and Critical Care Medicine, Aichi Medical University, Aichi, Japan | no | no | no | no | no | no | no | no | no | no | no | no | no | no | No | no | SR | WG2 |
| COI, Conflicts of Interest; JSCNM, Japanese Society for Clinical Nutrition and Metabolism; JSPEN, Japanese Society for Parenteral and Enteral Nutrition Therapy; JSICM, Japanese Society of Intensive Care Medicine; JSPICC, Japanese Society of Pediatric Intensive and Critical Care; J-SSCG, Japanese Clinical Practice Guideline for Management of Sepsis and Septic Shock; J-ReCIP, Japanese Clinical Practice Guideline for Rehabilitation in Critically Ill Patients; JCCNG, Japanese Critical Care Nutrition Guideline; JRC, Japan Resuscitation Council; JATEC, Japan Advanced Trauma Evaluation and Care | | | | | | | | | | | | | | | | | | | |
|  |  |  |  |  |  |  |  |  |  |  |  |  |  |  |  |  |  |  |  |
|  |  |  |  |  |  |  |  |  |  |  |  |  |  |  |  |  |  |  |  |
|  |  |  |  |  |  |  |  |  |  |  |  |  |  |  |  |  |  |  |  |
| 【Policy of COI disclosure】 ・The Japanese Society of Intensive Care Medicine submitted this COI disclosure, based on the same policy issued by the Japanese Association of Medical Sciences. ・This group used the modified submission format provided by the Japanese Association of Medical Sciences. ・In accordance with these guidelines, organizations are only required to disclose COI that relate to associated companies or for-profit organizations as financial COI. ・We asked all members to submit their financial and academic COI for the past three years, in accordance with the current policy, shown in the last table. | | | | | | | | | | | | | | | | | | | |
|  |  |  |  |  |  |  |  |  |  |  |  |  |  |  |  |  |  |  |  |
| Standard of conflict of interest disclosure | | | | | | | | | | | | | | | | | | |  |
| Financial COI | A-1 | Presence of an officer or advisor of a for-profit company or organization, or received any remuneration (list those where the remuneration amount from one company/organization is 1 million yen or more per year) | | | | | | | | | | | | | | | | |  |
|  | A-2 | Holding of shares or profits obtained from those shares (profit from shares in the last year) (list those where the annual profit of one company is 1 million yen or more, or those who own more than 5% of shares) | | | | | | | | | | | | | | | | |  |
|  | A-3 | Remuneration paid as a patent royalty from a for-profit company or organization (list those where one patent royalty was 1 million yen or more) | | | | | | | | | | | | | | | | |  |
|  | A-4 | Remuneration, such as a daily allowance or lecture fee, paid for the time and labor requested by the researcher by a for-profit company or organization for attending meetings (e.g., presentation and advising) (list the lecture fee from one company/organization based on the annual total amount) | | | | | | | | | | | | | | | | |  |
|  | A-5 | Manuscript fee paid for writing (e.g., a pamphlet or roundtable article) created by a for-profit company or organization | | | | | | | | | | | | | | | | |  |
|  | A-6 | Research funds provided by a for-profit company or organization (list those where 1 million yen or more were actually allocated with a research contract from a one company/organization for medical research [e.g., joint research, contract research, or clinical trial] that allows the filer to substantially decide the purpose of use) | | | | | | | | | | | | | | | | |  |
|  | A-7 | Scholarship (incentive) donations provided by a for-profit company or organization (list donations over 1 million yen actually allocated to the individual filer or the course/field of laboratory with which the filer is affiliated, where they can substantially decide the purpose of use) | | | | | | | | | | | | | | | | |  |
|  | A-8 | Donation courses provided by companies (list donations over 1 million yen that are actually allocated and can be used for practical purposes) | | | | | | | | | | | | | | | | |  |
|  | A-9 | Other remuneration (e.g., travel and gifts that are not directly related to research) (list based on the annual remuneration received from one company/organization) | | | | | | | | | | | | | | | | |  |
|  | B-1 | Presence of an officer or advisor of a for-profit company or organization, or received any remuneration (list based on the annual remuneration from one company/organization) | | | | | | | | | | | | | | | | |  |
|  | B-2 | Holding of shares or profits obtained from those shares (profit from shares in the last year) (list the total annual profits of one company, or those holding 5% or more of shares) | | | | | | | | | | | | | | | | |  |
|  | B-3 | Remuneration paid as a patent royalty from a for-profit company or organization (list those where one patent royalty was 1 million yen or more) | | | | | | | | | | | | | | | | |  |
|  | C-1 | Research funds provided by a for-profit company or organization (list those where research contract money was actually allocated for medical research [e.g., joint research or contract research] based on one company/organization or contract, where the applicable head of the organization/department could substantially determine the purpose of use) | | | | | | | | | | | | | | | | |  |
|  | C-2 | Scholarship (incentive) donations provided by a for-profit company or organization (list those where the donation was actually allocated from one company/for-profit organization with the filer's research, and where the head of the research institution, hospital, faculty, center, or course could substantially determine the purpose of use) | | | | | | | | | | | | | | | | |  |
| Academic COI |  | Position higher than the academic society director or auditor | | | | | | | | | | | | | | | | |  |
|  |  | Involvement in the development of other guidelines | | | | | | | | | | | | | | | | |  |
